# Supplementary material for: Impact of obstetric unit closures, travel time and distance to obstetric services on maternal and neonatal outcomes in high-income countries: a systematic review
Source: BMJ Open. 2020 Dec 13;10(12):e036852. doi: 10.1136/bmjopen-2020-036852 (PMC7735086; doi:10.1136/bmjopen-2020-036852)
Supplement: Supplementary data [file bmjopen-2020-036852supp001.pdf]

Appendix 1: Current high-income countries (OECD) members

- Australia
- Austria
- Belgium
- Canada
- Chile
- Czech Republic
- Denmark
- Estonia
- Finland
- France
- Germany
- Greece
- Hungary
- Iceland
- Ireland
- Israël
- Italy
- Japan
- Korea
- Latvia
- Luxembourg
- Mexico
- Netherlands
- New Zealand
- Norway
- Poland
- Portugal
- Slovak Republic
- Slovenia
- Spain

- Sweden
- Switzerland
- Turkey
- United Kingdom
- United States

http://www.oecd.org/about/membersandpartners/
