# Supplementary material for: Impact of obstetric unit closures, travel time and distance to obstetric services on maternal and neonatal outcomes in high-income countries: a systematic review
Source: BMJ Open. 2020 Dec 13;10(12):e036852. doi: 10.1136/bmjopen-2020-036852 (PMC7735086; doi:10.1136/bmjopen-2020-036852)
Supplement: Supplementary data [file bmjopen-2020-036852supp002.pdf]

Appendix 2: MEDLINE search results in October 2017

1	Maternal Health Services/	12707
2	Delivery Rooms/	1417
3	Intensive Care Units, Neonatal/ and Intensive Care, Neonatal/	860
4	exp Hospitals/ and (exp Pregnancy/ or exp Delivery, Obstetric/ or Prenatal Care/ or Perinatal Care/ or Postnatal Care/)	10061
5	exp Hospitals/ and (foetal or fetal or foetus or fetus or newborn* or neonat* or infant* or baby or babies or maternal or maternity or pregnant or pregnancy or pregnancies or prenatal or pre-natal or antenatal or ante-natal or perinatal or peri-natal or postnatal or post-natal).ti,ab.	13308
6	(Community Health Services/ or Rural health Services/) and (exp Pregnancy/ or exp Delivery, Obstetric/ or Prenatal Care/ or Perinatal Care/ or Postnatal Care/)	2130
7	(Community Health Services/ or Rural health Services/) and (foetal or fetal or foetus or fetus or newborn* or neonat* or infant* or baby or babies or maternal or maternity or pregnant or pregnancy or pregnancies or prenatal or pre-natal or antenatal or ante-natal or perinatal or peri-natal or postnatal or post-natal).ti,ab.	2436
8	((maternity or obstetric?) adj3 (service? or hospital? or ward? or unit? or department? or dept? or centre? or center?)).ti,ab.	20646
9	((maternity or maternal or maternal health or obstetric) adj care).ti,ab.	8066
10	((delivery or birth*) adj (service? or hospital? or ward? or unit? or department? or dept? or centre? or center?)).ti,ab.	2990
11	((delivery or birth) adj care).ti,ab.	797
12	((foetal or fetal or foetus or fetus or newborn* or neonat* or infant* or baby or babies or maternal or maternity or pregnant or pregnancy or pregnancies or prenatal or pre-natal or antenatal or ante-natal or perinatal or peri-natal or postnatal or post-natal) adj3 (service? or hospital? or ward? or unit? or department? or dept? or centre? or center?)).ti,ab.	49515
13	((foetal or fetal or foetus or fetus or newborn* or neonat* or infant* or baby or babies or maternal or maternity or pregnant or pregnancy or pregnancies or prenatal or pre-natal or antenatal or ante-natal or perinatal or peri-natal or postnatal or post-natal) adj care).ti,ab.	29272
14	(((communit* or rural or district? or local*) adj3 (service? or hospital? or ward? or unit? or department? or dept? or centre? or center?)) and (foetal or fetal or foetus or fetus or newborn* or neonat* or infant* or baby or babies or maternal or maternity or pregnant or pregnancy or pregnancies or prenatal or pre-natal or antenatal or ante-natal or perinatal or peri-natal or postnatal or post-natal)).ti,ab.	7602
15	(((communit* or rural or district? or local*) adj care) and (foetal or fetal or foetus or fetus or newborn* or neonat* or infant* or baby or babies or maternal or maternity or pregnant or pregnancy or pregnancies or prenatal or pre-natal or antenatal or ante-natal or perinatal or peri-natal or postnatal or post-natal)).ti,ab.	141
16	(((level 1 or level i or level one or level 2 or level ii or level two or level 3 or level iii or level three) adj5 (service? or hospital? or ward? or unit? or department? or dept? or centre? or center? or care)) and (foetal or fetal or foetus or fetus or newborn* or neonat* or infant* or maternal or maternity or pregnant or pregnancy or prenatal or pre-natal or antenatal or ante-natal or perinatal or peri-natal or postnatal or post-natal)).ti,ab.	1408
17	1 or 2 or 4 or 5 or 6 or 7 or 8 or 9 or 10 or 11 or 12 or 13 or 14 or 15 or 16	108968
18	regional health planning/ or "catchment area (health)"/	12545

19	"Transportation of Patients"/	8903
20	Transportation/	8928
21	Health Services Accessibility/	65383
22	Travel/	23261
23	(travel* adj5 (distance or time)).ti,ab.	7255
24	("distance to" adj5 (hospital? or service? or unit? or ward? or centre? or center?)).ti,ab.	5849
25	("time to" adj5 (hospital? or service? or unit? or ward? or centre? or center?)).ti,ab.	37417
26	(("travel to" or "travel?ing to") adj5 (hospital? or service? or unit? or ward? or centre? or center?)).ti,ab.	1394
27	(("transfer to" or "transferred to") adj5 (hospital? or service? or unit? or ward? or centre? or center?)).ti,ab.	13476
28	(("transport to" or "transported to" or "transportation to") adj5 (hospital? or service? or unit? or ward? or centre? or center?)).ti,ab.	6259
29	(("access to" or accessibility) adj5 (hospital? or service? or unit? or ward? or centre? or center?)).ti,ab.	22957
30	(access* or travel* or distance* or transport* or transfer*).ti.	360710
31	((regional* or central or decentral*) adj5 (hospital? or service? or unit? or ward? or centre? or center?)).ti,ab.	36894
32	(regionali?e* or regionali?ation or centrali?e* or centrali?ation or decentrali?e or decentrali?ation).ti,ab.	18456
33	(geographic adj5 (hospital? or service? or unit? or ward? or centre? or center?)).ti,ab.	1951
34	((geographic* or district? or residence) adj5 area?).ti,ab.	24860
35	catchment*.ti,ab.	10031
36	((small or medium or large) adj2 (hospital? or unit?)).ti,ab.	14557
37	(small volume or medium volume or high volume).ti,ab.	18240
38	Health Facility Closure/	2317
39	(close or closed or closing or closure? or discontinu*).ti,ab.	609722
40	(preclos* or pre-clos* or postclos* or post-clos*).ti,ab.	406
41	downsiz*.ti,ab.	1966
42	(midwifery led adj2 (service? or unit? or ward? or centre? or center?)).ti,ab.	46
43	(midwifery adj (service? or unit? or ward? or centre? or center?)).ti,ab.	352
44	((consultant led or obstetrician led) adj2 (service? or unit? or ward? or centre? or center?)).ti,ab.	132
45	((consultant or obstetrician) adj (service? or unit? or ward? or centre? or center?)).ti,ab.	159
46	(referral* adj5 (service? or unit? or ward? or centre? or center?)).ti,ab.	28869
47	("model of care" or care model* or "model of delivery" or delivery model* or "model of service" or service model*).ti,ab.	12723
48	18 or 19 or 20 or 21 or 22 or 23 or 24 or 25 or 26 or 27 or 28 or 30 or 31 or 32 or 33 or 34 or 35 or 36 or 37 or 38 or 39 or 40 or 41 or 42 or 43 or 44 or 45 or 46 or 47	1251197
49	17 and 48	15581
