# Supplementary material for: Impact of obstetric unit closures, travel time and distance to obstetric services on maternal and neonatal outcomes in high-income countries: a systematic review
Source: BMJ Open. 2020 Dec 13;10(12):e036852. doi: 10.1136/bmjopen-2020-036852 (PMC7735086; doi:10.1136/bmjopen-2020-036852)
Supplement: Author's manuscript [file bmjopen-2020-036852.draft_revisions.pdf]

BMJ Open is committed to open peer review. As part of this commitment we make the peer review history of every article we publish publicly available.

When an article is published we post the peer reviewers' comments and the authors' responses online. We also post the versions of the paper that were used during peer review. These are the versions that the peer review comments apply to.

The versions of the paper that follow are the versions that were submitted during the peer review process. They are not the versions of record or the final published versions. They should not be cited or distributed as the published version of this manuscript.

BMJ Open is an open access journal and the full, final, typeset and author-corrected version of record of the manuscript is available on our site with no access controls, subscription charges or pay-per-view fees (http://bmjopen.bmj.com).

If you have any questions on BMJ Open's open peer review process please email
info.bmjopen@bmj.com

BMJ Open

The impact of obstetric unit closures, travel time and distance from women's home to obstetric services on maternal and neonatal outcomes: a systematic review

Journal:	*BMJ Open*
Manuscript ID	bmjopen-2020-036852
Article Type:	Original research
Date Submitted by the Author:	13-Jan-2020
Complete List of Authors:	Malouf, Reem; University of Oxford, Policy Research Unit in Maternal Health and Care, National Perinatal Epidemiology Unit, Nuffield Department of Population Health<br>Smith, Claire; University of Birmingham, Institute of Applied Health Research, College of Medical and Dental Sciences<br>Henderson, Jane; University of Oxford, Policy Research Unit in Maternal Health and Care, National Perinatal Epidemiology Unit, Nuffield Department of Population Health<br>Opondo, Charles; University of Oxford, Policy Research Unit in Maternal Health and Care, National Perinatal Epidemiology Unit, Nuffield Department of Population Health;<br>Brocklehurst, Peter; University of Birmingham, Institute of Applied Health Research, College of Medical and Dental Sciences<br>Alderdice, Fiona; University of Oxford, Policy Research Unit in Maternal Health and Care, National Perinatal Epidemiology Unit, Nuffield Department of Population Health<br>Phalguni, Angaja; University of Birmingham, Institute of Applied Health Research, College of Medical and Dental Sciences<br>Dretzke, Janine; University of Birmingham, Institute of Applied Health Research, College of Medical and Dental Sciences
Keywords:	Organisation of health services < HEALTH SERVICES ADMINISTRATION & MANAGEMENT, EPIDEMIOLOGY, OBSTETRICS

SCHOLARONE™
Manuscripts

I, the Submitting Author has the right to grant and does grant on behalf of all authors of the Work (as defined in the below author licence), an exclusive licence and/or a non-exclusive licence for contributions from authors who are: i) UK Crown employees; ii) where BMJ has agreed a CC-BY licence shall apply, and/or iii) in accordance with the terms applicable for US Federal Government officers or employees acting as part of their official duties; on a worldwide, perpetual, irrevocable, royalty-free basis to BMJ Publishing Group Ltd ("BMJ") its licensees and where the relevant Journal is co-owned by BMJ to the co-owners of the Journal, to publish the Work in this journal and any other BMJ products and to exploit all rights, as set out in our licence.

The Submitting Author accepts and understands that any supply made under these terms is made by BMJ to the Submitting Author unless you are acting as an employee on behalf of your employer or a postgraduate student of an affiliated institution which is paying any applicable article publishing charge ("APC") for Open Access articles. Where the Submitting Author wishes to make the Work available on an Open Access basis (and intends to pay the relevant APC), the terms of reuse of such Open Access shall be governed by a Creative Commons licence – details of these licences and which Creative Commons licence will apply to this Work are set out in our licence referred to above.

Other than as permitted in any relevant BMJ Author's Self Archiving Policies, I confirm this Work has not been accepted for publication elsewhere, is not being considered for publication elsewhere and does not duplicate material already published. I confirm all authors consent to publication of this Work and authorise the granting of this licence.

For peer review only

Title: The impact of obstetric unit closures, travel time and distance from women's home to obstetric services on maternal and neonatal outcomes: a systematic review

Reem Malouf[1*], Claire Tomlinson[2], Jane Henderson[1], Charles Opondo[1], Peter Brocklehurst[1], Fiona Alderdice[1], Angaja Phalguni[2], Janine Dretzke[2]

[1]Policy Research Unit in Maternal Health and Care, National Perinatal Epidemiology Unit, Nuffield Department of Population Health, University of Oxford, Old Road Campus, Old Road, Oxford, UK

[2]Institute of Applied Health Research, College of Medical and Dental Sciences, University of Birmingham

*** Corresponding author**

reem.malouf@npeu.ox.ac.uk

For peer review only

For peer review only - http://bmjopen.bmj.com/site/about/guidelines.xhtml

Abstract

Objectives: To systematically review a)The effect of obstetric unit (OU) closures on maternal and neonatal outcomes in the surrounding or comparable population; b) The association between travel distance/time to an OU and maternal and neonatal outcomes.

Design: Systematic review of any quantitative study design with a comparison group.

Data sources: Embase, Medline, PsycINFO, Applied Social Science Index and Abstracts and Cumulative Index to Nursing and Allied Health databases and grey literature were all searched.

Methods: Eligible studies explored the impact of closure of an OU or the effect of travel distance/time on pre-specified maternal or neonatal outcomes. Only studies of women giving birth in high-income countries with universal health coverage of maternity services comparable to the UK were included. Identification of studies, extraction of data and risk of bias assessment were undertaken by at least two reviewers independently. The risk of bias checklist was adapted from Effective Practice and Organisation of Care (EPOC) and Newcastle-Ottawa scale (NOS).

Results: 31 studies met the inclusion criteria and one study included information on both OU closure and travel distance. There was limited or no evidence on the impact of the exposures on the majority of maternal and neonatal outcomes. Ten studies provided information on OU closures: babies born before arrival (BBA) were significantly increased following closure (4/6 studies). Seven studies reported on travel distances: all four studies that reported on BBA found a significant increase in BBA with longer travel distance. Fifteen studies reported on travel time: seven studies provided information on perinatal mortality and the findings were inconsistent. Association between increased travel time and increased risk of BBA was found in 5/6 studies.

Conclusion: It is not possible to conclude from this review whether OU closure, increased travel distances or times are associated with worse outcomes for the mother or the baby.

Prospero registration number: CRD42017078503.

For peer review only - http://bmjopen.bmj.com/site/about/guidelines.xhtml

Strengths and Limitations

- This work is the first to synthesis systematically the current evidence relevant to the closure of obstetric units and the impact of travel distance and travel time.
- A wide and sensitive search strategy to identify the relevant literature was used.
- The heterogeneity across the included studies precluded the conduct of any form of meta-analysis.

For peer review only

For peer review only - http://bmjopen.bmj.com/site/about/guidelines.xhtml

Background

Closure of small obstetric units (OUs) and centralisation of obstetric services in larger units has been proposed to increase levels of consultant obstetrician cover to improve safety and limit costs. However, closure of OUs or conversion of OUs to midwifery-led units/community-based services potentially leads to an increase in travel distance or time for women in labour from their home to the nearest obstetric unit. Increases in travel time could potentially increase the risk of adverse birth outcomes, specifically a rise in the risk of babies born before arrival (BBA, also referred to as unplanned out-of-hospital births) and other adverse birth outcomes such as maternal and neonatal death. However, uncertainty remains about the association between OU closure, prolonged time or distance to OUs and adverse birth outcomes.

Travel time and distance are widely used as measures to explore the geographical accessibility of health services (1). In a systematic review (2) the association between travelling further to healthcare facilities and having worse health outcomes was established, but the review did not include studies of maternity care. The impact of OU closure and increase in travel time/distance to the OU on perinatal and maternal outcomes have not been systematically assessed. One review (3) evaluating the effects of regionalisation of perinatal services has been published. This concluded that regionalisation programmes appeared to be correlated with improvements in perinatal outcomes but that the evidence was weak. A narrative review (4) included 10 studies that explored travel time and distance to and between maternity services and adverse birth outcomes to inform the consultation on maternity services in Wales. The review was limited to studies reported in English and there was no clear association between travel distance or time and adverse birth outcomes. In this review we aimed to systematically identify, critically appraise and synthesise the evidence relating to:

a) The effect of OU closures on maternal and neonatal outcomes in the surrounding or a comparable population.

For peer review only - http://bmjopen.bmj.com/site/about/guidelines.xhtml

b) The association between travel distance or time to an OU and maternal and neonatal outcomes.

Review method

The protocol was registered with Prospero (registration number CRD42017078503) and the Meta-Analyses and Systematic Reviews of Observational Studies MOOSE reporting guideline was followed (5).

Criteria for considering studies for this review

Types of studies

Any quantitative study design with a comparison group was eligible for inclusion. Studies were included from 1990 onwards. The year 1990 was chosen as a cut-off date because significant advances were made in neonatal care in the early 1990s, such as surfactant therapy, assisted ventilation, prophylactic infection control and antenatal steroid therapy, which impacted on the delivery of maternity services (6). The quantitative components of mixed methods studies were also eligible. Studies were eligible for inclusion if they:

- Explored the impact of closure of an OU on maternal or neonatal outcomes either in a before-and-after comparison (same population catchment area), or a geographical comparison of different areas (comparable populations)

And/or

- Compared maternal and neonatal outcomes after an OU closure and retention or creation of midwifery led units to replace the OU
- Explored the effect of travel time and/or distance on maternal and neonatal outcomes providing at least two travel times and/or distances from women's home to the nearest OU

For peer review only - http://bmjopen.bmj.com/site/about/guidelines.xhtml

For peer review only

- Explored maternal and neonatal outcomes following maternal transfer from planned or unplanned home birth to the nearest maternity centre.

We included studies of women giving birth in high-income (OECD) countries with universal health coverage (UHC) of maternity services comparable to the UK. The list of OECD countries is shown in appendix 1. UHC is defined as health care that meets everyone's right to access high quality essential health services where and when they need them without financial difficulty (7).

Types of exposures

OU closure: the closure of an OU was compared to no closure of an OU for the same or comparable geographical catchment areas prior to the closure. For a study comparing different geographical areas affected by the closure of an obstetric unit, the least affected area was used as a control group. For the purpose of this review we used the definition of an OU used in the Birthplace Research programme in England (8), which defined an OU as "a clinical location in which care is provided by a team, with obstetricians taking primary professional responsibility for women at high risk of complications during labour and birth. Midwives offer care to all women in an OU, whether or not they are considered at high or low risk, and take primary responsibility for women with straightforward pregnancies during labour and birth. Diagnostic and treatment medical services including obstetric, neonatal and anaesthetic care are available on site, 24 hours a day"(8)(P12).

Travel distance or time to the nearest OU: a shorter travel distance or time was compared with a longer travel distance or time. We used the definition of a shorter or a longer time or distance as indicated by the included studies. When a study compared different travel times or distances to the nearest OU, all of the comparators were sought for inclusion and those with the shortest travel distance or time were used as the control group.

The following types of studies were excluded:

For peer review only - http://bmjopen.bmj.com/site/about/guidelines.xhtml

For peer review only

- Studies comparing maternal and or neonatal outcomes based on hospital size, level of neonatal intensive care, type of hospital or model of care (e.g. caseload midwifery care versus consultant care)
- Studies on regionalisation of neonatal care (number of centres with neonatal intensive care (NICUs)
- Studies where a proximity rather than the actual travel time or travel distance was given (e.g. rural vs urban, remote vs very remote areas)
- Studies which did not report at least one of the outcomes.

Review outcomes

The following outcomes were pre-defined in the study protocol:

Maternal outcomes

Maternal mortality (MM), caesarean section (CS) (overall, emergency or intrapartum), severe perineal trauma(including 3rd and 4th degree tears) , postpartum haemorrhage (PPH), maternal admission to intensive care unit (ICU) and maternal blood transfusion.

Neonatal outcomes

Stillbirth (SB) (overall or intrapartum), neonatal mortality (NM), perinatal mortality (PM), infant mortality (IM), babies born before arrival (BBA), neonatal unit admission (NNU), Apgar score and hypoxic-ischemic encephalopathy (HIE).

Review methods

A comprehensive search strategy was developed in collaboration with an information specialist (NR). We searched Embase, Medline, PsycINFO, Applied Social Science Index and Abstracts (ASSIA) and Cumulative Index to Nursing and Allied Health (CINAHL) databases (from 1990 to February

For peer review only - http://bmjopen.bmj.com/site/about/guidelines.xhtml

For peer review only

2019). We also searched the Grey literature in the databanks of British Library EThOS, Open Grey and ProQuest Dissertations & Theses Global. NHS Trusts and Health Boards in the UK were also contacted where we had been able to identify an OU closure to request information about any evaluations that were conducted. The references of eligible studies and relevant reviews were also checked to identify additional studies not retrieved by the search. Searches were based on index and text words relating to the population/setting (e.g. maternity service, pregnancy, neonatal) and exposures (e.g. travel/distance or closure/regionalisation). Due to the variable nature or terms and indexing used, the strategy was kept broad by using a range of alternate terms and not limiting by outcome. No language restriction was applied. A sample search strategy for MEDLINE is shown in appendix 2.

At least two reviewers (RM, CT, AP, FA, JH) independently screened the references for relevance against the review eligibility criteria using Eppi-reviewer software (version 4). Full-text study screening was also performed by at least two reviewers (RM, CT, AP, JH, FA). When disagreement regarding study eligibility occurred the differences of opinion were resolved through discussion and consensus within the review team. We contacted several authors of relevant studies published as abstracts for further information. Data extraction and risk of bias assessment were undertaken by at least two reviewers. The risk of bias checklist was adapted from the Effective Practice and Organisation of Care (EPOC) (9) and the risk of bias in non-randomised studies of interventions Newcastle-Ottawa scale (NOS) (for case-control studies) (10). Risk of bias assessment included selection of study groups, measurement of exposure and outcomes, missing data and appropriateness of analysis (e.g. logistic regression analysis). For case-control studies, selection and comparability of cases and controls were also considered. The review team rated the quality of evidence for each domain in the tool as low, high or unclear risk of bias, or yes, no and unclear in meeting quality criteria.

For peer review only - http://bmjopen.bmj.com/site/about/guidelines.xhtml

Results were synthesised narratively and the key findings tabulated. The included studies varied in their study design, categories of exposure, outcomes reported, whether adjusted or unadjusted results were presented and factors adjusted for. Due to this clinical and methodological variability quantitative pooling was not feasible. Pre-specified sub-groups were risk-status of woman (low versus high), parity, gestational age, UK studies compared with non-UK studies, and planned vs unplanned CS; formal sub-group analyses were however not possible . Evidence regarding OU closure, travel distance and travel time is reported separately, and by outcome. We have highlighted where crude (unadjusted) odds ratios (cOR) and adjusted odds ratios (adj OR) have been reported.

Patient and Public Involvement

The need for a broad systematic review of OU closure, travel time and distance to obstetric unit was identified through discussion with our stakeholder groups included discussion with our Parent, Patient, and Public Involvement (PPPI) Stakeholders Network. Dissemination of findings to stakeholders will be through plain language summaries developed with members of our PPPI stakeholder network.

Search results

Searches of bibliographic databases and other sources yielded 13,271 unique references (Figure 1). The eligibility of 291 full-text articles were assessed independently. 260 articles were excluded for various reasons, including: studies conducted in low/middle income countries, comparing different models or levels of maternity care, assessing women’s transfer from primary to secondary maternity centres, or not providing quantifiable measures of travel time/distance (full list available from authors). Thirty-one studies, reported in 35 papers, met the review eligibility criteria (Fig 1). One study (11), included information on both OU closure and travel distance. Ten studies provided information on OU closures, seven studies compared different travel distances from women’s homes

For peer review only - http://bmjopen.bmj.com/site/about/guidelines.xhtml

to the nearest OU; and 15 studies compared different travel times from women's homes to the nearest OU.

Review results

Evidence from obstetric unit (OU) closures

A detailed description of the ten included OU closure studies is presented in Table 1A. Three studies were from the UK, two as abstracts only(12, 13); and one unpublished data series from East Lancashire Hospitals National Health Services (NHS) Trust, UK. There appeared to be overlap between populations reported in two studies (East Lancashire Hospitals NHS Trust) (12). Three studies were from Scandinavia (14-16), three from Canada (17-19) and one from France (11).

All studies compared rates of adverse birth outcomes before and after OU closure or centralisation of obstetric services. Four studies were published after 2014 (12, 13, 15, 18); the earliest was from 1990 (19). Three studies included all births (15, 16, 19); the other studies varied in their eligibility criteria, e.g. restricting the analysis to singletons, live births, various gestational ages and birthweight, hospital births or location. Reporting of eligibility criteria and participant characteristics across studies was inconsistent (Table 1A).

Two studies from the UK (East Lancashire Hospitals NHS Trust) (12); examined the impact of the amalgamation of two OUs. The other studies relate to regional or national reconfigurations or centralisation of services with varying numbers of units closing.

Risk of bias assessment

Risks of bias related to a lack of reporting of whether changes over time (other than closure/reconfiguration) could have influenced the findings, with only one (16) of ten studies reporting that temporal variation was adjusted for in the analysis (Table 1B). Further, six out of ten

For peer review only - http://bmjopen.bmj.com/site/about/guidelines.xhtml

studies did not adjust results for potential confounding factors or did not provide sufficient information to gauge the completeness of data. Only four studies reported and used appropriate data analysis methods. Other potential sources of bias (e.g. relating to selection, exposure and outcome) were less of a concern due to the use of routinely collected registry data before and after the closure and the objective nature of most outcomes.

Findings

A summary of maternal and neonatal outcomes is presented in Table 1C.

Maternal outcomes

Maternal mortality (MM)

In the two studies that reported maternal mortality (13, 18), the number of deaths (<5) was too low to allow comparisons between the pre- and post-closure groups .

Caesarean section (CS) (overall or intrapartum)

Total CS rates were reported in four studies (12, 14, 18, 19). One UK study,(12) reported a decline in CS rates following the amalgamation of two units from 26.1% to 21.5%

A Norwegian study (14), reported an increase in CS rates from 13.1% to 16.4% following OU closure, (cOR=1.31, 95% 1.27, 1.35) as did two Canadian studies (19)cOR= 1.13, 95%CI: 1.09, 1.18) and (cOR= 1.10, 95%CI: 1.01, 1.19)(18). No adjusted results were reported.

Emergency caesarean section (CS)

Emergency CS rates were reported in one UK study (13), which found no difference before/after the amalgamation of two OUs (cOR=0.95, 95%CI: 0.86, 1.05).

Severe perineal trauma (3rd or 4th degree tear).

For peer review only - http://bmjopen.bmj.com/site/about/guidelines.xhtml

For peer review only

Two studies (13, 18) reported this outcome and found no statistically significant difference between the before/after closure groups. The incidence of the outcome in both studies was low (<3%).

Postpartum haemorrhage – No studies reported this outcome.

Maternal admission to intensive care unit (ICU)

Two studies (13, 18) found no significant difference before/after the amalgamation of two OUs on the number of women requiring admission to ICU (cOR= 0.80, 95%CI: 0.44, 1.46) (13). The numbers in one study (18) were too small (<5) to allow a comparison.

Maternal blood transfusion

One study (18) found no significant differences before/after OU closure (cOR=0.82, 95%CI: 0.55, 1.21). The incidence of the outcome was low (<1% of women).

Neonatal outcomes

Stillbirth (SB) (overall or intrapartum)

Three studies examined the impact of OU closure on stillbirth. One unpublished UK study (East Lancashire Hospitals NHS Trust) showed a statistically significant reduction in stillbirths over the period after the amalgamation of two units (cOR=0.61, 95%CI: 0.47, 0.78). Similar findings were seen from Canada (20) during post closure years (cOR=0.80, 95%CI 0.67, 0.96). The third study from the UK (13) found no difference in stillbirth rates after OU closure.

Neonatal mortality (NM)

Three studies reported this outcome. Two studies from the UK (East Lancashire Hospitals NHS Trust) (13), showed no statistically significant difference in the rate of NM in the years after OU closure (cOR= 1.33, 95%CI: 0.81, 2.17; cOR= 0.80, 95%CI: 0.29, 2.26). A study from Norway (15) also reported no difference (no OR presented).

Perinatal mortality (PM)

For peer review only - http://bmjopen.bmj.com/site/about/guidelines.xhtml

For peer review only

Two studies reported this outcome. In a study from Norway (14), PM was significantly lower following OU closure (cOR= 0.76, 95%CI: 0.58, 0.98). A Canadian study (20) also reported a significant reduction in PM after OU closure (cOR= 0.75, 95%CI: 0.64, 0.87).

Infant mortality (IM)

One study (15) reported this outcome, IM rates were "not statistically elevated" after the closure of thirteen hospitals in Norway.

Born before arrival (BBA)

Six studies reported this outcome, with four suggesting a statistically significant increase in BBA following OU closure. Data from East Lancashire Hospitals NHS Trust, showed the BBA rate almost doubled over the 10 year period (cOR= 1.88, 95%CI:1.25, 2.82). Studies from Norway and Finland also found that the BBA rate increased over a similar period (cOR=1.8, 95%CI: 1.6, 1.9 and cOR=4.14, 95%CI: 3.16, 5.41 respectively). A Canadian study found that the BBA rate trebled over a 16 year period (cOR= 3.47, 95%CI 2.31, 5.20). One UK study(13) found no statistically significant change (cOR= 1.28, 95%CI: 0.63, 2.60) and in one French study (11), there was weak evidence of a small reduction in the adjusted risk of BBA in communities affected by OU closure (adj. OR 0.91, 95%CI 0.84, 1.00).

Neonatal Unit admission (NNU)

One Canadian study (18) suggested a significant reduction in NNU admission following OU closure (cOR= 0.39, 95%CI 0.25, 0.60).

Apgar score

One Canadian study (18) found no statistically difference in 5 minute Apgar score of less than 7 before and after OU closure (cOR= 1.13, 95%CI: 0.82, 1.55)

Hypoxic-Ischemic Encephalopathy (HIE) – No studies reported this outcome.

For peer review only - http://bmjopen.bmj.com/site/about/guidelines.xhtml

Evidence from travel distance studies

Description of included studies

Seven studies described the effect of travel distance to the nearest OU on maternal and neonatal outcomes (Table 2A). All were published in full between 1991 and 2015. The earliest study (21) was conducted in the UK, three more recent studies were conducted in France (11, 22, 23), and one each from Norway (24), Finland (25), and Canada (26). Four were retrospective population-based cohort studies, and three were case-control studies. The eligibility criteria varied across studies. Pasquier et.al. (22), included a group with special needs in the form of babies with congenital malformations. Only singleton live births were included in two studies (11, 26).

Travel distance was estimated using geographical mapping software in all studies. However, only three studies (11, 21, 22, 25) measured the actual distance from women's homes to the nearest OU. In two studies (23, 24, 26) a central geographical point for the postcode or municipality was used to estimate distances and in one study the distance was self-reported (24) Additionally, the studies differed regarding their distance categories, which ranged from 2km to 150km (Table 2A).

Risk of bias assessment

The main risk of bias concerns related to the measurement of exposure, as three studies (21, 23, 24, 26) did not calculate the distance from the woman's home but used a central point instead or self-reported distance (Table 2B). Another study (21), measured distance between women homes and hospital using a straight line. Further risk of bias related to a lack of comparability between study groups in the three case-control studies (21, 24, 25) , a lack of adjustment for confounders in two studies and missing data in two studies (24, 25). There were no risk of bias concerns relating to sample selection in the cohort studies or outcome measurement.

Findings

Maternal outcomes

For peer review only - http://bmjopen.bmj.com/site/about/guidelines.xhtml

For peer review only

Maternal mortality (MM)

In one case-control study from Finland (25), no maternal deaths were reported in either group (Table 2C).

Caesarean section (CS) (overall, or intrapartum)

One study from Canada (26) found no statistically significant differences in CS rates with increasing distance (<50k, 50-150k, >150k) based on both crude and adjusted results.

Emergency Caesarean section (CS)

The same study from Canada (26) reported no significant difference in emergency CS rates between women living at different distances from an OU based on crude ORs.

No studies reported on *severe perineal trauma, postpartum haemorrhage, maternal admission to intensive care unit (ICU) or maternal blood transfusion.*

Neonatal outcomes

Stillbirth (SB) (overall or intrapartum)

Two cohort studies (23, 26) reported this outcome. A Canadian study (26) included births to women aged over 35 years who lived <50km, 50-150km and >150 km from the OU. Stillbirth rates did not change by distance category in the adjusted analysis. A French study (23) reported stillbirth rates at different distances (<5 km, 5-15, 15-30, 30-44, 45+ km) from mother's municipality of residence to the closest OU. The crude odd ratios showed women living at 30-44, 15-29 and 5-14km from an OU, had a statistically significantly lower rate of stillbirth compared to women living <5 km from an OU; (5-14 km vs <5 km, cOR = 0.87, 95%CI: 0.85, 0.90; 15-29 km vs <5 km; cOR = 0.85, 95%CI: 0.82, 0.88; 30-44 km vs <5 km, cOR= 0.85, 95%CI: 0.81, 0.90l). The findings still hold for the adjusted analysis (limited data reported). However, the crude and adjusted analysis showed no significant difference in risk of stillbirth for individuals resident 45+ km from an OU compared with <5km.

For peer review only - http://bmjopen.bmj.com/site/about/guidelines.xhtml

For peer review only

Neonatal mortality (NM)

Two French cohort studies (22, 23) reported this outcome. One study (22) examined the distance from women's home to the nearest OU with neonatal surgical facilities for 706 fetuses with severe malformations. Analyses adjusted for malformation type, number of malformations, amniotic fluid anomaly, previous anomaly in the family and parity showed no association between NM and distance (<11 km vs 11-50 km, adj OR = 0.89, 95%CI: 0.34, 2.88; <11 km vs > 50 km, adj OR= 1.37, 95%CI: 0.49, 3.86). The other study (23) included all births and found that, NM rates were significantly higher for women living at <5 km compared to 5 -44 km away from an OU (23); (5-14 km vs < 5 km, cOR= 0.78, 95%CI: 0.75, 0.81; 15-29 km vs < 5 km cOR = 0.81, 95%CI 0.78, 0.85; 30-44 km vs < 5 km, cOR= 0.78, 95%CI 0.72, 0.84; ≥ 45 km vs < 5 km, cOR= 0.82, 95%CI 0.70, 0.96). In this latter study, the NM of babies born before arrival was also explored. For the BBA group, there were statistically significant increases in the risk of NM when women had to travel 45km or more to an OU in comparison to <5 km (≥ 45 km vs < 5 km, cOR= 2.45, 95%CI 1.14, 5.25).

Perinatal mortality (PM)

A study from Canada (26), reported that PM risk increased with travel distance in an adjusted model (<50 km v 50-150 km adjOR=1.53, 95%CI: 1.1-2.12; <50 km >150km adjOR=3.06, 95%CI 2.20,4.24).

Infant mortality (IM) - No studies reported this outcome.

Born before arrival (BBA)

Three case control studies (21, 24, 25), and one cohort study (11) reported this outcome. All four studies reported a significant increase in BBA rate with longer travel distance, although only two reported adjusted analyses (11, 25). In the UK study (21), the risk of BBA increased 12 fold for women living >7 km from the OU compared to women living <2 km away (cOR = 12.5, 95%CI: 4.02, 36.01). The risk of BBA increased significantly for women living >13 km from an OU in a Norwegian

For peer review only - http://bmjopen.bmj.com/site/about/guidelines.xhtml

study (24) (cOR= 1.81, 95%CI: 1.03, 3.18). The Finnish study (25) reported a fivefold increased risk of BBA for women living >35 km from the OU compared to <35 km (adj OR 5.02, 95%CI: 1.80, 14.04).

In France (11), the rate of BBA significantly increased with longer distances and it tripled for all women living 45+ km from the OU compared to women living <5km away (cOR: 3.77, 95%CI: 3.23, 4.39). The association persisted in an adjusted analysis which included women of parity three or higher and living 45+ km from the OU, who had a six fold increased risk of BBA compared to women living <5 km away and of parity one or two (adj OR= 6.49, 95%CI: 4.92, 8.48).

Neonatal unit admission (NNU)

A study from Canada (26) reported an increase in NNU admission for births to women living >150 km from an OU compared to those living <50 km away (6.8% vs 4.8%).

Apgar score – No studies reported this outcome.

Hypoxic-Ischemic Encephalopathy (HIE) – No studies reported this outcome.

Evidence from travel time studies

Description of included studies

We included 15 studies relating to travel time from a woman's home to OU, all detailed in Table 3A. Two studies (one reported as an abstract only) were conducted in the UK (27-29), three studies in France (30-32), three studies (reported in five articles) in the Netherlands (33-37), one study reported in two articles from Norway (38, 39), five studies in Canada (40-44) and one study in Japan (45).

Eleven studies were of a retrospective cohort design, one was a prospective cohort study (37), one was a before-and-after design (45) and two were case-control studies (31, 32). All the studies clearly stated the eligibility criteria. Only singleton births were included in five studies (28-30, 33-36, 40).

For peer review only - http://bmjopen.bmj.com/site/about/guidelines.xhtml

One study (37) specifically enrolled women with postnatal haemorrhage after home birth, and one study (40) focussed on planned home birth regardless of the actual place of birth.

The studies were heterogeneous in their travel time intervals. With the exception of one study in Canada (40), longer time cut-off points were examined in Norway, Japan and Canada compared to the ones in Europe. Travel duration was estimated using geographical mapping software in all studies. However, most studies estimated travel duration to and from central points within areas rather than actual addresses.

Risk of bias assessment

Quality assessment and supported explanations for each of the risk of bias domains are presented in Table 3B. With the exception of Stolp et al (37), sample selection and measurement of outcomes were considered low risk across all studies as such data were obtained from national databases and birth registries. The two case and control studies were appropriately selected and defined, however the case and control groups were not comparable in both studies (eg difference in antenatal care attendance and socio-demographics). Eight studies (27, 32-40), were considered at low risk of exposure measurement bias, as the women's actual place of residence was used to estimate travel time to nearest OU. The risk of attrition bias was low for the majority of the included studies. Similarly, appropriateness of analyses and adjustment for potential confounders were found in the majority of studies.

Findings

Maternal outcomes

Maternal mortality - no studies reported this outcome

Caesarean section (overall, or intrapartum)

Five Canadian studies (40-44) reported CS rates (Table 3C). Across three studies (41-43), crude OR for CS rates were higher among women who lived closer to OUs with CS rates highest for women

For peer review only - http://bmjopen.bmj.com/site/about/guidelines.xhtml

living less than one hour away compared with other categories (1-2 hours, 2-4 hours and > 4 hours). One study (44) included women who had a midwife involved in their care, and found no significant differences in CS rates for women living 1-2 hours and more than 2 hours away compared to within an 1 hour of an OU (1-2 vs < 1 hr, cOR= 1.23, 95%CI 0.80, 1.91 and>2 hrs vs < 1 hr, cOR=1.11, 95%CI: 0.71, 1.72). A further study (40) showed also a higher CS rate among women who planned a home birth and lived less than half an hour away from OU services (>30 mins vs ≤ 30 mins,(cOR=0.74, 95%CI 0.59, 0.92).

Emergency Caesarean section

Shorter travel time to an OU was associated with a statistically significant higher emergency CS rate in one Canadian study (43) (> 1hr vs < 1 hr, cOR= 0.80, 95%CI: 0.75, 0.86).

Severe perineal trauma – No studies reported this outcome.

Postpartum haemorrhage (PPH)

One Canadian study found the risk of PPH was significantly higher for women who lived more than one hour away from obstetric services compared to women who lived less than one hour away (42) (> 1hr vs < 1 hr, cOR=1.27, 95%CI: 1.13, 1.43).

Maternal admission to intensive care unit

One study from the Netherlands (37) involved women who had a PPH after midwifery-supervised home births and examined adverse maternal outcomes associated with travel time longer than 45 minutes to hospital. No difference was found in the number of women admitted to ICU who travelled more than 45 minutes compared with < 45 travel time to hospital, but the numbers of events were few.

Maternal blood transfusion

For peer review only - http://bmjopen.bmj.com/site/about/guidelines.xhtml

For peer review only

One study from the Netherlands (37) found no significant difference in the median number of units of blood transfused to women who travelled more than 45 minutes to an OU compared to <45 minutes travel time.

Neonatal outcomes:

Stillbirth SB (overall or intrapartum)

Three studies examined the association between increasing travel time and SB, one study each from the UK (28, 29), France (30) and Canada (42).

In the UK study (28, 29), there was no association between travel time and SB when analysing all women (adjOR-1.13, 95%CI:0.98, 1.30). However subgroup analyses of term pregnancies and those to nulliparous women showed a significant increase in the risk of SB with every 15 minute increase in travel time to the OU for term pregnancies (adjOR=1.36, 95%CI-1.17, 1.59) and those to nulliparous women (adjOR=1.21, 95%CI:1.02,1.44). The other two studies (30, 42) found no significant increase in the incidence of SB with increasing travel time .

Neonatal mortality (NM)

Five studies examined the association between travel time and NM, two from the UK (27-29), one from the Netherland (33-35), one from Canada (42) and one from Japan (45).

The adjusted analysis in one UK study(27) showed no statistically significant association between NM and travel time. The adjusted analyses in the other UK study (29) showed a significant increase in early and late NM, with every 15 minutes increase in travel time for all women travelling from home to the OU (adjOR= 1.13, 95%CI: 1.07, 1.20) and (adjOR= 1.15, 95%CI 1.05, 1.26) respectively. Subgroup analysis for nulliparous women showed a statistically significant increased risk of early

For peer review only - http://bmjopen.bmj.com/site/about/guidelines.xhtml

neonatal death associated with every 15 min increase in travel time from home to the OU (adjOR= 1.15, 95%CI: 1.06, 1.25). For term births, late (but not early) NM increased significantly with every 15 mins increase travel time from home to the OU (adjOR= 1.34, 95%CI: 1.13, 1.59).

In one study from the Netherlands (33), a travel time of 20 minutes or more was associated with a significant increase in the combined intrapartum, early and late NM (33-35) (≥20 mins vs <20 mins, adjOR= 1.23, 1.07,1.41). No neonatal mortality events were reported in the study in Canada (42). The study from Japan (45) reported that following a median reduction in travel time from 67 minutes in 2002 to 39 minutes in 2006 that there was a decrease in NM rate from 1.67 to 1.28, however no further analyses were presented.

Perinatal mortality (PM)

Seven studies examined PM, one from France (30), one from the Netherlands (36) and five from Canada (40-44). The French study (30) found no significant association between increasing travel time to the nearest OU and PM based on unadjusted data. However, in the Dutch study a longer travel time 20 mins or more was significantly associated with higher PM (36) (>=20 mins vs < 20 mins, adjOR 1.66, 95%CI 1.59-1.74).

The Canadian studies also reported longer travel times to OUs being associated with an elevated risk of PM. A significant increase in PM was reported in women living more than four hours away from OUs compared to women living less than one hour (> 4hrs vs < 1 hr adjOR= 3.17, 95%CI: 1.45, 6.95) (41). However, findings from the same study suggested no significant increase between women living 1-2 hours and 2 to 4 hours from an OU compared to those living less than one hour from services. Similarly, the PM risk significantly increased in women who lived > 1 hour from OUs in a further Canadian study (42), (cOR=1.54, 95%CI: 1.09, 2.17). When this was divided into different Canadian provinces (43), the rates of PM were highest in communities living more than 4 hrs from an

For peer review only - http://bmjopen.bmj.com/site/about/guidelines.xhtml

OU in comparison to less than one hour in British Colombia only (adjOR=2.84, 95%CI 2.84,5.10). Stoll et al (44) found, that in women who received midwifery care only, PM was not statistically significantly different for women living more than two hours away from an OU compared to women living less than one hour from an OU based on an unadjusted analysis (cOR= 3.57, 95%CI 0.81,15.76). In Darling et al (40), the PM rates were not statistically significantly different for women with a planned home birth and more than 30 minute drive from hospital (adjRR=2.2, 95%CI 0.67,7.43).

Infant Mortality (IM)

One Canadian study (41) reported no significant difference in IM rates for women living less than one hour travel time to OU compared with more than one hour travel time to OU (cOR= 1.51, 95%CI: 0.86, 2.63).

Born before arrival (BBA)

Six studies reported this outcome., four cohort studies (30, 39, 41, 42), and two case-control studies (31, 32). Five of the six studies found some association between travel time and BBA, four based on adjusted analyses.

There were three studies conducted in France (30-32). Combier et al (30), reported that a travel time greater than 15 minutes was significantly associated with an increased risk of BBA (16-30 mins vs < 16 mins, adjOR= 1.73, 95%CI: 1.23, 2.46); (31-45 mins vs < 16 mins, adjOR= 1.64, 95%CI: 1.06, 2.54) (30). In a case control study (32) the BBA rate increased six fold when the travel time increased to more than 45 minutes from home to the OU compared to women who travelled less than 15 minutes (> 45 mins vs > 15 mins, adjOR= 6.18 95%CI 1.33, 28.65). However, in the other case control study the risk of BBA was not significantly increased in women who travelled for greater than 20 minutes (31).

For peer review only

In a study from Norway Engjom et al (38, 39) the risk of BBA increased significantly with longer travel time to the nearest OU from home. Women who travelled more than two hours had an eight fold increased risk of BBA compared to women who lived within one hour of the nearest OU (>2hrs vs <1 hr, cOR= 8.21, 95%CI: 7.19, 9.37) (39).

The studies from Canada (41, 42) found a significant increase in BBA in women living in communities greater than one hour travel time from an OU compared to those living less than one hour away. In Grzybowski et al (41), women who lived one to two hours from an OU had the highest risk of BBA compared to less than one hour (adjOR= 6.41, 95%CI: 3.69, 11.28) and women who lived greater than four hours away also had increased risk compared to those living less than one hour away (adjOR: 3.63, 95% 1.40,9.40), however there was no difference between those who lived 2-4 hours from an OU and those living less than one hour away (adj OR=0.92, 95%CI: 0.22, 3.88). Gryzbowski et al (42), found a five fold increase in BBA in women who lived more than an hour away from an OU in comparison to women who lived less than an hour away (cOR=5.06, 95%CI 3.82,6.70).

Neonatal unit admission (NNU)

Three studies from Canada reported on NNU admission (40-42). The two studies from British Columbia (41, 42) reported neonatal unit admissions depending on whether the admission was for Level 2 care (High Dependency) or Level 3 care (Intensive Care). Findings from one of these studies (41), showed NNU level 2 admission increased significantly in babies born to women living more than one hour away from an OU compared to less than one hour (adjOR= 2.20, 95%CI: 1.59, 3.05). For those living 2-4 hours away, Level 2 admissions were significantly lower compared with those living less than 1 hour away (adjOR=0.31, 95%CI 0.14,0.65). For those living more than 4 hours away there appeared to be no increase in NNU level 2 admission. For level 3 NNU admission, a significantly increased risk was found for the 1-2 hour category (1-2 hrs vs < 1 hr, cOR= 2.72, 95%CI: 1.46, 5.09). For the other two categories, 2-4 and >4 hours, neither crude nor adjusted analyses showed any significant difference. The number of women in each group decreased with increasing

For peer review only - http://bmjopen.bmj.com/site/about/guidelines.xhtml

time from an OU. In Grzybowski et al (42), there was no increased risk of admission to NNU level 2 in babies born to women living more than one hour from an OU compared to less than one hour, however admission to NNU level 3 was significantly higher (cOR: 1.50, 95%CI: 1.01, 2.23). The third Canadian study from Ontario (40), showed a lower relative risk of NNU admission for planned home births with a travel time greater than 30 minutes when compared to less than 30 minutes (adjRR=0.6, 95% CI:0.44, 0.81).

Apgar score

Two studies reported on Apgar Score; one from Canada and one from the Netherlands (35, 40). In Darling et al (40), no significant difference was found for Apgar score less than seven at five minutes between women who planned home birth and lived more or less than 30 minutes away from an OU, either for nulliparous or multiparous sub-groups (adj RR=1.02, 95%CI 0.95, 1.10).

Ravelli et al (35) used a composite outcome of mortality and/or Apgar < 4 at 5 minutes and/or transfer to NNU, and showed a small but significant increase in this outcome in women whose travel time to an OU exceeded 15 minutes (15-20 mins vs <15 mins adjOR=1.11 95%CI: 1.02, 1.21 and >=20 mins vs <15 mins, adjOR= 1.27, 95%CI: 1.17, 1.38).

Hypoxic-Ischemic Encephalopathy (HIE) – No studies reported this outcome.

Discussion

This review describes studies which have explored the associations between OU closure, distance or time to an OU, and maternal and neonatal outcomes. The included studies were conducted in the UK, France, the Netherlands, Norway, Canada and Japan. Many studies were from parts of the world where service configuration varied and the study populations were sometimes dispersed over a large geographical area. The included studies differed in their design, geographical boundaries, outcomes measures used, and included a wide range of travel time/ distance thresholds used. In addition, although many studies reported that potential confounders were adjusted for in their

For peer review only - http://bmjopen.bmj.com/site/about/guidelines.xhtml

analyses, many of the outcomes of interest for this review were crude measures of effect without adjustment. Therefore, comparing these studies with each other was a challenge.

All of these studies were brought together to explore whether women who had to travel longer and further to their planned OU were at increased risk of adverse outcomes. There was one reasonably consistent finding which was that there appeared to be an increased risk of BBA the longer it took to reach the OU. This may have been associated with an increased risk for the baby with a suggestion of an increased risk of perinatal or neonatal mortality in some studies, however, this effect was not consistent across all the studies. There was also an increase in CS rates following closure of an OU and with shorter travel distance and time, however it is unclear if the difference was related to the exposure or unmeasured differences in CS rates.

Strengths and limitations of the review

This work is the first to synthesise systematically the current evidence relevant to obstetric unit closure and the impact of travel time and travel distance on maternal and neonatal outcomes. We applied a wide and sensitive search strategy to identify the relevant literature. All screening, data extraction and risk of bias assessment were performed independently by at least two reviewers.

The process of selecting studies for inclusion was challenging due to a lack of reporting of some details, for example, it was not always clear which level of maternity services the study referred to, in others, the authors declared a particular interest to study travel time and distance but those data were not given.

Interpretation of findings

It is difficult to conclude from this review whether reconfiguration of maternity services, with closure of OUs, resulting in increased travel distances and times for women is unequivocally associated with worse outcomes for the mother or the baby.

For peer review only - http://bmjopen.bmj.com/site/about/guidelines.xhtml

There remains an urgent need to evaluate the impact of changing maternity service provision. The imperative to close and consolidate OUs into larger units is based on a belief that this will improve safety for both mother and baby. If increasing travel times and distances increases risks to mothers and babies, then the postulated benefits of larger OUs could be offset by the harms of the reconfiguration.

Waiting for closure of OUs to prospectively evaluate the impact on the surrounding maternity population will always be challenging. However, exploring the existing impact of distance and travel time from home to an OU may be a reasonable approach to explore what the impact of reconfiguration may be for a proportion of the women in the area served by the OU which would have these parameters increased by closure of one of more local OU(s). Such a study would need to be large to explore the impact of travel time and distance on substantive harms such as mortality for the baby, so will almost certainly need to use routinely collected data. To obtain large numbers these studies will almost certainly include vigorous evaluation of confounders. Measurement of travel time and distance from the woman's place of residence to an OU would also need more sophisticated approaches than has previously been used in many studies.

Such a study design assumes that travel time and distance have a constant effect on outcomes. If local OUs are far away, it is possible that women will modify their behaviour in relation to when they set off for their OU in labour, if they know they have an hour's journey compared with a 20 minute journey. The extent to which this will mitigate the effects of longer travel times would not be seen in a study looking at existing travel times and distances.

Conclusion

Given the substantial variation in these studies we were unable to draw firm conclusions regarding the association between OU closure, travel distance or time to obstetric services and maternal and neonatal outcomes. There appears to be a consistent association with BBA with increasing distance

For peer review only - http://bmjopen.bmj.com/site/about/guidelines.xhtml

and travel time to an OU and a suggestion of increasing risk to the baby. However, few studies have rigorously controlled for potential confounders.

Funding source: This research is funded by the National Institute for Health Research (NIHR) Policy Research Programme, conducted through the Policy Research Unit in Maternal Health and Care, 108/0001. The views expressed are those of the author(s) and not necessarily those of the NIHR or the Department of Health and Social Care.

Competing Interests: None of the authors has any competing interest.

Acknowledgment: Our thanks to Pam White for contacting NHS Trusts and obtaining papers, Nia Roberts for development of the search strategy and Mark Willett for providing unpublished data.

Authors' contributions

PB, FA, RM and JD conceived the research. All authors developed the protocol and RM developed the search strategy. RM, CT, AP, FA and JH screened the search results and full papers. RM, CT, JH, FA and CO assessed the quality of included papers, extracted the data and synthesised the results. RM and FA drafted the manuscript and all authors agreed the final manuscript.

Data sharing statement

All the data included in this systematic review are in the public domain.

For peer review only

For peer review only - http://bmjopen.bmj.com/site/about/guidelines.xhtml

Tables:

Table 1A: Description of included studies – Obstetric unit closures

Author, year, Country	Study design & setting	Study objectives	Study period	Eligibility criteria	Participant characteristics	Description of exposure	Services context information	Review Outcomes	
								Perinatal	Maternal
**UK STUDIES**									
Fleming/East Lancashire study									
Fleming 2013 (12), UK (abstract)	Retrospective analysis pre- and post-service reconfiguration	To examine the effect of major service reconfiguration from 2 OUs to 1 OU + 3 MW-led units on CS rates	Jan-Jun 2010 vs Jan-Jun 2012	NR	N: NR<br><br>Characteristics: NR	Major service reconfiguration in Nov 2010 from 2 OUs to 1 OU + 3 MW-led units	Universal state provision of maternity care. Approx 7000 births /yr at the new unit.	NR	CS
East Lancashire Hospitals NHS Trust 2017, UK (unpublished-data)	Retrospective population-based analysis of routinely collected data	NR	2009-2017	NR	2009-2017 N=53,870 births (2010 data excluded)<br><br>Characteristics: NR	Before and after amalgamation of two obstetric units	Universal state provision of maternity care. Changes driven by pressure on staff rotas, European Working Time Directive, and desire to maintain high	BBA; SB; NM	NR

For peer review only - http://bmjopen.bmj.com/site/about/guidelines.xhtml

							quality service. 6000-7000 births/yr at new unit.		
Mackie 2014 (13), UK (abstract & (unpublished data)	Retrospective analysis pre- and post-service reconfiguration	To assess the effect of the amalgamation of 2 OUs to form a "super-centre" with increased consultant labour ward cover	NR	NR	Pre-amalgamation N=5422<br>Post-amalgamation N=5046<br><br>Characteristics: NR	Service reconfiguration	Universal state provision of maternity care. Approx 5000 births /yr at the new unit.	SB; NM; BBA	Maternal mortality; Emergency CS; 3rd& 4th degree perineal tea; Maternal transfer to ICU
**OTHER EUROPEAN STUDIES**									
Blondel* 2011 (11), France	Retrospective population-based analysis of routinely collected data	To calculate the incidence of BBA births in relation to distance from OU and impact of recent closure on different socio-demographic groups	2005-2006	*Included:* Singleton births<br>*Excluded:* Municipalities if >8% missing data, or high OoH rates. Departments excl. if >20% births already excl.	N=1,349,751 births;<br>OoH n=5740<br>*N Births 1,349, 751*<br>Age (yrs) (n):<br><20 - 26,152<br>20-24 - 188,350<br>25-29 - 427,462<br>30-34 - 442,089<br>35-39 - 213,534<br>40+ - 52,164<br><br>Nullip (n) 774,460	Closure of maternity unit between 2003 and 2006 within 15km radius of home	Centralisation of births in larger units due to safety concerns, financial pressure, efficiency savings, and staff shortage	BBA	NR

For peer review only - http://bmjopen.bmj.com/site/about/guidelines.xhtml

					SES:<br>occupation<br>professional (n) 217,045<br>intellectual 325,746<br>admin 266,000<br>retail 122,727<br>skilled 49,201<br>unskilled 84,664<br>none 184,368<br><br>Ethnicity, education: NR				
Hemminki 2011(16),(16, 46)(17, 47)(17, 47)Fnland	Retrospective population-based analysis of routinely collected data	To describe i) trends in centralisation and unplanned out of hospital births ii) perinatal mortality by place of birth; iii) health and birth outcomes in areas served by hospitals of different levels	Finland 1991-2008;<br><br>Uusimaa district 2004-2008	*Inclusion*: All births<br><br>*Exclusion*: NR	1991-2008 N=474,419<br><br>Characteristics: NR	Centralisation of births, number of maternity units declining from 49 to 34 between 1991 and 2008	Universal access to maternity care, minimal private care. Pre- and postnatal care decentralised, birth hospital-based service, care of high-risk pregnancies centralised. Mean no. births/hospital increased from 1339-1733 over study period.	BBA	NR

For peer review only - http://bmjopen.bmj.com/site/about/guidelines.xhtml

Engjom 2014 (14), Norway	Retrospective population-based study, 3 cohort and 2 cross-sectional studies	To assess the availability of OUs, the risk of unplanned delivery outside OU and maternal morbidity	Cohort: 1979-2009 Cross-sectional: 2000 and 2010	*Included:* Age 15-49 yrs, known place of birth, GA ≥22 wks and/or bthwt ≥500g *Excluded:* Missing maternal address, planned home birth	1979-83 n=252,621 2004-09 n=409,432<br><br>Characteristics: NR	Number of OUs in Norway declined from 95 to 51 between 1979 and 2009	Universal access to maternity care; relatively dispersed population	Perinatal mortality (PM)	CS
Grytten 2014 (15), Norway	Retrospective population-based analysis of routinely collected data	To study whether neonatal and infant mortality were independent of the type of hospital in which the delivery was carried out	1979-2005	*Inclusion*: All births<br><br>*Exclusion*: NR	N=33,677<br><br>Characteristics: NR	Number of local hospitals fell from 43 to 26 between 1979 and 2005. 17 maternity wards in local hospitals closed	Universal free access to maternity care. Low-risk births in local hospitals, high risk in central/ regional hospitals. Births in Central/Regional hospitals increased from 65-81% over study period.	NM; Infant mortality (IM)	NR
**CANADIAN STUDIES**									
Le Coutour 1990 (19), Canada	Retrospective population-	To describe the evolution of	1981-1985	*Inclusion*: All births	1981-1984 N=128,688	7/13 university hospitals, 5/13	State provision of maternity	NR	Overall CS

For peer review only - http://bmjopen.bmj.com/site/about/guidelines.xhtml

	based analysis of routinely collected data	obstetric practice in Montreal metropolitan area before/ after closure of units		*Exclusion*: NR	Characteristics: NR	specialist hospitals, 4/13 smaller units closed	care. Closures due to budgetary restrictions.		
Allen 2004 (20), Canada	Retrospective population-based analysis of routinely collected data	To evaluate the effect of hospital closures on critical obstetrical interventions and perinatal outcomes in rural communities	Pre-closure : 1988-1993<br><br>Post-closure : 1996-2002	*Inclusion*: All births<br><br>*Exclusion*: Delivery <20 weeks; bthwt <500g; triplets+; major congenital anomaly	1988-93 n=69,213<br>1996-2002 n=63,510<br>*Range %*<br>Age >34 yrs: 5.6-14.8;<br>Nullip 39.4-46.8<br>Twins 1.0-1.3<br><br>Ethnicity, socioeconomic status, education: NR	1988-1993 =27 hospitals<br>1996-2002 =19 hospitals<br><br>Reduction in maternity units from 42 to 11 between 1970 and 2002	State provision of maternity care.<br>Reduction in number of units and physicians due to financial constraints and difficulty maintaining clinical competence and confidence.	SB; Fetal/ neonatal mortality (NM)	NR
Hutcheon 2017 (18), Canada	Retrospective population based analysis of routinely collected data	To examine the effect of these service closures on the intrapartum outcomes of pregnant women living in affected communities	1998-2014	*Inclusion*: Women living in 25 communiti es affected by OU closure,<br><br>*Exclusion*: Proximity to metropolit an areas;	*Pre-closure*<br>N=5796<br>Median maternal age 27 yrs (IQR 23-31);<br>Nullip 39.3%<br><br>*Post-closure*<br>N=6153<br>Median maternal age	Between 1998 and 2014 nearly one-third of hospitals stopped providing maternity services	State provision of maternity care.<br>Centralisation of obstetric services, majority of hospital closures in low-volume hospitals	BBA;<br>Perinatal/ Neonatal mortality; neonatal unit admission (NNU)	Overall CS; Maternal mortality; 3rd/4th degree perineal tear, blood transfusion , maternal admission to ICU

For peer review only - http://bmjopen.bmj.com/site/about/guidelines.xhtml

				uncertainty about dates of service closure	28 yrs (IQR 24-32); Nullip 40.7% Ethnicity, Socioeconomic status, education: NR				

Abbreviations: Approx: approximately; bthwt: Birth Wight; BBA: Born before arrival; CS: Caesarean Section; excl: Excluded; ITU: Intensive Care Unit; IQR: Interquartile Range; MW: Midwife; NHS: National Health Services; NNU: Neonatal Unit; NM: Neonatal Mortality, NR: Not Reported; Nullip: Nulliparous; OU: Obstetric unit; SB: Stillbirth; wo: Without; yr: Year. *Blondel 2011 is also included in travel distance.

Table 1B: Risk of bias - Obstetric unit closure studies

Author, year, country	Study sample selection bias	Bias in measurement of exposure	Bias in measurement of outcomes	Attrition bias	Analysis method reported and appropriate	Closure independent of other changes over time	Potential confounders adjusted for and listed
**UK STUDIES**							
Fleming/East Lancashire study							
Fleming 2013 (12), UK (abstract)	**LOW** All births in East Lancashire Maternity Services catchment	**LOW** All births in catchment area affected by the closure Number of OUs closed reported	**LOW** Objective outcome (CS)	**UNCLEAR** Not reported	**UNCLEAR** None reported	**UNCLEAR** None reported	**UNCLEAR** None reported

For peer review only - http://bmjopen.bmj.com/site/about/guidelines.xhtml

East Lancashire Hospitals NHS Trust 2017, UK (unpublished)	**UNCLEAR** Unpublished data, no details	**UNCLEAR** Unpublished data, no details	**LOW** Objective outcomes (BBA,SB, NM)	**UNCLEAR** Unpublished data, no details	**UNCLEAR** Unpublished data, no details	**UNCLEAR** Unpublished data, no details	**UNCLEAR** Unpublished data, no details
Mackie 2014 (13), UK (abstract & unpublished)	**LOW** Data from Maternity Information System	**LOW** All births in catchment area affected by the closure. Number of OUs closed reported	**LOW** Objective outcomes (SB, NM, BBA, MM, ICU admission, perineal tears)	**UNCLEAR** Not reported	**UNCLEAR** None reported	**UNCLEAR** None reported	**UNCLEAR** None reported
**OTHER EUROPEAN STUDIES**							
Blondel 2011 (11), France*	**LOW** Data from birth certificates	**LOW** Number of OUs closed reported	**LOW** Objective outcome (BBA)	**LOW** 11% excluded due to missing data	**LOW** Analysis method was described and appropriate, a multi-level model analysis	**UNCLEAR** None reported	**LOW** Adjusted for maternal age, occupational category and rurality
Hemminki 2011(16), Finland	**LOW** Data from Finnish medical birth register	**LOW** All births in catchment area affected by the closure Number of OUs closed reported	**LOW** Objective outcome (BBA)	**LOW** Births with missing information excluded (<0.05%)	**LOW** Analysis method was described and appropriate, a regression model with adjusted analysis	**UNCLEAR** None reported	**LOW** Adjusted for Parity, plurality, age, socioeconomic status and smoking

For peer review only - http://bmjopen.bmj.com/site/about/guidelines.xhtml

Engjom 2014 (14), Norway	**LOW** Data from Medical Birth Registry of Norway	**LOW** All births in Norway affected by the closure Number of OUs closed reported	**LOW** Objective outcome (BBA)	**LOW** All units report to Medical Birth Registry	**LOW** Analysis method appropriate, a logistic regression model, crude and adjusted results given	**UNCLEAR** None reported	**LOW** Adjusted for maternal age, parity, education and partner status
Grytten 2014 (15)(16)(16)(15), Norway	**LOW** Data from Medical Birth Registry of Norway	**LOW** All births in Norway Number of OUs closed reported	**LOW** Objective outcome (NM, Infant mortality)	**LOW** All maternity units report to Medical Birth Registry	**HIGH** Not standard method for data analyses; difference-in-difference statistical method reporting coefficients	**LOW** Temporal variation adjusted for in analysis	**LOW** Maternal age, immigrant status, level of education, marital status, predisposing medical factors and characteristics of the birth
**CANADIAN STUDIES**							
Le Coutour 1990 (19), Canada	**LOW** Data from MED-ECHO - regional data collection system	**LOW** All births in catchment area Number of OUs closed reported	**LOW** Objective outcome (CS)	**UNCLEAR** No information	**UNCLEAR** No details on data analysed method	**UNCLEAR** None reported	**HIGH** No adjustment
Allen 2004 (20), Canada	**LOW** Data from Nova Scotia Atlee Perinatal Database	**LOW** All births in catchment area affected by the closure Number of OUs closed reported	**LOW** Objective outcomes (SB, NM)	**UNCLEAR** Population based dataset but no information about missing data	**LOW** Analysis method appropriate and data from logistic regression models were reported	**UNCLEAR** None reported	**LOW** Maternal age, smoking and maternal diseases

For peer review only - http://bmjopen.bmj.com/site/about/guidelines.xhtml

Hutcheon 2017 (18), Canada	**LOW** Data from British Columbia Perinatal registry	**LOW** All births in catchment area affect by the closure Number of OUs closed reported	**LOW** Except for 3rd/4th degree tears. Objective outcomes (BBA, PM, NM, ICU admission, CS, MM, blood transfusion, Maternal admission to ICU)	**LOW** >99% complete	**HIGH** Used a within-community fixed-effects design and Poisson regression	**UNCLEAR** None reported	**HIGH** No adjustment

Abbreviations: BBA: Born Before Arrival; CS: Caesarean Section; ICU: Intensive Care Unit; MM: Maternal Mortality; NHS: National Health Services; NM: Neonatal Mortality; PM: Perinatal Mortality; SB: Stillbirth. *Blondel 2011 included in travel distance & OU closure.

For peer review only

Table 1C: Outcomes - Obstetric unit closure

Outcomes	Author, Year, Country	Exposure and comparator groups	Participants (N, n, %)			Findings		
**MATERNAL OUTCOMES**								
**Maternal mortality**	Mackie 2014 (13), UK	Before and after	**Year**	**Deliveries (N= 15,349)**	**MM n (%)**	**Year**	**Crude OR (95%CI)**	**Adjusted OR (95%CI)**

For peer review only - http://bmjopen.bmj.com/site/about/guidelines.xhtml

**(MM)**	(unpublished data)	amalgamation of 2 OUs	Pre 2010-2011	5354	1 (0.02)	Pre 2010-2011	1	NR
			Post 2011-2013	9995	1(0.01)	Post 2011-2013	*0.54 (0.03, 8.56)*	NR
	Hutcheon 2017 (18), British Columbia, Canada	Before and after OU closure (1998-2014)	**Closure status**	**Deliveries (N=11,949)**	**Maternal deaths n (%)**	**Closure status**	***Crude OR (95%CI)***	***Adjusted OR (95%CI)***
			Preclosure	5796	<5 (<0.09)	Preclosure	NR	NR
			Postclosure	6153	<5 (<0.08)	Postclosure	NR	NR
			No significant difference pre/post closure in adverse events during labour and delivery					
**Caesarean section (CS) (overall or intrapartum)**	Fleming 2013 (12), UK (abstract only)	Before and after closure of OU in 2010	***Closure status***	***Deliveries (N=NR)***	***CS n (%)***	**Closure status**	**Crude OR (95%CI)**	**Adjusted OR (95%CI)**
			Preclosure, early 2010	NR	(NR) 26.1	Preclosure, early 2010	NR	NR
			Postclosure, 2012	NR	(NR) 21.5	Postclosure, 2012	NR	NR
			Proportions of CS presented with no other data.					
	Engjom 2014 (14), Norway	2000 compared to 2009 during which time number of OUs declined from 47 to 41	**Year**	**Deliveries (N=2,177,934)**	**CS n (%)**	**Year**	**Crude OR (95% CI)**	**Adjusted OR (95%CI)**
			Pre 2000	58,632	7653 (13.10)	Pre 2000	1	NR
			Post 2009	61,895	10,154 (16.41)	Post 2009	*1.31 (1.27, 1.35)*	NR
	Le Coutour 1990 (19), Quebec, Canada	Before and after closure of OUs between 1982	**Year**	**Deliveries (N= 64,274)**	**CS n (%)**	**Year**	**Crude OR (95% CI)**	**Adjusted OR (95%CI)**
			Pre 1981	32,807	5852 (17.84)	Pre 1981	1	NR

For peer review only - http://bmjopen.bmj.com/site/about/guidelines.xhtml

		and 1983	Post 1984	31,467	6214 (19.7)	Post 1984	*1.13 (1.09, 1.18)*	NR
	Hutcheon 2017 (18), British Columbia, Canada	Before and after OUs, closure (1998-2014)	**Closure status**	**Deliveries (N= 11,949)**	**CS n (%)**	**Closure status**	**Crude OR (95% CI)**	**Adjusted OR (95%CI)**
			Preclosure	5796	1387 (23.93)	Preclosure	1	NR
			Postclosure	6153	1579 (25.70)	Postclosure	*1.10 (1.01, 1.19)*	NR
**Emergency caesarean section (CS)**	Mackie 2014 (13), UK (abstract only)	Before and after amalgamation of two OUs	**Year**	**Deliveries (N=15,349)**	**Emergency CS n(%)**	**Year**	**Crude OR (95%CI)**	**Adjusted OR (95%CI)**
			Pre 2010-2011	5354	739 (13.80)	Pre 2010-2011	1	NR
			Post 2011-2013	9995	1322 (13.23)	Post 2011-2013	*0.95 (0.86, 1.05)*	NR
**Severe perineal trauma (3rd or 4th degree tear)**	Mackie 2014 (13), UK (unpublished data)	Before and after amalgamation of two OUs	**Year**	**Deliveries (N=15,349)**	**3rd & 4th n (%)**	**Year**	**Crude OR (95%CI)**	**Adjusted OR (95%CI)**
			Pre 2010-2011	5354	133 (2.48)	Pre 2010-2011	1	NR
			Post 2011-2013	9995	276 (2.76)	Post 2011-2013	*1.11 (0.90, 1.37)*	NR
	Hutcheon 2017 (18), British Columbia, Canada	Before and after OU closure (1998-2014)	**Closure status**	**Deliveries (N=11,949)**	**3rd or 4th degree tear n (%)**	**Closure status**	**Crude OR (95% CI)**	**Adjusted OR (95%CI)**
			Preclosure	5796	136 (2.40)	Preclosure	1	NR
			Postclosure	6153	174 (2.82)	Postclosure	*1.21 (0.96, 1.52)*	NR
**Postpartum haemorrhage**	No studies							
**Maternal admission to**	Mackie 2014 (13), UK	Before and after	**Year**	**Deliveries (N=15,349)**	**n (%)**	**Year**	**Crude OR (95%CI)**	**Adjusted OR (95%CI)**

For peer review only - http://bmjopen.bmj.com/site/about/guidelines.xhtml

**ICU**	(abstract only)	amalgamation of two OUs	Pre 2010-2011	5354	18 (0.34)	Pre 2010-2011	1	NR
			Post 2011-2013	9995	27(0.27)	Post 2011-2013	*0.80 (0.44, 1.46)*	NR
	Hutcheon 2017 (18), British Columbia, Canada	Before and after OU closure (1998-2014)	**Closure status**	**Deliveries (N= 11,949)**	**ICU admission n**	**Closure status**	**Crude OR (95%CI)**	**Adjusted OR (95%CI)**
			Preclosure	5796	<5	Preclosure	NR	NR
			Postclosure	6153	<5	Postclosure	NR	NR
**Maternal Blood transfusion**	Hutcheon 2017 (18), British Columbia, Canada	Before and after OU closure (1998-2014)	**Closure status**	**Deliveries (N= 11,949)**	**Blood transfusion n (%)**	**Closure status**	**Crude OR (95% CI)**	**Adjusted OR (95%CI)**
			Preclosure	5796	53 (0.91)	Preclosure	1	NR
			Postclosure	6153	46 (0.75)	Postclosure	*0.82 (0.55, 1.21)*	NR
**NEONATAL OUTCOMES**								
**Stillbirth (SB)**	East Lancashire Hospitals NHS Trust, 2017, UK (unpublished data)	Before and after amalgamation of two obstetric units (OUs) in 2010	**Year**	**Deliveries (N= 53,870)**	**SB n (%)**	**Year**	**Crude OR (95%CI)**	**Adjusted OR (95%CI)**
			Pre 2009	6492	75 (1.16)	Pre 2009	1	NR
			Post 2011-2017	47,378	333 (*0.70*)	Post 2011-2017	*0.61 (0.47, 0.78)*	NR
			Stillbirth>24 weeks					
	Allen 2004 (20), Nova Scotia, Canada	Before and after closure of OUs	**Year**	**Deliveries (N=132,723)**	**SB n (%)**	**Year**	**Crude OR (95%CI)**	**Adjusted OR (95%CI)**
			Pre 1988-93	69,213	291 (*0.42*)	Pre 1988-1993	1	NR

			Post 1996-2002	63,510	214 *(0.34)*	Post 1996-2002	*0.80 (0.67, 0.96)*	NR
	Mackie 2014(13), UK (unpublished data)	Before and after amalgamation of 2 OUs	**Year**	**Deliveries (N=15,552)**	**SB n (%)**	**Year**	**Crude OR (95%CI)**	**Adjusted OR (95%CI)**
			Pre 2010-2011	5422	29 (*0.53*)	Pre 2010 -2011	1	NR
			Post 2011-2013	10,130	60 (*0.59*)	Post 2011-2013	*1.11 (0.71, 1.73)*	NR
**Neonatal mortality (NM)**	East Lancashire Hospitals NHS Trust, 2017, UK (unpublished data)	Before and after amalgamation of two obstetric units in 2010	**Year**	**Deliveries (N=53,870)**	**NM n (%)**	**Year**	**Crude OR (95%CI)**	**Adjusted OR(95%CI)**
			Pre 2009	6492	4 (*0.06*)	Pre 2009	1	NR
			Post 2011-2017	47,378	39 (*0.08*)	Post 2011-2017	*1.33 (0.81, 2.17)*	NR
			NM not defined					
	Mackie 2014(13), UK (unpublished data)	Before and after amalgamation of 2 OUs	**Year**	**Deliveries (N=15,552)**	**NM n (%)**	**Year**	**Crude OR (95%CI)**	**Adjusted OR(95%CI)**
			Pre 2010-2011	5422	6 (*0.11*)	Pre 2010-2011	1	NR
			Post 2011-2013	10,130	9 (*0.90*)	Post 2011-2013	*0.80 (0.29, 2.26)*	NR
	Grytten 2014(15), Norway	Before and after 13 hospital closures	**Year**	**Deliveries (N=33,677)**	**NM n (%)**	**Year**	**Crude OR (95%CI)**	**Adjusted OR(95%CI)**
			5 yrs before	16,297	NR	5 yrs before	NR	NR
			5 yrs after	17,380	NR	5 yrs after	NR	NR
			No statistically significant difference					

For peer review only

For peer review only - http://bmjopen.bmj.com/site/about/guidelines.xhtml

**Perinatal mortality (PM)**	Engjom 2014(14), Norway	2000 compared to 2009 during which time number of OUs declined from 47 to 41	**Year**	**Deliveries (N=2,177,934)**	**PM n (/1000 births)**	**Year**	**Crude OR (95% CI)**	**Adjusted OR(95%CI)**
			Pre 2000	58,632	124 (2.11)	Pre 2000	1	NR
			Post 2009	61,895	99 (1.60)	Post 2009	*0.76 (0.58, 0.98)*	NR
			PM (Intrapartum & neonatal death < 24 hrs, both live & still born)					
	Allen 2004(20), Nova Scotia, Canada	Before and after closure of OUs	**Years**	**Deliveries (N=132,723)**	**PM n (%)**	**Years**	**Crude OR (95%CI)**	**Adjusted OR (95%CI)**
			Pre 1988-1993	69,213	422 (0.61)	Pre 1988-93	1	NR
			Post 1996-2002	63,510	278 (0.43)	Post 1966-2002	*0.75 (0.64, 0.87)*	*NR*
			Fetal/neonatal mortality not defined					
**Infant mortality**	Grytten 2014(15), Norway	Before and after 13 hospital closures	**Year**	**Deliveries (N=33,677)**	**IM n (%)**	**Years**	**Crude OR (95%CI)**	**Adjusted OR (95%CI)**
			5 yrs before	16,297	NR	5 yrs before	NR	NR
			5 yrs after	17,380	NR	5 yrs after	NR	NR
			No significant difference in infant mortality					
**Born before arrival (BBA)**	East Lancashire Hospitals NHS Trust, 2017, UK (unpublished data)	Before and after amalgamation of two obstetric units	**Year**	**Deliveries (N= 53,870)**	**BBA n (%)**	**Year**	**Crude OR (95%CI)**	**Adjusted OR (95%%CI)**
			Pre 2009	6492	25 (0.39)	Pre 2009	1	NR
			Post 2011-2017	47,378	341 (0.72)	Post 2011-2017	*1.88 (1.25, 2.82)*	NR

For peer review only - http://bmjopen.bmj.com/site/about/guidelines.xhtml

	Mackie 2014(13), UK (unpublished data)	Before and after amalgamation of 2 OUs	**Year**	**Deliveries (N=15,349)**		**BBA n (%)**	**Year**	**Crude OR (95%CI)**	**Adjusted OR (95%CI)**
			Pre 2010-2011	5354		11 (0.21)	Pre 2010-2011	1	NR
			Post 2011-2013	9995		26 (0.26)	Post 2011-2013	*1.28 (0.63, 2.60)*	NR
	Blondel 2011(11), France	OU closure within 15 km radius 2003-2006	**Yrs 2003-2006**	**Deliveries (N=1,349,751)**		**BBA n (/1000 births)**	**Yrs 2003-2009**	**Crude OR (95%CI)**	**Adjusted OR (95%CI)**
			No closure	1,001,858		4531 (4.52)	No closure	1	1
			Closure within 15km radius	347,893		1209 (3.47)	Closure within 15km radius	0.77 (0.72, 0.82)	0.91 (0.84, 1.00)
	Engjom 2014(14), Norway	1979-83 compared to 2004-09, number of emergency OUs declined from 47 to 41	**Year**	**Deliveries (N=662,053)**		**BBA n (%)**	**Year**	**Crude OR**	**Adjusted OR (95%CI)**
			Pre 1979-83	252,621		984 (0.39)	Pre 1979-83	1	1
			Post 2004-09	409,432		2832 (0.69)	Post 2004-09	1.8 (1.6, 1.9)	2.0 (1.9, 2.2)
	Hemminki 2011(16), Finland	Centralisation of hospitals over years 1991-2008	**Year**	**Births (N)**	**Unplanned BBA n(/1000)**	**Planned or unplanned BBA n(/1000)**	**Year**	**Crude OR (95%CI)**	**Adjusted OR (95%CI)**
			Pre 1991	65,632	-	68 (1.0)	Pre 1991	1	NR
			Post 2004-2008	56,873	222 (3.76)	243 (4.1)	Post 2004-2008	*4.14 (3.16, 5.41)*	NR
			Total N (1991-2008)= 122,505						
	Hutcheon 2017(18), British Columbia,	Before and after OU closure (1998-2014)	**Closure status**	**Deliveries (N=11,949)**		**Unplanned BBA n (%)**	**Closure status**	**Crude OR (95%CI)**	**Adjusted OR (95%CI)**
			Preclosure	5796		30 (0.5)	Preclosure	1	NR

42

For peer review only

	Canada		Postclosure	6153	109 (1.8)	Postclosure	*3.47 (2.31, 5.20)*	NR
**Neonatal unit admission (NNU) >2 days or transfer within 24 hrs of birth to ICU facility for newborn >/=2500g**	Hutcheon 2017(18), British Columbia, Canada	Before and after OU closure (1998-2014)	**Closure status**	**Deliveries (N=11,949)**	**NNU admission n (%)**	**Closure status**	**Crude OR (95% CI)**	**Adjusted OR (95%CI)**
			Preclosure	5796	68 (1.17)	Preclosure	1	NR
			Postclosure	6153	28 (0.46)	Postclosure	*0.39 (0.25, 0.60)*	NR
**Apgar score (5 minute Apgar score <7)**	Hutcheon 2017(18), British Columbia, Canada	Before and after OU closure (1998-2014) compared to communities unaffected by closure	**Closure status**	**Deliveries (N=11,949)**	**5 min Apgar score <7 n (%)**	**Closure status**	Crude OR (95% CI)	Adjusted OR (95%CI)
			Preclosure	5796	71 (1.22)	Preclosure	1	NR
			Postclosure	6153	85 (1.28)	Postclosure	*1.13 (0.82, 1.55)*	NR
**Hypoxic Ischaemic Encephalopathy (HIE)**	No studies							

Abbreviations: aOR: Adjusted Odds Ratio; BBA: Born Before Arrival; ICU: Intensive Care Unit; NHS: National Health Services; PM: Perinatal Mortality; OR: Odds Ratio; OU: Obstetric Unit.

For peer review only - http://bmjopen.bmj.com/site/about/guidelines.xhtml

Table 2A: Description of included studies – Travel distance

Author, year, country	Study design & setting	Study objectives	Study period	Eligibility criteria	Participant characteristics	Description of exposures Travel Distance	Services context information	Review Outcomes	
								Perinatal	Maternal
**UK STUDIES**									
Bhoopalam 1991 (21), UK	Case-control study, 2 OUs	To establish BBA prevalence and women at risk of BBA, and morbidity and mortality associated with BBA births	1983 – 1987	*Included cases:* Women and their BBA babies *Included controls:* Two controls for each BBA case, one random (next born in the same hospital), one matched (next born in same hospital matched by GA and BW) *Excluded:* BW < 500g	N(BBAs) =137, 1 twins All N= 398 Age yrs (n): <21 (69) 21-35 (339) >35 (27) Nullips (107) Ethnicity (n): European(191) Asian (101) Other (16) SES & education: NR	Distance (km): <2 2-7 >7	Universal state provision, 2 units 6 miles apart, serving rural areas of Warwickshire	BBA	NR
**OTHER EUROPEAN STUDIES**									
Pasquier 2007(22), France	Retrospective, population-based cohort, 3 Level-III	To examine maternal origin, distance to the nearest	1990-95 & 1996-2000 (2 periods separated	*Included:* Fetuses with omphalocele, gastroschisis,	N =706 infants N= 554 (analysed) Age: yrs (n, %) <21 (15, 2)	Distance (km): <11 11-50	Distance to Level III maternity ward with NNU and	Neonatal Mortality (NM)	NR

For peer review only - http://bmjopen.bmj.com/site/about/guidelines.xhtml

	maternity wards with neonatal surgical centre, Rhône-Alpes Region	maternity ward with a neonatal surgical centre, on perinatal diagnosis, elective termination of pregnancy, delivery in an adequate place and neonatal mortality for pregnancies with severe malformations	due to changes in prenatal screening)	diaphragmatic hernia or spina bifida that required surgical repair<br>*Excluded:* Chromosomal anomalies fetuses and babies without anomalies	21-35 (550, 82)<br>>35 (106, 16)<br>Ethnicity: (n,%)<br>Western European (393, 76)<br>Non-Western European (124, 24)<br>Parity, SES & education: NR	>50	a neonatal surgical centre, there were 3 in the Rhone-Alps Region		
Blondel 2011 (11), France*	Retrospective cohort, population based study, metropolitan France	To calculate the incidence of BBA birth in relation to distance from maternity units and the impact of recent closure on different sociodemographic groups	2005-2006	*Included:* Singleton live births<br>*Excluded:* Municipalities with >8% missing data, unrealistically high BBA births, Departments were excl. if >20% births already excl.	N=1,517,599 livebirths<br>N=1,349,751(analysed)<br>Age yrs - n<br><20 - 26,152<br>20-34 –<br>1,057,90135-39 -<br>213,534<br>40+ - 52,164<br>Parity: Nullip n -<br>774,460<br>SES n: Occupation professional-<br>217,045<br>Intellectual-<br>325,746<br>Admin- 266,000	Distance (km):<br><5<br>5-14<br>15-29<br>30-44<br>45+	Centralising births in larger units	BBA;<br>BBA by parity	NR

For peer review only - http://bmjopen.bmj.com/site/about/guidelines.xhtml

					Retail-122,727<br>Skilled- 149,201<br>Unskilled- 84,664<br>None- 84,368<br>Ethnicity & education: NR				
Pilkington 2014 (23), France	Retrospective, population-based cohort study, French National Vital Statistics registry from mainland France	To investigate the impact of distance to closest maternity unit on perinatal outcomes	2001-2008, Stillbirth data 2002-2005	*Included:*<br>All births<br>*Excluded:*<br>*NR*	N= 3,086,128 all births<br>N=3,085,839(analysed)<br>Age yrs (n)<br><25 - 494,689<br>25-34 – 2,008,320<br>35-39 - 469,975<br>40+ - 113,144<br>Singleton pregnancy N= 2,988,169<br>Multiple pregnancy: N= 97,959<br>Parity, ethnicity and socioeconomic status & education: NR	Distance (km):<br><5<br>5-14<br>15-29<br>30-44<br>≥45	1998 to 2003, 20% of maternity units closed<br>Mean distance to nearest maternity unit increased (6.6–7.2 km)	Stillbirths (SB); NM	NR
Ovaskainen 2015 (25), Finland	Case–control study, one centre, Tampere University Hospital	To establish if BBA births increased over time, to identify risk factors associated with BBAs, also if	1996 - 2011	*Included Cases:*<br>Planned and unplanned BBA<br>*Included Controls:*<br>2 controls for infant and	Cases:<br>BBAs (n = 67 births): Age yrs (mean, SD) (range)- 29.0, 5.9 (15-47)<br>Parity 1 (0-16)<br>Controls:	Distance (km):<br><35<br>≥35	Tampere University Hospital is the catchment area for 23 municipalities, 521700	BBA	NR

For peer review only - http://bmjopen.bmj.com/site/about/guidelines.xhtml

		BBAs babies were more prone to neonatal morbidities compared to those delivered in hospital		mother for each BBA case<br>*Excluded:*<br>BBA with no information whether planned or unplanned	N= 134<br>Plurality, ethnicity, education & SES: NR.		residents 5000 births/yr		
Fougner 2000 (24), Norway	Case-control study, 14 municipalities, Oppland County	To compare the experience and care of women who delivered during transport to hospital and women who delivered an hour after arriving to hospital	1989 - 1997	*Included Cases:* Women who delivered their babies before arriving at hospital<br>*Included Controls:* Women who delivered their babies with one hour after arriving at hospital<br>*Excluded:* NR	N=202<br>Cases: N= 115 BBA women<br>Parity n (%)- Nullips 15 (13%)<br>Controls: N=87 women<br>Parity- Nullips 18 (20%)<br>Age, ethnicity, education & SES: NR	Distance (km):<br>< 12.88<br>≥12.88	Oppland county: 4 hospitals	BBA	NR
**CANADIAN STUDIES**									
Lisonkova 2011 (26), Canada	Retrospective population-based cohort study, British Columbia	To examine the association between rural residence and birth outcomes in older mothers	1999 - 2003	*Included:*<br>Singleton mothers aged 35+<br>*Excluded:*<br>Women with missing postcodes, babies with	N= 29,698 women<br>Age >35 yrs<br>Parity n (%):<br>Nullip 87,733 (0%)<br>Low SES (n, %)<br>(4385 14% 22.6 vs 3,615, 13.7)<br>Ethnicity n(%)	Distance (km):<br><50<br>50-150<br>>150	17 small maternity units (250-2,500 births/yr) closed between 1999 and 2003	SB; Perinatal Mortality (PM); NNU admission ≥1day	All CS; Emergency CS

For peer review only - http://bmjopen.bmj.com/site/about/guidelines.xhtml

				congenital anomaly	1st nation 826 (2,8%) Education: NR				

Abbreviations: BBA: Born Before Arrival; BW: Birth Weight; CS: Caesarean Section; GA: Gestational Age; NNU: Neonatal Unit; Nullip: Nulliparous; SES: Socio Economic Status. *Blondel 2011 is also included in the OU closure.

Table 2B: Risk of bias – Travel distance

**Author, year, country**	**Study sample selection bias** Additional criteria for case control studies		**Bias in measurement of exposure**	**Bias in measurement of outcomes**	**Attrition bias**	**Analysis method reported and appropriate**	**Potential confounders adjusted for and listed**
**UK STUDIES**							
Bhoopalam 1991 (21), UK	Case definition adequate	**YES** From hospital records	**HIGH** Distance measured in straight line from home address	**LOW** Objective outcome (BBA)	**LOW** 3/134 (2.2%) BBA cases & 10/274 (3.6%) excluded from distance analysis	**HIGH** Descriptive analysis only	**HIGH** No adjustments
	Representative-ness of cases	**YES** BBA cases from 2 hospitals over 5 yrs					
	Appropriate selection of controls	**YES** 2 controls from same hospital					
	Definition of control appropriate	**YES** The outcome (BBA) could not have occurred					

For peer review only - http://bmjopen.bmj.com/site/about/guidelines.xhtml

	Comparability of cases and controls	**NO** Significant differences in maternal age, parity, ethnicity & antenatal booking					
**OTHER EUROPEAN STUDIES**							
Pasquier 2007 (22), France	**LOW** Data from France Central-East malformation registry		**LOW** GIS software used to estimate distance between maternal residence and nearest maternity ward with neonatal surgical centre	**LOW** Objective outcomes (NM)	**LOW** 12 births (0.03%) missing survival data	**LOW** Method detailed and appropriate, univariate analysis & multiple logistic regression were reported	**LOW** Adjusted for parity, ethnicity and other characteristics
Blondel 2011(11), France*	**LOW** Birth certificates		**LOW** GIS software used to estimate distance to hospital from home	**LOW** Objective outcome (BBA)	**LOW** 11% of births excluded	**LOW** Method detailed and appropriate, multilevel model was reported	**LOW** Adjusted for maternal age, occupation, parity and other characteristics

For peer review only

For peer review only - http://bmjopen.bmj.com/site/about/guidelines.xhtml

Pilkington 2014 (23), France	**LOW**<br>French National Vital Statistics Registry		**HIGH**<br>Distance calculated from centre of municipality not home address, using road networks provided by the French National Geography Institute	**LOW**<br>Objective outcomes (SB, NM)	**LOW**<br>10% missing for type of pregnancy and 17% for maternal age	**LOW**<br>Method detailed and appropriate, logistic regression analysis was reported	**LOW**<br>Adjusted for maternal age, plurality, unemployment rate, single parent households
Fougner 2000 (24), Finland	Case definition adequate	**NO**<br>Data from a questionnaire	**HIGH**<br>Data for distance was self-reported in questionnaire	**LOW**<br>Objective outcome (BBA)	**HIGH**<br>Data from women who responded to questionnaire	**HIGH**<br>Descriptive analysis only	**HIGH**<br>No adjustment
	Representative-ness of cases	**YES**<br>Cases from 3 hospitals in one county over 8 yrs					
	Appropriate selection of controls	**Yes**<br>Women who delivered their babies within 1 hour of arriving at hospital					
	Definition of control appropriate	**Yes**<br>Women with no BBA					
	Comparability of cases and controls	**Unclear**					

For peer review only

Ovaskainen 2015 (25), Finland	Case definition adequate	**YES** Medical records	**LOW** Distance from women's home calculated using web-based route planner	**LOW** Objective outcome (BBA)	**High** 13 out-of-hospital deliveries (19%) excluded as could not ascertain whether planned or not planned	**LOW** Method detailed and appropriate, logistic regression data were given	**LOW** Adjusted for single-mother, parity, and other characteristics
	Representative-ness of cases	**YES** Cases from 1 centre, but over 15 yrs					
	Appropriate selection of controls	**YES** Births occurring immediately preceding and following case					
	Definition of control appropriate	**YES** The outcome (BBA) could not have occurred					
	Comparability of cases and controls	**No** Sig. differences for parity, partnership status, smoking, antenatal visits, labour duration &distance to delivery unit					
**CANADIAN STUDIES**							

For peer review only

For peer review only - http://bmjopen.bmj.com/site/about/guidelines.xhtml

Lisonkova 2011 (26), Canada	**LOW**<br>Population based study	**HIGH**<br>GIS used to calculate distance from postcode central point of residence to hospital; mostly using straight line distance	**LOW**<br>Objective outcomes (SB; PM);<br>NNU admission)	**LOW**<br>492 (1.7%) women excluded due to missing postcodes	**LOW**<br>Method detailed and appropriate, multivariate regression analysis reported	**LOW**<br>Adjusted for parity, single mother, low income, ethnicity and other characteristics

Abbreviations: BBA: Born Before Arrival; GIS Geographical Information System; NM: Neonatal Mortality; NNU: Neonatal Unit; PM: Perinatal Mortality; Sig: Significant; SB: Stillbirth. *Blondel 2011 included in travel distance & OU closure.

For peer review only - http://bmjopen.bmj.com/site/about/guidelines.xhtml

Table 2C: Outcomes – Travel distance

Outcomes	Author, year, country	Exposure groups	Participants (N, n, %)			Findings		
**MATERNAL OUTCOMES**								
**Maternal mortality (MM)**	Ovaskainen 2015 (25), Finland	**Travel distance (km):** <35 ≥35	**Groups**	**N (201)**	**NM n (%)**	No events in either group		
			< 35	NR	0			
			≥35	NR	0			
**Caesarean section (CS) (overall or intrapartum)**	Lisonkova 2011 (26), Canada	**Travel distance (km):** <50 50-150 >150	**Groups**	**N (29,698)**	**CS n (%)**	**Groups**	**Crude OR (95%CI)**	**Adjusted OR (95%CI)**
			<50	27,836	9099 (32.70)	<50	1	NR
			50-150	1534	464 (30.25)	50-150	*0.89 (0.80, 1.00)*	NR
			>50	328	94 (28.70)	>50	*0.83 (0.65, 1.05)*	NR
**Emergency CS**	Lisonkova 2011(26), Canada	**Travel distance (km):** <50 50-150 >150	**Groups**	**N (9,657)**	**Emergency CS n (%)**	**Groups**	**Crude OR (95%CI)**	**Adjusted OR (95%CI)**
			<50	9,099	5,378 (59.11)	<50	*1*	NR
			50-150	464	258 (55.60)	50-150	*0.87 (0.72, 1.05)*	NR
			>50	94	52 (55.32)	>50	*0.86 (0.57, 1.29)*	NR

For peer review only - http://bmjopen.bmj.com/site/about/guidelines.xhtml

**Severe perineal trauma**	No studies							
**Postpartum haemorrhage**	No studies							
**Admission to ICU**	No studies							
**Blood transfusion**	No studies							
**NEONATAL OUTCOMES**								
**Stillbirth (SB)**	Pilkington 2014(46), France	**Travel distance (km):**<br><5<br>5-14<br>15-29<br>30-44<br>≥45	**Groups**	**N (3,085,839) (2002-2005)**	**SB n (/per 1000)**	**Groups**	**Crude OR (95%CI)**	**Adjusted OR (95%CI)**
			<5	1,404,665	*13,204* (9.4)	<5	1	1
			5-14	81, 1775	*6,657* (8.2)	5-14	*0.87 (0.85, 0.90)*	Reported as RR 0.87 (NR)*
			15-29	648,495	*5,188* (8.0)	15-29	*0.85 (0.82, 0.88)*	Reported as RR 0.85 (NR)*
			30-44	186,537	*1492* (8.0)	30-44	*0.85 (0.81, 0.90)*	Reported as RR 0.85 (NR)*
			≥45	34,367	*306* (8.9)	≥45	*0.95 (0.84, 1.06)*	Reported as RR 0.95 (NR)(NS)
	Lisonkova 2011 (26), Canada	**Travel distance (km):**<br><50<br>50-150<br>>150	**Groups**	**N (29,698)**	**NM n (%)**	**Groups**	**Crude OR (95%CI)**	**Adjusted OR (95%CI)**
			<50	27,836	150	<50	NR	NR
			50-150	1534	NR	50-150	NR	NR

For review only

			>150	328	NR	>150	NR	NR
			OR not reported. Authors noted stillbirth rate was higher among women living 50- 150 km and >150 km versus <50 km, no significant difference found after adjusting for confounders.					
**Neonatal mortality (NM)**	Pasquier 2007(22), France	**Travel distance (km):** <11 11-50 >50	**Groups**	**N (554)**	**NM n (%)**	**Groups**	**Crude OR (95%CI)**	**Adjusted OR (95%CI)**
			<11	239	NR	<11	NR	1
			11-50	156	NR	11-50	NR	0.98 (0.34, 2.88)
			>50	159	NR	>50	NR	1.37 (0.49, 3.86)
	Pilkington 2014 (23), France	**Travel distance (km):** <5 5-14 15-29 30-44 ≥45	**Groups**	**N (6,202,918) (2001-2008)**	**NM n(/per 1000)**	**Groups**	**Crude OR (95%CI)**	**Adjusted RR (95%CI)**
			<5	2,808,068	*7582* (2.7)	<5	1	1
			5-14	1,626,885	*3416* (2.1)	5-14	*0.78 (0.75, 0.81)*	Reported as RR 0.91 (NR) *
			15-29	1,316,329	*2896* (2.2)	15-29	*0.81 (0.78, 0.85)*	Reported as RR 0.94 (NR)(NS)
			30-44	381,288	*801* (2.1)	30-44	*0.78 (0.72, 0.84)*	Reported as RR 0.9 (NR)*
			≥45	69,787	*154* (2.2)	≥45	*0.82 (0.70, 0.96)*	Reported as RR 0.96 (NR)(NS)
			**NM After BBA**			**NM after BBA**		
			**Groups**	**N (6,202,918) (2001-2008)**	**NM n (/per 100,000)**	**Groups**	**Crude OR (95%CI)**	**Adjusted RR (95%CI)**
			<5	2,808,068	*115* (4.1)	<5	1	1

For peer review only

			5-14	1,626,885	*65* (4.0)	5-14	*0.98 (0.72, 1.32)*	Reported as RR 1.1 (NR)(NS)
			15-29	1,316,329	*72* (5.5)	15-29	*1.34 (0.99, 1.79)*	Reported as RR 1.58 (NR)*
			30-44	381,288	*23* (6.0)	30-44	*1.47 (0.94, 2.30)*	Reported as RR 1.51 (NR)(NS)
			≥45	69,787	*7* (10.0)	≥45	*2.45 (1.14, 5.25)*	Reported as RR 3.68 (NR)*
**Perinatal mortality (PM)**	Lisonkova 2011 (26), Canada	**Travel distance (km):** <50 50-150 >150	**Groups**	**N (29,698)**	**PM n (%)**	**Groups**	***Crude OR (95%CI)***	**Adjusted OR (95%CI)**
			<50	27,836	221 (0.80)	<50	*1*	1
			50-150	1534	19 (1.24)	50-150	*1.57 (0.98, 2.51)*	1.53 (1.10-2.12)
			>150	328	8 (2.44)	>50	*3.12 (1.53, 6.38)*	3.06 (2.20-4.24)
**Infant mortality (IM)**	No studies							
**Born before arrival (BBA)**	Bhoopalam 1991(21), UK	**Travel distance (km):** <2 2-7 >7	**Groups**	**N (398) Cases & controls**	**BBA cases n (%)**	**Groups**	**Crude OR (95%CI)**	**Adjusted OR (95%CI)**
			<2	*59*	4 *(6.80)*	<2	*1*	NR
			2-7	*249*	88 *(35.34)*	2-7	*7.52 (2.64, 21.43)*	NR
			>7	*90*	42 *(46.70)*	>7	*12.03 (4.02, 36.01)*	NR
	Blondel 2011 (11), France	**Travel distance (km):**	**Groups**	**N (1,359,756)**	***BBA n* (Rate /1000 births)**	**Groups**	**Crude OR (95%CI)**	**Adjusted OR (95%CI)**

For peer review only - http://bmjopen.bmj.com/site/about/guidelines.xhtml

		<5<br>5-14<br>15-29<br>30-44<br>>45	<5	596,363	*1849* (3.1)	<5	*1*	NR
			5-14	352,279	*1395* (3.9)	5-14	*1.28 (1.19, 1.37)*	NR
			15-29	296,734	*1659* (5.6)	15-29	*1.81 (1.69, 1.93)*	NR
			30-44	88,670	*692* (7.8)	30-44	*2.53 (2.32, 2.76)*	NR
			>45	15,705	*182* (11.)	45+	*3.77 (3.23, 4.39)*	NR
							**Parity 1&2<br>N =152,426**	**Parity 3+<br>N = 197,325**
						**Groups**	**Adjusted OR (95%CI)**	**Adjusted OR (95%CI)**
						<5	1	1.73 (1.57, 1.90)[a]
						5-14	1.14 (1.03, 1.27)	2.32 (2.04, 2.63)
						15-29	1.39 (1.24, 1.57)	3.25 (2.84, 3.71)
						30-44	1.78 (1.55, 2.05)	3.71 (3.13, 4.41)
						>45	2.47 (2.02, 3.02)	6.46 (4.92, 8.48)
	Ovaskainen 2015 (25), Finland	**Travel distance (km):**<br>< 35<br>≥35	**Groups**	**N (201)**	**BBA n (%)**	**Groups**	**Crude OR (95%CI)**	**Adjusted OR (95%CI)**
			BBA cases	67	NR	<35	NR	1
			Controls	134	NR	≥35	NR	5.02 (1.80-14.04)
			<35 km	NR	NR			

For peer review only

			≥35 km	NR	NR			
	Fougner 2000 (24), Norway	**Travel distance (km):** <12.88 ≥12.88	**Groups**	**N (202) Cases & controls**	**BBA n (%)**	**Groups**	**Crude OR (95%CI)**	**Adjusted OR (95%CI)**
			<12.88	*90*	*44 (48.90)*	<12.88	1	NR
			≥12.88	*112*	*71 (63.34)*	≥12.88	*1.81 (1.03, 3.18)*	NR
**Neonatal unit admission (NNU)**	Lisonkova 2011(26), Canada	**Travel distance (km):** <50 50-150 >150	**Groups**	**N (15,325)**	**NNU n (%)**	**Groups**	**Crude OR (95%CI)**	**Adjusted OR (95%CI)**
			<50	14,333	648 (4.80)	<50	1	NR
			50-150	815	32 (3.92)	50-150	*0.86 (0.60, 1.24)*	NR
			>150	177	12 (6.80)	>150	*1.54 (0.85, 2.77)*	NR
**Apgar score**	No studies							
**HIE**	No studies							

Abbreviations: CS: caesarean section; GA: Gestational age; HIE: Hypoxic-ischemic encephalopathy; ICU: intensive care unit; NNU: neonatal unit; NS: not significant, NR: not reported; RR: relative risk, OR: Odd ratio.

*Significant difference

[a] Reference group: women with 1&2 parity and < 5 km

For peer review only

For peer review only - http://bmjopen.bmj.com/site/about/guidelines.xhtml

Table 3A: Description of included studies – Travel time

Author, year, country	Study design & setting	Study objectives	Study period	Eligibility criteria	Participant characteristics	Description of exposures Travel Distance	Services context information	Review Outcomes	
								Perinatal	Maternal
**UK STUDIES**									
Dummer & Parker 2004 (27), UK	Retrospective population-based cohort study, Cumbria	To investigate whether geographical accessibility to hospitals affected SB rates and infant mortality	1950-1993 grouped: 1950-59 1960-69 1970-79 1980-93	*Included:* All births *Excluded:* Women with missing postcodes	N=283,668 births<br><br>Other characteristics: NR	Travel time (mins): < 17 18-35 >35	Universal state provision of maternity care. 1950-93: 4 hospitals opened, 2 closed	Neonatal mortality (NM); Early NM; Post NM	NR
Paranjothy 2013 (abstract) & 2014 (28, 29), UK	Retrospective cohort study, All Wales Perinatal Survey & National Community Child Health Database	To study the association between travel time from home to OU on intrapartum stillbirth (SB) & NM	1995-2009	*Included:* All registered birth >23 wks GA *Excluded:* Antepartum SB, lethal congenital anomalies, multiple pregnancies, invalid or missing GA, missing	N= 466,255 singleton births<br>*Maternal age yrs %*<br>*<20 9.7*<br>*20-34 76.5*<br>*34-44 13.8*<br>*45+ 0.1*<br>*Parity:*<br>*Nullips 44.9%*<br>*Social deprivation quintile %:*<br>*1 (least depr) 16.7*<br>*2-4 57.8*	Travel time (mins): <15 15-29 30-44 >45	Universal state provision of maternity care. 50 hospitals (16 outside Wales)	Intrapartum SB; Early NM; Late NM	NR

For peer review only - http://bmjopen.bmj.com/site/about/guidelines.xhtml

				maternal age/postcode/ hospital of birth or baby's gender	*5 (most depr) 25.6* Ethnicity, education: NR				
**OTHER EUROPEAN STUDIES**									
Combier 2013 (30), France	Retrospective cohort study, based on hospital discharge summaries, Burgundy	To analyse the effect of travel time to closest OU on pregnancy outcome & prenatal management in Burgundy	2002 - 2009	*Included:* Singleton births >21 wks GA *Excluded:* Medical ToP, multiple pregnancy, births outside Burgundy, births in 2002 & 2008 due to closure of 3 units	N=111,001 births<br><br>Other characteristics =NR	Travel time (mins):<br>≤15<br>16-30<br>31-45<br>≥46	2000-2001: 2 private maternity units closed 2002-2009: 3 public maternity units closed.<br><br>Units(n):<br>2000 (20)<br>2009 (15)	SB; Perinatal mortality (PM); BBA	NR
Renesme 2013 (32), France	Case-control, multicentre study, 8 units, Finistere District, Brittany	To evaluate the social-geographical factors associated with BBAs	2007 - 2009	*Included cases:* BBA of live birth *Included controls*: 2 controls for each case irrespective of delivery mode. *Excluded:* GA <22 weeks,	N=225<br>Cases vs controls<br>N= 76 vs 149<br>Age (median, range) yrs: 30(16–41) vs 30(16-41)<br>Parity (median, range): 2(1–6)<br>Maternal INSEE code n(%): 1, 2, 3 or 4 = 15 (23.8) vs	Travel time (mins):<br>15<br>15–29<br>30–44<br>>45	9700 births/year in Finistere In 2012 units with <300 births/yr were closed. Universal state provision of maternity care	BBA	NR

For peer review only - http://bmjopen.bmj.com/site/about/guidelines.xhtml

				BW <500g, planned home birth	56(43.4); 5 or 6: 20 (31.8) vs 55 (42,6); 8 = 28 (44.4%) vs 18(14)<br>Ethnicity, education*:* NR				
Nguyen 2016 (31), France	Case control study, university hospital in Caen	To estimate the incidence of BBA during the study period	2002-2009	*Included cases:* Unplanned BBA<br>*Included controls:* Next spontaneous birth in hospital<br>*Excluded*: NR	N=188<br>*Cases* n=94<br>Mean age: 28.9 yrs<br>Parity: 1.8<br>SES: 73.4% no profession/student<br>Control n=94<br>Mean age: 29.2 yrs<br>Parity: 0.9<br>SES: 47.9% no profession/student<br>Ethnicity, education: NR	Travel time (mins):<br>≤ 20 mins<br>>20 mins	University Hospital with neonatal care facilities.<br>Universal state provision of maternity care	BBA	NR
RAVELLI STUDY									

For peer review only

Ravelli 2011 (33-35), Netherlands (full papers & abstract**)**	Retrospective population-based cohort study, rural and urban areas, 12 provinces	To study the effect of travel time from home to OU on mortality and other adverse outcomes in pregnant women at term in primary and secondary care	2000 – 2006	*Included:* Singleton term births *Excluded:* Antepartum deaths, congenital anomalies, invalid/missing postcodes or outpatient codes, or births from Wadden islands, home deliveries, hospitals participated for 1-2 yrs	N=751,926 singleton births Age yrs ,% <20, 2 20-34, 78.3 35-39, 17.2 ≥40, 2.4 Parity*:* Nullips: 49.9% Ethnicity*:* White 81.7% SES % *: high 25.2, medium 48.2* , low 26.7 Education: NR	Travel time (mins): < 20 >=20	Universal state provision of maternity care. 99 OUs including tertiary perinatal centres	NM (Combined intrapartum & early & late NM up to 28 days) NM (0-24 hrs) NM (0-27 days) NM(8-27 days); Combined (mortality and/or Apgar <4 at 5min, and/or NNU admission)	NR
Ravelli 2012 (36), Netherlands	Retrospective cohort study in 9 regions	To investigate provincial differences in perinatal mortality and to determine the influence of different risk factors, including travel time from home to	2000-2006	*Included:* Singleton births *Excluded:* Women with incorrect post codes	N=1,242,725 singletons Age yrs, % <20, 1.8 >35, 19.5 Parity, % Nullips, 46.3 Ethnicity*, %* Non-western 16.2 SES low (10th centile): 10% Education NR	Travel time(mins): <20 >=20	Universal state provision of maternity care	PM	NR

For peer review only - http://bmjopen.bmj.com/site/about/guidelines.xhtml
For peer review only

		the OU during labour							
Stolp 2015 (37), Netherlands	Prospective cohort study, rural & urban areas	To assess whether the limit of 45 mins is met for ambulance transfer of women with PPH after home birth	2008-2010	*Included:* Women with PPH after MW supervised home birth *Excluded:* Cases of PPH with missing data	N= 72 (54 analysed) Age median (range) yrs: 31 (23-41); Parity (n,%): Primip 27-50% Ethnicity, Education, SES: NR	Travel time (mins): <45 >45	Home birth for low risk women and hospital birth. Universal state provision of maternity care	NR	Maternal admission to intensive care; (ICU); Blood transfusion; Postpartum haemorrhage (PPH)
ENGJOM STUDY									
Engjom 2017 & 2015 (abstract) (38, 39), Norway	Retrospective population-based cohort study, Medical Birth Registry of Norway and Statistics Norway, 19 counties	To assess peripartum mortality associated with place of birth and availability of obstetric units.	1999-2009	*Included:* All births in Norway with GA >=22 wks or BW>=500g *Excluded:* Lack of address and municipality, antepartum stillbirths, planned home births	N= 646,898 96.4% singletons. Age yrs, % <20 2.4 20-35, 80.7 >35, 16.9%; Multips 58.7%; Education >11y 77.2 Ethnicity: Western 90.7%	Travel time (hrs): <1 1-2 >2	Basic obstetric care for normal delivery; Emergency obstetric care < 1500 births/yr. Universal state provision of maternity care	BBA	NR
**CANADIAN STUDIES**									
Grzybowski & Stroll Study									

For peer review only - http://bmjopen.bmj.com/site/about/guidelines.xhtml

Grzybowski 2011(41), Canada	Retrospective cohort study, rural areas of British Columbia	To document newborn and maternal outcomes in relation to travel time to the nearest OU with CS capability	2000-2004	*Included:* All deliveries >20 weeks' GA *Excluded:* Multiple birth, congenital anomalies or late ToP, core urban areas	*N=35,426 births* *Groups:* *< 1 hr, 1-2, 2-4, >4 hrs* Group N: 32,814, 1359, 747, 506 Mean maternal age yrs: 28.7,28.67, 27.25, 27.2 Parity % primips: 42.6, 38.6, 36.7, 36.8 SES[1]: 0.12, 0.10, 0.30, 0.33, 1st Nations % 0.05, 0.30, 0.23, 0.42 Education: NR	Travel time (hrs): <1 1-2 2-4 >4	Universal medical coverage for core health care, 13 NNUs, 42,000 births /year	Perinatal mortality (SB & early NND); BBA; NNU admission	CS
Grzybowski, 2013 (42), Canada	Retrospective cohort study, rural areas of British Columbia	To compare rural maternity care by level of services	2000 - 2007	*Included:* Singleton births *Excluded:* Women with residential postcode of large urban centres	N=4672 births; Mean age, yrs: 27.7 Parity: primips: 39.7 % SES[1]: 0.22%; Ethnicity: 1st Nations 0.3% Education: NR	Travel time (hrs): <1 >1	Universal medical coverage for core health care	SB; NND (late < 1 month); PM; IM; BBA; NNU admission	CS; Emergency CS; PPH
Grzybowski 2015 (43), Canada	Retrospective cohort study, British Columbia (BC), Alberta, Nova Scotia (NS)	To examine the safety of rural Canadian maternity services	2003-2008	*Included:* Singleton deliveries *Excluded:* Multiple births, infants born with congenital anomalies, planned	*Alberta, BC, NS* Age yrs (n%) <18: 1618 (2.3), 1256 (2.0), 413 (2.2) >35 yrs: 5127 (7.3), 8866 (14.3), 2387 (12.7) Multips n(%) 41730 (59.6), 35089 (56.6), 10656 (56.8)	Travel time (hrs): <1 1-2 2-4 >4	Universal medical coverage for core health care , 20 small maternity closures since 2000	Perinatal mortality (SB & NND up to 7 days)	CS

For peer review only - http://bmjopen.bmj.com/site/about/guidelines.xhtml

				home births, accidental BBA	Ethnicity, SES, education: NR				
Stoll 2014 (44), Canada	Retrospective cohort study, rural British Columbia	To report on characteristics and perinatal outcomes of rural women with only MW involved in care	2003-2008	*Included:* Women residing outside core urban areas, singletons >20wk GA & care by a MW *Excluded:* Late ToP, congenital anomalies	*< 1 hr, 1-2 hrs, > 2hrs:* N= 3438, 124, 130 Mean age yrs: 29.78, 31.4, 30.5 Primips n(%) 1574 (45), 63 (50.8), 63 (48.5) Ethnicity, SES & Education*:* NR	Travel time (hrs): <1 1-2 >2	Universal medical coverage for core health care, closure of 22 rural maternity services	Perinatal mortality (SB & NND up to 7 days)	CS
Darling 2019 (40), Canada	Retrospective population-based cohort study, Ontario	Whether greater diving distances to OU associated with a higher risk of adverse neonatal outcomes	2012-2015	*Included:* Women who planned home births regardless of actual place of births *Excluded:* Multiple births, Preterm < 37 wks	*N= 11,869* *Age yrs,%:* <25, 9.5 25-39,87.6 ≥40, 2.9 Primps n (%) 4208 (35.5) SES low, n(%) 2465(20.8) Ethnicity & education: NR	Travel time (mins): ≤30 >30	Universal medical coverage for core health care	Perinatal mortality (PM); NNU admission; 5-mins Apgar <7	CS
**OTHER COUNTRIES**									

For peer review only - http://bmjopen.bmj.com/site/about/guidelines.xhtml

Aoshima 2011 (45), Japan	Before and after study design, data from perinatal care centres	Whether reducing travel time influences the neonatal mortality rate (NM)	2002-2006	*Included:* All births *Excluded:* Municipalities consisting of isolated islands	Number of births: 2002= 347,284 2006 = 322,514 Other characteristics: NR	Travel time (hrs): <= 1 >1	Universal healthcare insurance system, 346 perinatal care centres	NM	NR

Abbreviations: BBA: Born Before Arrival; Bthwt: Birth Weight; depr: deprive; GA: Gestational Age; IM: Infant Mortality; LBW: low birth weight; MW: midwife; NNU: Neonatal Unit; NM: Neonatal Mortality;NND: neonatal death; OBs: Obstetrician; PM: Perinatal mortality; PPH: postpartum haemorrhage; SB: stillbirth; SES: Socioeconomic status; ToP: Termination of pregnancy .

INSEE: Institute National de la Statistique et des Etudes Economiques; INSEE codes: 1: farmer; 2: craftsperson, merchant or entrepreneur; 3: business executive, intellectual occupation; 4: other professionals; 5: employee; 6: worker; 8: no occupation;

[1]SES: Catchment level Social vulnerability -1 to +1

[2]GPESS = general practitioner with enhanced surgical skills.

For peer review only - http://bmjopen.bmj.com/site/about/guidelines.xhtml

Table 3B: Risk of bias – Travel time

**Author, year, country**	**Study sample selection bias** Additional criteria for case control studies	**Bias in measurement of exposure**	**Bias in measurement of outcomes**	**Attrition bias**	**Analysis method reported and appropriate**	**Potential confounders adjusted for and listed**
**UK STUDIES**						
Dummer & Parker 2004 (27), UK	**LOW** Cumbrian Births Database	**LOW** Modelled using GIS	**LOW** Objective outcome (NM)	**LOW** Of 3,352 live births, 42 stillbirths excluded as the outcome NM	**LOW** Method detailed, results of LR were reported	**LOW** Adjusted for year of birth, social class, birth order, multiple births
Paranjothy 2013 & 2014 (28, 29), UK	**LOW** National Community Child Health Database & All Wales Perinatal Survey (AWPS)	**HIGH** Women's address replaced by population-weighted centroid, travel time calculated using Google Maps API (v3)	**LOW** Objective outcomes (SB, NM)	**LOW** 11% excluded where information on parity was missing	**LOW** Analysis method described and multilevel LR data were reported	**LOW** Adjusted for maternal age, parity, urban/rural location, SES, and other characteristics
**EUROPEAN STUDIES**						
Combier 2013 (30), France	**LOW** Burgundy perinatal network database	**HIGH** Municipality town hall not woman's home address	**LOW** Objective outcomes (SB, PM, BBA)	**LOW** All births identified included in the analysis	**LOW** Method described; hierarchical LR and multilevel LR reported	**LOW** Adjusted for maternal age, urbanization level and other characteristics

For peer review only - http://bmjopen.bmj.com/site/about/guidelines.xhtml

Renesme 2013 (32), France	Case definition	**YES** linked to perinatal network database	**LOW** Distance & travel time estimated using GIS	**LOW** Objective outcomes retrieved from regional and hospital databases	**LOW** 5/81 (6%) BBAs missing, 3/162 (2%) controls missing	**LOW** Method described and univariate and appropriate; multivariate reported	**LOW** Adjusted for age, family status, INSEE maternal occupation, parity, and other characteristics
	Representativeness of cases	**YES** All cases in defined period					
	Appropriate selection of controls	**YES** Controls chosen randomly from same databases and from births occurring at the nearest delivery date and hour to cases					
	Definition of control appropriate	**YES** Outcome could not have occurred					
	Comparability of cases and controls	**NO** Difference in antenatal care attendance					
Nguyen 2016 (31), France	Case definition	**YES** Using medical records	**UNCLEAR** No information	**LOW** Objective outcome (BBA)	**UNCLEAR** No information	**HIGH** No details of the analysis method and analysis was only descriptive	**HIGH** No adjustment for any potential confounders
	Representativeness of cases	**YES** All cases in defined period					

For peer review only - http://bmjopen.bmj.com/site/about/guidelines.xhtml

	Appropriate selection of controls	**YES**<br>Next birth, of equivalent GA					
	Definition of control appropriate	**YES**<br>Outcome could not have occurred					
	Comparability of cases and controls	**NO**<br>Significant differences in parity, smoking, pregnancy monitoring, profession					
Ravelli 2011 (33-35), Netherlands (abstract & full papers)	**LOW**<br>Population based study using the Netherlands Perinatal Registry		**LOW**<br>GIS software used to measure travel time from women's postcodes	**LOW**<br>Objective outcomes from perinatal registry	**LOW**<br>Small proportion (0.3%) of women excluded due to incorrect zip code	**LOW**<br>Method reported; descriptive analysis & LR results given	**LOW**<br>Analysis adjusted for age, parity, ethnicity, SES
Ravelli 2012 (36), Netherlands	**LOW**<br>Population based study using the Netherlands Perinatal Registry		**LOW**<br>GIS software used to measure travel time from women's postcodes	**LOW**<br>Objective outcomes from perinatal registry	**LOW**<br>Small proportion 4% of women excluded	**UNCLEAR**<br>No information	**LOW**<br>Adjusted for age, parity, very urban /very rural, SES
Stolp 2015 (37), Netherlands	**HIGH**<br>Study participants were selected by midwives		**LOW**<br>Ambulance interval includes total time from dispatch call to arrival at hospital	**UNCLEAR**<br>Method of measuring blood loss not reported	**HIGH**<br>Missing data 18/72 (25%) due to incomplete documentation	**HIGH**<br>Data only analysed descriptively	**HIGH**<br>No adjusted analysis

For peer review only

Engjom 2017 & Engjom 2015 (38, 39), Norway (abstract & full paper)	**LOW** Medical Birth Registry of Norway	**LOW** Travel time polygon from home address using GIS	**LOW** Objective outcomes from birth registry	**UNCLEAR** No information	**LOW** Analysis appropriate, details of LR, multilevel modelling were reported	**LOW** Adjusted for maternal age, parity, education, ethnicity
Grzybowski 2011 (41), Canada	**LOW** Population based study using British Columbia Perinatal Health Program	**HIGH** GIS used to create one hour travel zone for each maternity service, but central postal code to the nearest maternity care used	**LOW** Objective outcomes from Perinatal Health Program	**LOW** 0.3% excluded due to incorrect zip code	**LOW** Analysis appropriate, descriptive analysis & hierarchical LR reported	**LOW** Adjusted for maternal age, parity, SES, ethnicity
Grzybowski 2013 (42), Canada	**LOW** Data from Perinatal Data Registry	**HIGH** Community central postal code used not women's home address	**LOW** Objective outcomes from Perinatal Data Registry	**HIGH** Number of women excluded due to incorrect postal address not reported	**LOW** Analysis appropriate descriptive analysis & LR	**LOW** Adjusted for maternal age, parity, lone parent status, ethnicity, SES
Grzybowski 2015 (43), Canada	**LOW** Provincial perinatal registries	**HIGH** Community central point postal code used not women's home address	**LOW** Objective outcomes from Perinatal Data Registries	**UNCLEAR** No information on missing data	**LOW** Analysis appropriate, descriptive analysis & LR reported	**LOW** Adjusted for maternal age, parity

For peer review only - http://bmjopen.bmj.com/site/about/guidelines.xhtml

Stoll 2014 (44), Canada	**LOW**<br>Based on British Columbia Perinatal Database Registry	**LOW**<br>Used GIS and Google maps; travel times were adjusted for travel conditions	**LOW**<br>Objective outcomes (CS)	**LOW**<br>No missing data	**HIGH**<br>Data were only analysed descriptively	**HIGH**<br>No adjusted analysis
Darling 2019 (40), Canada	**LOW**<br>Data from Perinatal Registries	**LOW**<br>Driving time from women's residence using online mapping tool ArcGIS	**LOW**<br>Objective outcomes from Perinatal Data Registries	**LOW**<br>3.7% excluded not being able to calculate distance to nearest hospital	**LOW**<br>Method reported, results of descriptive analysis & LR reported	**LOW**<br>Adjusted for maternal age, parity, gestational age, season, SES
Aoshima 2011 (45), Japan	**LOW**<br>All Japan except for isolated islands outside road network (96.6% of all Medical Service Areas)	**HIGH**<br>Used central point of municipality not home address but analysis based on (larger) Medical Service Areas.	**LOW**<br>Objective outcomes from Medical Service Area databases	**UNCLEAR**<br>No information on missing data	**LOW**<br>Method appropriate, unpaired t-test, difference-in-difference analysis	**HIGH**<br>No adjusted analysis

Abbreviations: BBA: Born Before Arrival; CS: caesarean section; GA gestational age; GIS: Geographical Information System; INSEE: Institute National de la Statistique et des Etudes Economiques; LR: Logistic regression; NM: Neonatal Mortality; NNU: Neonatal Unit; PM: Perinatal Mortality; SES: socioeconomic status; SB: Still Birth.

For peer review only - http://bmjopen.bmj.com/site/about/guidelines.xhtml

Table C3: Outcomes – Travel time

Outcomes	Study, Year, Country	Exposure groups	Participants (N, n, %)			Findings		
**MATERNAL OUTCOMES**								
**Maternal mortality (MM)**	No studies							
**Caesarean section (CS) (overall or intrapartum)**	Grzybowski 2011 (41), Canada	**Travel time (hrs):**<br>**<1**<br>1-2<br>2-4<br>>4				**All CS**		
			**Groups**	**N (35,429)**	**All CS n (%)**	**Groups**	**Crude OR (95%CI)**	**Adjusted OR (95%CI)**
			<1	32814	*8597* (26.2)	<1	1	NR
			1-2	1359	*313* (23)	1-2	*0.84 (0.74, 0.96)*	NR
			2-4	747	*156* (20.9)	2-4	*0.74 (0.62, 0.89)*	NR
			>4	509	*97* (19.06)	>4	*0.66 (0.53, 0.83)*	NR
	Grzybowski 2013 (42), Canada	**Travel time (hrs):**<br><1<br>>1				**All CS**		
			**Groups**	**N (59,386)**	***n* (%)**	**Groups**	**Crude OR (95%CI)**	**Adjusted OR (95%CI)**
			<1	54 714	*14882* (27.20)	<1	1	NR
			>1	4672	*1075* (23.01)	>1	*0.80 (0.75, 0.86)*	NR

For peer review only - http://bmjopen.bmj.com/site/about/guidelines.xhtml

	Grzybowski 2015 (43), Canada	**Travel time (hrs):**<br><1<br>1-2<br>2-4<br>>4				**Alberta CS**		
			**Groups**	**Alberta N (34,453)**	**n%**	**Groups**	**Crude OR (95%CI)**	**Adjusted OR (95%CI)**
			<1 hr	29906	NR	<1	NR	1
			1-2	2940	NR	1-2	NR	0.86 (0.78, 0.94)
			2-4	1297	NR	2-4	NR	0.67 (0.58, 0.77)
			>4	310	NR	>4	NR	0.64 (0.48, 0.87)
						**British Columbia CS**		
			**Groups**	**British Columbia N (42,217)**	**n%**	**Groups**	**Crude OR (95%CI)**	**Adjusted OR (95%CI)**
			<1 hr	39,101	NR	<1	NR	1
			1-2	1892	NR	1-2	NR	0.92 (0.83, 1.03)
			2-4	623	NR	2-4	NR	0.74 (0.61, 0.90)
			>4	601	NR	>4	NR	0.70 (0.57, 0.85)
						**Nova Scotia CS**		
			**Groups**	**Nova Scotia N (17,336)**	**n%**	**Groups**	**Crude OR (95%CI)**	**Adjusted OR (95%CI)**
			<1 hr	15465	NR	<1	NR	1
			1-2	1772	NR	1-2	NR	0.87 (0.77, 0.98)

For peer review only - http://bmjopen.bmj.com/site/about/guidelines.xhtml

			2-4	99	NR	2-4	NR	0.67 (0.40, 1.10)
						>4	NR	-
	Stoll 2014 (44), Canada	**Travel time (hrs):**<br><1<br>1-2<br>>2	**Groups**	**N (3,692)**	**n (%)**	**Groups**	**Crude OR (95%CI)**	**Adjusted OR (95%CI)**
			<1	3438	633 (18.41)	<1	1	NR
			1-2	124	27 (21.80)	1-2	*1.23 (0.80, 1.91)*	NR
			>2	130	26 (20.0)	>2	*1.11 (0.71, 1.72)*	NR
	Darling 2019 (40), Canada	**Travel time (mins):**<br>≤ 30<br>>30	**Groups**	**N**	**n (%)**	**Groups**	***Crude OR (95%CI)***	**Adjusted OR (95%CI)**
			≤ 30	9189	536 (5.83)	≤ 30	*1*	NR
			>30	2236	98 (4.44)	>30	*0.74 (0.59, 0.92)*	NR
**Emergency CS**	Grzybowski 2013 (42), Canada	**Travel time (hrs):**<br><1<br>>1	**Groups**	**N (59,386)**	**n (%)**	**Groups**	***Crude OR (95%CI)***	**Adjusted OR (95%CI)**
			<1	54,714	9247 (16.99)	<1	*1*	NR
			>1	4672	701 (15.00)	>1	*0.80 (0.75, 0.86)*	NR
**Severe perineal trauma (3rd or 4th degree tear)**	No studies							
**Postpartum haemorrhage**	Stolp 2015 (37),	**Travel time (mins):**	**Groups**	**N (54)**	**n (%)**	**Groups**	**Median (range) ml**	

For peer review only - http://bmjopen.bmj.com/site/about/guidelines.xhtml

	Netherlands	<45<br>>45	<45	34	NR	<45	2,000 (1,100–7,000)	
			>45	20	NR	>45	2,050 (1,000–6,000) (P=0.9)	
	Grzybowski 2013 (42), Canada	**Travel time (hrs):**<br><1<br>>1	**Groups**	**N (59,386)**	**n (%)**	**Groups**	**Crude OR (95%CI)**	
			<1	54 714	*3064* (5.6)	<1	1	
			>1	4672	*327* (7.0)	>1	*1.27 (1.13, 1.43)*	
**Maternal admission to intensive care unit**	Stolp 2015 (37), Netherlands	**Travel time (mins):**<br><45<br>>45	**Groups**	**N (54)**	**n (%)**	**Groups**	**Crude OR (95%CI)**	**Adjusted OR (95%CI)**
			≤45	34	1 *(2.94)*	≤45	1	NR
			>45	20	1 *(5.0)*	>45	*1.74 (0.10, 29.39)*	NR
**Maternal blood transfusion**	Stolp 2015 (37), Netherlands	**Travel time (mins):**<br><45<br>>45	**Groups**	**N (54)**	**n (%)**	**Groups**	**Median (range) L**	
			≤45	34	≤45	≤45	0 (0-8)	
			>45	20	>45	>45	2 (0-8)	
**NEONATAL OUTCOMES**								
**Stillbirth (SB) (overall or intrapartum)**	Paranjothy 2014 (29), UK	**Every 15 min increase in travel time** (continuous variable)	**Groups**	**N (412,827)**	**SB n (%)**	**Groups**	**Crude OR (95%CI)**	**Adjusted OR (95%CI)**
			All women	412,827	135 (0.03)	All women	1.29 (1.14, 1.47)	1.13 (0.98, 1.30)

For peer review only - http://bmjopen.bmj.com/site/about/guidelines.xhtml

			Term births in hospital	387,429	85 (*0.02*)	Term births only	1.35 (1.16, 1.57)	1.36 (1.17, 1.59)
			Nullips births in hospital	185,419	69 (*0.04*)	Nullips only	1.33 (1.13, 1.57)	1.21 (1.02, 1.44)
	Combier 2013 (30), France	**Travel time (mins):**<br>≤ 15<br>16-30<br>31-45<br>≥46	**Groups**	**N (111,001)**	**SB n (%)**	**Groups**	**Crude OR (95%CI)**	**Adjusted OR (95%CI)**
			≤ 15	70,427	333 (0.47)	≤ 15	1	1
			16-30	31,792	148 (0.47)	16-30	0.98 (0.81, 1.20)	1.16 (0.96, 1.40)
			31-45	8445	50 (0.59)	31-45	1.25 (0.93, 1.69)	1.31 (0.89, 1.93)
			≥46	337	3 (0.89)	≥46	1.89 (0.60, 5.92)	1.90 (0.70, 5.15)
	Grzybowski 2013 (42), Canada	**Travel time (hrs):**<br><1<br>>1	**Groups**	**N (59,386)**	**SB n (Rate/1000)**	**Groups**	**Crude OR (95%CI)**	**Adjusted OR (95%%CI)**
			<1	54,714	*274* (5.0)	<1	1	1
			>1	4672	*28* (6.0)	>1	*1.20 (0.81, 1.77)*	NR
**Neonatal mortality (NM)**	Dummer 2004 (27), UK	**Travel time (mins):**<br>≤ 17<br>17-35<br>>35	**Groups**	**N (28,7993)**	**Early NM (0-6 days) n (%)**	**Early NM (0-6 days) Groups**	**Crude OR (95%CI)**	**Adjusted OR (95%CI)**
			≤ 17	NR	1850 (NR)	≤ 17	NR	1
			17-35	NR	789 (NR)	17-35	NR	0.97 (0.89-1.06)
			>35	NR	196 (NR)	>35	NR	0.95 (0.81-1.1)

For peer review only - http://bmjopen.bmj.com/site/about/guidelines.xhtml

			**Groups**	**N (28,7993)**	**NM (0-27days) n (%)**	**NM (0-27 days) Groups**	**Crude OR (95%CI)**	**Adjusted OR (95%CI)**
			≤ 17	NR	1854 (NR)	≤ 17	NR	1
			17-35	NR	946 (NR)	17-35	NR	0.96 (0.89-10.4)
			>35	NR	239 (NR)	>35	NR	0.95 (0.83-1.09)
			Groups	**N (28,7993)**	**Post NM (28-1yr) n (%)**	**Post NM (28 days – 1yr) Groups**	**Crude OR (95%CI)**	**Adjusted OR (95%CI)**
			≤ 17	NR	961 (NR)	≤ 17	NR	1
			17-35	NR	400 (NR)	17-35	NR	0.97 (0.86-10.9)
			>35	NR	98 (NR)	>35	NR	0.95 (0.77-1.17)
	Paranjothy 2014 (29), UK	**Every 15 min increase in travel time** (continuous variable)	**Groups**	**N**	**Early NM n (%)**	**Early NM (0-6 days) Groups**	**Crude OR (95%CI)**	**Adjusted OR (95%CI)**
			All women	412,827	609 *(0.15)*	All women	1.37 (1.31, 1.45)	1.13 (1.07, 1.20)
			Term births only	387,429	177 *(0.05)*	Term births only	1.02 (0.86, 1.21)	0.97 (0.80, 1.17)
			Nullips only	185,419	303 *(0.16)*	Nullips only	1.42 (1.33, 1.51)	1.15 (1.06, 1.25)
			**Groups**	**N**	**Late NM n (%)**	**Late NM (7-27 days) Groups**	**Crude OR (95%CI)**	**Adjusted OR (95%CI)**
			All women	412,827	251 *(0.06)*	All women	1.33 (1.23, 1.44)	1.15 (1.05, 1.26)

For peer review only - http://bmjopen.bmj.com/site/about/guidelines.xhtml

			Term births only	387,429	77 *(0.02)*	Term births only	1.24 (1.03, 1.50)	1.34 (1.13, 1.59)
			Nullips only	185,419	116 *(0.06)*	Nullips only	1.31 (1.15, 1.49)	1.11 (0.97, 1.28)
	Ravelli 2011 (33-35), Netherlands	**Travel time (mins):** < 20 mins ≥20 mins	**NM (Combined intrapartum & early neonatal mortality)**					
			**Groups**	**N (1,054,342)**	**NM n (%)**	**Groups**	**Crude OR (95%CI)**	**Adjusted OR (95%CI)**
			< 20	558,181	789 *(0.14)*	< 20 mins	1	1
			≥20	193,745	336 *(0.17)*	≥20 mins	*1.23 (1.08, 1.39)*	1.23 (1.07, 1.41)
			**Low risk women**	**N (120,896)**	**NM n(/1000) 63 (0.05/1000)**	**Low risk women**	**Crude OR (95%CI)**	**Adjusted OR (95%CI)**
			< 20	**NR**	**NR**	< 20	NR	1
			≥20	**NR**	**NR**	≥20	NR	0.8 (0.4–1.7)
			**Low risk women became high risk during labour**	**N (142,824)**	**NM n(/1000) 1.9/1000**	**Low risk women became high risk during labour**	**Crude OR (95%CI)**	**Adjusted OR (95%CI)**
			<20	NR	NR	<20	NR	1
			≥20	NR	NR	≥20	NR	1.23 (1.04, 1.47)
		**Travel time (mins):** <15 15-19				**NM (Combined intrapartum & early & late NM up to 28 days)**		
			**Groups**	**N (751,926)**	**NM n (1125) (%)**	**Groups**	**Crude OR (95%CI)**	**Adjusted OR (95%CI)**

For peer review only - http://bmjopen.bmj.com/site/about/guidelines.xhtml

		≥20	<15	425,952	NR	<15	1	1
			15-19	132,229	NR	15-19	0.97 (0.82, 1.15)	0.94 (0.79, 1.12)
			≥20	193,745	336	≥ 20	1.22 (1.07, 1.39)	1.17 (1.02, 1.36)
						**Neonatal mortality within 24 hrs**		
			**Groups**	**N (751,926)**	**NM within 24 hrs n (255) (%)**	**Groups**	**Crude OR (95%CI)**	**Adjusted OR (95%CI)**
			<20	558,181	NR	<20	1	1
			≥20	193,745	NR	≥ 20	1.52 (1.17, 1.97)	1.51 (1.13, 2.02)
						**Neonatal mortality 0-7 days**		
			**Groups**	**N (751,926)**	**NM 0-7 dys (523) (%)**	**Groups**	**Crude OR (95%CI)**	**Adjusted OR (95%CI)**
			<20	558,181	NR	<20	1	1
			≥20	193,745	NR	≥ 20	1.44 (1.20, 1.72)	1.37 (1.12, 1.67)
						**Neonatal mortality 8-27 days**		
			**Groups**	**N (751,926)**	**NM 8-27 dys (58) (%)**	**Groups**	**Crude OR (95%CI)**	**Adjusted OR(95%CI)**
			<20	558,181	NR	<20	1	1
			≥20	193,745	NR	≥ 20	1.30 (0.74, 2.26)	1.24 (0.67, 2.27)
	Grzybowski 2013 (42),	**Travel time**	**Groups**	**N (59,386)**	**NM n (%)**	Late neonatal mortality age <1 month, no events		

For peer review only - http://bmjopen.bmj.com/site/about/guidelines.xhtml

	Canada	**(hrs):**<br><1<br>>1	<1	54,714	0			
			>1	4672	0			
	Aoshima 2011 (45), Japan	**Travel time (mins):**<br>Median 39.09 (2006)<br>Median 66.99 (2002)	**Groups**	**N**	**NM n (Rate/ 1000)**	**Groups**	**Crude OR (95%CI)**	**Adjusted OR (95%CI)**
			2006	NR	NR (1.28)	39.09	NR	NR
			2002	NR	NR (1.67)	66.99	NR	NR
**Perinatal mortality (PM)**	Combier 2013 (30), France	**Travel time (mins):**<br>≤15<br>16 -30<br>31-45<br>≥46	**Groups**	**N (110,664)**	**PM n (%)**	**Groups**	Crude OR (95%CI)	**Adjusted OR (95%CI)**
			≤15	70,427	452 (0.64)	≤15	1	1
			16-30	31,792	195 (0.61)	16-30	0.96 (0.81, 1.13)	1.08 (0.90, 1.29)
			31-45	8445	59 (0.7.0)	31-45	1.09 (0.83, 1.43)	1.18 (0.86, 1.62)
			≥46	337	4 (1.19)	≥46	1.86 (0.69, 5.01)	1.85 (0.66, 5.19)
	Ravelli 2012 (36)	**Travel time (mins):**<br><20<br>≥20	**Groups**	**N (1,242,725)**	**PM n (Rate/1000)**	**Groups**	**Crude OR (95%CI)**	**Adjusted OR (95%CI)**
			<20	*1,006,607*	81 (0.08)	<20	1	1
			≥20	*236,118*	19 (0.08)	≥ 20	1.53 (1.47,1.50)	1.66 (1.59,1.74)
	Grzybowski 2011(41), Canada	**Travel time (hrs):**<br><1<br>1-2<br>2-4<br>>4	**Groups**	**N (35,429)**	**PM n (Rate/1000)**	**Groups**	**Crude OR (95%CI)**	**Adjusted OR (95%CI)**
			<1	32,814	*197* (6.0)	<1	1	1
			1-2	1359	*8* (6.0)	1-2	0.98 (0.48, 1.99)	1.04 (0.48, 2.22)

For peer review only - http://bmjopen.bmj.com/site/about/guidelines.xhtml

			2-4	747	*4* (5.0)	2-4	0.89 (0.33, 2.40)	0.92 (0.33, 2.53)
			>4	509	*9* (18.0)	>4	2.98 (1.52, 5.85)	3.17 (1.45, 6.95)
			PM (stillbirths & early neonatal mortality)					
	Grzybowski 2013 (42), Canada	**Travel time (hrs):**<br><1<br>>1	**Groups**	**N (59,386)**	**PM n (Rate/1000)**	**Groups**	**Crude OR (95%CI)**	**Adjusted (OR 95%CI)**
			< 1	54 714	*383* (7.0)	<1	1	NR
			>1	4672	*37* (8.0)	>1	*1.54 (1.09, 2.17)*	NR
	Grzybowski 2015 (43), Canada	**Travel time (hrs):**<br><1<br>1-2<br>2-4<br>>4				**PM (stillbirths & early neonatal mortality) Alberta**		
			**Groups**	**Alberta (N=34,453)**	**PM n (%)**	**Groups**	**Crude OR (95%CI)**	**Adjusted OR (95%CI)**
			<1 hr	29,906	NR	<1	1	1
			1-2	2940	NR	1-2	NR	1.50 (1.03, 2.18)
			2-4	1297	NR	2-4	NR	1.35 (0.77, 2.38)
			>4	310	NR	>4	NR	1.40 (0.44, 4.39)
						**PM (stillbirths & early neonatal mortality) BC**		
			**Groups**	**British Columbia (N=42,317)**	**PM n (%)**	**Groups**	**Crude OR (95%CI)**	**Adjusted OR (95%CI)**
			<1 hr	39,101	NR	<1	1	1

For peer review only - http://bmjopen.bmj.com/site/about/guidelines.xhtml

			1-2	1892	NR	1-2	NR	0.79 (0.43, 1.45)
			2-4	623	NR	2-4	NR	1.33 (0.59, 3.01)
			>4	601	NR	>4	NR	2.84 (1.58, 5.10)
						**PM (stillbirths & early neonatal mortality) Nova Scotia**		
			**Groups**	**Nova Scotia (N= 17,336)**	**PM n (%)**	**Groups**	**Crude OR (95%CI)**	**Adjusted OR (95%CI)**
			<1 hr	15,465	NR	<1	1	1
			1-2	1772	NR	1-2	NR	0.66 (0.38, 1.14)
			2-4	99	NR	2-4	NR	NR
			>4	0	NR	>4	NR	-
	Stoll 2014 (44), Canada	**Travel time (hrs):**<br><1<br>1-2<br>>2	**Groups**	**N (3,692)**	**PM n (%)**	**Groups**	**Crude OR (95%CI)**	**Adjusted OR (95%CI)**
			<1	3438	15 (0.4)	<1	1	NR
			1-2	124	0	1-2	*0.89 (0.05, 14.91)*	NR
			>2	130	2 (1.5)	>2	*3.57 (0.81, 15.76)*	NR
			PM (Stillbirth and early neonatal death up to 7 days)					
	Darling 2019 (40), Canada	**Travel time (mins):**<br>≤ 30<br>>30	**Groups**	**N (10,681)**	**PM n (%)**	**Groups**	***Crude OR (95%CI)***	**Adjusted OR (95%CI)**
			≤ 30	NR	NR	≤ 30	NR	NR

For peer review only - http://bmjopen.bmj.com/site/about/guidelines.xhtml

			>30	NR	NR	>30	NR	NR<br>Reported as RR<br>2.2 (0.67, 7.43)
**Infant mortality (IM)**	Grzybowski 2013 (42), Canada	*Travel time (hrs):*<br><1<br>>1				**IM (age 1-12 month)**		
			**Groups**	**N (59,386)**	**IM n(Rate/1000)**	**Groups**	**Crude OR (95%CI)**	**Adjusted OR (95%CI)**
			<1	54,714	*109* (2.0)	<1	1	NR
			>1	4672	*14* (3.0)	>1	*1.51 (0.86, 2.63)*	NR
**Born before arrival (BBA)**	Combier 2013 (30), France	Travel time (mins):<br><15<br>15-29<br>30-44<br>>45	**Groups**	**N (111,001)**	**BBA n (%)**	**Groups**	**Crude OR (95%CI)**	**Adjusted OR (95%CI)**
			<16	70,427	132 (0.19)	<16	1	1
			16-30	31,792	93 (0.29)	16-30	1.56 (1.20, 2.04)	1.73 (1.23, 2.46)
			31-45	8445	29 (0.34)	31-45	1.84 (1.23, 2.75)	1.64 (1.06, 2.54)
			>45	337	0	>45	-	-
	Renesme 2013 (32), France	**Travel time (mins):**<br><15<br>15-29<br>30-44<br>>45	**Groups**	**Cases N (73) (%)**	**Control N(148) (%)**	**Groups**	**Crude OR (95%CI)**	**Adjusted OR(95%CI)**
			<15	22 (30.2)	59 (39.9)	<15	1	1
			15-29	33 (45.2)	64 (43.2)	15-29	*1.79 (0.87, 3.68)*	1.92 (0.86-4.96)
			30-44	9 (12.3)	18 (12.2)	30-44	*1.68 (0.58, 4.87)*	1.10 (0.35-3.48)
			>45	9 (12.3)	7 (4.7)	>45	*5.89 (1.12, 30.89)*	6.18 (1.33-8.65)

For peer review only

	Nguyen 2016 (31), France	**Travel time (mins):** ≤ 20 > 20	**Groups**	**N (188)**	**BBA n (%)**	**Groups**	**Crude OR (95%CI)**	**Adjusted OR(95%CI)**
			>20	94 controls	22 (23.4)	>20 controls	1	NR
			>20	94 cases	27 (28.7)	>20 cases	*1.3 (0.7, 2.6)*	NR
	Engjom 2017 (39), Norway	**Travel time (hrs):** <1 1-2 >2	**Groups**	**N (646,898)**	**BBA n (%)**	**Groups**	**Crude OR (95%CI)**	**Adjusted OR (95%CI)**
			1	615,896	3488 (0.60)	1	1	1
			1-2	25,494	844 (3.31)	1-2	*6.01 (5.57, 6.49)*	NR reported as RR* 5.3 (5.0,5.8)
			>2	5508	246 (4.50)	>2	*8.21 (7.19, 9.37)*	NR reported as RR* 7.2 (6.3,8.2)
	Grzybowski 2011 (41), Canada	**Travel time (hrs):** <1 1-2 2-4 >4	**Groups**	**N (35,429)**	**BBA n (%)**	**Groups**	**Crude OR (95%CI)**	**Adjusted OR (95%CI)**
			<1	32,814	*66* (0.20)	<1	1	1
			1-2	1359	*31* (2.30)	1-2	*11.58 (7.53, 17.81)*	6.41(3.69,11.28)
			2-4	747	*3* (0.3)	2-4	*2.00 (0.63, 6.38)*	0.92 (0.22, 3.88)
			>4	506	*7* (1.4)	>4	*6.96 (3.18, 15.25)*	3.63 (1.40, 9.40)
	Grzybowski 2013 (42), Canada	**Travel time (hrs):** <1 >1	**Groups**	**N (59,386)**	**BBA n (%)**	**Groups**	**Crude OR (95%CI)**	**Adjusted OR (95%CI)**
			<1	54,714	*164* (0.3)	<1	1	NR
			>1	4672	*70* (1.5)	>1	*5.06 (3.82, 6.70)*	NR
**Combined mortality &**	Ravelli 2011 (33-35),	**Travel time (mins):**	**Groups**	**N(751,926)**	**Event n (4,543) (%)**	**Groups**	**Crude OR(95%CI)**	**Adjusted OR (95%CI)**

For peer review only - http://bmjopen.bmj.com/site/about/guidelines.xhtml

**or Apgar < 4 at 5 mins & or transfer to NICU**	Netherlands	<15<br>15-19<br>>=20	<15	425,952	NR	<15	1	1
			15-20	132,229	NR	15-20	0.99 (091-1.07)	1.11 (1.02-1.21)
			>=20	193,745	NR	>=20	1.11 (1.04-1.19)	1.27 (1.17-1.38)
**Neonatal Unit admission (NNU)**	Grzybowski 2011 (41), Canada	Travel time (hrs):<br><1<br>1-2<br>2-4<br>>4	**NICU level 2 admissions per 1000 births (2001-2004)**			**NICU 2**		
			**Groups**	**N (35,429)**	**NICU2 n (Rate/1000)**	**Groups**	**Crude OR (95%CI)**	**Adjusted OR (95%CI)**
			<1	32,814	*1082* (33.0)	<1	1	1
			1-2	1359	*69* (51.0)	1-2	*1.57 (1.22, 2.01)*	2.20 (1.59, 3.05)
			2-4	747	*8* (11.0)	2-4	*0.32 (0.16, 0.64)*	0.31 (0.14, 0.65)
			>4	506	*14* (27.0)	>4	*0.83 (0.49, 1.42)*	1.07 (0.54, 2.12)
			**NICU level 3 per 1000 births (2001-2004)**			**NICU 3**		
			**Groups**	**N (34,920)**	**NICU3 n (Rate/1000)**	**Groups**	**Crude OR (95%CI)**	**Adjusted OR (95%CI)**
			<1	32,814	*98* (3.0)	<1	1	NR
			1-2	1359	*11* (8.0)	1-2	*2.72 (1.46, 5.09)*	NR
			2-4	747	*4* (5.0)	2-4	*1.80 (0.66, 4.90)*	NR
			>4	509	*2* (4.0)	>4	*1.32 (0.32, 5.35)*	NR
	Grzybowski 2013 (42),	**Travel time (hrs):**	**NICU level 2 (2001-2 & 2006-7) n=74697**			**NICU 2**		

For peer review only

	Canada	<1<br>>1	**Groups**	**N**	**NICU2 n (Rate/1000)**	**Groups**	**Crude OR (95%CI)**	**Adjusted OR (95%CI)**
			<1	54,714	*1751* (32.0)	<1	1	NR
			>1	4672	*154* (33.0)	>1	*1.03 (0.87, 1.22)*	NR
			**NICU level 3 (2001-2 & 2006-7) n=74697**			**NICU 3**		
			**Groups**	**N**	**NICU3 n(Rate/1000)**	**Groups**	**Crude OR (95%CI)**	**Adjusted OR (95%CI)**
			<1	54,714	*219* (4.0)	<1	1	NR
			>1	4672	*28* (6.0)	>1	*1.50 (1.01, 2.23)*	NR
			**NICU (2&3)**			**NICU admission**		
			**Groups**	**N (59,386)**	**NICU n (Rate/1000)**	**Groups**	**Crude OR (95%CI)**	**Adjusted OR (95%CI)**
			<1	54,714	*1970 (36.0)*	<1	1	NR
			>1	4672	*182 (39.0)*	>1	*1.09 (0.93, 1.27)*	NR
	Darling 2019 (40), Canada	**Travel time (mins):**<br>≤ 30<br>>30	**Groups**	**N (10,687)**	**NICU n (%)**	**Groups**	**Crude OR (95%CI)**	**Adjusted OR (95%CI)**
			≤ 30	NR	NR	≤ 30	NR	1
			>30	NR	NR	>30	NR	NR<br>Reported as RR*<br>0.6 (0.44, 0.81)
**Apgar <7 at 5 mins**	Darling 2019 (40), Canada	**Travel time (mins):**	**Groups**	**N (10,578)**	**Apgar <7 n (%)**	**Groups**	**Crude RR (95%CI)**	**Adjusted OR (95%CI)**

For peer review only - http://bmjopen.bmj.com/site/about/guidelines.xhtml

		≤ 30<br>>30	≤ 30	NR	NR	≤ 30	NR	1
			>30	NR	NR	>30	NR	NR<br>Reported as RR*<br>1.02 (0.95, 1.10)
			**Nullips**	**N (4208)**	**Apgar <7 n (%)**	**Groups**	**Crude OR (95%CI)**	**Adjusted OR (95%CI)**
			≤ 30	3425	51 (1.5)	≤ 30	1	NR
			>30	621	14 (2.3)	>30	*1.53 (0.84, 2.77)*	NR
			**Mullips**	**N (7661)**	**Apgar <7 n (%)**	**Groups**	**Crude OR (95%CI)**	**Adjusted OR (95%CI)**
			≤ 30	5764	30 (0.5)	≤ 30	*1*	NR
			>30	1615	11 (0.7)	>30	*1.31 (0.66, 2.62)*	NR
**HIE**	No studies reported							

Abbreviations: BW: Birth Weight; NR: not reported; Nullips: Nulliparous; NICU: Neonatal Intensive Care Unit; * RR: relative risk.

For peer review only

For peer review only - http://bmjopen.bmj.com/site/about/guidelines.xhtml

References:

1. Jordan H, Roderick P, Martin D, Barnett S. Distance, rurality and the need for care: access to health services in South West England. International Journal of Health Geographics. 2004;3(1):21.
2. Kelly C, Hulme C, Farragher T, Clarke G. Are differences in travel time or distance to healthcare for adults in global north countries associated with an impact on health outcomes? A systematic review. BMJ open. 2016;6(11):e013059-e.
3. Rashidian A, Omidvari AH, Vali Y, Mortaz S, Yousefi-Nooraie R, Jafari M, et al. The effectiveness of regionalization of perinatal care services - a systematic review. Public Health. 2014;128(10):872-85.
4. Price Sian. Research evidence review -impact of distance/travel time to maternity services on birth outcomes. Public Health Wales NHS Trust 2015.
5. Stroup DF, Berlin JA, Morton SC, Olkin I, Williamson GD, Rennie D, et al. Meta-analysis of Observational Studies in EpidemiologyA Proposal for Reporting. JAMA. 2000;283(15):2008-12.
6. Merenstein G, Glicken A. Best Evidence-Based Practices: A Historic Perspective. Neonatal Network. 2002(5):31-5.
7. World Health Organization. Universal health coverage (UHC). https://wwwwhoint/news-room/fact-sheets/detail/universal-health-coverage-(uhc). 2019.
8. Rowe R, The Birthplace in England Collaborative Group, ., McCourt, C. and MacFarlane, A, . Birthplace terms and definitions: consensus process Birthplace in England research programme. (Final Report 2. 08/1604/140). Southampton: HMSO. http://openaccesscityacuk/3651/1/Birthplace%20definitions%20rpt%20SDO_FR2_08-1604-140_V02pdf. 2011.
9. Cochrane Effective Practice and Organisation of Care (EPOC). Suggested risk of bias cri teria for EPOC reviews. EPOC Resources fo r review authors. Available at: http ://ep occochraneorg/res ources /epoc-res ources-revi ew-authors. 2017.
10. Stang A. Critical evaluation of the Newcastle-Ottawa scale for the assessment of the quality of nonrandomized studies in meta-analyses. European journal of epidemiology. 2010;25(9):603-5.
11. Blondel BD, Nicolas; Pilkington, Hugo; Zeitlin, Jennifer. Out-of-hospital births and the supply of maternity units in France. Health & Place. 2011;17(5):1170-3.
12. Fleming AM, E. A.; Schram, C. M. H. Reducing caesarean section rates through choice and collaboration. Archives of Disease in Childhood: Fetal and Neonatal Edition Conference: 16th Annual Conference of the British Maternal and Fetal Medicine Society Dublin Ireland Conference Publication:. 2013;98(no pagination).
13. Mackie FLA, F.; Moise, J.; Amu, O. Maternal and neonatal outcomes after the amalgamation of two maternity units and consequent increased consultant labour ward presence: A retrospective population-based study. Archives of Disease in Childhood: Fetal and Neonatal Edition. 2014;99:A21-A2.
14. Engjom HMM, N. H.; Norheim, O. F.; Klungsoyr, K. Availability and access in modern obstetric care: a retrospective population-based study. BJOG: An International Journal of Obstetrics & Gynaecology. 2014;121(3):290-9.
15. Grytten JM, Lars; Skau, Irene; Sorensen, Rune. Regionalization and local hospital closure in Norwegian maternity care--the effect on neonatal and infant mortality. Health Services Research. 2014;49(4):1184-204.
16. Hemminki EH, A.; Gissler, M. Should births be centralised in higher level hospitals? Experiences from regionalised health care in Finland. BJOG: An International Journal of Obstetrics & Gynaecology. 2011;118(10):1186-95.
17. Allen VM, Jilwah N, Joseph KS, Dodds L, O'Connell CM, Luther ER, et al. The Influence of Hospital Closures in Nova Scotia on Perinatal Outcomes. Journal of Obstetrics and Gynaecology Canada. 2004;26(12):1077-85.

For peer review only - http://bmjopen.bmj.com/site/about/guidelines.xhtml

18. Hutcheon J, A.; Riddell Corinne, A.; Strumpf Erin, C.; Lee, Lily; Harper, Sam. Safety of labour and delivery following closures of obstetric services in small community hospitals. CMAJ Canadian Medical Association Journal. 2017;189(11):E431-E6.
19. Le Coutour I-R, C.; Danzon, A. [Regionalization of health care and obstetric practice]. Revue d Epidemiologie et de Sante Publique. 1990;38(3):211-20.
20. Allen V, M.; Jilwah, Nadia; Joseph, K. S.; Dodds, Linda; O'Connell Colleen, M.; Luther Edwin, R.; Fahey, T. John; Attenborough, Rebecca; Allen Alexander, C. The influence of hospital closures in Nova Scotia on perinatal outcomes. Journal of Obstetrics & Gynaecology Canada: JOGC. 2004;26(12):1077-85.
21. Bhoopalam PS, Watkinson M. Babies born before arrival at hospital. BJOG: An International Journal of Obstetrics & Gynaecology. 1991;98(1):57-64.
22. Pasquier JCM, M.; Bagouet, S.; Moret, S.; Luo, Z. C.; Rabilloud, M.; Gaucherand, P.; Robert-Gnansia, E. Effects of residential distance to hospitals with neonatal surgery care on prenatal management and outcome of pregnancies with severe fetal malformations. Ultrasound in Obstetrics & Gynecology. 2007;29(3):271-5.
23. Pilkington HB, Beatrice; Drewniak, Nicolas; Zeitlin, Jennifer. Where does distance matter? Distance to the closest maternity unit and risk of foetal and neonatal mortality in France. European Journal of Public Health. 2014;24(6):905-10.
24. Fougner BN, J. [Deliveries during transportation and shortly after admission to hospital]. Tidsskrift for Den Norske Laegeforening. 2000;120(11):1288-90.
25. Ovaskainen KO, Riitta; Gissler, Mika; Luukkaala, Tiina; Tammela, Outi. Out-of-hospital deliveries have risen involving greater neonatal morbidity: Risk factors in out-of-hospital deliveries in one University Hospital region in Finland. Acta Paediatrica. 2015;104(12):1248-52.
26. Lisonkova S, Sheps SB, Janssen PA, Lee SK, Dahlgren L, MacNab YC. Birth Outcomes Among Older Mothers in Rural Versus Urban Areas: A Residence-Based Approach. The Journal of Rural Health. 2011;27(2):211-9.
27. Dummer TJBP, L. Hospital accessibility and infant death risk. Archives of Disease in Childhood. 2004;89(3):232-4.
28. Paranjothy SW, W. J.; Rolfe, K.; Gong, Y.; Adappa, R.; Dunstan, F.; Kotecha, S. Perinatal outcomes and travel time to maternity services: Analysis of birth outcome data in Wales from 1995 to 2009. Archives of Disease in Childhood: Fetal and Neonatal Edition Conference: 16th Annual Conference of the British Maternal and Fetal Medicine Society Dublin Ireland Conference Publication:. 2013;98(no pagination).
29. Paranjothy SW, W. John; Rolfe, Kim; Adappa, Roshan; Gong, Yi; Dunstan, Frank; Kotecha, Sailesh. Perinatal outcomes and travel time from home to hospital: Welsh data from 1995 to 2009. Acta Paediatrica. 2014;103(12):e522-7.
30. Combier EC, Helene; Le, Vaillant; Marc,; Michaut, Francis; Ferdynus, Cyril; Amat-Roze, Jeanne-Marie; Gouyon, Jean-Bernard; Quantin, Catherine; Zeitlin, Jennifer. Perinatal health inequalities and accessibility of maternity services in a rural French region: closing maternity units in Burgundy. Health & Place. 2013;24:225-33.
31. Nguyen MLL, P.; Dreyfus, M. [Maternal and neonatal outcomes of unplanned deliveries]. Journal de Gynecologie, Obstetrique et Biologie de la Reproduction. 2016;45(1):86-91.
32. Renesme L, Garlantézec R, Anouilh F, Bertschy F, Carpentier M, Sizun J. Accidental out-of-hospital deliveries: a case–control study. Acta Paediatrica. 2013;102(4):e174-e7.
33. Ravelli ACJJ, K. J.; De, Groot; M, H.; Rijninks-Van, Driel; G, C.; Tromp, M.; Eskes, M.; Willem, B.; Mol, J.; Abu-Hanna, A.; Jaap, J.; Erwich, H. M. Travel time from home to hospital and intrapartum and neonatal mortality in term pregnant women. Journal of Maternal-Fetal and Neonatal Medicine. 2010;23:234.
34. Ravelli ACJJ, K. J.; De, Groot; M, H.; Erwich, J. J. H. M.; Rijninks-Van, Driel; G, C.; Tromp, M.; Eskes, M.; Abu-Hanna, A.; Mol, B. W. J. Travel time from home to hospital and adverse perinatal

For peer review only - http://bmjopen.bmj.com/site/about/guidelines.xhtml

outcomes in women at term in the Netherlands. Obstetrical and Gynecological Survey. 2011;66(7):396-8.
35. Ravelli ACJJ, K. J.; de, Groot; M, H.; Erwich, J. J. H. M.; Rijninks-van, Driel; G, C.; Tromp, M.; Eskes, M.; Abu-Hanna, A.; Mol, B. W. J. Travel time from home to hospital and adverse perinatal outcomes in women at term in the Netherlands. BJOG: An International Journal of Obstetrics & Gynaecology. 2011;118(4):457-65.
36. Ravelli AEM, Erwich JJHM, Brouwers HAA, Abu-Hanna A, Kerkhof FGM, van der Post JAM, H.O.PC., Neoned* & B.W.J. Mol. Perinatale sterfteverschillen in de negen perinatale zorgregio's Nederlands tijdschrift voor Obstetrie & Gynaecologie 2012 125, (augustus 2012):270-7.
37. Stolp IS, Marrit; Luxemburg, Sanne; Akker, Thomas; Waard, Jan; Roosmalen, Jos; Vos, Rien. Ambulance Transfer in Case of Postpartum Hemorrhage after Birth in Primary Midwifery Care in The Netherlands: A Prospective Cohort Study. Birth: Issues in Perinatal Care. 2015;42(3):227-34.
38. Engjom HM, N. H.; Hoydal, E.; Norheim, O. F.; Klungsoyr, K. Obstetric health system structure and perinatal outcomes in Norway. International Journal of Gynecology and Obstetrics. 2015;131:E487-E8.
39. Engjom HM, Morken N-H, Høydahl E, Norheim OF, Klungsøyr K. Increased risk of peripartum perinatal mortality in unplanned births outside an institution: a retrospective population-based study. American Journal of Obstetrics and Gynecology. 2017;217(2):210.e1-.e12.
40. Darling EK, Lawford KMO, Wilson K, Kryzanauskas M, Bourgeault IL. Distance from Home Birth to Emergency Obstetric Services and Neonatal Outcomes: A Cohort Study. Journal of Midwifery & Women's Health. 2019;64(2):170-8.
41. Grzybowski SS, Kathrin; Kornelsen, Jude. Distance matters: a population based study examining access to maternity services for rural women. BMC Health Services Research. 2011;11:147.
42. Grzybowski SS, Kathrin; Kornelsen, Jude. The outcomes of perinatal surgical services in rural British Columbia: a population-based study. Canadian Journal of Rural Medicine. 2013;18(4):123-9.
43. Grzybowski SF, John; Lai, Barbara; Zhang, Sharon; Aelicks, Nancy; Leung Brenda, M.; Stoll, Kathrin; Attenborough, Rebecca. The safety of Canadian rural maternity services: a multi-jurisdictional cohort analysis. BMC Health Services Research. 2015;15:410.
44. Stoll KK, Jude. Midwifery Care in Rural and Remote British Columbia: A Retrospective Cohort Study of Perinatal Outcomes of Rural Parturient Women With a Midwife Involved in Their Care, 2003 to 2008. Journal of Midwifery & Women's Health. 2014;59(1):60-6.
45. Aoshima KK, H.; Kawahara, K. Neonatal mortality rate reduction by improving geographic accessibility to perinatal care centers in Japan. Journal of Medical and Dental Sciences. 2011;58(2):29-40.
46. Pilkington HP, Caroline; Blondel, Beatrice; Charreire, Helene; Combier, Evelyne; Le, Vaillant; Marc,; Amat-Roze, Jeanne-Marie; Zeitlin, Jennifer. Travel Time to Hospital for Childbirth: Comparing Calculated Versus Reported Travel Times in France. Maternal & Child Health Journal. 2017;05:05.

For peer review only

For peer review only - http://bmjopen.bmj.com/site/about/guidelines.xhtml

PRISMA Flow Diagram

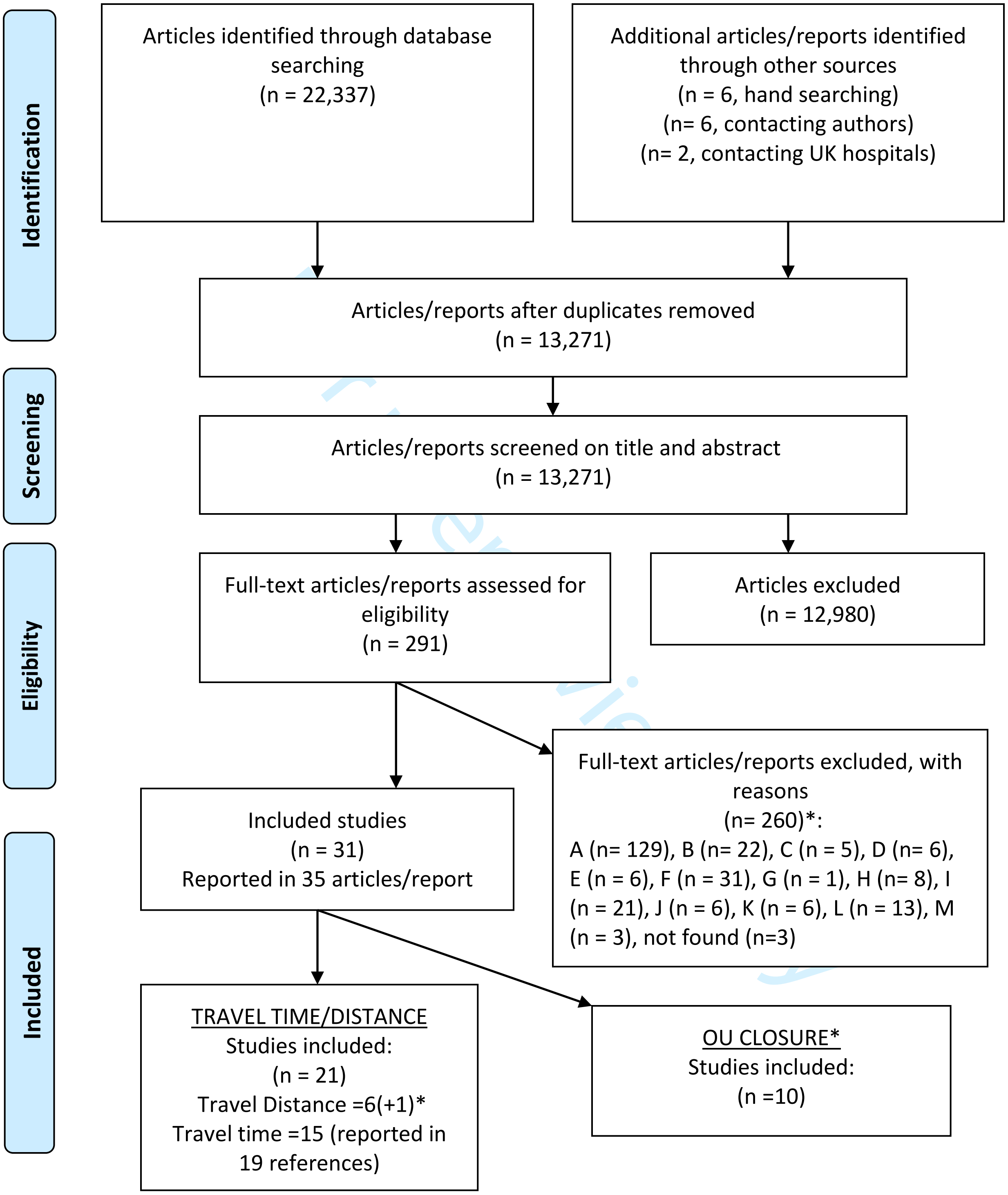

***One study, Blondel 2011 contribute data to both travel distance and closure review questions**

From: Moher D, Liberati A, Tetzlaff J, Altman DG, The PRISMA Group (2009). *P*referred *R*eporting *I*tems for *S*ystematic Reviews and *M*eta-*A*nalyses: The PRISMA Statement. PLoS Med 6(7): e1000097. doi:10.1371/journal.pmed1000097

For more information, visit www.prisma-statement.org.

Reasons for inclusion:

A. Different study designs
B. Studies comparing different models of maternity care
C. Studies comparing different hospital volumes
D. Studies comparing different levels of care
E. Studies comparing different hospital types (private vs. public, local vs. central)
F. Transfer from primary to secondary birth centres
G. Cost of care
H. Neonatal transfer
I. Regionalisation
J. Not in high-income countries or in the USA
K. Distance or travel time proximity
L. No outcomes
M. Modelling studies
N. Not found

For peer review only

From: Moher D, Liberati A, Tetzlaff J, Altman DG, The PRISMA Group (2009). *P*referred *R*eporting *I*tems for *S*ystematic Reviews and *M*eta-*A*nalyses: The PRISMA Statement. PLoS Med 6(7): e1000097. doi:10.1371/journal.pmed1000097

For more information, visit www.prisma-statement.org.

Appendix 1: Current high-income countries (OECD) members

- Australia
- Austria
- Belgium
- Canada
- Chile
- Czech Republic
- Denmark
- Estonia
- Finland
- France
- Germany
- Greece
- Hungary
- Iceland
- Ireland
- Israël
- Italy
- Japan
- Korea
- Latvia
- Luxembourg
- Mexico
- Netherlands
- New Zealand
- Norway
- Poland
- Portugal
- Slovak Republic
- Slovenia
- Spain

For peer review only

- Sweden
- Switzerland
- Turkey
- United Kingdom
- United States

http://www.oecd.org/about/membersandpartners/

For peer review only

Appendix 2: MEDLINE search results in October 2017

1	Maternal Health Services/	12707
2	Delivery Rooms/	1417
3	Intensive Care Units, Neonatal/ and Intensive Care, Neonatal/	860
4	exp Hospitals/ and (exp Pregnancy/ or exp Delivery, Obstetric/ or Prenatal Care/ or Perinatal Care/ or Postnatal Care/)	10061
5	exp Hospitals/ and (foetal or fetal or foetus or fetus or newborn* or neonat* or infant* or baby or babies or maternal or maternity or pregnant or pregnancy or pregnancies or prenatal or pre-natal or antenatal or ante-natal or perinatal or peri-natal or postnatal or post-natal).ti,ab.	13308
6	(Community Health Services/ or Rural health Services/) and (exp Pregnancy/ or exp Delivery, Obstetric/ or Prenatal Care/ or Perinatal Care/ or Postnatal Care/)	2130
7	(Community Health Services/ or Rural health Services/) and (foetal or fetal or foetus or fetus or newborn* or neonat* or infant* or baby or babies or maternal or maternity or pregnant or pregnancy or pregnancies or prenatal or pre-natal or antenatal or ante-natal or perinatal or peri-natal or postnatal or post-natal).ti,ab.	2436
8	((maternity or obstetric?) adj3 (service? or hospital? or ward? or unit? or department? or dept? or centre? or center?)).ti,ab.	20646
9	((maternity or maternal or maternal health or obstetric) adj care).ti,ab.	8066
10	((delivery or birth*) adj (service? or hospital? or ward? or unit? or department? or dept? or centre? or center?)).ti,ab.	2990
11	((delivery or birth) adj care).ti,ab.	797
12	((foetal or fetal or foetus or fetus or newborn* or neonat* or infant* or baby or babies or maternal or maternity or pregnant or pregnancy or pregnancies or prenatal or pre-natal or antenatal or ante-natal or perinatal or peri-natal or postnatal or post-natal) adj3 (service? or hospital? or ward? or unit? or department? or dept? or centre? or center?)).ti,ab.	49515
13	((foetal or fetal or foetus or fetus or newborn* or neonat* or infant* or baby or babies or maternal or maternity or pregnant or pregnancy or pregnancies or prenatal or pre-natal or antenatal or ante-natal or perinatal or peri-natal or postnatal or post-natal) adj care).ti,ab.	29272
14	(((communit* or rural or district? or local*) adj3 (service? or hospital? or ward? or unit? or department? or dept? or centre? or center?)) and (foetal or fetal or foetus or fetus or newborn* or neonat* or infant* or baby or babies or maternal or maternity or pregnant or pregnancy or pregnancies or prenatal or pre-natal or antenatal or ante-natal or perinatal or peri-natal or postnatal or post-natal)).ti,ab.	7602
15	(((communit* or rural or district? or local*) adj care) and (foetal or fetal or foetus or fetus or newborn* or neonat* or infant* or baby or babies or maternal or maternity or pregnant or pregnancy or pregnancies or prenatal or pre-natal or antenatal or ante-natal or perinatal or peri-natal or postnatal or post-natal)).ti,ab.	141
16	(((level 1 or level i or level one or level 2 or level ii or level two or level 3 or level iii or level three) adj5 (service? or hospital? or ward? or unit? or department? or dept? or centre? or center? or care)) and (foetal or fetal or foetus or fetus or newborn* or neonat* or infant* or maternal or maternity or pregnant or pregnancy or prenatal or pre-natal or antenatal or ante-natal or perinatal or peri-natal or postnatal or post-natal)).ti,ab.	1408
17	1 or 2 or 4 or 5 or 6 or 7 or 8 or 9 or 10 or 11 or 12 or 13 or 14 or 15 or 16	108968
18	regional health planning/ or "catchment area (health)"/	12545

For peer review only

19	"Transportation of Patients"/	8903
20	Transportation/	8928
21	Health Services Accessibility/	65383
22	Travel/	23261
23	(travel* adj5 (distance or time)).ti,ab.	7255
24	("distance to" adj5 (hospital? or service? or unit? or ward? or centre? or center?)).ti,ab.	5849
25	("time to" adj5 (hospital? or service? or unit? or ward? or centre? or center?)).ti,ab.	37417
26	(("travel to" or "travel?ing to") adj5 (hospital? or service? or unit? or ward? or centre? or center?)).ti,ab.	1394
27	(("transfer to" or "transferred to") adj5 (hospital? or service? or unit? or ward? or centre? or center?)).ti,ab.	13476
28	(("transport to" or "transported to" or "transportation to") adj5 (hospital? or service? or unit? or ward? or centre? or center?)).ti,ab.	6259
29	(("access to" or accessibility) adj5 (hospital? or service? or unit? or ward? or centre? or center?)).ti,ab.	22957
30	(access* or travel* or distance* or transport* or transfer*).ti.	360710
31	((regional* or central or decentral*) adj5 (hospital? or service? or unit? or ward? or centre? or center?)).ti,ab.	36894
32	(regionali?e* or regionali?ation or centrali?e* or centrali?ation or decentrali?e or decentrali?ation).ti,ab.	18456
33	(geographic adj5 (hospital? or service? or unit? or ward? or centre? or center?)).ti,ab.	1951
34	((geographic* or district? or residence) adj5 area?).ti,ab.	24860
35	catchment*.ti,ab.	10031
36	((small or medium or large) adj2 (hospital? or unit?)).ti,ab.	14557
37	(small volume or medium volume or high volume).ti,ab.	18240
38	Health Facility Closure/	2317
39	(close or closed or closing or closure? or discontinu*).ti,ab.	609722
40	(preclos* or pre-clos* or postclos* or post-clos*).ti,ab.	406
41	downsiz*.ti,ab.	1966
42	(midwifery led adj2 (service? or unit? or ward? or centre? or center?)).ti,ab.	46
43	(midwifery adj (service? or unit? or ward? or centre? or center?)).ti,ab.	352
44	((consultant led or obstetrician led) adj2 (service? or unit? or ward? or centre? or center?)).ti,ab.	132
45	((consultant or obstetrician) adj (service? or unit? or ward? or centre? or center?)).ti,ab.	159
46	(referral* adj5 (service? or unit? or ward? or centre? or center?)).ti,ab.	28869
47	("model of care" or care model* or "model of delivery" or delivery model* or "model of service" or service model*).ti,ab.	12723
48	18 or 19 or 20 or 21 or 22 or 23 or 24 or 25 or 26 or 27 or 28 or 30 or 31 or 32 or 33 or 34 or 35 or 36 or 37 or 38 or 39 or 40 or 41 or 42 or 43 or 44 or 45 or 46 or 47	1251197
49	17 and 48	15581

For peer review only

Meta-analysis of Observational Studies in Epidemiology (MOOSE) Checklist

The impact of obstetric unit closures, travel time and distance from women's home to obstetric services on maternal and neonatal outcomes: a systematic review

Criteria		Brief description of how the criteria were handled in the meta-analysis
**Reporting of background should include**		
√	Problem definition	Closure of small obstetric units (OUs) and centralisation of obstetric services in larger units has been proposed to increase levels of consultant obstetrician cover to improve safety and limit costs. This has potentially led to an increase in travel distance or time for women in labour from their home to the nearest obstetric unit.
√	Hypothesis statement	Increases in travel time/distance could potentially increase the risk of adverse birth outcomes.
√	Description of study outcomes	Maternal and neonatal outcomes.
√	Type of exposure or intervention used	The closure of obstetric units was compared to no closure of obstetric units in the same population.<br>A shorter travel distance or time to OUs was compared with a longer travel distance or time.
√	Type of study designs used	We included any quantitative study design with a comparison group.
√	Study population	Women and their babies.
**Reporting of search strategy should include**		
√	Qualifications of searchers	All authors contributed to developing the search strategy. The information specialist NR has been acknowledged.
√	Search strategy, including time period included in the synthesis and keywords	See Medline search strategy in Appendix 2.<br>Search time: 1990 to 2019.
√	Databases and registries searched	We searched: Embase, Medline, PsycINFO, Applied Social Science Index and Abstracts (ASSIA) and Cumulative Index to Nursing and Allied Health (CINAHL) databases. We also searched British Library EThOS, Open Grey and ProQuest Dissertations & Theses Global.
√	Search software used, name and version, including special features	OvidSP, We detailed the MeSH/ Emtree Headings and text words in Appendix 2.
√	Use of hand searching	Bibliographies of the included papers were hand searched for additional references.
√	List of citations located and those excluded, including justifications	Details of the literature search process are outlined in the PRISMA flow chart. The citation list of excluded articles is available from authors upon request.
√	Method of addressing articles published in languages other than English	We placed no restrictions on language. Studies published in languages other than English were translated into English and included in the review if they fulfilled the review inclusion criteria.

For peer review only

√	Method of handling abstracts and unpublished studies	We contacted a number of authors for full report of relevant unpublished studies.
√	Description of any contact with authors	We contacted authors of relevant articles for full report of their unpublished studies. NHS Trusts and Health Boards in the UK were also contacted where we had been able to identify an OU closure. For details of the authors who provided extra unpublished information, see the acknowledgments section.
**Reporting of methods should include**		
√	Description of relevance or appropriateness of studies assembled for assessing the hypothesis to be tested	Detailed inclusion and exclusion criteria are described in tables 1A, 2A and 3A.
√	Rationale for the selection and coding of data	A data extraction form was developed (available on request) to extract data related to characteristics of the study participants, time and distance measured, outcomes reported and quality assessment.
√	Assessment of confounding	When possible, we reported both crude and adjusted analyses for each outcome. However, data were adjusted for different confounding factors across the included studies.
√	Assessment of study quality, including blinding of quality assessors; stratification or regression on possible predictors of study results	We used a modified version of the Effective Practice and Organisation of Care (EPOC) and the risk of bias in non-randomised studies of interventions Newcastle-Ottawa scale (NOS) (for case-control studies). No meta-analysis was feasible therefore; we were unable to conduct any sensitivity analysis to compare results from only low risk of bias studies.
√	Assessment of heterogeneity	NA
√	Description of statistical methods in sufficient detail to be replicated	We found the included studies varied in their study design, categories of exposure, outcomes reported, whether adjusted or unadjusted results were presented and factors adjusted for. Due to this clinical and methodological variability quantitative pooling data was not feasible.
√	Provision of appropriate tables and graphics	We included PRISMA flow chart to show the method of studies identification.<br>Table 1A: Description of included studies - Obstetric unit closures<br>Table 1B: Risk of bias - Obstetric unit closure studies<br>Table 1C: Outcomes - Obstetric unit closure<br><br>Table 2A: Description of included studies - Travel distance<br>Table 2B: Risk of bias - Travel distance<br>Table 2C: Outcomes - Travel distance<br><br>Table 3A: Description of included studies - Travel time<br>Table 3B: Risk of bias - Travel time<br>Table 3C: Outcomes - Travel time
**Reporting of results should include**		

√	Graph summarizing individual study estimates and overall estimate	NA
√	Table giving descriptive information for each study included	Table 1A, 2A, 3A.
√	Results of sensitivity testing	NA
√	Indication of statistical uncertainty of findings	The 95% confidence interval (CI) is reported to estimate the precision of the ORs and RRs.
**Reporting of discussion should include**		
√	Quantitative assessment of bias	
√	Justification for exclusion	Reasons for exclusion were reported in PRISMA flow chart.
√	Assessment of quality of included studies	All presented in details in table 1B, 2B and 3B.
**Reporting of conclusions should include**		
√	Consideration of alternative explanations for observed results	Results were inconsistent across studies and no meta-analysis was feasible.
√	Generalization of the conclusions	We reported the fact that almost all of the included studies were from Europe.
√	Guidelines for future research	We highlighted the need to use routinely collected data. In addition to the need of applying, a standardized way of measuring travel time and distance from woman's place of residence to an OU.
√	Disclosure of funding source	This research is funded by the National Institute for Health Research (NIHR) Policy Research Programme, conducted through the Policy Research Unit in Maternal Health and Care, 108/0001.

For peer review only

BMJ Open

Title: The impact of obstetric unit closures, travel time and distance to obstetric services on maternal and neonatal outcomes in high-income countries: a systematic review

Journal:	*BMJ Open*
Manuscript ID	bmjopen-2020-036852.R1
Article Type:	Original research
Date Submitted by the Author:	18-Jul-2020
Complete List of Authors:	Malouf, Reem; University of Oxford, Policy Research Unit in Maternal Health and Care, National Perinatal Epidemiology Unit, Nuffield Department of Population Health<br>Tomlinson, Claire; University of Birmingham, Institute of Applied Health Research, College of Medical and Dental Sciences<br>Henderson, Jane; University of Oxford, Policy Research Unit in Maternal Health and Care, National Perinatal Epidemiology Unit, Nuffield Department of Population Health<br>Opondo, Charles; University of Oxford, Policy Research Unit in Maternal Health and Care, National Perinatal Epidemiology Unit, Nuffield Department of Population Health;<br>Brocklehurst, Peter; University of Birmingham, Institute of Applied Health Research, College of Medical and Dental Sciences<br>Alderdice, Fiona; University of Oxford, Policy Research Unit in Maternal Health and Care, National Perinatal Epidemiology Unit, Nuffield Department of Population Health<br>Phalguni, Angaja; University of Birmingham, Institute of Applied Health Research, College of Medical and Dental Sciences<br>Dretzke, Janine; University of Birmingham, Institute of Applied Health Research, College of Medical and Dental Sciences
**Primary Subject Heading**:	Health services research
Secondary Subject Heading:	Obstetrics and gynaecology, Health services research, Evidence based practice
Keywords:	EPIDEMIOLOGY, OBSTETRICS, PUBLIC HEALTH

SCHOLARONE™
Manuscripts

I, the Submitting Author has the right to grant and does grant on behalf of all authors of the Work (as defined in the below author licence), an exclusive licence and/or a non-exclusive licence for contributions from authors who are: i) UK Crown employees; ii) where BMJ has agreed a CC-BY licence shall apply, and/or iii) in accordance with the terms applicable for US Federal Government officers or employees acting as part of their official duties; on a worldwide, perpetual, irrevocable, royalty-free basis to BMJ Publishing Group Ltd ("BMJ") its licensees and where the relevant Journal is co-owned by BMJ to the co-owners of the Journal, to publish the Work in this journal and any other BMJ products and to exploit all rights, as set out in our licence.

The Submitting Author accepts and understands that any supply made under these terms is made by BMJ to the Submitting Author unless you are acting as an employee on behalf of your employer or a postgraduate student of an affiliated institution which is paying any applicable article publishing charge ("APC") for Open Access articles. Where the Submitting Author wishes to make the Work available on an Open Access basis (and intends to pay the relevant APC), the terms of reuse of such Open Access shall be governed by a Creative Commons licence – details of these licences and which Creative Commons licence will apply to this Work are set out in our licence referred to above.

Other than as permitted in any relevant BMJ Author's Self Archiving Policies, I confirm this Work has not been accepted for publication elsewhere, is not being considered for publication elsewhere and does not duplicate material already published. I confirm all authors consent to publication of this Work and authorise the granting of this licence.

For peer review only

Title: The impact of obstetric unit closures, travel time and distance to obstetric services on maternal and neonatal outcomes in high-income countries: a systematic review

Reem Malouf[1*], Claire Tomlinson[2], Jane Henderson[1], Charles Opondo[1], Peter Brocklehurst[1], Fiona Alderdice[1], Angaja Phalguni[2], Janine Dretzke[2]

[1]Policy Research Unit in Maternal Health and Care, National Perinatal Epidemiology Unit, Nuffield Department of Population Health, University of Oxford, Old Road Campus, Old Road, Oxford, UK

[2]Institute of Applied Health Research, College of Medical and Dental Sciences, University of Birmingham

*** Corresponding author**

reem.malouf@npeu.ox.ac.uk

For peer review only

Abstract

Objectives: To systematically review a) The effect of obstetric unit (OU) closures on maternal and neonatal outcomes; b) The association between travel distance/time to an OU and maternal and neonatal outcomes.

Design: Systematic review of any quantitative studies with a comparison group.

Data sources: Embase, MEDLINE, PsycINFO, Applied Social Science Index and Abstracts, CINAHL and grey literature were searched.

Methods: Eligible studies explored the impact of closure of an OU or the effect of travel distance/time on pre-specified maternal or neonatal outcomes. Only studies of women giving birth in high-income countries with universal health coverage of maternity services comparable to the UK were included. Identification of studies, extraction of data and risk of bias assessment were undertaken by at least two reviewers independently. The risk of bias checklist was based on the Cochrane Effective Practice and Organisation of Care (EPOC) criteria and the Newcastle-Ottawa scale (NOS). Heterogeneity across studies precluded meta-analysis and synthesis was narrative, with key findings tabulated.

Results: 31 studies met the inclusion criteria. There was some evidence to suggest an increase in babies born before arrival (BBA) following OU closures and/or associated with longer travel distances or time. This may be associated with an increased risk of perinatal or neonatal mortality, but this finding was not consistent across studies. Evidence on other maternal and neonatal outcomes was limited but did not suggest worse outcomes after closures or with longer travel times/distances. Interpretation of findings for some studies was hampered by concerns around how accurately exposures were measured, and/or a lack of adjustment for confounders or temporal changes.

For peer review only - http://bmjopen.bmj.com/site/about/guidelines.xhtml

Conclusion: It is not possible to conclude from this review whether OU closure, increased travel distances or times are associated with worse outcomes for the mother or the baby.

Prospero registration number: CRD42017078503.

Strengths and Limitations

- This review is the first to synthesise systematically the current evidence on the impact of closure of obstetric units and of travel distance and travel time to obstetric units on neonatal and maternal outcomes.
- Rigorous systematic review methodology was applied including a sensitive search strategy to ensure all relevant evidence was identified.
- Heterogeneity across included studies precluded any form of meta-analysis.
- A paucity of evidence on a number of outcomes, and methodological concerns for some studies limited conclusions that could be drawn.

For peer review only

For peer review only - http://bmjopen.bmj.com/site/about/guidelines.xhtml

For peer review only

Background

Closure of small obstetric units (OUs) and centralisation of obstetric services in larger units has been proposed to increase levels of consultant obstetrician cover to improve safety and limit costs. However, closure of OUs or conversion of OUs to midwifery-led units/community-based services potentially leads to an increase in travel distance or time for women in labour from their home to the nearest obstetric unit. Increases in travel time could potentially increase the risk of adverse birth outcomes.

Travel time and distance are widely used as measures to explore the geographical accessibility of health services (1). In a systematic review (2) the association between travelling further to healthcare facilities and having worse health outcomes was established, but the review did not include studies of maternity care. The impact of OU closure and increase in travel time/distance to the OU on perinatal and maternal outcomes have not been systematically assessed. One review (3) evaluating the effects of regionalisation of perinatal services has been published. This concluded that regionalisation programmes appeared to be correlated with improvements in perinatal outcomes but that the evidence was weak. A narrative review (4) included 10 studies that explored travel time and distance to and between maternity services and adverse birth outcomes to inform the consultation on maternity services in Wales. The review was limited to studies reported in English and there was no clear association between travel distance or time and adverse birth outcomes

Therefore, uncertainty remains about the association between OU closure, prolonged time or distance to OUs and adverse perinatal outcomes. Specifically, there is a rise in the risk of babies born before arrival (BBA, also referred to as unplanned out of hospital births). Being born before arrival is more common before term and has been reported to be associated with higher perinatal mortality (5). Conversely, Lasswellet et al. (6), found neonatal mortality was reduced when services were configured to ensure very preterm infants are born in a large maternity hospital with neonatal intensive care unit (Level III). In addition to mortality, Apgar scores (a standardised measure of the

For peer review only - http://bmjopen.bmj.com/site/about/guidelines.xhtml

physical condition of a newborn infant) and neonatal admission to intensive care provide an indication of perinatal infant health.

The impact on maternal outcomes is also unclear. There are concerns that low risk women who give birth in larger hospitals may experience more interventions, for example, increased frequency of caesarean section (CS) (7). Along with CS, evidence on maternal mortality and maternal birth complications such as post-partum haemorrhage (PPH) and maternal blood transfusion, was also sought in this review to identify the potential impact of OU closure on maternal outcomes.

In this review we aimed to systematically identify, critically appraise and synthesise the evidence relating to:

a) The effect of OU closures on maternal and neonatal outcomes (compared with the surrounding area or a comparable population).

b) The association between travel distance or time to an OU and maternal and neonatal outcomes.

Review method

The protocol was registered with Prospero (registration number CRD42017078503) and the Meta-Analyses and Systematic Reviews of Observational Studies MOOSE reporting guideline was followed (8).

Criteria for considering studies for this review

Types of studies

Any quantitative study design with a comparison group was eligible for inclusion. Studies were included from 1990 onwards. The year 1990 was chosen as a cut-off date because significant advances were made in neonatal care in the early 1990s, such as surfactant therapy, assisted ventilation, prophylactic infection control and antenatal steroid therapy, which impacted on the

For peer review only - http://bmjopen.bmj.com/site/about/guidelines.xhtml

delivery of maternity services (9). The quantitative components of mixed methods studies were also eligible. Studies were included if they:

- Explored the impact of closure of an OU on maternal or neonatal outcomes either in a before-and-after comparison (same population catchment area), or a geographical comparison of different areas (comparable populations)

And/or

- Compared maternal and neonatal outcomes after an OU closure and retention or creation of midwifery led units to replace the OU
- Explored the effect of travel time and/or distance on maternal and neonatal outcomes providing at least two travel times and/or distances from women's homes to the nearest OU
- Explored maternal and neonatal outcomes following maternal transfer from planned or unplanned home birth to the nearest maternity centre.

We included studies of women giving birth in high-income (OECD) countries with universal health coverage (UHC) of maternity services comparable to the UK. The list of OECD countries is shown in appendix 1. UHC is defined as health care that meets everyone's right to access high quality essential health services where and when they need them without financial difficulty (10).

Types of exposures

OU closure: the closure of an OU was compared to no closure of an OU for the same or comparable geographical catchment areas prior to the closure. For a study comparing different geographical areas affected by the closure of an obstetric unit, the least affected area was used as a control group. For the purpose of this review we used the definition of an OU used in the Birthplace Research programme in England (11), which defined an OU as "*a clinical location in which care is provided by a team, with obstetricians taking primary professional responsibility for women at high*

For peer review only - http://bmjopen.bmj.com/site/about/guidelines.xhtml

risk of complications during labour and birth. Midwives offer care to all women in an OU, whether or not they are considered at high or low risk, and take primary responsibility for women with straightforward pregnancies during labour and birth. Diagnostic and treatment medical services including obstetric, neonatal and anaesthetic care are available on site, 24 hours a day"(11)(P12).

Travel distance or time to the nearest OU: a shorter travel distance or time was compared with a longer travel distance or time. We used the definition of a shorter or a longer time or distance as defined by the included studies. When a study compared several different travel times or distances to the nearest OU, those with the shortest travel distance or time were used as the control group.

The following types of studies were excluded:

- Studies comparing maternal and or neonatal outcomes based on hospital size, level of neonatal intensive care, type of hospital or model of care (e.g. caseload midwifery care versus consultant care)
- Studies on regionalisation of neonatal care (number of centres with neonatal intensive care (NICUs))
- Studies where a proximity rather than the actual travel time or travel distance was given (e.g. rural vs urban, remote vs very remote areas)
- Studies which did not report at least one of the outcomes.

Review outcomes

The following outcomes were pre-defined in the study protocol:

Maternal outcomes

For peer review only - http://bmjopen.bmj.com/site/about/guidelines.xhtml

For peer review only

Maternal mortality (MM), caesarean section (CS) (overall, emergency or intrapartum), severe perineal trauma (including 3rd and 4th degree tears), postpartum haemorrhage (PPH), maternal admission to intensive care unit (ICU) and maternal blood transfusion.

Neonatal outcomes

Stillbirth (SB) (overall or intrapartum), neonatal mortality (NM), perinatal mortality (PM), infant mortality (IM), babies born before arrival (BBA), neonatal unit admission (NNU), Apgar score and hypoxic-ischemic encephalopathy (HIE).

Review methods

A comprehensive search strategy was developed in collaboration with an information specialist (NR). We searched Embase, Medline, PsycINFO, Applied Social Science Index and Abstracts (ASSIA) and Cumulative Index to Nursing and Allied Health (CINAHL) databases (from 1990 to February 2019). We also searched the Grey literature in the databanks of British Library EThOS, Open Grey and ProQuest Dissertations & Theses Global. NHS Trusts and Health Boards in the UK were also contacted where we had been able to identify an OU closure to request information about any evaluations that were conducted. The references of eligible studies and relevant reviews were checked to identify additional studies not retrieved by the search. Searches were based on index terms and text words relating to the population/setting (e.g. maternity service, pregnancy, neonatal) and exposures (e.g. travel/distance or closure/regionalisation). Due to the variable nature of terms and indexing used, the strategy was kept broad by using a range of alternate terms and not limiting by outcome. No language restriction was applied. A sample search strategy for MEDLINE is shown in appendix 2.

At least two reviewers (RM, CT, AP, FA, JH) independently screened the references for relevance against the review eligibility criteria using Eppi-reviewer software (version 4)(12). Full-text study screening was also performed by at least two reviewers (RM, CT, CO, JH, FA). Disagreements

For peer review only - http://bmjopen.bmj.com/site/about/guidelines.xhtml

regarding study eligibility were resolved through discussion and consensus within the review team. We contacted authors of relevant studies published as abstracts for further information. Data extraction and risk of bias assessment were undertaken by at least two reviewers (RM, CT, CO, JH, FA, JD). The risk of bias checklist was adapted from the Effective Practice and Organisation of Care (EPOC) (13) and the risk of bias in non-randomised studies of interventions Newcastle-Ottawa scale (NOS) (for case-control studies) (14). Risk of bias assessment included selection of study groups, measurement of exposure and outcomes, missing data and appropriateness of analysis (e.g. logistic regression analysis). For case-control studies, selection and comparability of cases and controls were also considered. The review team rated the quality of evidence for each domain in the tool as low, high or unclear risk of bias, or yes, no and unclear in meeting quality criteria.

Results were synthesised narratively and the key findings tabulated. The included studies varied in their study design, categories of exposure, outcomes reported, whether adjusted or unadjusted results were presented and factors adjusted for. This clinical and methodological heterogeneity across the included studies precluded any form of meta-analysis. Pre-specified sub-groups were risk-status of woman (low versus high), parity, gestational age, UK studies compared with non-UK studies, and planned vs unplanned CS; formal sub-group analyses were however not possible . Evidence regarding OU closure, travel distance and travel time is reported separately, and by outcome. We have highlighted where crude (unadjusted) odds ratios (cOR) and adjusted odds ratios (adj OR) have been reported.

Patient and Public Involvement

We involved our Parent, Patient, and Public Involvement (PPPI) Stakeholders Network, to explore which outcomes were important from a maternal perspective. The dissemination of findings to stakeholders will be through plain language summaries developed with members of our PPPI stakeholder network.

For peer review only - http://bmjopen.bmj.com/site/about/guidelines.xhtml

Search results

Searches of bibliographic databases and other sources from 1990 to February 2019 yielded 13,271 unique references (Figure 1). The eligibility of 295 full-text articles were assessed independently. 260 articles were excluded for various reasons, including: studies conducted in low/middle income countries, comparing different models or levels of maternity care, assessing women's transfer from primary to secondary maternity centres, or not providing quantifiable measures of travel time/distance (full list available from authors). Thirty-one studies, reported in 35 articles, met the review eligibility criteria (Figure 1). One study (5), included information on both OU closure and travel distance. Ten studies provided information on OU closures, seven studies compared different travel distances from women's homes to the nearest OU; and 15 studies compared different travel times from women's homes to the nearest OU.

Review results

Evidence from obstetric unit (OU) closures

A detailed description of the ten included OU closure studies is presented in Table 1A. Three studies were from the UK, with two reported as abstracts only (15, 16) and one an unpublished data series from East Lancashire Hospitals National Health Services (NHS) Trust, UK (17). There appeared to be overlap between populations reported in two studies (East Lancashire Hospitals NHS Trust) (15, 17). Three studies were from Scandinavia (7, 18, 19), three from Canada (20-22) and one from France (5).

Seven studies compared adverse birth outcomes before and after centralisation of services, which included closure of varying numbers of OUs. All three studies from the UK (15-17) examined the impact of the amalgamation of two OUs. Four studies were published after 2014 (15, 16, 18, 21); the earliest was from 1990 (22). Three studies included all births (18, 19, 22); the other studies varied in

For peer review only - http://bmjopen.bmj.com/site/about/guidelines.xhtml

their eligibility criteria, e.g. restricting the analysis to singletons, live births, various gestational ages and birthweight, hospital births or location. Reporting of eligibility criteria and participant characteristics across studies was inconsistent (Table 1A).

Risk of bias assessment

Risks of bias related to a lack of reporting of whether changes over time (other than closure/reconfiguration) could have influenced the findings, with only two (18, 21) of ten studies reporting that temporal variation was adjusted for in the analysis (Table 1B). Further, five out of ten studies either did not adjust results for potential confounding factors or provided insufficient information to know whether this was undertaken. Five out of ten studies did not provide sufficient information to gauge the completeness of data. Half of the studies reported and used appropriate data analysis methods. Other potential sources of bias (e.g. relating to selection, exposure and outcome) were less of a concern due to the use of routinely collected registry data before and after the closure and the objective nature of most outcomes.

Findings

A summary of maternal and neonatal outcomes is presented in Table 1C.

Maternal outcomes

Maternal mortality (MM)

In the two studies that reported maternal mortality (16, 21), the number of deaths (<5) was too low to allow comparisons between the pre- and post-closure groups .

Caesarean section (CS) (overall or intrapartum)

For peer review only - http://bmjopen.bmj.com/site/about/guidelines.xhtml

Total CS rates were reported in four studies (7, 15, 21, 22). One UK study(15) reported a decline in CS rates following the amalgamation of two units from 26.1% to 21.5%.

A Norwegian study (7), reported an increase in CS rates from 13.1% to 16.4% following OU closure, (cOR=1.31, 95%: 1.27, 1.35) as did two Canadian studies (22)cOR= 1.13, 95%CI: 1.09, 1.18) and (cOR= 1.10, 95%CI: 1.01, 1.19)(21). No adjusted results were reported.

Emergency caesarean section (CS)

Emergency CS rates were reported in one UK study (16), which found no difference before/after the amalgamation of two OUs (cOR=0.95, 95%CI: 0.86, 1.05).

Severe perineal trauma (3rd or 4th degree tear).

Two studies (16, 21) reported this outcome and found no statistically significant difference between the before/after closure groups. The incidence of the outcome in both studies was low (<3%).

Postpartum haemorrhage – No studies reported this outcome.

Maternal admission to intensive care unit (ICU)

Two studies (16, 21) found no significant difference before/after the amalgamation of two OUs on the number of women requiring admission to ICU (cOR= 0.80, 95%CI: 0.44, 1.46) (16). The numbers in one study (21) were too small (<5) to allow a comparison.

Maternal blood transfusion

One study (21) found no significant differences before/after OU closure (cOR=0.82, 95%CI: 0.55, 1.21). The incidence of the outcome was low (<1% of women).

Neonatal outcomes

Stillbirth (SB) (overall or intrapartum)

For peer review only - http://bmjopen.bmj.com/site/about/guidelines.xhtml

Three studies examined the impact of OU closure on stillbirth. One unpublished UK study (17) showed a statistically significant reduction in stillbirths over the period after the amalgamation of two units (cOR=0.61, 95%CI: 0.47, 0.78). Similar findings were seen in one study from Canada (23) during post closure years (cOR=0.80, 95%CI: 0.67, 0.96). A third study from the UK (16) found no difference in stillbirth rates after OU closure.

Neonatal mortality (NM)

Three studies reported this outcome. Two studies from the (16, 17), showed no statistically significant difference in the rate of NM in the years after OU closure (cOR= 1.33, 95%CI: 0.81, 2.17; cOR= 0.80, 95%CI: 0.29, 2.26). A study from Norway (18) also reported no difference (no OR presented).

Perinatal mortality (PM)

Two studies reported this outcome. In a study from Norway (7), PM was significantly lower following OU closure (cOR= 0.76, 95%CI: 0.58, 0.98). A Canadian study (23) also reported a significant reduction in PM after OU closure (cOR= 0.75, 95%CI: 0.64, 0.87).

Infant mortality (IM)

One study (18) reported this outcome, IM rates were “not statistically elevated” after the closure of thirteen hospitals in Norway.

Born before arrival (BBA)

Six studies reported this outcome, with four suggesting a statistically significant increase in BBA following OU closure. Data from East Lancashire Hospitals NHS Trust (17), showed the BBA rate almost doubled over the 10 year period (cOR= 1.88, 95%CI: 1.25, 2.82). Studies from Norway (7) and Finland (19) also found that the BBA rate increased over a similar period (cOR=1.8, 95%CI: 1.6, 1.9 and cOR=4.14, 95%CI: 3.16, 5.41 respectively). A Canadian study (21) found that the BBA rate trebled over a 16 year period (cOR= 3.47, 95%CI: 2.31, 5.20). One UK study (16) found no statistically

For peer review only - http://bmjopen.bmj.com/site/about/guidelines.xhtml

significant change (cOR= 1.28, 95%CI: 0.63, 2.60) and in one French study (5), there was weak evidence of a small reduction in the adjusted risk of BBA in communities affected by OU closure (adjOR = 0.91, 95%CI: 0.84, 1.00).

Neonatal Unit admission (NNU)

One Canadian study (21) suggested a significant reduction in NNU admission following OU closure (cOR= 0.39, 95%CI: 0.25, 0.60).

Apgar score

One Canadian study (21) found no statistically difference in 5 minute Apgar score of less than 7 before and after OU closure (cOR= 1.13, 95%CI: 0.82, 1.55)

Hypoxic-Ischemic Encephalopathy (HIE) – No studies reported this outcome.

Evidence from travel distance studies

Description of included studies

Seven studies described the effect of travel distance to the nearest OU on maternal and neonatal outcomes (Table 2A). All were published in full between 1991 and 2015. The earliest study (24) was conducted in the UK, three more recent studies were conducted in France (5, 25, 26), and one each from Norway (27), Finland (28), and Canada (29). Four were retrospective population-based cohort studies, and three were case-control studies. The eligibility criteria varied across studies. Pasquier et al. (25), included a group with special needs in the form of babies with congenital malformations. Only singleton live births were included in two studies (5, 29).

Travel distance was estimated using geographical mapping software in all studies. However, only three studies (5, 24, 25, 28) measured the actual distance from women's homes to the nearest OU. In two studies (26, 27, 29) a central geographical point for the postcode or municipality was used to

For peer review only

For peer review only - http://bmjopen.bmj.com/site/about/guidelines.xhtml

estimate distances and in one study the distance was self-reported (27) Additionally, the studies differed regarding their distance categories, which ranged from 2km to 150km (Table 2A).

Risk of bias assessment

The main risk of bias concerns related to the measurement of exposure, as three studies (24, 26, 27, 29) did not calculate the distance from the woman's home but used a central point instead or self-reported distance (Table 2B). Another study (24), measured distance between women's homes and hospital using a straight line. Further risk of bias related to a lack of comparability between study groups in the three case-control studies (24, 27, 28) , a lack of adjustment for confounders in two studies and missing data in two studies (27, 28). There were no risk of bias concerns relating to sample selection in the cohort studies or outcome measurement.

Findings

Maternal outcomes

Maternal mortality (MM)

In one case-control study from Finland (28), no maternal deaths were reported in either group (Table 2C).

Caesarean section (CS) (overall, or intrapartum)

One study from Canada (29) found no statistically significant differences in CS rates with increasing distance (<50k, 50-150k, >150k) based on both crude and adjusted results.

Emergency Caesarean section (CS)

The same study from Canada (29) reported no significant difference in emergency CS rates between women living at different distances from an OU based on crude ORs.

For peer review only - http://bmjopen.bmj.com/site/about/guidelines.xhtml

For peer review only

No studies reported on *severe perineal trauma, postpartum haemorrhage, maternal admission to intensive care unit (ICU) or maternal blood transfusion.*

Neonatal outcomes

Stillbirth (SB) (overall or intrapartum)

Two cohort studies (26, 29) reported this outcome. A Canadian study (29) included births to women aged over 35 years who lived <50km, 50-150km and >150 km from the OU. Stillbirth rates did not change by distance category in the adjusted analysis. A French study (26) reported stillbirth rates at different distances (<5 km, 5-15, 15-30, 30-44, 45+ km) from mother's municipality of residence to the closest OU. The crude odd ratios showed women living at 30-44, 15-29 and 5-14km from an OU had a statistically significantly lower rate of stillbirth compared to women living <5 km from an OU (5-14 km vs <5 km, cOR = 0.87, 95%CI: 0.85, 0.90; 15-29 km vs <5 km; cOR = 0.85, 95%CI: 0.82, 0.88; 30-44 km vs <5 km, cOR= 0.85, 95%CI: 0.81, 0.90). The findings still hold for the adjusted analysis (limited data reported). However, neither the crude nor the adjusted analysis showed a significant difference in risk of stillbirth for individuals resident 45+ km from an OU compared with <5km.

Neonatal mortality (NM)

Two French cohort studies (25, 26) reported this outcome. One study (25) examined the distance from women's homes to the nearest OU with neonatal surgical facilities for 706 foetuses with severe malformations. Analyses adjusted for malformation type, number of malformations, amniotic fluid anomaly, previous anomaly in the family and parity showed no association between NM and distance (<11 km vs 11-50 km, adj OR = 0.89, 95%CI: 0.34, 2.88; <11 km vs > 50 km, adj OR= 1.37, 95%CI: 0.49, 3.86). The other study (26) included all births and found that NM rates were significantly higher for women living <5 km compared to 5 -44 km away from an OU (26) (5-14 km vs < 5 km, cOR= 0.78, 95%CI: 0.75, 0.81; 15-29 km vs < 5 km cOR = 0.81, 95%CI: 0.78, 0.85; 30-44 km

For peer review only - http://bmjopen.bmj.com/site/about/guidelines.xhtml

vs < 5 km, cOR= 0.78, 95%CI: 0.72, 0.84; ≥ 45 km vs < 5 km, cOR= 0.82, 95%CI: 0.70, 0.96). In this latter study, the NM of babies born before arrival was also explored. For the BBA group, there was a statistically significant increase in the risk of NM when women had to travel 45km or more to an OU in comparison to <5 km (≥ 45 km vs < 5 km, cOR= 2.45, 95%CI: 1.14, 5.25).

Perinatal mortality (PM)

A study from Canada (29) reported that PM risk increased with travel distance in an adjusted model (<50 km v 50-150 km adjOR=1.53, 95%CI: 1.1-2.12; <50 km >150km adjOR=3.06, 95%CI: 2.20,4.24).

Infant mortality (IM) - No studies reported this outcome.

Born before arrival (BBA)

Three case control studies (24, 27, 28), and one cohort study (5) reported this outcome. All four studies reported a significant increase in BBA rate with longer travel distance, although only two reported adjusted analyses (5, 28). In the UK study (24), the risk of BBA increased 12 fold for women living >7 km from the OU compared to women living <2 km away (cOR = 12.5, 95%CI: 4.02, 36.01). The risk of BBA increased significantly for women living >13 km from an OU in a Norwegian study (27) (cOR= 1.81, 95%CI: 1.03, 3.18). The Finnish study (28) reported a fivefold increased risk of BBA for women living >35 km from the OU compared to <35 km (adj OR 5.02, 95%CI: 1.80, 14.04).

In France (5), the rate of BBA significantly increased with longer distances and it tripled for all women living 45+ km from the OU compared to women living <5km away (cOR: 3.77, 95%CI: 3.23, 4.39). The association persisted in an adjusted analysis which included women of parity three or higher and living 45+ km from the OU, who had a six fold increased risk of BBA compared to women living <5 km away and of parity one or two (adj OR= 6.49, 95%CI: 4.92, 8.48).

Neonatal unit admission (NNU)

A study from Canada (29) reported an increase in NNU admission for births to women living >150 km from an OU compared to those living <50 km away (6.8% vs 4.8%).

For peer review only - http://bmjopen.bmj.com/site/about/guidelines.xhtml

For peer review only

Apgar score – No studies reported this outcome.

Hypoxic-Ischemic Encephalopathy (HIE) – No studies reported this outcome.

Evidence from travel time studies

Description of included studies

Fifteen studies explored the impact of travel time from a woman's home to an OU (see Table 3A). Two studies (one reported as an abstract only) were conducted in the UK (30-32), three studies in France (33-35), three studies (reported in five articles) in the Netherlands (36-40), one study reported in two articles from Norway (41, 42), five studies in Canada (43-47) and one study in Japan (48).

Eleven studies were of a retrospective cohort design, one was a prospective cohort study (40), one was a before-and-after design (48) and two were case-control studies (34, 35). All the studies clearly stated the eligibility criteria. Only singleton births were included in five studies (31-33, 36-39, 43). One study (40) specifically enrolled women with postnatal haemorrhage after home birth, and one study (43) focussed on planned home birth regardless of the actual place of birth.

The studies were heterogeneous in their travel time intervals. With the exception of one study in Canada (43), longer time cut-off points were examined in studies from Norway, Japan and Canada compared to studies in other countries (all European). Travel duration was estimated using geographical mapping software in all studies. However, most studies estimated travel duration to and from central points within areas rather than actual addresses.

Risk of bias assessment

Quality assessment and supported explanations for each of the risk of bias domains are presented in Table 3B. With the exception of Stolp et al. (40), sample selection and measurement of outcomes

For peer review only - http://bmjopen.bmj.com/site/about/guidelines.xhtml

were considered to be at low risk of bias across all studies as such data were obtained from national databases and birth registries. The two case- control studies were appropriately selected and defined, however the case and control groups were not comparable in both studies (e.g. difference in antenatal care attendance and socio-demographics). Eight studies (30, 35-43), were considered at low risk of exposure measurement bias, as the women's actual place of residence was used to estimate travel time to nearest OU. The risk of attrition bias was low for the majority of the included studies. Similarly, analyses and adjustment for potential confounders were found to be appropriate in the majority of studies.

Findings

Maternal outcomes

Maternal mortality - no studies reported this outcome

Caesarean section (overall, or intrapartum)

Five Canadian studies (43-47) reported CS rates (Table 3C). Across three studies (44-46), crude ORs for CS rates were higher among women who lived closer to OUs with CS rates highest for women living less than one hour away compared with other categories (1-2 hours, 2-4 hours and > 4 hours). One study (47) included women who had a midwife involved in their care, and found no significant differences in CS rates for women living 1-2 hours and more than 2 hours away compared to within one hour of an OU (1-2 vs < 1 hr, cOR= 1.23, 95%CI: 0.80, 1.91 and>2 hrs vs < 1 hr, cOR=1.11, 95%CI: 0.71, 1.72). A further study (43) showed also a higher CS rate among women who planned a home birth and lived less than half an hour away from OU services (>30 mins vs ≤ 30 mins,(cOR=0.74, 95%CI: 0.59, 0.92).

Emergency Caesarean section

Shorter travel time to an OU was associated with a statistically significant higher emergency CS rate in one Canadian study (46) (> 1hr vs < 1 hr, cOR= 0.80, 95%CI: 0.75, 0.86).

For peer review only - http://bmjopen.bmj.com/site/about/guidelines.xhtml

Severe perineal trauma – No studies reported this outcome.

Postpartum haemorrhage (PPH)

One Canadian study found the risk of PPH was significantly higher for women who lived more than one hour away from obstetric services compared to women who lived less than one hour away (45) (> 1hr vs < 1 hr, cOR=1.27, 95%CI: 1.13, 1.43).

Maternal admission to intensive care unit

One study from the Netherlands (40) involved women who had a PPH after midwifery-supervised home births and examined adverse maternal outcomes associated with travel time longer than 45 minutes to hospital. No difference was found in the number of women admitted to ICU who travelled more than 45 minutes compared with < 45 travel time to hospital, but the numbers of events were low.

Maternal blood transfusion

One study from the Netherlands (40) found no significant difference in the median number of units of blood transfused to women who travelled more than 45 minutes to an OU compared to <45 minutes travel time.

Neonatal outcomes:

Stillbirth SB (overall or intrapartum)

Three studies examined the association between increasing travel time and SB, one study each from the UK (31, 32), France (33) and Canada (45).

In the UK study (31, 32), there was no association between travel time and SB when analysing all women (adjOR = 1.13, 95%CI: 0.98, 1.30). However subgroup analyses showed a significant increase in the risk of SB with every 15 minute increase in travel time to the OU for term pregnancies

For peer review only - http://bmjopen.bmj.com/site/about/guidelines.xhtml

(adjOR=1.36, 95%CI: 1.17, 1.59) and for nulliparous women (adjOR=1.21, 95%CI: 1.02,1.44). The other two studies (33, 45) found no significant increase in the incidence of SB with increasing travel time .

Neonatal mortality (NM)

Five studies examined the association between travel time and NM, two from the UK (30-32), one from the Netherland (36-38), one from Canada (45) and one from Japan (48).

The adjusted analysis in one UK study(30) showed no statistically significant association between NM and travel time. The adjusted analyses in the other UK study (32) showed a significant increase in early and late NM, with every 15 minutes increase in travel time (adjOR= 1.13, 95%CI: 1.07, 1.20) and (adjOR= 1.15, 95%CI: 1.05, 1.26) respectively. Subgroup analysis for nulliparous women showed a statistically significant increased risk of early NM associated with every 15 min increase in travel time from home to the OU (adjOR= 1.15, 95%CI: 1.06, 1.25). For term births, late (but not early) NM increased significantly with every 15 mins increase travel time from home to the OU (adjOR= 1.34, 95%CI: 1.13, 1.59).

In one study from the Netherlands (36), a travel time of 20 minutes or more was associated with a significant increase in the combined intrapartum, early and late NM (36-38) (≥20 mins vs <20 mins, adjOR= 1.23, 95%CI: 1.07, 1.41). No neonatal mortality events were reported in the study from Canada (45). The study from Japan (48) reported that following a median reduction in travel time from 67 minutes in 2002 to 39 minutes in 2006 that there was a decrease in NM rate from 1.67 to 1.28, however no further analyses were presented.

Perinatal mortality (PM)

For peer review only - http://bmjopen.bmj.com/site/about/guidelines.xhtml

Seven studies examined PM, one from France (33), one from the Netherlands (39) and five from Canada (43-47). The French study (33) found no significant association between increasing travel time to the nearest OU and PM based on unadjusted data. However, in the Dutch study a longer travel time (20 mins or more) was significantly associated with higher PM (39) (>=20 mins vs < 20 mins, adjOR= 1.66, 95%CI: 1.59, 1.74).

The Canadian studies also reported longer travel times to OUs being associated with an elevated risk of PM. A significant increase in PM was reported in women living more than four hours away from OUs compared to women living less than one hour (> 4hrs vs < 1 hr adjOR= 3.17, 95%CI: 1.45, 6.95) (44). However, findings from the same study suggested no significant increase for women living 1-2 hours and 2 to 4 hours from an OU compared to those living less than one hour from services. Similarly, the PM risk significantly increased in women who lived > 1 hour from OUs in a further Canadian study (45), (cOR=1.54, 95%CI: 1.09, 2.17). When this was divided into different Canadian provinces (46), the rates of PM were highest in communities living more than 4 hrs from an OU in comparison to less than one hour in British Colombia only (adjOR=2.84, 95%CI: 2.84,5.10). Stoll et al. (47), found that in women who received midwifery care only, PM was not statistically significantly different for women living more than two hours away from an OU compared to women living less than one hour from an OU based on an unadjusted analysis (cOR= 3.57, 95%CI: 0.81,15.76). In Darling et al. (43), the PM rates were not statistically significantly different for women with a planned home birth and more than 30 minute drive from hospital (adjRR=2.2, 95%CI: 0.67,7.43).

Infant Mortality (IM)

One Canadian study (44) reported no significant difference in IM rates for women living less than one hour travel time to OU compared with more than one hour travel time to OU (cOR= 1.51, 95%CI: 0.86, 2.63).

For peer review only - http://bmjopen.bmj.com/site/about/guidelines.xhtml

Born before arrival (BBA)

Six studies reported this outcome, four cohort studies (33, 42, 44, 45), and two case-control studies (34, 35). Five of the six studies found some association between travel time and BBA, four based on adjusted analyses.

There were three studies conducted in France (33-35). Combier et al. (33), reported that a travel time greater than 15 minutes was significantly associated with an increased risk of BBA (16-30 mins vs < 16 mins, adjOR= 1.73, 95%CI: 1.23, 2.46); (31-45 mins vs < 16 mins, adjOR= 1.64, 95%CI: 1.06, 2.54) (33). In a case control study (35) the BBA rate increased six fold when the travel time increased to more than 45 minutes from home to the OU compared to women who travelled less than 15 minutes (> 45 mins vs > 15 mins, adjOR= 6.18 95%CI: 1.33, 28.65). However, in the other case control study the risk of BBA was not significantly increased in women who travelled for greater than 20 minutes (34).

In a study from Norway (41, 42) the risk of BBA increased significantly with longer travel time to the nearest OU from home. Women who travelled more than two hours had an eight fold increased risk of BBA compared to women who lived within one hour of the nearest OU (>2hrs vs <1 hr, cOR= 8.21, 95%CI: 7.19, 9.37) (42).

The studies from Canada (44, 45) found a significant increase in BBA in women living in communities greater than one hour travel time from an OU compared to those living less than one hour away. In Grzybowski et al. (44), women who lived one to two hours from an OU had the highest risk of BBA compared to less than one hour (adjOR= 6.41, 95%CI: 3.69, 11.28) and women who lived greater than four hours away also had increased risk compared to those living less than one hour away (adjOR: 3.63, 95%CI: 1.40,9.40); however there was no difference between those who lived 2-4 hours from an OU and those living less than one hour away (adjOR=0.92, 95%CI: 0.22, 3.88). Gryzbowski et al. (45), found a five-fold increase in BBA in women who lived more than an hour

For peer review only - http://bmjopen.bmj.com/site/about/guidelines.xhtml

away from an OU in comparison to women who lived less than an hour away (cOR=5.06, 95%CI: 3.82,6.70).

Neonatal unit admission (NNU)

Three studies from Canada reported on NNU admission (43-45). The two studies from British Columbia (44, 45) reported NNU depending on whether the admission was for Level 2 care (High Dependency) or Level 3 care (Intensive Care). Findings from one of these studies (44), showed NNU level 2 admission increased significantly in babies born to women living more than one hour away from an OU compared to less than one hour (adjOR = 2.20, 95%CI: 1.59, 3.05). For those living 2-4 hours away, Level 2 admissions were significantly lower compared with those living less than 1 hour away (adjOR=0.31, 95%CI: 0.14,0.65). For those living more than 4 hours away there appeared to be no increase in NNU level 2 admission. For level 3 NNU admission, a significantly increased risk was found for the 1-2 hour category (1-2 hrs vs < 1 hr, cOR= 2.72, 95%CI: 1.46, 5.09). For the other two categories, 2-4 and >4 hours, neither crude nor adjusted analyses showed any significant difference. The number of women in each group decreased with increasing time from an OU. In Grzybowski et al. (45), there was no increased risk of admission to NNU level 2 in babies born to women living more than one hour from an OU compared to less than one hour, however admission to NNU level 3 was significantly higher (cOR = 1.50, 95%CI: 1.01, 2.23). The third Canadian study from Ontario (43), showed a lower relative risk of NNU admission for planned home births with a travel time greater than 30 minutes when compared to less than 30 minutes (adjRR = 0.6, 95% CI: 0.44, 0.81).

Apgar score

Two studies reported on Apgar Score; one from Canada and one from the Netherlands (38, 43). In the Canadian study (43) no significant difference was found for Apgar score <7at five minutes

For peer review only - http://bmjopen.bmj.com/site/about/guidelines.xhtml

between women who planned home birth and lived more or less than 30 minutes away from an OU, either for nulliparous or multiparous sub-groups (adjRR =1 .02, 95%CI: 0.95, 1.10).

The study from the Netherlands (38) used a composite outcome of mortality and/or Apgar < 4 at 5 minutes and/or transfer to NNU, and showed a small but significant increase in this outcome in women whose travel time to an OU exceeded 15 minutes (15-20 mins vs <15 mins adjOR=1.11 95%CI: 1.02, 1.21 and >=20 mins vs <15 mins, adjOR= 1.27, 95%CI: 1.17, 1.38).

Hypoxic-Ischemic Encephalopathy (HIE) – No studies reported this outcome.

Discussion

This review describes studies which have explored the associations between OU closure, distance or travel time to an OU, and maternal and neonatal outcomes. The included studies were conducted in the UK, France, the Netherlands, Norway, Canada and Japan. Many studies were from parts of the world where service configuration varied and the study populations were sometimes dispersed over a large geographical area. The included studies differed in their design, geographical boundaries, outcomes measures used, and included a wide range of travel time/ distance thresholds used. In addition, although many studies reported that potential confounders were adjusted for in their analyses, many of the outcomes of interest for this review were crude measures of effect without adjustment. Therefore, comparing these studies with each other was a challenge.

All of these studies were brought together to explore whether women who had to travel longer and further to their planned OU were at increased risk of adverse outcomes. There was one reasonably consistent finding which was that there appeared to be an increased risk of BBA the longer it took to reach the OU. This may have been associated with an increased risk for the baby with a suggestion of an increased risk of perinatal or neonatal mortality in some studies, however, this effect was not consistent across all the studies. There was also an increase in CS rates following closure of an OU

For peer review only - http://bmjopen.bmj.com/site/about/guidelines.xhtml

and with shorter travel distance and time, however it is unclear if the difference was related to the exposure or unmeasured differences in CS rates.

Strengths and limitations of the review

This work is the first to synthesise systematically the current evidence relevant to obstetric unit closure and the impact of travel time and travel distance on maternal and neonatal outcomes. Rigorous systematic review methodology was applied, including a sensitive search strategy to identify all the relevant literature, and thorough assessment of potential risks of bias. All screening, data extraction and risk of bias assessment were performed independently by at least two reviewers.

The process of selecting studies for inclusion was challenging due to a lack of reporting of some details, for example, it was not always clear which level of maternity services the study referred to, in others, findings related to the impact of travel time and distance were not always presented despite this being described as a study objective.

Interpretation of findings

It is difficult to conclude from this review whether reconfiguration of maternity services, with closure of OUs, resulting in increased travel distances and times for women is unequivocally associated with worse outcomes for the mother or the baby. Assessing the impact of OU closure and prolonged travel time and distance is not straightforward; to isolate the impact of the closure and travel time and distance on maternal and neonatal outcomes we need to fully understand the models of maternity care, transport services, landscape characteristics and places of birth available to women in that specific geographical area. Understanding how services are delivered to women is vital when assessing the impact of travel distance and time as services may be adapted to meet the challenges for women living in remote areas , for example by transferring women antenatally a few weeks

For peer review only - http://bmjopen.bmj.com/site/about/guidelines.xhtml

For peer review only

before birth. Some studies found an increase in CS rates with shorter distance/travel time. Attributing this solely to closure or reconfiguration of services is problematic as simple analytical comparisons of rates before and after changes do not account for underlying time trends. Future studies might want to consider an interrupted time series design as a more appropriate method.

There remains an urgent need to evaluate the impact of changing maternity service provision. The imperative to close and consolidate OUs into larger units is based on a belief that this will improve safety for both mother and baby. If increasing travel times and distances increases risks to mothers and babies, then the postulated benefits of larger OUs could be offset by the harms of the reconfiguration.

Waiting for closure of OUs to prospectively evaluate the impact on the surrounding maternity population will always be challenging. However, exploring the existing impact of distance and travel time from home to an OU may be a reasonable approach to explore what the impact of reconfiguration may be for a proportion of the women in the area served by the OU which would have these parameters increased by closure of one of more local OU(s). Such a study would need to be large to explore the impact of travel time and distance on substantive harms such as mortality for the baby, so will almost certainly need to use routinely collected data to obtain large numbers. Such studies will also need to include vigorous evaluation of confounders, such as maternal characteristics, socioeconomic status and maternal medical history, which are known to influence birth outcomes; controlling for these factors is vital to determine the OU closure impacts. These studies should also collect data at multiple time points after the closure and apply statistical analysis which considers time-varying relationships and the outcomes.

Measurement of travel time and distance from the woman's place of residence to an OU would also need more sophisticated approaches than previously used in many studies; for example the use of web-based route planners and adjustment for travel conditions rather than using straight line distances or relying on self-reports.

For peer review only - http://bmjopen.bmj.com/site/about/guidelines.xhtml

For peer review only

Many study designs assume that travel time and distance have a constant effect on outcomes. If local OUs are far away, it is possible that women will modify their behaviour in relation to when they set off for their OU in labour, if they know they have an hour's journey compared with a 20 minute journey. The extent to which this will mitigate the effects of longer travel times would not be seen in a study looking at existing travel times and distances.

Conclusion

Given the substantial variation across studies we were unable to draw firm conclusions regarding the association between OU closure, travel distance or time to obstetric services and maternal and neonatal outcomes. There appears to be a consistent association with BBA with increasing distance and travel time to an OU and a suggestion of increasing risk to the baby. However, few studies have rigorously controlled for potential confounders.

Funding source: This research is funded by the National Institute for Health Research (NIHR) Policy Research Programme, conducted through the Policy Research Unit in Maternal Health and Care, 108/0001. The views expressed are those of the author(s) and not necessarily those of the NIHR or the Department of Health and Social Care.

Competing Interests: None of the authors has any competing interest.

Acknowledgment: Our thanks to Pam White for contacting NHS Trusts and obtaining papers, Nia Roberts for development of the search strategy and Mark Willett and Fiona Mackie for providing unpublished data.

Authors' contributions

PB, FA, RM and JD conceived the research. All authors developed the protocol and RM developed the search strategy. RM, CT, AP, FA and JH screened the search results and full papers. RM, CT, JH, FA and CO assessed the quality of included papers, extracted the data and synthesised the results. RM and FA drafted the manuscript and all authors agreed the final manuscript.

For peer review only - http://bmjopen.bmj.com/site/about/guidelines.xhtml

Data sharing statement

All the data included in this systematic review are in the public domain.

For peer review only

For peer review only - http://bmjopen.bmj.com/site/about/guidelines.xhtml

Tables:

Table 1A: Description of included studies – Obstetric unit closures

Author, year, Country	Study design & setting	Study objectives	Study period	Eligibility criteria	Participant characteristics	Description of exposure (change over time)	Services context information	Review Outcomes	
								Perinatal	Maternal
**UK STUDIES**									
Fleming/East Lancashire study									
Fleming 2013 (15), UK (abstract)	Retrospective analysis pre- and post-service reconfiguration<br><br>East Lancashire maternity services	To examine the effect of major service reconfiguration on CS rates	Time of reconfiguration: Nov 2010<br>Time of analysis: Jan-Jun 2010 vs Jan-Jun 2012	NR	N: NR<br><br>Characteristics: NR	Service reconfiguration<br>Pre-change: 2 OUs<br>Post-change: 1 OU + 3 MW-led units	Universal state provision of maternity care. Approx 7000 births /yr at the new unit.	NR	CS
East Lancashire Hospitals NHS Trust 2017 (17), UK (unpublished-data	Retrospective population-based analysis of routinely collected data of service reconfiguration	To review outcomes after centralisation of services on the Burnley general hospital site	Time of reconfiguration: Nov 2010<br>Time of analysis: 2009-2017	NR	2009-2017 N=53,870 births (2010 data excluded)<br><br>Characteristics: NR	Amalgamation of 2 obstetric units<br>Pre-change: 2 OUs<br>Post-change:	Universal state provision of maternity care. Changes driven by pressure on	BBA; SB; NM	NR

For peer review only - http://bmjopen.bmj.com/site/about/guidelines.xhtml

	East Lancashire, Blackburn and Burnley					1 OU plus 2 birth centres	staff rotas, European Working Time Directive, and desire to maintain high quality service. 6000-7000 births/yr at new unit.		
Mackie 2014 (16), UK (abstract & (unpublished data)	Retrospective analysis pre- and post-service reconfiguration Pennine Acute Trust (PAT): Royal Oldham Hospital (ROH), North Manchester General Hospital (NMGH) and Fairfield General Hospital (FGH)	To assess the effect of the amalgamation of 2 OUs to form a "super-centre" with increased consultant labour ward cover	Time of OUs amalgamation 2011 Time of analysis: pre-changes 2010 - 2011 vs. post changes 2011 - 2013	NR	Pre-amalgamation N=5422 Post-amalgamation N=5046<br><br>Characteristics : NR	Service reconfiguration<br><br>Pre-change: 2 OUs Post-change: 1 OU	Universal state provision of maternity care. Approx 5000 births /yr at the new unit.	SB; NM; BBA	Maternal mortality; Emergency CS; 3rd& 4th degree perineal tea; Maternal transfer to ICU
**OTHER EUROPEAN STUDIES**									
Blondel* 2011 (5), France	Retrospective population-based analysis of routinely	To report on BBA incidence in relation to distance from OU and the closure	Time of OU closure: 2003 and 2006 Time of analysis:	*Included:* Singleton births *Excluded:*	N=1,349,751 births; OoH n=5740 *N Births 1,349, 751*	Closure of maternity unit Pre-change: Number of OUs NR	Centralisation of births in larger units due to safety concerns,	BBA	NR

For peer review only - http://bmjopen.bmj.com/site/about/guidelines.xhtml

	collected data, OU closure<br><br>Across France	impact on different socio-demographic groups	2005-2006	Municipalities if >8% missing data, or high OoH rates. Departments excl. if >20% births already excl.	Age (yrs) (n):<br><20 - 26,152<br>20-24 - 188,350<br>25-29 - 427,462<br>30-34 - 442,089<br>35-39 - 213,534<br>40+ - 52,164<br><br>Nullip (n) 774,460<br>SES: occupation professional (n) 217,045<br>intellectual 325,746<br>admin 266,000<br>retail 122,727<br>skilled 49,201<br>unskilled 84,664<br>none 184,368<br>Ethnicity, education: NR	Post-change: Closure of units within 15km radius of home, number of units closed NR	financial pressure, efficiency savings, and staff shortage		
Hemminki 2011 (19), Finland	Retrospective population-based analysis of routinely collected data, OU closure	To describe centralisation trend, unplanned out of hospital births, perinatal mortality, health and birth	Time of OU closure: 1991-2008<br>Time of data analysis: Finland 1991-2008;	*Inclusion*: All births<br><br>*Exclusion*: NR	1991-2008 N=474,419<br><br>Characteristics: NR	Centralisation of births, maternity units number declined	Universal access to maternity care, minimal private care. Pre- and postnatal care	BBA	NR

For peer review only - http://bmjopen.bmj.com/site/about/guidelines.xhtml

	Across Finland and a specific district Uusimaa	outcomes in areas served by different levels hospitals	Uusimaa district 2004-2008			Pre-change: 49 OUs in 1991 Post-change: 34 OUs in 2008	decentralised, birth hospital-based service, care of high-risk pregnancies centralised. Mean no. births/hospital increased from 1339-1733 over study period.		
Engjom 2014 (7), Norway	Retrospective population-based study, 3 cohort and 2 cross-sectional studies, *OU closure*<br><br>Across Norway	To assess the availability of OUs, the risk of unplanned delivery outside OU and maternal morbidity	Cohort: 1979-2009 Cross-sectional: 2000 and 2010 Time of OUs closure: 1979-2009<br><br>Time of analysis: Cohort: 1979-2009 Cross-sectional: 2000 and 2010	*Included:* Age 15-49 yrs, known place of birth, GA ≥22 wks and/or bthwt ≥500g *Excluded:* Missing maternal address, planned home birth	1979-83 n=252,621 2004-09 n=409,432<br><br>Characteristics: NR	Declined in number of OUs in Norway Pre-change: 95 OUs in 1979 Post-change: 51 OUs in 2009	Universal access to maternity care; relatively dispersed population	Perinatal mortality (PM)	CS
Grytten 2014 (18), Norway	Retrospective population-based analysis of routinely collected data, OU closure	To study whether neonatal and infant mortality were independent of the type of hospital in which	Time of closures: between 1979 and 2005 Time period for analysis: 5 years pre-and post-	*Inclusion*: All births<br><br>*Exclusion*: NR	N=33,677<br><br>Characteristics: NR	Centralisation /OU closures Number of local hospitals fell from 43 to 26 between 1979 and 2005. 17 maternity wards	Universal free access to maternity care. Low-risk births in local hospitals, high risk in central/ regional	NM; Infant mortality (IM)	NR

For peer review only - http://bmjopen.bmj.com/site/about/guidelines.xhtml

	Across Norway	the delivery was carried out	closure for each hospital			in local hospitals closed<br>Pre-change:<br>22 hospitals with neonatal department; 43 local hospitals; 30 maternity clinics<br><br>Post-change:<br>22 hospitals with neonatal department; 26 local hospitals, 10 maternity clinics	hospitals. Births in Central/Regional hospitals increased from 65-81% over study period.		
**CANADIAN STUDIES**									
Le Coutour 1990 (22), Canada	Retrospective population-based analysis of routinely collected data, OU closure<br><br>Montreal metropolitan area	To describe the evolution of obstetric practice in Montreal metropolitan area before/ after closure of units	Time of closure: 1984-1985<br>Time of analysis: 1981-1985	*Inclusion*: All births<br><br>*Exclusion*: NR	1981-1984<br>N=128,688<br><br>Characteristics: NR	7/13 university hospitals, 5/13 specialist hospitals, 4/13 smaller units closed<br>Pre-change: 39 units in 1981<br>Post-change:16	State provision of maternity care. Closures due to budgetary restrictions.	NR	Overall CS
Allen 2004 (23), Canada	Retrospective population-based analysis of routinely collected data, OU closure	To evaluate the effect of hospital closures on critical obstetrical interventions and perinatal	Pre-closure: 1988-1993<br><br>Post-closure: 1996-2002	*Inclusion*: All births<br><br>*Exclusion*: Delivery <20 weeks;	1988-93 n=69,213<br>1996-2002 n=63,510<br>*Range %*	1988-1993 =27 hospitals<br>1996-2002 =19 hospitals	State provision of maternity care. Reduction in number of	SB; Fetal/ neonatal mortality (NM)	NR

	Eastern, Northern, Western, and Central in Nova Scotia	outcomes in rural communities	Time of analysis: 1988 – 1993 vs. 1996 - 2002	bthwt <500g; triplets+; major congenital anomaly	Age >34 yrs: 5.6-14.8; Nullip 39.4-46.8 Twins 1.0-1.3<br><br>Ethnicity, socioeconomic status, education: NR	Reduction in maternity units from 42 to 11 between 1970 and 2002 Pre-change: 42 units in 1970 Post-change: 11 units in 2002	units and physicians due to financial constraints and difficulty maintaining clinical competence and confidence.		
Hutcheon 2017 (21), Canada	Retrospective population based analysis of routinely collected data, OU closure<br><br>25 communities within British Columbia, Canada	To examine the effect of obstetric service closures on intrapartum outcomes	1998-2014 Time of closures: between 2000 and 2012<br><br>Time of analysis: 1998-2014	*Inclusion*: All births recorded in British Columbia Perinatal Data Registry (99% of deliveries) *Exclusion*: Communities close to larger metropolitan areas and or uncertainty about dates of service closures.	*Pre-closure* N=5796 Median maternal age 27 yrs (IQR 23-31); Nullip 39.3%<br><br>*Post-closure* N=6153 Median maternal age 28 yrs (IQR 24-32); Nullip 40.7% Ethnicity, Socioeconomic status, education: NR	Centralisation /OU closures Between 1998 and 2014 one-third of hospitals stopped providing maternity services Pre-change: 21 hospitals with obstetric services<br><br>Post-change: Obstetric services closed in same 21 hospitals	State provision of maternity care. Centralisation of obstetric services, majority of hospital closures in low-volume hospitals	BBA; Perinatal / Neonatal mortality ; NNU admission	Overall CS; Maternal mortality; 3rd/4th degree perineal tear, blood transfusion, maternal admission to ICU

Abbreviations: Approx: approximately; bthwt: Birth Wight; BBA: Born before arrival; CS: Caesarean Section; excl: Excluded; ITU: Intensive Care Unit; IQR: Interquartile Range; MW: Midwife; NHS: National Health Services; NNU: Neonatal Unit; NM: Neonatal Mortality, NR: Not Reported; Nullip: Nulliparous; OU: Obstetric unit; SB: Stillbirth; wo: Without; yr: Year. *Blondel 2011 is also included in travel distance.

For peer review only

For peer review only - http://bmjopen.bmj.com/site/about/guidelines.xhtml

Table 1B: Risk of bias - Obstetric unit closure studies

Author, year, country	Study sample selection bias	Bias in measurement of exposure	Bias in measurement of outcomes	Attrition bias	Analysis method reported and appropriate	Closure independent of other changes over time	Potential confounders adjusted for and listed
**UK STUDIES**							
Fleming/East Lancashire study							
Fleming 2013 (15), UK (abstract)	**LOW** All births in East Lancashire Maternity Services catchment	**LOW** All births in catchment area affected by the closure Number of OUs closed reported	**LOW** Objective outcome (CS)	**UNCLEAR** Not reported	**UNCLEAR** None reported	**UNCLEAR** None reported	**UNCLEAR** None reported
East Lancashire Hospitals NHS Trust 2017 (17), UK (unpublished)	**UNCLEAR** Unpublished data, no details	**UNCLEAR** Unpublished data, no details	**LOW** Objective outcomes (BBA, SB, NM)	**UNCLEAR** Unpublished data, no details	**UNCLEAR** Unpublished data, no details	**UNCLEAR** Unpublished data, no details	**UNCLEAR** Unpublished data, no details
Mackie 2014 (16), UK (abstract & unpublished)	**LOW** Data from Maternity Information System	**LOW** All births in catchment area affected by the closure. Number of OUs closed reported	**LOW** Objective outcomes (SB, NM, BBA, MM, ICU admission, perineal tears)	**UNCLEAR** Not reported	**UNCLEAR** None reported	**UNCLEAR** None reported	**UNCLEAR** None reported

For peer review only - http://bmjopen.bmj.com/site/about/guidelines.xhtml

**OTHER EUROPEAN STUDIES**							
Blondel 2011 (5), France*	**LOW** Data from birth certificates	**LOW** Number of OUs closed reported	**LOW** Objective outcome (BBA)	**LOW** 11% excluded due to missing data	**LOW** Analysis method was described and appropriate, a multi-level model analysis	**UNCLEAR** None reported	**LOW** Adjusted for maternal age, occupational category and rurality
Hemminki 2011(19), Finland	**LOW** Data from Finnish medical birth register	**LOW** All births in catchment area affected by the closure Number of OUs closed reported	**LOW** Objective outcome (BBA)	**LOW** Births with missing information excluded (<0.05%)	**LOW** Analysis method was described and appropriate, a regression model with adjusted analysis	**UNCLEAR** None reported	**LOW** Adjusted for Parity, plurality, age, socioeconomic status and smoking
Engjom 2014 (7), Norway	**LOW** Data from Medical Birth Registry of Norway	**LOW** All births in Norway affected by the closure Number of OUs closed reported	**LOW** Objective outcome (BBA)	**LOW** All units report to Medical Birth Registry	**LOW** Analysis method appropriate, a logistic regression model, crude and adjusted results given	**UNCLEAR** None reported	**LOW** Adjusted for maternal age, parity, education and partner status
Grytten 2014 (18), Norway	**LOW** Data from Medical Birth Registry of Norway	**LOW** All births in Norway Number of OUs closed reported	**LOW** Objective outcomes (NM, Infant mortality)	**LOW** All maternity units report to Medical Birth Registry	**Unclear** Difference-in-difference statistical method used, but reporting of findings were unclear	**LOW** Adjusted for trend in infant outcomes based on local hospitals that were not closed.	**LOW** Maternal age, immigrant status, level of education, marital status, predisposing medical factors and characteristics of the birth

For peer review only

**CANADIAN STUDIES**							
Le Coutour 1990 (22), Canada	**LOW** Data from MED-ECHO - regional data collection system	**LOW** All births in catchment area Number of OUs closed reported	**LOW** Objective outcome (CS)	**UNCLEAR** No information	**UNCLEAR** No details on data analysed method	**UNCLEAR** None reported	**HIGH** No adjustment
Allen 2004 (23), Canada	**LOW** Data from Nova Scotia Atlee Perinatal Database	**LOW** All births in catchment area affected by the closure Number of OUs closed reported	**LOW** Objective outcomes (SB, NM)	**UNCLEAR** Population based dataset but no information about missing data	**LOW** Analysis method appropriate and data from logistic regression models were reported	**UNCLEAR** None reported	**LOW** Maternal age, smoking and maternal diseases
Hutcheon 2017 (21), Canada	**LOW** Data from British Columbia Perinatal registry	**LOW** All births in catchment area affect by the closure Number of OUs closed reported	**LOW** Except for 3rd/4th degree tears. Objective outcomes (BBA, PM, NM, ICU admission, CS, MM, blood transfusion, Maternal admission to ICU)	**LOW** >99% complete	**LOW** Used a within-community fixed-effects design and Poisson regression	**LOW** Using difference in difference analysis which separates the effect of the closure from underlying time trends of reported outcomes	**HIGH** No adjustment

Abbreviations: BBA: Born Before Arrival; CS: Caesarean Section; ICU: Intensive Care Unit; MM: Maternal Mortality; NHS: National Health Services; NM: Neonatal Mortality; PM: Perinatal Mortality; SB: Stillbirth. *Blondel 2011 included in travel distance & OU closure.

For peer review only - http://bmjopen.bmj.com/site/about/guidelines.xhtml

Table 1C: Outcomes - Obstetric unit closure

Outcomes	Author, Year, Country	Exposure and comparator groups	Participants (N, n, %)			Findings		
**MATERNAL OUTCOMES**								
**Maternal mortality (MM)**	Mackie 2014 (16), UK (unpublished data)	Before and after amalgamation of 2 OUs	**Year**	**Deliveries (N= 15,349)**	**MM n (%)**	**Year**	**Crude OR (95%CI)**	**Adjusted OR (95%CI)**
			Pre 2010-2011	5354	1 (0.02)	Pre 2010-2011	1	NR
			Post 2011-2013	9995	1(0.01)	Post 2011-2013	*0.54 (0.03, 8.56)*	NR
	Hutcheon 2017 (21), British Columbia, Canada	Before and after OU closure (1998-2014)	**Closure status**	**Deliveries (N=11,949)**	**Maternal deaths n (%)**	**Closure status**	***Crude OR (95%CI)***	***Adjusted OR (95%CI)***
			Preclosure	5796	<5 (<0.09)	Preclosure	NR	NR
			Postclosure	6153	<5 (<0.08)	Postclosure	NR	NR
			No significant difference pre/post closure in adverse events during labour and delivery					
**Caesarean section (CS) (overall or intrapartum)**	Fleming 2013 (15), UK (abstract)	Before and after closure of OU in 2010	***Closure status***	***Deliveries (N=NR)***	***CS* n (%)**	**Closure status**	**Crude OR (95%CI)**	**Adjusted OR (95%CI)**
			Preclosure, early 2010	NR	(NR) 26.1	Preclosure, early 2010	NR	NR
			Postclosure, 2012	NR	(NR) 21.5	Postclosure, 2012	NR	NR

For peer review only - http://bmjopen.bmj.com/site/about/guidelines.xhtml

			Proportions of CS presented with no other data.					
	Engjom 2014 (7), Norway	2000 compared to 2009 during which time number of OUs declined from 47 to 41	**Year**	**Deliveries (N=2,177,934)**	**CS n (%)**	**Year**	**Crude OR (95% CI)**	**Adjusted OR (95%CI)**
			Pre 2000	58,632	7653 (13.10)	Pre 2000	1	NR
			Post 2009	61,895	10,154 (16.41)	Post 2009	*1.31 (1.27, 1.35)*	NR
	Le Coutour 1990 (22), Quebec, Canada	Before and after closure of OUs between 1982 and 1983	**Year**	**Deliveries (N= 64,274)**	**CS n (%)**	**Year**	**Crude OR (95% CI)**	**Adjusted OR (95%CI)**
			Pre 1981	32,807	5852 (17.84)	Pre 1981	1	NR
			Post 1984	31,467	6214 (19.7)	Post 1984	*1.13 (1.09, 1.18)*	NR
	Hutcheon 2017 (21), British Columbia, Canada	Before and after OUs, closure (1998-2014)	**Closure status**	**Deliveries (N= 11,949)**	**CS n (%)**	**Closure status**	**Crude OR (95%CI)**	**Adjusted OR (95%CI)**
			Preclosure	5796	1387 (23.93)	Preclosure	1	NR
			Postclosure	6153	1579 (25.70)	Postclosure	*1.10 (1.01, 1.19)*	NR
**Emergency caesarean section (CS)**	Mackie 2014 (16), UK (abstract only)	Before and after amalgamation of two OUs	**Year**	**Deliveries (N=15,349)**	**Emergency CS n(%)**	**Year**	**Crude OR (95%CI)**	**Adjusted OR (95%CI)**
			Pre 2010-2011	5354	739 (13.80)	Pre 2010-2011	1	NR
			Post 2011-2013	9995	1322 (13.23)	Post 2011-2013	*0.95 (0.86, 1.05)*	NR
**Severe perineal trauma (3rd or 4th**	Mackie 2014 (16), UK (unpublished data)	Before and after amalgamation of two OUs	**Year**	**Deliveries (N=15,349)**	**3rd & 4th n (%)**	**Year**	**Crude OR (95%CI)**	**Adjusted OR (95%CI)**
			Pre 2010-2011	5354	133 (2.48)	Pre 2010-2011	1	NR

For peer review only

For peer review only - http://bmjopen.bmj.com/site/about/guidelines.xhtml

**degree tear)**			Post 2011-2013	9995	276 (2.76)	Post 2011-2013	*1.11 (0.90, 1.37)*	NR
	Hutcheon 2017 (21), British Columbia, Canada	Before and after OU closure (1998-2014)	**Closure status**	**Deliveries (N=11,949)**	**3rd or 4th degree tear n (%)**	**Closure status**	**Crude OR (95%CI)**	**Adjusted OR (95%CI)**
			Preclosure	5796	136 (2.40)	Preclosure	1	NR
			Postclosure	6153	174 (2.82)	Postclosure	*1.21 (0.96, 1.52)*	NR
**Postpartum haemorrhage**	No studies							
**Maternal admission to ICU**	Mackie 2014 (16), UK (abstract only)	Before and after amalgamation of two OUs	**Year**	**Deliveries (N=15,349)**	**n (%)**	**Year**	**Crude OR (95%CI)**	**Adjusted OR (95%CI)**
			Pre 2010-2011	5354	18 (0.34)	Pre 2010-2011	1	NR
			Post 2011-2013	9995	27(0.27)	Post 2011-2013	*0.80 (0.44, 1.46)*	NR
	Hutcheon 2017 (21), British Columbia, Canada	Before and after OU closure (1998-2014)	**Closure status**	**Deliveries (N= 11,949)**	**ICU admission n**	**Closure status**	**Crude OR (95%CI)**	**Adjusted OR (95%CI)**
			Preclosure	5796	<5	Preclosure	NR	NR
			Postclosure	6153	<5	Postclosure	NR	NR
**Maternal Blood transfusion**	Hutcheon 2017 (21), British Columbia, Canada	Before and after OU closure (1998-2014)	**Closure status**	**Deliveries (N= 11,949)**	**Blood transfusion n (%)**	**Closure status**	**Crude OR (95%CI)**	**Adjusted OR (95%CI)**
			Preclosure	5796	53 (0.91)	Preclosure	1	NR
			Postclosure	6153	46 (0.75)	Postclosure	*0.82 (0.55, 1.21)*	NR

For peer review only

NEONATAL OUTCOMES								
**Stillbirth (SB)**	East Lancashire Hospitals NHS Trust, 2017 (17), UK (unpublished data)	Before and after amalgamation of two obstetric units (OUs) in 2010	**Year**	**Deliveries (N= 53,870)**	**SB n (%)**	**Year**	**Crude OR (95%CI)**	**Adjusted OR (95%CI)**
			Pre 2009	6492	75 (1.16)	Pre 2009	1	NR
			Post 2011-2017	47,378	333 (*0.70*)	Post 2011-2017	*0.61 (0.47, 0.78)*	NR
			Stillbirth>24 weeks					
	Allen 2004 (23), Nova Scotia, Canada	Before and after closure of OUs	**Year**	**Deliveries (N=132,723)**	**SB n (%)**	**Year**	**Crude OR (95%CI)**	**Adjusted OR (95%CI)**
			Pre 1988-93	69,213	291 (*0.42*)	Pre 1988-1993	1	NR
			Post 1996-2002	63,510	214 *(0.34*)	Post 1996-2002	*0.80 (0.67, 0.96)*	NR
	Mackie 2014(16), UK (unpublished data)	Before and after amalgamation of 2 OUs	**Year**	**Deliveries (N=15,552)**	**SB n (%)**	**Year**	**Crude OR (95%CI)**	**Adjusted OR (95%CI)**
			Pre 2010-2011	5422	29 (*0.53*)	Pre 2010 -2011	1	NR
			Post 2011-2013	10,130	60 (*0.59*)	Post 2011-2013	*1.11 (0.71, 1.73)*	NR
**Neonatal mortality (NM)**	East Lancashire Hospitals NHS Trust, 2017 (17), UK (unpublished data)	Before and after amalgamation of two obstetric units in 2010	**Year**	**Deliveries (N=53,870)**	**NM n (%)**	**Year**	**Crude OR (95%CI)**	**Adjusted OR (95%CI)**
			Pre 2009	6492	4 (*0.06*)	Pre 2009	1	NR
			Post 2011-2017	47,378	39 (*0.08*)	Post 2011-2017	*1.33 (0.81, 2.17)*	NR
			NM not defined					

For peer review only - http://bmjopen.bmj.com/site/about/guidelines.xhtml

	Mackie 2014(16), UK (unpublished data)	Before and after amalgamation of 2 OUs	**Year**	**Deliveries (N=15,552)**	**NM n (%)**	**Year**	**Crude OR (95%CI)**	**Adjusted OR (95%CI)**
			Pre 2010-2011	5422	6 (*0.11*)	Pre 2010-2011	1	NR
			Post 2011-2013	10,130	9 (*0.90*)	Post 2011-2013	*0.80 (0.29, 2.26)*	NR
	Grytten 2014 (18), Norway	Before and after 13 hospital closures	**Year**	**Deliveries (N=33,677)**	**NM n (%)**	**Year**	**Crude OR (95%CI)**	**Adjusted OR(95%CI)**
			5 yrs before	16,297	NR	5 yrs before	NR	NR
			5 yrs after	17,380	NR	5 yrs after	NR	NR
			No statistically significant difference					
**Perinatal mortality (PM)**	Engjom 2014 (7), Norway	2000 compared to 2009 during which time number of OUs declined from 47 to 41	**Year**	**Deliveries (N=2,177,934)**	**PM n (/1000 births)**	**Year**	**Crude OR (95%CI)**	**Adjusted OR(95%CI)**
			Pre 2000	58,632	124 (2.11)	Pre 2000	1	NR
			Post 2009	61,895	99 (1.60)	Post 2009	*0.76 (0.58, 0.98)*	NR
			PM (Intrapartum & neonatal death < 24 hrs, both live & still born)					
	Allen 2004 (23), Nova Scotia, Canada	Before and after closure of OUs	**Years**	**Deliveries (N=132,723)**	**PM n (%)**	**Years**	**Crude OR (95%CI)**	**Adjusted OR (95%CI)**
			Pre 1988-1993	69,213	422 (0.61)	Pre 1988-93	1	NR
			Post 1996-2002	63,510	278 (0.43)	Post 1966-2002	*0.75 (0.64, 0.87)*	*NR*
			Fetal/neonatal mortality not defined					
**Infant mortality**	Grytten 2014 (18), Norway	Before and after 13	**Year**	**Deliveries (N=33,677)**	**IM n (%)**	**Years**	**Crude OR (95%CI)**	**Adjusted OR (95%CI)**

For peer review only - http://bmjopen.bmj.com/site/about/guidelines.xhtml

		hospital closures	5 yrs before	16,297	NR	5 yrs before	NR	NR
			5 yrs after	17,380	NR	5 yrs after	NR	NR
			No significant difference in infant mortality					
**Born before arrival (BBA)**	East Lancashire Hospitals NHS Trust, 2017 (17), UK (unpublished data)	Before and after amalgamation of two obstetric units	**Year**	**Deliveries (N= 53,870)**	**BBA n (%)**	**Year**	**Crude OR (95%CI)**	**Adjusted OR (95%CI)**
			Pre 2009	6492	25 (0.39)	Pre 2009	1	NR
			Post 2011-2017	47,378	341 (0.72)	Post 2011-2017	*1.88 (1.25, 2.82)*	NR
	Mackie 2014 (16), UK (unpublished data)	Before and after amalgamation of 2 OUs	**Year**	**Deliveries (N=15,349)**	**BBA n (%)**	**Year**	**Crude OR (95%CI)**	**Adjusted OR (95%CI)**
			Pre 2010-2011	5354	11 (0.21)	Pre 2010-2011	1	NR
			Post 2011-2013	9995	26 (0.26)	Post 2011-2013	*1.28 (0.63, 2.60)*	NR
	Blondel 2011 (5), France	OU closure within 15 km radius 2003-2006	**Yrs 2003-2006**	**Deliveries (N=1,349,751)**	**BBA n (/1000 births)**	**Yrs 2003-2009**	**Crude OR (95%CI)**	**Adjusted OR (95%CI)**
			No closure	1,001,858	4531 (4.52)	No closure	1	1
			Closure within 15km radius	347,893	1209 (3.47)	Closure within 15km radius	0.77 (0.72, 0.82)	0.91 (0.84, 1.00)
	Engjom 2014 (7), Norway	1979-83 compared to 2004-09, number of emergency OUs declined from 47 to 41	**Year**	**Deliveries (N=662,053)**	**BBA n (%)**	**Year**	**Crude OR (95%CI)**	**Adjusted OR (95%CI)**
			Pre 1979-83	252,621	984 (0.39)	Pre 1979-83	1	1
			Post 2004-09	409,432	2832 (0.69)	Post 2004-09	1.8 (1.6, 1.9)	2.0 (1.9, 2.2)

For peer review only - http://bmjopen.bmj.com/site/about/guidelines.xhtml

	Hemminki 2011(19), Finland	Centralisation of hospitals over years 1991-2008	**Year**	**Births (N)**	**Unplanned BBA n(/1000)**	**Planned or unplanned BBA n(/1000)**	**Year**	**Crude OR (95%CI)**	**Adjusted OR (95%CI)**
			Pre 1991	65,632	-	68 (1.0)	Pre 1991	1	NR
			Post 2004-2008	56,873	222 (3.76)	243 (4.1)	Post 2004-2008	*4.14 (3.16, 5.41)*	NR
			Total N (1991-2008)= 122,505						
	Hutcheon 2017(21), British Columbia, Canada	Before and after OU closure (1998-2014)	**Closure status**	**Deliveries (N=11,949)**	**Unplanned BBA n (%)**	**Closure status**	**Crude OR (95%CI)**	**Adjusted OR (95%CI)**	
			Preclosure	5796	30 (0.5)	Preclosure	1	NR	
			Postclosure	6153	109 (1.8)	Postclosure	*3.47 (2.31, 5.20)*	NR	
**Neonatal unit admission (NNU) >2 days or transfer within 24 hrs of birth to ICU facility for newborn >/=2500g**	Hutcheon 2017(21), British Columbia, Canada	Before and after OU closure (1998-2014)	**Closure status**	**Deliveries (N=11,949)**	**NNU admission n (%)**	**Closure status**	**Crude OR (95%CI)**	**Adjusted OR (95%CI)**	
			Preclosure	5796	68 (1.17)	Preclosure	1	NR	
			Postclosure	6153	28 (0.46)	Postclosure	*0.39 (0.25, 0.60)*	NR	
**Apgar score (5 minute Apgar score <7)**	Hutcheon 2017(21), British	Before and after OU closure (1998-	**Closure status**	**Deliveries (N=11,949)**	**5 min Apgar score <7 n (%)**	**Closure status**	**Crude OR (95%CI)**	**Adjusted OR (95%CI)**	
			Preclosure	5796	71 (1.22)	Preclosure	1	NR	

For peer review only

	Columbia, Canada	2014) compared to communities unaffected by closure	Postclosure	6153	85 (1.28)	Postclosure	*1.13 (0.82, 1.55)*	NR
**Hypoxic Ischaemic Encephalopathy (HIE)**	No studies							

Abbreviations: aOR: Adjusted Odds Ratio; BBA: Born Before Arrival; ICU: Intensive Care Unit; NHS: National Health Services; PM: Perinatal Mortality; OR: Odds Ratio; OU: Obstetric Unit.

For peer review only

Table 2A: Description of included studies – Travel distance

**Author, year, country**	**Study design & setting**	**Study objectives**	**Study period**	**Eligibility criteria**	**Participant characteristics**	**Description of exposures Travel Distance**	**Services context information**	**Review Outcomes**	
								**Perinatal**	**Maternal**
**UK STUDIES**									
Bhoopalam 1991 (24), UK	Case-control study, 2 OUs	To establish BBA prevalence and women at risk of BBA, and	1983 – 1987	*Included cases:* Women and their BBA babies	N(BBAs) =137, 1 twins<br>All N= 398<br>Age yrs (n):	Distance (km):<br><2<br>2-7	Universal state provision, 2 units 6 miles apart, serving	BBA	NR

For peer review only - http://bmjopen.bmj.com/site/about/guidelines.xhtml

		morbidity and mortality associated with BBA births		*Included controls:* Two controls for each BBA case, one random (next born in the same hospital), one matched (next born in same hospital matched by GA and BW)<br>*Excluded:*<br>BW < 500g	<21 (69)<br>21-35 (339)<br>>35 (27)<br>Nullips (107)<br>Ethnicity (n):<br>European(191)<br>Asian (101)<br>Other (16)<br>SES & education: NR	>7	rural areas of Warwickshire		
**OTHER EUROPEAN STUDIES**									
Pasquier 2007(25), France	Retrospective, population-based cohort, 3 Level-III maternity wards with neonatal surgical centre, Rhône-Alpes Region	To examine maternal origin, distance to the nearest maternity ward with a neonatal surgical centre, on perinatal diagnosis, elective termination of pregnancy, delivery in an adequate place and neonatal mortality for pregnancies	1990-95 & 1996-2000 (2 periods separated due to changes in prenatal screening)	*Included:*<br>Fetuses with omphalocele, gastroschisis, diaphragmatic hernia or spina bifida that required surgical repair<br>*Excluded:*<br>Chromosomal anomalies fetuses and babies without anomalies	N =706 infants<br>N= 554 (analysed)<br>Age: yrs (n, %)<br><21 (15, 2)<br>21-35 (550, 82)<br>>35 (106, 16)<br>Ethnicity: (n,%)<br>Western European (393, 76)<br>Non-Western European (124, 24)<br>Parity, SES & education: NR	Distance (km):<br><11<br>11-50<br>>50	Distance to Level III maternity ward with NNU and a neonatal surgical centre, there were 3 in the Rhone-Alps Region	Neonatal Mortality (NM)	NR

For peer review only - http://bmjopen.bmj.com/site/about/guidelines.xhtml

		with severe malformations							
Blondel 2011 (5), France*	Retrospective cohort, population based study, metropolitan France	To calculate the incidence of BBA birth in relation to distance from maternity units and the impact of recent closure on different sociodemographic groups	2005-2006	*Included:* Singleton live births *Excluded:* Municipalities with >8% missing data, unrealistically high BBA births, Departments were excl. if >20% births already excl.	N=1,517,599 livebirths N=1,349,751(analysed) Age yrs - n <20 - 26,152 20-34 – 1,057,90135-39 - 213,534 40+ - 52,164 Parity: Nullip n - 774,460 SES n: Occupation professional-217,045 Intellectual-325,746 Admin- 266,000 Retail-122,727 Skilled- 149,201 Unskilled- 84,664 None- 84,368 Ethnicity & education: NR	Distance (km): <5 5-14 15-29 30-44 45+	Centralising births in larger units	BBA; BBA by parity	NR
Pilkington 2014 (26), France	Retrospective, population-based cohort study, French National Vital Statistics registry from	To investigate the impact of distance to closest maternity unit on perinatal outcomes	2001-2008, Stillbirth data 2002-2005	*Included:* All births *Excluded:* *NR*	N= 3,086,128 all births N=3,085,839(analysed) Age yrs (n) <25 - 494,689 25-34 – 2,008,320	Distance (km): <5 5-14 15-29 30-44 ≥45	1998 to 2003, 20% of maternity units closed Mean distance to	Stillbirths (SB); NM	NR

	mainland France				35-39 - 469,975<br>40+ - 113,144<br>Singleton pregnancy N= 2,988,169<br>Multiple pregnancy: N= 97,959<br>Parity, ethnicity and socioeconomic status & education: NR		nearest maternity unit increased (6.6–7.2 km)		
Ovaskainen 2015 (28), Finland	Case–control study, one centre, Tampere University Hospital	To establish if BBA births increased over time, to identify risk factors associated with BBAs, also if BBAs babies were more prone to neonatal morbidities compared to those delivered in hospital	1996 - 2011	*Included Cases:* Planned and unplanned BBA<br>*Included Controls:* 2 controls for infant and mother for each BBA case<br>*Excluded:* BBA with no information whether planned or unplanned	Cases:<br>BBAs (n = 67 births): Age yrs (mean, SD) (range)- 29.0, 5.9 (15-47)<br>Parity 1 (0-16)<br>Controls:<br>N= 134<br>Plurality, ethnicity, education & SES: NR.	Distance (km):<br><35<br>≥35	Tampere University Hospital is the catchment area for 23 municipalities, 521700 residents 5000 births/yr	BBA	NR
Fougner 2000 (27), Norway	Case-control study, 14 municipalities , Oppland County	To compare the experience and care of women who delivered during	1989 - 1997	*Included Cases:* Women who delivered their babies before	N=202<br>Cases: N= 115 BBA women<br>Parity n (%)-<br>Nullips 15 (13%)	Distance (km):<br>< 12.88<br>≥12.88	Oppland county: 4 hospitals	BBA	NR

50

		transport to hospital and women who delivered an hour after arriving to hospital		arriving at hospital<br>*Included Controls:* Women who delivered their babies with one hour after arriving at hospital<br>*Excluded:* NR	Controls: N=87 women<br>Parity- Nullips 18 (20%)<br>Age, ethnicity, education & SES: NR				
**CANADIAN STUDIES**									
Lisonkova 2011 (29), Canada	Retrospective population-based cohort study, British Columbia	To examine the association between rural residence and birth outcomes in older mothers	1999 - 2003	*Included:* Singleton mothers aged 35+<br>*Excluded:* Women with missing postcodes, babies with congenital anomaly	N= 29,698 women<br>Age >35 yrs<br>Parity n (%):<br>Nullip 87,733 (0%)<br>Low SES (n, %)<br>(4385 14% 22.6 vs 3,615, 13.7)<br>Ethnicity n(%)<br>1st nation 826 (2,8%) Education: NR	Distance (km):<br><50<br>50-150<br>>150	17 small maternity units (250-2,500 births/yr) closed between 1999 and 2003	SB; Perinatal Mortality (PM); NNU admission ≥1day	All CS; Emergency CS

Abbreviations: BBA: Born Before Arrival; BW: Birth Weight; CS: Caesarean Section; GA: Gestational Age; NNU: Neonatal Unit; Nullip: Nulliparous; SES: Socio Economic Status. *Blondel 2011 is also included in the OU closure.

Table 2B: Risk of bias – Travel distance

**Author, year, country**	**Study sample selection bias** Additional criteria for case control studies	**Bias in measurement of exposure**	**Bias in measurement of outcomes**	**Attrition bias**	**Analysis method reported and appropriate**	**Potential confounders adjusted for and listed**

For peer review only

**UK STUDIES**							
Bhoopalam 1991 (24), UK	Case definition adequate	**YES** From hospital records	**HIGH** Distance measured in straight line from home address	**LOW** Objective outcome (BBA)	**LOW** 3/134 (2.2%) BBA cases & 10/274 (3.6%) excluded from distance analysis	**HIGH** Descriptive analysis only	**HIGH** No adjustments
	Representative-ness of cases	**YES** BBA cases from 2 hospitals over 5 yrs					
	Appropriate selection of controls	**YES** 2 controls from same hospital					
	Definition of control appropriate	**YES** The outcome (BBA) could not have occurred					
	Comparability of cases and controls	**NO** Significant differences in maternal age, parity, ethnicity & antenatal booking					
**OTHER EUROPEAN STUDIES**							

For peer review only - http://bmjopen.bmj.com/site/about/guidelines.xhtml

Pasquier 2007 (25), France	**LOW** Data from France Central-East malformation registry		**LOW** GIS software used to estimate distance between maternal residence and nearest maternity ward with neonatal surgical centre	**LOW** Objective outcomes (NM)	**LOW** 12 births (0.03%) missing survival data	**LOW** Method detailed and appropriate, univariate analysis & multiple logistic regression were reported	**LOW** Adjusted for parity, ethnicity and other characteristics
Blondel 2011(5), France*	**LOW** Birth certificates		**LOW** GIS software used to estimate distance to hospital from home	**LOW** Objective outcome (BBA)	**LOW** 11% of births excluded	**LOW** Method detailed and appropriate, multilevel model was reported	**LOW** Adjusted for maternal age, occupation, parity and other characteristics
Pilkington 2014 (26), France	**LOW** French National Vital Statistics Registry		**HIGH** Distance calculated from centre of municipality not home address, using road networks provided by the French National Geography Institute	**LOW** Objective outcomes (SB, NM)	**LOW** 10% missing for type of pregnancy and 17% for maternal age	**LOW** Method detailed and appropriate, logistic regression analysis was reported	**LOW** Adjusted for maternal age, plurality, unemployment rate, single parent households
Fougner 2000 (27), Finland	Case definition adequate	**NO** Data from a questionnaire	**HIGH** Data for distance was self-reported	**LOW** Objective outcome (BBA)	**HIGH** Data from women who responded to	**HIGH** Descriptive analysis only	**HIGH** No adjustment

For peer review only

	Representative-ness of cases	**YES**<br>Cases from 3 hospitals in one county over 8 yrs	in questionnaire		questionnaire		
	Appropriate selection of controls	**Yes**<br>Women who delivered their babies within 1 hour of arriving at hospital					
	Definition of control appropriate	**Yes**<br>Women with no BBA					
	Comparability of cases and controls	**Unclear**					
Ovaskainen 2015 (28), Finland	Case definition adequate	**YES**<br>Medical records	**LOW**<br>Distance from women's home calculated using web-based route planner	**LOW**<br>Objective outcome (BBA)	**High**<br>13 out-of-hospital deliveries (19%) excluded as could not ascertain whether planned or not planned	**LOW**<br>Method detailed and appropriate, logistic regression data were given	**LOW**<br>Adjusted for single-mother, parity, and other characteristics
	Representative-ness of cases	**YES**<br>Cases from 1 centre, but over 15 yrs					
	Appropriate selection of controls	**YES**<br>Births occurring immediately preceding and following case					

For peer review only

For peer review only - http://bmjopen.bmj.com/site/about/guidelines.xhtml

	Definition of control appropriate	**YES**<br>The outcome (BBA) could not have occurred					
	Comparability of cases and controls	**No**<br>Sig. differences for parity, partnership status, smoking, antenatal visits, labour duration &distance to delivery unit					
**CANADIAN STUDIES**							
Lisonkova 2011 (29), Canada	**LOW**<br>Population based study		**HIGH**<br>GIS used to calculate distance from postcode central point of residence to hospital; mostly using straight line distance	**LOW**<br>Objective outcomes (SB; PM);<br>NNU admission)	**LOW**<br>492 (1.7%) women excluded due to missing postcodes	**LOW**<br>Method detailed and appropriate, multivariate regression analysis reported	**LOW**<br>Adjusted for parity, single mother, low income, ethnicity and other characteristics

Abbreviations: BBA: Born Before Arrival; GIS Geographical Information System; NM: Neonatal Mortality; NNU: Neonatal Unit; PM: Perinatal Mortality; Sig: Significant; SB: Stillbirth. *Blondel 2011 included in travel distance & OU closure.

For peer review only - http://bmjopen.bmj.com/site/about/guidelines.xhtml

Table 2C: Outcomes – Travel distance

Outcomes	Author, year, country	Exposure groups	Participants (N, n, %)			Findings		
**MATERNAL OUTCOMES**								
**Maternal mortality (MM)**	Ovaskainen 2015 (28), Finland	**Travel distance (km):**<br><35<br>≥35	**Groups**	**N (201)**	**NM n (%)**	No events in either group		
			< 35	NR	0			
			≥35	NR	0			
**Caesarean section (CS) (overall or intrapartum)**	Lisonkova 2011 (29), Canada	**Travel distance (km):**<br><50<br>50-150<br>>150	**Groups**	**N (29,698)**	**CS n (%)**	**Groups**	**Crude OR (95%CI)**	**Adjusted OR (95%CI)**
			<50	27,836	9099 (32.70)	<50	1	NR
			50-150	1534	464 (30.25)	50-150	*0.89 (0.80, 1.00)*	NR
			>50	328	94 (28.70)	>50	*0.83 (0.65, 1.05)*	NR
**Emergency CS**	Lisonkova 2011(29), Canada	**Travel distance (km):**<br><50<br>50-150<br>>150	**Groups**	**N (9,657)**	**Emergency CS n (%)**	**Groups**	**Crude OR (95%CI)**	**Adjusted OR (95%CI)**
			<50	9,099	5,378 (59.11)	<50	*1*	NR
			50-150	464	258 (55.60)	50-150	*0.87 (0.72, 1.05)*	NR
			>50	94	52 (55.32)	>50	*0.86 (0.57, 1.29)*	NR

For peer review only - http://bmjopen.bmj.com/site/about/guidelines.xhtml

**Severe perineal trauma**	No studies							
**Postpartum haemorrhage**	No studies							
**Admission to ICU**	No studies							
**Blood transfusion**	No studies							
**NEONATAL OUTCOMES**								
**Stillbirth (SB)**	Pilkington 2014(49), France	**Travel distance (km):** <5 5-14 15-29 30-44 ≥45	**Groups**	**N (3,085,839) (2002-2005)**	**SB n (/per 1000)**	**Groups**	**Crude OR (95%CI)**	**Adjusted OR (95%CI)**
			<5	1,404,665	*13,204* (9.4)	<5	1	1
			5-14	81, 1775	*6,657* (8.2)	5-14	*0.87 (0.85, 0.90)*	Reported as RR 0.87 (NR)*
			15-29	648,495	*5,188* (8.0)	15-29	*0.85 (0.82, 0.88)*	Reported as RR 0.85 (NR)*
			30-44	186,537	*1492* (8.0)	30-44	*0.85 (0.81, 0.90)*	Reported as RR 0.85 (NR)*
			≥45	34,367	*306* (8.9)	≥45	*0.95 (0.84, 1.06)*	Reported as RR 0.95 (NR)(NS)
	Lisonkova 2011 (29), Canada	**Travel distance (km):** <50 50-150 >150	**Groups**	**N (29,698)**	**NM n (%)**	**Groups**	**Crude OR (95%CI)**	**Adjusted OR (95%CI)**
			<50	27,836	150	<50	NR	NR
			50-150	1534	NR	50-150	NR	NR

For peer review only

			>150	328	NR	>150	NR	NR
			OR not reported. Authors noted stillbirth rate was higher among women living 50- 150 km and >150 km versus <50 km, no significant difference found after adjusting for confounders.					
**Neonatal mortality (NM)**	Pasquier 2007(25), France	**Travel distance (km):**<br><11<br>11-50<br>>50	**Groups**	**N (554)**	**NM n (%)**	**Groups**	**Crude OR (95%CI)**	**Adjusted OR (95%CI)**
			<11	239	NR	<11	NR	1
			11-50	156	NR	11-50	NR	0.98 (0.34, 2.88)
			>50	159	NR	>50	NR	1.37 (0.49, 3.86)
	Pilkington 2014 (26), France	**Travel distance (km):**<br><5<br>5-14<br>15-29<br>30-44<br>≥45	**Groups**	**N (6,202,918) (2001-2008)**	**NM n(/per 1000)**	**Groups**	**Crude OR (95%CI)**	**Adjusted RR (95%CI)**
			<5	2,808,068	*7582* (2.7)	<5	1	1
			5-14	1,626,885	*3416* (2.1)	5-14	*0.78 (0.75, 0.81)*	Reported as RR 0.91 (NR) *
			15-29	1,316,329	*2896* (2.2)	15-29	*0.81 (0.78, 0.85)*	Reported as RR 0.94 (NR)(NS)
			30-44	381,288	*801* (2.1)	30-44	*0.78 (0.72, 0.84)*	Reported as RR 0.9 (NR)*
			≥45	69,787	*154* (2.2)	≥45	*0.82 (0.70, 0.96)*	Reported as RR 0.96 (NR)(NS)
			**NM After BBA**			**NM after BBA**		
			**Groups**	**N (6,202,918) (2001-2008)**	**NM n (/per 100,000)**	**Groups**	**Crude OR (95%CI)**	**Adjusted RR (95%CI)**
			<5	2,808,068	*115* (4.1)	<5	1	1

For peer review only - http://bmjopen.bmj.com/site/about/guidelines.xhtml

			5-14	1,626,885	*65* (4.0)	5-14	*0.98 (0.72, 1.32)*	Reported as RR 1.1 (NR)(NS)
			15-29	1,316,329	*72* (5.5)	15-29	*1.34 (0.99, 1.79)*	Reported as RR 1.58 (NR)*
			30-44	381,288	*23* (6.0)	30-44	*1.47 (0.94, 2.30)*	Reported as RR 1.51 (NR)(NS)
			≥45	69,787	*7* (10.0)	≥45	*2.45 (1.14, 5.25)*	Reported as RR 3.68 (NR)*
**Perinatal mortality (PM)**	Lisonkova 2011 (29), Canada	**Travel distance (km):** <50 50-150 >150	**Groups**	**N (29,698)**	**PM n (%)**	**Groups**	***Crude OR (95%CI)***	**Adjusted OR (95%CI)**
			<50	27,836	221 (0.80)	<50	*1*	1
			50-150	1534	19 (1.24)	50-150	*1.57 (0.98, 2.51)*	1.53 (1.10, 2.12)
			>150	328	8 (2.44)	>50	*3.12 (1.53, 6.38)*	3.06 (2.20, 4.24)
**Infant mortality (IM)**	No studies							
**Born before arrival (BBA)**	Bhoopalam 1991(24), UK	**Travel distance (km):** <2 2-7 >7	**Groups**	**N (398) Cases & controls**	**BBA cases n (%)**	**Groups**	**Crude OR (95%CI)**	**Adjusted OR (95%CI)**
			<2	*59*	4 *(6.80)*	<2	*1*	NR
			2-7	*249*	88 *(35.34)*	2-7	*7.52 (2.64, 21.43)*	NR
			>7	*90*	42 *(46.70)*	>7	*12.03 (4.02, 36.01)*	NR
	Blondel 2011 (5), France	**Travel distance (km):**	**Groups**	**N (1,359,756)**	***BBA n* (Rate /1000 births)**	**Groups**	**Crude OR (95%CI)**	**Adjusted OR (95%CI)**

For peer review only - http://bmjopen.bmj.com/site/about/guidelines.xhtml

		<5<br>5-14<br>15-29<br>30-44<br>>45	<5	596,363	*1849* (3.1)	<5	*1*	NR
			5-14	352,279	*1395* (3.9)	5-14	*1.28 (1.19, 1.37)*	NR
			15-29	296,734	*1659* (5.6)	15-29	*1.81 (1.69, 1.93)*	NR
			30-44	88,670	*692* (7.8)	30-44	*2.53 (2.32, 2.76)*	NR
			>45	15,705	*182* (11.)	45+	*3.77 (3.23, 4.39)*	NR
							**Parity 1&2 N =152,426**	**Parity 3+ N = 197,325**
						**Groups**	**Adjusted OR (95%CI)**	**Adjusted OR (95%CI)**
						<5	1	1.73 (1.57, 1.90)[a]
						5-14	1.14 (1.03, 1.27)	2.32 (2.04, 2.63)
						15-29	1.39 (1.24, 1.57)	3.25 (2.84, 3.71)
						30-44	1.78 (1.55, 2.05)	3.71 (3.13, 4.41)
						>45	2.47 (2.02, 3.02)	6.46 (4.92, 8.48)
	Ovaskainen 2015 (28), Finland	**Travel distance (km):**<br>< 35<br>≥35	**Groups**	**N (201)**	**BBA n (%)**	**Groups**	**Crude OR (95%CI)**	**Adjusted OR (95%CI)**
			BBA cases	67	NR	<35	NR	1
			Controls	134	NR	≥35	NR	5.02 (1.80, 14.04)
			<35 km	NR	NR			

For peer review only

For peer review only - http://bmjopen.bmj.com/site/about/guidelines.xhtml

			≥35 km	NR	NR			
	Fougner 2000 (27), Norway	**Travel distance (km):** <12.88 ≥12.88	**Groups**	**N (202) Cases & controls**	**BBA n (%)**	**Groups**	**Crude OR (95%CI)**	**Adjusted OR (95%CI)**
			<12.88	*90*	*44 (48.90)*	<12.88	1	NR
			≥12.88	*112*	*71 (63.34)*	≥12.88	*1.81 (1.03, 3.18)*	NR
**Neonatal unit admission (NNU)**	Lisonkova 2011(29), Canada	**Travel distance (km):** <50 50-150 >150	**Groups**	**N (15,325)**	**NNU n (%)**	**Groups**	**Crude OR (95%CI)**	**Adjusted OR (95%CI)**
			<50	14,333	648 (4.80)	<50	1	NR
			50-150	815	32 (3.92)	50-150	*0.86 (0.60, 1.24)*	NR
			>150	177	12 (6.80)	>150	*1.54 (0.85, 2.77)*	NR
**Apgar score**	No studies							
**HIE**	No studies							

Abbreviations: CS: caesarean section; GA: Gestational age; HIE: Hypoxic-ischemic encephalopathy; ICU: intensive care unit; NNU: neonatal unit; NS: not significant, NR: not reported; RR: relative risk, OR: Odd ratio.

*Significant difference

[a] Reference group: women with 1&2 parity and < 5 km

For peer review only

For peer review only - http://bmjopen.bmj.com/site/about/guidelines.xhtml

Table 3A: Description of included studies – Travel time

Author, year, country	Study design & setting	Study objectives	Study period	Eligibility criteria	Participant characteristics	Description of exposures Travel Distance	Services context information	Review Outcomes	
								Perinatal	Maternal
**UK STUDIES**									
Dummer & Parker 2004 (30), UK	Retrospective population-based cohort study, Cumbria	To investigate whether geographical accessibility to hospitals affected SB rates and infant mortality	1950-1993 grouped: 1950-59 1960-69 1970-79 1980-93	*Included:* All births *Excluded:* Women with missing postcodes	N=283,668 births<br><br>Other characteristics: NR	Travel time (mins):<br>< 17<br>18-35<br>>35	Universal state provision of maternity care. 1950-93: 4 hospitals opened, 2 closed	Neonatal mortality (NM); Early NM; Post NM	NR
Paranjothy 2013, 2014 (31, 32), UK (abstract& full paper)	Retrospective cohort study, All Wales Perinatal Survey & National Community Child Health Database	To study the association between travel time from home to OU on intrapartum stillbirth (SB) & NM	1995-2009	*Included:* All registered birth >23 wks GA *Excluded:* Antepartum SB, lethal congenital anomalies, multiple pregnancies, invalid or missing GA, missing	N= 466,255 singleton births<br>*Maternal age yrs %*<br>*<20 9.7*<br>*20-34 76.5*<br>*34-44 13.8*<br>*45+ 0.1*<br>*Parity:*<br>*Nullips 44.9%*<br>*Social deprivation quintile %:*<br>*1 (least depr) 16.7*<br>*2-4 57.8*	Travel time (mins):<br><15<br>15-29<br>30-44<br>>45	Universal state provision of maternity care. 50 hospitals (16 outside Wales)	Intrapartum SB; Early NM; Late NM	NR

For peer review only - http://bmjopen.bmj.com/site/about/guidelines.xhtml

				maternal age/postcode/ hospital of birth or baby's gender	*5 (most depr) 25.6* Ethnicity, education: NR				
**OTHER EUROPEAN STUDIES**									
Combier 2013 (33), France	Retrospective cohort study, based on hospital discharge summaries, Burgundy	To analyse the effect of travel time to closest OU on pregnancy outcome & prenatal management in Burgundy	2002 - 2009	*Included:* Singleton births >21 wks GA *Excluded:* Medical ToP, multiple pregnancy, births outside Burgundy, births in 2002 & 2008 due to closure of 3 units	N=111,001 births<br><br>Other characteristics =NR	Travel time (mins):<br>≤15<br>16-30<br>31-45<br>≥46	2000-2001: 2 private maternity units closed 2002-2009: 3 public maternity units closed.<br><br>Units(n):<br>2000 (20)<br>2009 (15)	SB; Perinatal mortality (PM); BBA	NR
Renesme 2013 (35), France	Case-control, multicentre study, 8 units, Finistere District, Brittany	To evaluate the social-geographical factors associated with BBAs	2007 - 2009	*Included cases:* BBA of live birth *Included controls*: 2 controls for each case irrespective of delivery mode. *Excluded:* GA <22 weeks,	N=225<br>Cases vs controls<br>N= 76 vs 149<br>Age (median, range) yrs: 30(16–41) vs 30(16-41)<br>Parity (median, range): 2(1–6)<br>Maternal INSEE code n(%): 1, 2, 3 or 4 = 15 (23.8) vs	Travel time (mins):<br>15<br>15–29<br>30–44<br>>45	9700 births/year in Finistere In 2012 units with <300 births/yr were closed. Universal state provision of maternity care	BBA	NR

For peer review only - http://bmjopen.bmj.com/site/about/guidelines.xhtml

				BW <500g, planned home birth	56(43.4); 5 or 6: 20 (31.8) vs 55 (42,6); 8 = 28 (44.4%) vs 18(14)<br>Ethnicity, education*:* NR				
Nguyen 2016 (34), France	Case control study, university hospital in Caen	To estimate the incidence of BBA during the study period	2002-2009	*Included cases:* Unplanned BBA<br>*Included controls:* Next spontaneous birth in hospital<br>*Excluded*: NR	N=188<br>*Cases* n=94<br>Mean age: 28.9 yrs<br>Parity: 1.8<br>SES: 73.4% no profession/student<br>Control n=94<br>Mean age: 29.2 yrs<br>Parity: 0.9<br>SES: 47.9% no profession/student<br>Ethnicity, education: NR	Travel time (mins):<br>≤ 20 mins<br>>20 mins	University Hospital with neonatal care facilities. Universal state provision of maternity care	BBA	NR
RAVELLI STUDY									

For peer review only - http://bmjopen.bmj.com/site/about/guidelines.xhtml

Ravelli 2011 (36-38), Netherlands (full papers & abstract**)**	Retrospective population-based cohort study, rural and urban areas, 12 provinces	To study the effect of travel time from home to OU on mortality and other adverse outcomes in pregnant women at term in primary and secondary care	2000 – 2006	*Included:* Singleton term births *Excluded:* Antepartum deaths, congenital anomalies, invalid/missing postcodes or outpatient codes, or births from Wadden islands, home deliveries, hospitals participated for 1-2 yrs	N=751,926 singleton births Age yrs ,% <20, 2 20-34, 78.3 35-39, 17.2 ≥40, 2.4 Parity*:* Nullips: 49.9% Ethnicity*:* White 81.7% SES % *: high 25.2, medium 48.2* , low 26.7 Education: NR	Travel time (mins): < 20 >=20	Universal state provision of maternity care. 99 OUs including tertiary perinatal centres	NM (Combined intrapartum & early & late NM up to 28 days) NM (0-24 hrs) NM (0-27 days) NM(8-27 days); Combined (mortality and/or Apgar <4 at 5min, and/or NNU admission)	NR
Ravelli 2012 (39), Netherlands	Retrospective cohort study in 9 regions	To investigate provincial differences in perinatal mortality and to determine the influence of different risk factors, including travel time from home to	2000-2006	*Included:* Singleton births *Excluded:* Women with incorrect post codes	N=1,242,725 singletons Age yrs, % <20, 1.8 >35, 19.5 Parity, % Nullips, 46.3 Ethnicity*, %* Non-western 16.2 SES low (10th centile): 10% Education NR	Travel time(mins): <20 >=20	Universal state provision of maternity care	PM	NR

For peer review only - http://bmjopen.bmj.com/site/about/guidelines.xhtml

		the OU during labour							
Stolp 2015 (40), Netherlands	Prospective cohort study, rural & urban areas	To assess whether the limit of 45 mins is met for ambulance transfer of women with PPH after home birth	2008-2010	*Included:* Women with PPH after MW supervised home birth *Excluded:* Cases of PPH with missing data	N= 72 (54 analysed) Age median (range) yrs: 31 (23-41); Parity (n,%): Primip 27-50% Ethnicity, Education, SES: NR	Travel time (mins): <45 >45	Home birth for low risk women and hospital birth. Universal state provision of maternity care	NR	Maternal admission to intensive care; (ICU); Blood transfusion; Postpartum haemorrhage (PPH)
ENGJOM STUDY									
Engjom 2017 & 2015 (41, 42), Norway (full paper & abstract)	Retrospective population-based cohort study, Medical Birth Registry of Norway and Statistics Norway, 19 counties	To assess peripartum mortality associated with place of birth and availability of obstetric units.	1999-2009	*Included:* All births in Norway with GA >=22 wks or BW>=500g *Excluded:* Lack of address and municipality, antepartum stillbirths, planned home births	N= 646,898 96.4% singletons. Age yrs, % <20 2.4 20-35, 80.7 >35, 16.9%; Multips 58.7%; Education >11y 77.2 Ethnicity: Western 90.7%	Travel time (hrs): <1 1-2 >2	Basic obstetric care for normal delivery; Emergency obstetric care < 1500 births/yr. Universal state provision of maternity care	BBA	NR
**CANADIAN STUDIES**									
Grzybowski & Stroll Study									

For peer review only

For peer review only - http://bmjopen.bmj.com/site/about/guidelines.xhtml

Grzybowski 2011(44), Canada	Retrospective cohort study, rural areas of British Columbia	To document newborn and maternal outcomes in relation to travel time to the nearest OU with CS capability	2000-2004	*Included:* All deliveries >20 weeks' GA *Excluded:* Multiple birth, congenital anomalies or late ToP, core urban areas	*N=35,426 births* *Groups:* *< 1 hr, 1-2, 2-4, >4 hrs* Group N: 32,814, 1359, 747, 506 Mean maternal age yrs: 28.7,28.67, 27.25, 27.2 Parity % primips: 42.6, 38.6, 36.7, 36.8 SES[1]: 0.12, 0.10, 0.30, 0.33, 1st Nations % 0.05, 0.30, 0.23, 0.42 Education: NR	Travel time (hrs): <1 1-2 2-4 >4	Universal medical coverage for core health care, 13 NNUs, 42,000 births /year	Perinatal mortality (SB & early NND); BBA; NNU admission	CS
Grzybowski, 2013 (45), Canada	Retrospective cohort study, rural areas of British Columbia	To compare rural maternity care by level of services	2000 - 2007	*Included:* Singleton births *Excluded:* Women with residential postcode of large urban centres	N=4672 births; Mean age, yrs: 27.7 Parity: primips: 39.7 % SES[1]: 0.22%; Ethnicity: 1st Nations 0.3% Education: NR	Travel time (hrs): <1 >1	Universal medical coverage for core health care	SB; NND (late < 1 month); PM; IM; BBA; NNU admission	CS; Emergency CS; PPH
Grzybowski 2015 (46), Canada	Retrospective cohort study, British Columbia (BC), Alberta, Nova Scotia (NS)	To examine the safety of rural Canadian maternity services	2003-2008	*Included:* Singleton deliveries *Excluded:* Multiple births, infants born with congenital anomalies, planned	*Alberta, BC, NS* Age yrs (n%) <18: 1618 (2.3), 1256 (2.0), 413 (2.2) >35 yrs: 5127 (7.3), 8866 (14.3), 2387 (12.7) Multips n(%) 41730 (59.6), 35089 (56.6), 10656 (56.8)	Travel time (hrs): <1 1-2 2-4 >4	Universal medical coverage for core health care , 20 small maternity closures since 2000	Perinatal mortality (SB & NND up to 7 days)	CS

For peer review only - http://bmjopen.bmj.com/site/about/guidelines.xhtml

				home births, accidental BBA	Ethnicity, SES, education: NR				
Stoll 2014 (47), Canada	Retrospective cohort study, rural British Columbia	To report on characteristics and perinatal outcomes of rural women with only MW involved in care	2003-2008	*Included:* Women residing outside core urban areas, singletons >20wk GA & care by a MW *Excluded:* Late ToP, congenital anomalies	*< 1 hr, 1-2 hrs, > 2hrs:* N= 3438, 124, 130 Mean age yrs: 29.78, 31.4, 30.5 Primips n(%) 1574 (45), 63 (50.8), 63 (48.5) Ethnicity, SES & Education*:* NR	Travel time (hrs): <1 1-2 >2	Universal medical coverage for core health care, closure of 22 rural maternity services	Perinatal mortality (SB & NND up to 7 days)	CS
Darling 2019 (43), Canada	Retrospective population-based cohort study, Ontario	Whether greater diving distances to OU associated with a higher risk of adverse neonatal outcomes	2012-2015	*Included:* Women who planned home births regardless of actual place of births *Excluded:* Multiple births, Preterm < 37 wks	*N= 11,869* *Age yrs,%:* <25, 9.5 25-39,87.6 ≥40, 2.9 Primps n (%) 4208 (35.5) SES low, n(%) 2465(20.8) Ethnicity & education: NR	Travel time (mins): ≤30 >30	Universal medical coverage for core health care	Perinatal mortality (PM); NNU admission; 5-mins Apgar <7	CS
**OTHER COUNTRIES**									

68

For peer review only - http://bmjopen.bmj.com/site/about/guidelines.xhtml

Aoshima 2011 (48), Japan	Before and after study design, data from perinatal care centres	Whether reducing travel time influences the neonatal mortality rate (NM)	2002-2006	*Included:* All births *Excluded:* Municipalities consisting of isolated islands	Number of births: 2002= 347,284 2006 = 322,514 Other characteristics: NR	Travel time (hrs): <= 1 >1	Universal healthcare insurance system, 346 perinatal care centres	NM	NR

Abbreviations: BBA: Born Before Arrival; Bthwt: Birth Weight; depr: deprive; GA: Gestational Age; IM: Infant Mortality; LBW: low birth weight; MW: midwife; NNU: Neonatal Unit; NM: Neonatal Mortality;NND: neonatal death; OBs: Obstetrician; PM: Perinatal mortality; PPH: postpartum haemorrhage; SB: stillbirth; SES: Socioeconomic status; ToP: Termination of pregnancy .

INSEE: Institute National de la Statistique et des Etudes Economiques; INSEE codes: 1: farmer; 2: craftsperson, merchant or entrepreneur; 3: business executive, intellectual occupation; 4: other professionals; 5: employee; 6: worker; 8: no occupation;

[1]SES: Catchment level Social vulnerability -1 to +1

[2]GPESS = general practitioner with enhanced surgical skills.

For peer review only - http://bmjopen.bmj.com/site/about/guidelines.xhtml

Table 3B: Risk of bias – Travel time

Author, year, country	**Study sample selection bias** Additional criteria for case control studies	**Bias in measurement of exposure**	**Bias in measurement of outcomes**	**Attrition bias**	**Analysis method reported and appropriate**	**Potential confounders adjusted for and listed**
**UK STUDIES**						
Dummer & Parker 2004 (30), UK	**LOW** Cumbrian Births Database	**LOW** Modelled using GIS	**LOW** Objective outcome (NM)	**LOW** Of 3,352 live births, 42 stillbirths excluded as the outcome NM	**LOW** Method detailed, results of LR were reported	**LOW** Adjusted for year of birth, social class, birth order, multiple births
Paranjothy 2013 & 2014 (31, 32), UK	**LOW** National Community Child Health Database & All Wales Perinatal Survey (AWPS)	**HIGH** Women's address replaced by population-weighted centroid, travel time calculated using Google Maps API (v3)	**LOW** Objective outcomes (SB, NM)	**LOW** 11% excluded where information on parity was missing	**LOW** Analysis method described and multilevel LR data were reported	**LOW** Adjusted for maternal age, parity, urban/rural location, SES, and other characteristics
**EUROPEAN STUDIES**						
Combier 2013 (33), France	**LOW** Burgundy perinatal network database	**HIGH** Municipality town hall not woman's home address	**LOW** Objective outcomes (SB, PM, BBA)	**LOW** All births identified included in the analysis	**LOW** Method described; hierarchical LR and multilevel LR reported	**LOW** Adjusted for maternal age, urbanization level and other characteristics

70

For peer review only - http://bmjopen.bmj.com/site/about/guidelines.xhtml

Renesme 2013 (35), France	Case definition	**YES** linked to perinatal network database	**LOW** Distance & travel time estimated using GIS	**LOW** Objective outcomes retrieved from regional and hospital databases	**LOW** 5/81 (6%) BBAs missing, 3/162 (2%) controls missing	**LOW** Method described and univariate and appropriate; multivariate reported	**LOW** Adjusted for age, family status, INSEE maternal occupation, parity, and other characteristics
	Representativeness of cases	**YES** All cases in defined period					
	Appropriate selection of controls	**YES** Controls chosen randomly from same databases and from births occurring at the nearest delivery date and hour to cases					
	Definition of control appropriate	**YES** Outcome could not have occurred					
	Comparability of cases and controls	**NO** Difference in antenatal care attendance					
Nguyen 2016 (34), France	Case definition	**YES** Using medical records	**UNCLEAR** No information	**LOW** Objective outcome (BBA)	**UNCLEAR** No information	**HIGH** No details of the analysis method and analysis was only descriptive	**HIGH** No adjustment for any potential confounders
	Representativeness of cases	**YES** All cases in defined period					

For peer review only - http://bmjopen.bmj.com/site/about/guidelines.xhtml

	Appropriate selection of controls	**YES** Next birth, of equivalent GA					
	Definition of control appropriate	**YES** Outcome could not have occurred					
	Comparability of cases and controls	**NO** Significant differences in parity, smoking, pregnancy monitoring, profession					
Ravelli 2011 (36-38), Netherlands (abstract & full papers)	**LOW** Population based study using the Netherlands Perinatal Registry		**LOW** GIS software used to measure travel time from women's postcodes	**LOW** Objective outcomes from perinatal registry	**LOW** Small proportion (0.3%) of women excluded due to incorrect zip code	**LOW** Method reported; descriptive analysis & LR results given	**LOW** Analysis adjusted for age, parity, ethnicity, SES
Ravelli 2012 (39), Netherlands	**LOW** Population based study using the Netherlands Perinatal Registry		**LOW** GIS software used to measure travel time from women's postcodes	**LOW** Objective outcomes from perinatal registry	**LOW** Small proportion 4% of women excluded	**UNCLEAR** No information	**LOW** Adjusted for age, parity, very urban /very rural, SES
Stolp 2015 (40), Netherlands	**HIGH** Study participants were selected by midwives		**LOW** Ambulance interval includes total time from dispatch call to arrival at hospital	**UNCLEAR** Method of measuring blood loss not reported	**HIGH** Missing data 18/72 (25%) due to incomplete documentation	**HIGH** Data only analysed descriptively	**HIGH** No adjusted analysis

For peer review only - http://bmjopen.bmj.com/site/about/guidelines.xhtml

Engjom 2017 & Engjom 2015 (41, 42), Norway (abstract & full paper)	**LOW** Medical Birth Registry of Norway	**LOW** Travel time polygon from home address using GIS	**LOW** Objective outcomes from birth registry	**UNCLEAR** No information	**LOW** Analysis appropriate, details of LR, multilevel modelling were reported	**LOW** Adjusted for maternal age, parity, education, ethnicity
Grzybowski 2011 (44), Canada	**LOW** Population based study using British Columbia Perinatal Health Program	**HIGH** GIS used to create one hour travel zone for each maternity service, but central postal code to the nearest maternity care used	**LOW** Objective outcomes from Perinatal Health Program	**LOW** 0.3% excluded due to incorrect zip code	**LOW** Analysis appropriate, descriptive analysis & hierarchical LR reported	**LOW** Adjusted for maternal age, parity, SES, ethnicity
Grzybowski 2013 (45), Canada	**LOW** Data from Perinatal Data Registry	**HIGH** Community central postal code used not women's home address	**LOW** Objective outcomes from Perinatal Data Registry	**HIGH** Number of women excluded due to incorrect postal address not reported	**LOW** Analysis appropriate descriptive analysis & LR	**LOW** Adjusted for maternal age, parity, lone parent status, ethnicity, SES
Grzybowski 2015 (46), Canada	**LOW** Provincial perinatal registries	**HIGH** Community central point postal code used not women's home address	**LOW** Objective outcomes from Perinatal Data Registries	**UNCLEAR** No information on missing data	**LOW** Analysis appropriate, descriptive analysis & LR reported	**LOW** Adjusted for maternal age, parity

For peer review only - http://bmjopen.bmj.com/site/about/guidelines.xhtml

Stoll 2014 (47), Canada	**LOW** Based on British Columbia Perinatal Database Registry	**LOW** Used GIS and Google maps; travel times were adjusted for travel conditions	**LOW** Objective outcomes (CS)	**LOW** No missing data	**HIGH** Data were only analysed descriptively	**HIGH** No adjusted analysis
Darling 2019 (43), Canada	**LOW** Data from Perinatal Registries	**LOW** Driving time from women's residence using online mapping tool ArcGIS	**LOW** Objective outcomes from Perinatal Data Registries	**LOW** 3.7% excluded not being able to calculate distance to nearest hospital	**LOW** Method reported, results of descriptive analysis & LR reported	**LOW** Adjusted for maternal age, parity, gestational age, season, SES
Aoshima 2011 (48), Japan	**LOW** All Japan except for isolated islands outside road network (96.6% of all Medical Service Areas)	**HIGH** Used central point of municipality not home address but analysis based on (larger) Medical Service Areas.	**LOW** Objective outcomes from Medical Service Area databases	**UNCLEAR** No information on missing data	**LOW** Method appropriate, unpaired t-test, difference-in-difference analysis	**HIGH** No adjusted analysis

Abbreviations: BBA: Born Before Arrival; CS: caesarean section; GA gestational age; GIS: Geographical Information System; INSEE: Institute National de la Statistique et des Etudes Economiques; LR: Logistic regression; NM: Neonatal Mortality; NNU: Neonatal Unit; PM: Perinatal Mortality; SES: socioeconomic status; SB: Still Birth.

For peer review only

For peer review only - http://bmjopen.bmj.com/site/about/guidelines.xhtml

Table C3: Outcomes – Travel time

Outcomes	Study, Year, Country	Exposure groups	Participants (N, n, %)			Findings		
**MATERNAL OUTCOMES**								
**Maternal mortality (MM)**	No studies							
**Caesarean section (CS) (overall or intrapartum)**	Grzybowski 2011 (44), Canada	**Travel time (hrs):**<br>**<1**<br>1-2<br>2-4<br>>4				**All CS**		
			**Groups**	**N (35,429)**	**All CS n (%)**	**Groups**	**Crude OR (95%CI)**	**Adjusted OR (95%CI)**
			<1	32814	*8597* (26.2)	<1	1	NR
			1-2	1359	*313* (23)	1-2	*0.84 (0.74, 0.96)*	NR
			2-4	747	*156* (20.9)	2-4	*0.74 (0.62, 0.89)*	NR
			>4	509	*97* (19.06)	>4	*0.66 (0.53, 0.83)*	NR
	Grzybowski 2013 (45), Canada	**Travel time (hrs):**<br><1<br>>1				**All CS**		
			**Groups**	**N (59,386)**	***n* (%)**	**Groups**	**Crude OR (95%CI)**	**Adjusted OR (95%CI)**
			<1	54 714	*14882* (27.20)	<1	1	NR
			>1	4672	*1075* (23.01)	>1	*0.80 (0.75, 0.86)*	NR

For peer review only - http://bmjopen.bmj.com/site/about/guidelines.xhtml

	Grzybowski 2015 (46), Canada	**Travel time (hrs):**<br><1<br>1-2<br>2-4<br>>4				**Alberta CS**		
			**Groups**	**Alberta N (34,453)**	**n%**	**Groups**	**Crude OR (95%CI)**	**Adjusted OR (95%CI)**
			<1 hr	29906	NR	<1	NR	1
			1-2	2940	NR	1-2	NR	0.86 (0.78, 0.94)
			2-4	1297	NR	2-4	NR	0.67 (0.58, 0.77)
			>4	310	NR	>4	NR	0.64 (0.48, 0.87)
						**British Columbia CS**		
			**Groups**	**British Columbia N (42,217)**	**n%**	**Groups**	**Crude OR (95%CI)**	**Adjusted OR (95%CI)**
			<1 hr	39,101	NR	<1	NR	1
			1-2	1892	NR	1-2	NR	0.92 (0.83, 1.03)
			2-4	623	NR	2-4	NR	0.74 (0.61, 0.90)
			>4	601	NR	>4	NR	0.70 (0.57, 0.85)
						**Nova Scotia CS**		
			**Groups**	**Nova Scotia N (17,336)**	**n%**	**Groups**	**Crude OR (95%CI)**	**Adjusted OR (95%CI)**
			<1 hr	15465	NR	<1	NR	1
			1-2	1772	NR	1-2	NR	0.87 (0.77, 0.98)

For peer review only

			2-4	99	NR	2-4	NR	0.67 (0.40, 1.10)
						>4	NR	-
	Stoll 2014 (47), Canada	**Travel time (hrs):**<br><1<br>1-2<br>>2	**Groups**	**N (3,692)**	**n (%)**	**Groups**	**Crude OR (95%CI)**	**Adjusted OR (95%CI)**
			<1	3438	633 (18.41)	<1	1	NR
			1-2	124	27 (21.80)	1-2	*1.23 (0.80, 1.91)*	NR
			>2	130	26 (20.0)	>2	*1.11 (0.71, 1.72)*	NR
	Darling 2019 (43), Canada	**Travel time (mins):**<br>≤ 30<br>>30	**Groups**	**N**	**n (%)**	**Groups**	***Crude OR (95%CI)***	**Adjusted OR (95%CI)**
			≤ 30	9189	536 (5.83)	≤ 30	*1*	NR
			>30	2236	98 (4.44)	>30	*0.74 (0.59, 0.92)*	NR
**Emergency CS**	Grzybowski 2013 (45), Canada	**Travel time (hrs):**<br><1<br>>1	**Groups**	**N (59,386)**	**n (%)**	**Groups**	***Crude OR (95%CI)***	**Adjusted OR (95%CI)**
			<1	54,714	9247 (16.99)	<1	*1*	NR
			>1	4672	701 (15.00)	>1	*0.80 (0.75, 0.86)*	NR
**Severe perineal trauma (3rd or 4th degree tear)**	No studies							
**Postpartum haemorrhage**	Stolp 2015 (40),	**Travel time (mins):**	**Groups**	**N (54)**	**n (%)**	**Groups**	**Median (range) ml**	

For peer review only - http://bmjopen.bmj.com/site/about/guidelines.xhtml

	Netherlands	<45<br>>45	<45	34	NR	<45	2,000 (1,100–7,000)	
			>45	20	NR	>45	2,050 (1,000–6,000) (P=0.9)	
	Grzybowski 2013 (45), Canada	**Travel time (hrs):**<br><1<br>>1	**Groups**	**N (59,386)**	**n (%)**	**Groups**	**Crude OR (95%CI)**	
			<1	54 714	*3064* (5.6)	<1	1	
			>1	4672	*327* (7.0)	>1	*1.27 (1.13, 1.43)*	
**Maternal admission to intensive care unit**	Stolp 2015 (40), Netherlands	**Travel time (mins):**<br><45<br>>45	**Groups**	**N (54)**	**n (%)**	**Groups**	**Crude OR (95%CI)**	**Adjusted OR (95%CI)**
			≤45	34	1 *(2.94)*	≤45	1	NR
			>45	20	1 *(5.0)*	>45	*1.74 (0.10, 29.39)*	NR
**Maternal blood transfusion**	Stolp 2015 (40), Netherlands	**Travel time (mins):**<br><45<br>>45	**Groups**	**N (54)**	**n (%)**	**Groups**	**Median (range) L**	
			≤45	34	≤45	≤45	0 (0-8)	
			>45	20	>45	>45	2 (0-8)	
**NEONATAL OUTCOMES**								
**Stillbirth (SB) (overall or intrapartum)**	Paranjothy 2014 (32), UK	**Every 15 min increase in travel time** (continuous variable)	**Groups**	**N (412,827)**	**SB n (%)**	**Groups**	**Crude OR (95%CI)**	**Adjusted OR (95%CI)**
			All women	412,827	135 (0.03)	All women	1.29 (1.14, 1.47)	1.13 (0.98, 1.30)

For peer review only - http://bmjopen.bmj.com/site/about/guidelines.xhtml

			Term births in hospital	387,429	85 (*0.02*)	Term births only	1.35 (1.16, 1.57)	1.36 (1.17, 1.59)
			Nullips births in hospital	185,419	69 (*0.04*)	Nullips only	1.33 (1.13, 1.57)	1.21 (1.02, 1.44)
	Combier 2013 (33), France	**Travel time (mins):**<br>≤ 15<br>16-30<br>31-45<br>≥46	**Groups**	**N (111,001)**	**SB n (%)**	**Groups**	**Crude OR (95%CI)**	**Adjusted OR (95%CI)**
			≤ 15	70,427	333 (0.47)	≤ 15	1	1
			16-30	31,792	148 (0.47)	16-30	0.98 (0.81, 1.20)	1.16 (0.96, 1.40)
			31-45	8445	50 (0.59)	31-45	1.25 (0.93, 1.69)	1.31 (0.89, 1.93)
			≥46	337	3 (0.89)	≥46	1.89 (0.60, 5.92)	1.90 (0.70, 5.15)
	Grzybowski 2013 (45), Canada	**Travel time (hrs):**<br><1<br>>1	**Groups**	**N (59,386)**	**SB n (Rate/1000)**	**Groups**	**Crude OR (95%CI)**	**Adjusted OR (95%%CI)**
			<1	54,714	*274* (5.0)	<1	1	1
			>1	4672	*28* (6.0)	>1	*1.20 (0.81, 1.77)*	NR
**Neonatal mortality (NM)**	Dummer 2004 (30), UK	**Travel time (mins):**<br>≤ 17<br>17-35<br>>35	**Groups**	**N (28,7993)**	**Early NM (0-6 days) n (%)**	**Early NM (0-6 days) Groups**	**Crude OR (95%CI)**	**Adjusted OR (95%CI)**
			≤ 17	NR	1850 (NR)	≤ 17	NR	1
			17-35	NR	789 (NR)	17-35	NR	0.97 (0.89, 1.06)
			>35	NR	196 (NR)	>35	NR	0.95 (0.81,1.1)

For peer review only - http://bmjopen.bmj.com/site/about/guidelines.xhtml

			**Groups**	**N (28,7993)**	**NM (0-27days) n (%)**	**NM (0-27 days) Groups**	**Crude OR (95%CI)**	**Adjusted OR (95%CI)**
			≤ 17	NR	1854 (NR)	≤ 17	NR	1
			17-35	NR	946 (NR)	17-35	NR	0.96 (0.89, 10.4)
			>35	NR	239 (NR)	>35	NR	0.95 (0.83, 1.09)
			Groups	**N (28,7993)**	**Post NM (28-1yr) n (%)**	**Post NM (28 days – 1yr) Groups**	**Crude OR (95%CI)**	**Adjusted OR (95%CI)**
			≤ 17	NR	961 (NR)	≤ 17	NR	1
			17-35	NR	400 (NR)	17-35	NR	0.97 (0.86,10.9)
			>35	NR	98 (NR)	>35	NR	0.95 (0.77, 1.17)
	Paranjothy 2014 (32), UK	**Every 15 min increase in travel time** (continuous variable)	**Groups**	**N**	**Early NM n (%)**	**Early NM (0-6 days) Groups**	**Crude OR (95%CI)**	**Adjusted OR (95%CI)**
			All women	412,827	609 *(0.15)*	All women	1.37 (1.31, 1.45)	1.13 (1.07, 1.20)
			Term births only	387,429	177 *(0.05)*	Term births only	1.02 (0.86, 1.21)	0.97 (0.80, 1.17)
			Nullips only	185,419	303 *(0.16)*	Nullips only	1.42 (1.33, 1.51)	1.15 (1.06, 1.25)
			**Groups**	**N**	**Late NM n (%)**	**Late NM (7-27 days) Groups**	**Crude OR (95%CI)**	**Adjusted OR (95%CI)**
			All women	412,827	251 *(0.06)*	All women	1.33 (1.23, 1.44)	1.15 (1.05, 1.26)

For peer review only - http://bmjopen.bmj.com/site/about/guidelines.xhtml

			Term births only	387,429	77 *(0.02)*	Term births only	1.24 (1.03, 1.50)	1.34 (1.13, 1.59)
			Nullips only	185,419	116 *(0.06)*	Nullips only	1.31 (1.15, 1.49)	1.11 (0.97, 1.28)
	Ravelli 2011 (36-38), Netherlands	**Travel time (mins):**<br>< 20 mins<br>≥20 mins	**NM (Combined intrapartum & early neonatal mortality)**					
			**Groups**	**N (1,054,342)**	**NM n (%)**	**Groups**	**Crude OR (95%CI)**	**Adjusted OR (95%CI)**
			< 20	558,181	789 *(0.14)*	< 20 mins	1	1
			≥20	193,745	336 *(0.17)*	≥20 mins	*1.23 (1.08, 1.39)*	1.23 (1.07, 1.41)
			**Low risk women**	**N (120,896)**	**NM n(/1000) 63 (0.05/1000)**	**Low risk women**	**Crude OR (95%CI)**	**Adjusted OR (95%CI)**
			< 20	**NR**	**NR**	< 20	NR	1
			≥20	**NR**	**NR**	≥20	NR	0.8 (0.4, 1.7)
			**Low risk women became high risk during labour**	**N (142,824)**	**NM n(/1000) 1.9/1000**	**Low risk women became high risk during labour**	**Crude OR (95%CI)**	**Adjusted OR (95%CI)**
			<20	NR	NR	<20	NR	1
			≥20	NR	NR	≥20	NR	1.23 (1.04, 1.47)
		**Travel time (mins):**<br><15<br>15-19				**NM (Combined intrapartum & early & late NM up to 28 days)**		
			**Groups**	**N (751,926)**	**NM n (1125) (%)**	**Groups**	**Crude OR (95%CI)**	**Adjusted OR (95%CI)**

For peer review only - http://bmjopen.bmj.com/site/about/guidelines.xhtml

		≥20	<15	425,952	NR	<15	1	1
			15-19	132,229	NR	15-19	0.97 (0.82, 1.15)	0.94 (0.79, 1.12)
			≥20	193,745	336	≥ 20	1.22 (1.07, 1.39)	1.17 (1.02, 1.36)
						**Neonatal mortality within 24 hrs**		
			**Groups**	**N (751,926)**	**NM within 24 hrs n (255) (%)**	**Groups**	**Crude OR (95%CI)**	**Adjusted OR (95%CI)**
			<20	558,181	NR	<20	1	1
			≥20	193,745	NR	≥ 20	1.52 (1.17, 1.97)	1.51 (1.13, 2.02)
						**Neonatal mortality 0-7 days**		
			**Groups**	**N (751,926)**	**NM 0-7 dys (523) (%)**	**Groups**	**Crude OR (95%CI)**	**Adjusted OR (95%CI)**
			<20	558,181	NR	<20	1	1
			≥20	193,745	NR	≥ 20	1.44 (1.20, 1.72)	1.37 (1.12, 1.67)
						**Neonatal mortality 8-27 days**		
			**Groups**	**N (751,926)**	**NM 8-27 dys (58) (%)**	**Groups**	**Crude OR (95%CI)**	**Adjusted OR(95%CI)**
			<20	558,181	NR	<20	1	1
			≥20	193,745	NR	≥ 20	1.30 (0.74, 2.26)	1.24 (0.67, 2.27)
	Grzybowski 2013 (45),	**Travel time**	**Groups**	**N (59,386)**	**NM n (%)**	Late neonatal mortality age <1 month, no events		

For peer review only - http://bmjopen.bmj.com/site/about/guidelines.xhtml

	Canada	**(hrs):**<br><1<br>>1	<1	54,714	0			
			>1	4672	0			
	Aoshima 2011 (48), Japan	**Travel time (mins):**<br>Median 39.09 (2006)<br>Median 66.99 (2002)	**Groups**	**N**	**NM n (Rate/ 1000)**	**Groups**	**Crude OR (95%CI)**	**Adjusted OR (95%CI)**
			2006	NR	NR (1.28)	39.09	NR	NR
			2002	NR	NR (1.67)	66.99	NR	NR
**Perinatal mortality (PM)**	Combier 2013 (33), France	**Travel time (mins):**<br>≤15<br>16 -30<br>31-45<br>≥46	**Groups**	**N (110,664)**	**PM n (%)**	**Groups**	Crude OR (95%CI)	**Adjusted OR (95%CI)**
			≤15	70,427	452 (0.64)	≤15	1	1
			16-30	31,792	195 (0.61)	16-30	0.96 (0.81, 1.13)	1.08 (0.90, 1.29)
			31-45	8445	59 (0.7.0)	31-45	1.09 (0.83, 1.43)	1.18 (0.86, 1.62)
			≥46	337	4 (1.19)	≥46	1.86 (0.69, 5.01)	1.85 (0.66, 5.19)
	Ravelli 2012 (39)	**Travel time (mins):**<br><20<br>≥20	**Groups**	**N (1,242,725)**	**PM n (Rate/1000)**	**Groups**	**Crude OR (95%CI)**	**Adjusted OR (95%CI)**
			<20	*1,006,607*	81 (0.08)	<20	1	1
			≥20	*236,118*	19 (0.08)	≥ 20	1.53 (1.47,1.50)	1.66 (1.59,1.74)
	Grzybowski 2011(44), Canada	**Travel time (hrs):**<br><1<br>1-2<br>2-4<br>>4	**Groups**	**N (35,429)**	**PM n (Rate/1000)**	**Groups**	**Crude OR (95%CI)**	**Adjusted OR (95%CI)**
			<1	32,814	*197* (6.0)	<1	1	1
			1-2	1359	*8* (6.0)	1-2	0.98 (0.48, 1.99)	1.04 (0.48, 2.22)

For peer review only - http://bmjopen.bmj.com/site/about/guidelines.xhtml

			2-4	747	*4* (5.0)	2-4	0.89 (0.33, 2.40)	0.92 (0.33, 2.53)
			>4	509	*9* (18.0)	>4	2.98 (1.52, 5.85)	3.17 (1.45, 6.95)
			PM (stillbirths & early neonatal mortality)					
	Grzybowski 2013 (45), Canada	**Travel time (hrs):**<br><1<br>>1	**Groups**	**N (59,386)**	**PM n (Rate/1000)**	**Groups**	**Crude OR (95%CI)**	**Adjusted (OR 95%CI)**
			< 1	54 714	*383* (7.0)	<1	1	NR
			>1	4672	*37* (8.0)	>1	*1.54 (1.09, 2.17)*	NR
	Grzybowski 2015 (46), Canada	**Travel time (hrs):**<br><1<br>1-2<br>2-4<br>>4				**PM (stillbirths & early neonatal mortality) Alberta**		
			**Groups**	**Alberta (N=34,453)**	**PM n (%)**	**Groups**	**Crude OR (95%CI)**	**Adjusted OR (95%CI)**
			<1 hr	29,906	NR	<1	1	1
			1-2	2940	NR	1-2	NR	1.50 (1.03, 2.18)
			2-4	1297	NR	2-4	NR	1.35 (0.77, 2.38)
			>4	310	NR	>4	NR	1.40 (0.44, 4.39)
						**PM (stillbirths & early neonatal mortality) BC**		
			**Groups**	**British Columbia (N=42,317)**	**PM n (%)**	**Groups**	**Crude OR (95%CI)**	**Adjusted OR (95%CI)**
			<1 hr	39,101	NR	<1	1	1

For peer review only - http://bmjopen.bmj.com/site/about/guidelines.xhtml

			1-2	1892	NR	1-2	NR	0.79 (0.43, 1.45)
			2-4	623	NR	2-4	NR	1.33 (0.59, 3.01)
			>4	601	NR	>4	NR	2.84 (1.58, 5.10)
						**PM (stillbirths & early neonatal mortality) Nova Scotia**		
			**Groups**	**Nova Scotia (N= 17,336)**	**PM n (%)**	**Groups**	**Crude OR (95%CI)**	**Adjusted OR (95%CI)**
			<1 hr	15,465	NR	<1	1	1
			1-2	1772	NR	1-2	NR	0.66 (0.38, 1.14)
			2-4	99	NR	2-4	NR	NR
			>4	0	NR	>4	NR	-
	Stoll 2014 (47), Canada	**Travel time (hrs):**<br><1<br>1-2<br>>2	**Groups**	**N (3,692)**	**PM n (%)**	**Groups**	**Crude OR (95%CI)**	**Adjusted OR (95%CI)**
			<1	3438	15 (0.4)	<1	1	NR
			1-2	124	0	1-2	*0.89 (0.05, 14.91)*	NR
			>2	130	2 (1.5)	>2	*3.57 (0.81, 15.76)*	NR
			PM (Stillbirth and early neonatal death up to 7 days)					
	Darling 2019 (43), Canada	**Travel time (mins):**<br>≤ 30<br>>30	**Groups**	**N (10,681)**	**PM n (%)**	**Groups**	***Crude OR (95%CI)***	**Adjusted OR (95%CI)**
			≤ 30	NR	NR	≤ 30	NR	NR

For peer review only - http://bmjopen.bmj.com/site/about/guidelines.xhtml

			>30	NR	NR	>30	NR	NR<br>Reported as RR<br>2.2 (0.67, 7.43)
**Infant mortality (IM)**	Grzybowski 2013 (45), Canada	*Travel time (hrs):*<br><1<br>>1				**IM (age 1-12 month)**		
			**Groups**	**N (59,386)**	**IM n(Rate/1000)**	**Groups**	**Crude OR (95%CI)**	**Adjusted OR (95%CI)**
			<1	54,714	*109* (2.0)	<1	1	NR
			>1	4672	*14* (3.0)	>1	*1.51 (0.86, 2.63)*	NR
**Born before arrival (BBA)**	Combier 2013 (33), France	Travel time (mins):<br><15<br>15-29<br>30-44<br>>45	**Groups**	**N (111,001)**	**BBA n (%)**	**Groups**	**Crude OR (95%CI)**	**Adjusted OR (95%CI)**
			<16	70,427	132 (0.19)	<16	1	1
			16-30	31,792	93 (0.29)	16-30	1.56 (1.20, 2.04)	1.73 (1.23, 2.46)
			31-45	8445	29 (0.34)	31-45	1.84 (1.23, 2.75)	1.64 (1.06, 2.54)
			>45	337	0	>45	-	-
	Renesme 2013 (35), France	**Travel time (mins):**<br><15<br>15-29<br>30-44<br>>45	**Groups**	**Cases N (73) (%)**	**Control N(148) (%)**	**Groups**	**Crude OR (95%CI)**	**Adjusted OR(95%CI)**
			<15	22 (30.2)	59 (39.9)	<15	1	1
			15-29	33 (45.2)	64 (43.2)	15-29	*1.79 (0.87, 3.68)*	1.92 (0.86, 4.96)
			30-44	9 (12.3)	18 (12.2)	30-44	*1.68 (0.58, 4.87)*	1.10 (0.35, 3.48)
			>45	9 (12.3)	7 (4.7)	>45	*5.89 (1.12, 30.89)*	6.18 (1.33, 8.65)

For peer review only

For peer review only - http://bmjopen.bmj.com/site/about/guidelines.xhtml

	Nguyen 2016 (34), France	**Travel time (mins):** ≤ 20 > 20	**Groups**	**N (188)**	**BBA n (%)**	**Groups**	**Crude OR (95%CI)**	**Adjusted OR(95%CI)**
			>20	94 controls	22 (23.4)	>20 controls	1	NR
			>20	94 cases	27 (28.7)	>20 cases	*1.3 (0.7, 2.6)*	NR
	Engjom 2017 (42), Norway	**Travel time (hrs):** <1 1-2 >2	**Groups**	**N (646,898)**	**BBA n (%)**	**Groups**	**Crude OR (95%CI)**	**Adjusted OR (95%CI)**
			1	615,896	3488 (0.60)	1	1	1
			1-2	25,494	844 (3.31)	1-2	*6.01 (5.57, 6.49)*	NR reported as RR* 5.3 (5.0,5.8)
			>2	5508	246 (4.50)	>2	*8.21 (7.19, 9.37)*	NR reported as RR* 7.2 (6.3,8.2)
	Grzybowski 2011 (44), Canada	**Travel time (hrs):** <1 1-2 2-4 >4	**Groups**	**N (35,429)**	**BBA n (%)**	**Groups**	**Crude OR (95%CI)**	**Adjusted OR (95%CI)**
			<1	32,814	*66* (0.20)	<1	1	1
			1-2	1359	*31* (2.30)	1-2	*11.58 (7.53, 17.81)*	6.41(3.69,11.28)
			2-4	747	*3* (0.3)	2-4	*2.00 (0.63, 6.38)*	0.92 (0.22, 3.88)
			>4	506	*7* (1.4)	>4	*6.96 (3.18, 15.25)*	3.63 (1.40, 9.40)
	Grzybowski 2013 (45), Canada	**Travel time (hrs):** <1 >1	**Groups**	**N (59,386)**	**BBA n (%)**	**Groups**	**Crude OR (95%CI)**	**Adjusted OR (95%CI)**
			<1	54,714	*164* (0.3)	<1	1	NR
			>1	4672	*70* (1.5)	>1	*5.06 (3.82, 6.70)*	NR
**Combined mortality &**	Ravelli 2011 (36-38),	**Travel time (mins):**	**Groups**	**N(751,926)**	**Event n (4,543) (%)**	**Groups**	**Crude OR (95%CI)**	**Adjusted OR (95%CI)**

For peer review only - http://bmjopen.bmj.com/site/about/guidelines.xhtml

**or Apgar < 4 at 5 mins & or transfer to NICU**	Netherlands	<15<br>15-19<br>>=20	<15	425,952	NR	<15	1	1
			15-20	132,229	NR	15-20	0.99 (091, 1.07)	1.11 (1.02, 1.21)
			>=20	193,745	NR	>=20	1.11 (1.04, 1.19)	1.27 (1.17, 1.38)
**Neonatal Unit admission (NNU)**	Grzybowski 2011 (44), Canada	Travel time (hrs):<br><1<br>1-2<br>2-4<br>>4	**NICU level 2 admissions per 1000 births (2001-2004)**			**NICU 2**		
			**Groups**	**N (35,429)**	**NICU2 n (Rate/1000)**	**Groups**	**Crude OR (95%CI)**	**Adjusted OR (95%CI)**
			<1	32,814	*1082* (33.0)	<1	1	1
			1-2	1359	*69* (51.0)	1-2	*1.57 (1.22, 2.01)*	2.20 (1.59, 3.05)
			2-4	747	*8* (11.0)	2-4	*0.32 (0.16, 0.64)*	0.31 (0.14, 0.65)
			>4	506	*14* (27.0)	>4	*0.83 (0.49, 1.42)*	1.07 (0.54, 2.12)
			**NICU level 3 per 1000 births (2001-2004)**			**NICU 3**		
			**Groups**	**N (34,920)**	**NICU3 n (Rate/1000)**	**Groups**	**Crude OR (95%CI)**	**Adjusted OR (95%CI)**
			<1	32,814	*98* (3.0)	<1	1	NR
			1-2	1359	*11* (8.0)	1-2	*2.72 (1.46, 5.09)*	NR
			2-4	747	*4* (5.0)	2-4	*1.80 (0.66, 4.90)*	NR
			>4	509	*2* (4.0)	>4	*1.32 (0.32, 5.35)*	NR
	Grzybowski 2013 (45),	**Travel time (hrs):**	**NICU level 2 (2001-2 & 2006-7) n=74697**			**NICU 2**		

For peer review only - http://bmjopen.bmj.com/site/about/guidelines.xhtml

	Canada	<1<br>>1	**Groups**	**N**	**NICU2 n (Rate/1000)**	**Groups**	**Crude OR (95%CI)**	**Adjusted OR (95%CI)**
			<1	54,714	*1751* (32.0)	<1	1	NR
			>1	4672	*154* (33.0)	>1	*1.03 (0.87, 1.22)*	NR
			**NICU level 3 (2001-2 & 2006-7) n=74697**			**NICU 3**		
			**Groups**	**N**	**NICU3 n(Rate/1000)**	**Groups**	**Crude OR (95%CI)**	**Adjusted OR (95%CI)**
			<1	54,714	*219* (4.0)	<1	1	NR
			>1	4672	*28* (6.0)	>1	*1.50 (1.01, 2.23)*	NR
			**NICU (2&3)**			**NICU admission**		
			**Groups**	**N (59,386)**	**NICU n (Rate/1000)**	**Groups**	**Crude OR (95%CI)**	**Adjusted OR (95%CI)**
			<1	54,714	*1970 (36.0)*	<1	1	NR
			>1	4672	*182 (39.0)*	>1	*1.09 (0.93, 1.27)*	NR
	Darling 2019 (43), Canada	**Travel time (mins):**<br>≤ 30<br>>30	**Groups**	**N (10,687)**	**NICU n (%)**	**Groups**	**Crude OR (95%CI)**	**Adjusted OR (95%CI)**
			≤ 30	NR	NR	≤ 30	NR	1
			>30	NR	NR	>30	NR	NR<br>Reported as RR*<br>0.6 (0.44, 0.81)
**Apgar <7 at 5 mins**	Darling 2019 (43), Canada	**Travel time (mins):**	**Groups**	**N (10,578)**	**Apgar <7 n (%)**	**Groups**	**Crude RR (95%CI)**	**Adjusted OR (95%CI)**

For peer review only - http://bmjopen.bmj.com/site/about/guidelines.xhtml

		≤ 30<br>>30	≤ 30	NR	NR	≤ 30	NR	1
			>30	NR	NR	>30	NR	NR<br>Reported as RR*<br>1.02 (0.95, 1.10)
			**Nullips**	**N (4208)**	**Apgar <7 n (%)**	**Groups**	**Crude OR (95%CI)**	**Adjusted OR (95%CI)**
			≤ 30	3425	51 (1.5)	≤ 30	1	NR
			>30	621	14 (2.3)	>30	*1.53 (0.84, 2.77)*	NR
			**Mullips**	**N (7661)**	**Apgar <7 n (%)**	**Groups**	**Crude OR (95%CI)**	**Adjusted OR (95%CI)**
			≤ 30	5764	30 (0.5)	≤ 30	*1*	NR
			>30	1615	11 (0.7)	>30	*1.31 (0.66, 2.62)*	NR
**HIE**	No studies reported							

Abbreviations: BW: Birth Weight; NR: not reported; Nullips: Nulliparous; NICU: Neonatal Intensive Care Unit; * RR: relative risk.

For peer review only

References:

1. Jordan H, Roderick P, Martin D, Barnett S. Distance, rurality and the need for care: access to health services in South West England. International Journal of Health Geographics. 2004;3(1):21.
2. Kelly C, Hulme C, Farragher T, Clarke G. Are differences in travel time or distance to healthcare for adults in global north countries associated with an impact on health outcomes? A systematic review. BMJ open. 2016;6(11):e013059-e.
3. Rashidian A, Omidvari AH, Vali Y, Mortaz S, Yousefi-Nooraie R, Jafari M, et al. The effectiveness of regionalization of perinatal care services - a systematic review. Public Health. 2014;128(10):872-85.
4. Price S, Little K. Research evidence review -impact of distance/travel time to maternity services on birth outcomes. Public Health Wales NHS Trust 2015.
5. Blondel BD, Nicolas; Pilkington, Hugo; Zeitlin, Jennifer. Out-of-hospital births and the supply of maternity units in France. Health & Place. 2011;17(5):1170-3.
6. Lasswell SM, Barfield WD, Rochat RW, Blackmon L. Perinatal regionalization for very low-birth-weight and very preterm infants: a meta-analysis. Jama. 2010;304(9):992-1000.
7. Engjom HMM, N. H.; Norheim, O. F.; Klungsoyr, K. Availability and access in modern obstetric care: a retrospective population-based study. BJOG: An International Journal of Obstetrics & Gynaecology. 2014;121(3):290-9.
8. Stroup DF, Berlin JA, Morton SC, Olkin I, Williamson GD, Rennie D, et al. Meta-analysis of Observational Studies in EpidemiologyA Proposal for Reporting. Jama. 2000;283(15):2008-12.
9. Merenstein G, Glicken A. Best Evidence-Based Practices: A Historic Perspective. Neonatal Network. 2002(5):31-5.
10. World Health Organization. Universal health coverage (UHC) 2019 [Available from: https://www.who.int/news-room/fact-sheets/detail/universal-health-coverage-(uhc).
11. Rowe R, The Birthplace in England Collaborative Group, ., McCourt, C. and MacFarlane, A, . Birthplace terms and definitions: consensus process Birthplace in England research programme. (Final Report 2. 08/1604/140). Southampton: HMSO.; 2011.
12. Thomas J, Brunton J, S. G. EPPI-Reviewer 40: software for research synthesis EPPI-Centre Software London: Social Science Research Unit, Institute of Education, University of London. 2010.
13. Cochrane Effective Practice and Organisation of Care (EPOC). Suggested risk of bias cri teria for EPOC reviews. EPOC Resources fo r review authors. Available at: http ://ep occochraneorg/res ources /epoc-res ources-revi ew-authors. 2017.
14. Stang A. Critical evaluation of the Newcastle-Ottawa scale for the assessment of the quality of nonrandomized studies in meta-analyses. Eur J Epidemiol. 2010;25(9):603-5.
15. Fleming AM, E. A.; Schram, C. M. H. Reducing caesarean section rates through choice and collaboration. Archives of Disease in Childhood: Fetal and Neonatal Edition Conference: 16th Annual Conference of the British Maternal and Fetal Medicine Society Dublin Ireland Conference Publication:. 2013;98(no pagination).
16. Mackie FLA, F.; Moise, J.; Amu, O. Maternal and neonatal outcomes after the amalgamation of two maternity units and consequent increased consultant labour ward presence: A retrospective population-based study. Archives of Disease in Childhood: Fetal and Neonatal Edition. 2014;99:A21-A2.
17. East Lancashire hospitals. Unpublished data 2017.
18. Grytten JM, Lars; Skau, Irene; Sorensen, Rune. Regionalization and local hospital closure in Norwegian maternity care--the effect on neonatal and infant mortality. Health Services Research. 2014;49(4):1184-204.
19. Hemminki EH, A.; Gissler, M. Should births be centralised in higher level hospitals? Experiences from regionalised health care in Finland. BJOG: An International Journal of Obstetrics & Gynaecology. 2011;118(10):1186-95.

For peer review only - http://bmjopen.bmj.com/site/about/guidelines.xhtml

20. Allen VM, Jilwah N, Joseph KS, Dodds L, O'Connell CM, Luther ER, et al. The Influence of Hospital Closures in Nova Scotia on Perinatal Outcomes. Journal of Obstetrics and Gynaecology Canada. 2004;26(12):1077-85.
21. Hutcheon J, A.; Riddell Corinne, A.; Strumpf Erin, C.; Lee, Lily; Harper, Sam. Safety of labour and delivery following closures of obstetric services in small community hospitals. CMAJ Canadian Medical Association Journal. 2017;189(11):E431-E6.
22. Le Coutour I-R, C.; Danzon, A. [Regionalization of health care and obstetric practice]. Revue d Epidemiologie et de Sante Publique. 1990;38(3):211-20.
23. Allen V, M.; Jilwah, Nadia; Joseph, K. S.; Dodds, Linda; O'Connell Colleen, M.; Luther Edwin, R.; Fahey, T. John; Attenborough, Rebecca; Allen Alexander, C. The influence of hospital closures in Nova Scotia on perinatal outcomes. Journal of Obstetrics & Gynaecology Canada: JOGC. 2004;26(12):1077-85.
24. Bhoopalam PS, Watkinson M. Babies born before arrival at hospital. BJOG: An International Journal of Obstetrics & Gynaecology. 1991;98(1):57-64.
25. Pasquier JCM, M.; Bagouet, S.; Moret, S.; Luo, Z. C.; Rabilloud, M.; Gaucherand, P.; Robert-Gnansia, E. Effects of residential distance to hospitals with neonatal surgery care on prenatal management and outcome of pregnancies with severe fetal malformations. Ultrasound in Obstetrics & Gynecology. 2007;29(3):271-5.
26. Pilkington HB, Beatrice; Drewniak, Nicolas; Zeitlin, Jennifer. Where does distance matter? Distance to the closest maternity unit and risk of foetal and neonatal mortality in France. European Journal of Public Health. 2014;24(6):905-10.
27. Fougner BN, J. [Deliveries during transportation and shortly after admission to hospital]. Tidsskrift for Den Norske Laegeforening. 2000;120(11):1288-90.
28. Ovaskainen KO, Riitta; Gissler, Mika; Luukkaala, Tiina; Tammela, Outi. Out-of-hospital deliveries have risen involving greater neonatal morbidity: Risk factors in out-of-hospital deliveries in one University Hospital region in Finland. Acta Paediatrica. 2015;104(12):1248-52.
29. Lisonkova S, Sheps SB, Janssen PA, Lee SK, Dahlgren L, MacNab YC. Birth Outcomes Among Older Mothers in Rural Versus Urban Areas: A Residence-Based Approach. The Journal of Rural Health. 2011;27(2):211-9.
30. Dummer TJBP, L. Hospital accessibility and infant death risk. Archives of Disease in Childhood. 2004;89(3):232-4.
31. Paranjothy SW, W. J.; Rolfe, K.; Gong, Y.; Adappa, R.; Dunstan, F.; Kotecha, S. Perinatal outcomes and travel time to maternity services: Analysis of birth outcome data in Wales from 1995 to 2009. Archives of Disease in Childhood: Fetal and Neonatal Edition Conference: 16th Annual Conference of the British Maternal and Fetal Medicine Society Dublin Ireland Conference Publication:. 2013;98(no pagination).
32. Paranjothy SW, W. John; Rolfe, Kim; Adappa, Roshan; Gong, Yi; Dunstan, Frank; Kotecha, Sailesh. Perinatal outcomes and travel time from home to hospital: Welsh data from 1995 to 2009. Acta Paediatrica. 2014;103(12):e522-7.
33. Combier EC, Helene; Le, Vaillant; Marc,; Michaut, Francis; Ferdynus, Cyril; Amat-Roze, Jeanne-Marie; Gouyon, Jean-Bernard; Quantin, Catherine; Zeitlin, Jennifer. Perinatal health inequalities and accessibility of maternity services in a rural French region: closing maternity units in Burgundy. Health & Place. 2013;24:225-33.
34. Nguyen MLL, P.; Dreyfus, M. [Maternal and neonatal outcomes of unplanned deliveries]. Journal de Gynecologie, Obstetrique et Biologie de la Reproduction. 2016;45(1):86-91.
35. Renesme L, Garlantézec R, Anouilh F, Bertschy F, Carpentier M, Sizun J. Accidental out-of-hospital deliveries: a case–control study. Acta Paediatrica. 2013;102(4):e174-e7.
36. Ravelli ACJJ, K. J.; De, Groot; M, H.; Rijninks-Van, Driel; G, C.; Tromp, M.; Eskes, M.; Willem, B.; Mol, J.; Abu-Hanna, A.; Jaap, J.; Erwich, H. M. Travel time from home to hospital and intrapartum and neonatal mortality in term pregnant women. Journal of Maternal-Fetal and Neonatal Medicine. 2010;23:234.

For peer review only - http://bmjopen.bmj.com/site/about/guidelines.xhtml

37. Ravelli ACJJ, K. J.; De, Groot; M, H.; Erwich, J. J. H. M.; Rijninks-Van, Driel; G, C.; Tromp, M.; Eskes, M.; Abu-Hanna, A.; Mol, B. W. J. Travel time from home to hospital and adverse perinatal outcomes in women at term in the Netherlands. Obstetrical and Gynecological Survey. 2011;66(7):396-8.
38. Ravelli ACJJ, K. J.; de, Groot; M, H.; Erwich, J. J. H. M.; Rijninks-van, Driel; G, C.; Tromp, M.; Eskes, M.; Abu-Hanna, A.; Mol, B. W. J. Travel time from home to hospital and adverse perinatal outcomes in women at term in the Netherlands. BJOG: An International Journal of Obstetrics & Gynaecology. 2011;118(4):457-65.
39. Ravelli AEM, Erwich JJHM, Brouwers HAA, Abu-Hanna A, Kerkhof FGM, van der Post JAM, H.O.PC., Neoned* & B.W.J. Mol. Perinatale sterfteverschillen in de negen perinatale zorgregio's Nederlands tijdschrift voor Obstetrie & Gynaecologie 2012 125, (augustus 2012):270-7.
40. Stolp IS, Marrit; Luxemburg, Sanne; Akker, Thomas; Waard, Jan; Roosmalen, Jos; Vos, Rien. Ambulance Transfer in Case of Postpartum Hemorrhage after Birth in Primary Midwifery Care in The Netherlands: A Prospective Cohort Study. Birth: Issues in Perinatal Care. 2015;42(3):227-34.
41. Engjom HM, N. H.; Hoydal, E.; Norheim, O. F.; Klungsoyr, K. Obstetric health system structure and perinatal outcomes in Norway. International Journal of Gynecology and Obstetrics. 2015;131:E487-E8.
42. Engjom HM, Morken N-H, Høydahl E, Norheim OF, Klungsøyr K. Increased risk of peripartum perinatal mortality in unplanned births outside an institution: a retrospective population-based study. American Journal of Obstetrics and Gynecology. 2017;217(2):210.e1-.e12.
43. Darling EK, Lawford KMO, Wilson K, Kryzanauskas M, Bourgeault IL. Distance from Home Birth to Emergency Obstetric Services and Neonatal Outcomes: A Cohort Study. Journal of Midwifery & Women's Health. 2019;64(2):170-8.
44. Grzybowski SS, Kathrin; Kornelsen, Jude. Distance matters: a population based study examining access to maternity services for rural women. BMC Health Services Research. 2011;11:147.
45. Grzybowski SS, Kathrin; Kornelsen, Jude. The outcomes of perinatal surgical services in rural British Columbia: a population-based study. Canadian Journal of Rural Medicine. 2013;18(4):123-9.
46. Grzybowski SF, John; Lai, Barbara; Zhang, Sharon; Aelicks, Nancy; Leung Brenda, M.; Stoll, Kathrin; Attenborough, Rebecca. The safety of Canadian rural maternity services: a multi-jurisdictional cohort analysis. BMC Health Services Research. 2015;15:410.
47. Stoll KK, Jude. Midwifery Care in Rural and Remote British Columbia: A Retrospective Cohort Study of Perinatal Outcomes of Rural Parturient Women With a Midwife Involved in Their Care, 2003 to 2008. Journal of Midwifery & Women's Health. 2014;59(1):60-6.
48. Aoshima KK, H.; Kawahara, K. Neonatal mortality rate reduction by improving geographic accessibility to perinatal care centers in Japan. Journal of Medical and Dental Sciences. 2011;58(2):29-40.
49. Pilkington HP, Caroline; Blondel, Beatrice; Charreire, Helene; Combier, Evelyne; Le, Vaillant; Marc,; Amat-Roze, Jeanne-Marie; Zeitlin, Jennifer. Travel Time to Hospital for Childbirth: Comparing Calculated Versus Reported Travel Times in France. Maternal & Child Health Journal. 2017;05:05.

For peer review only - http://bmjopen.bmj.com/site/about/guidelines.xhtml

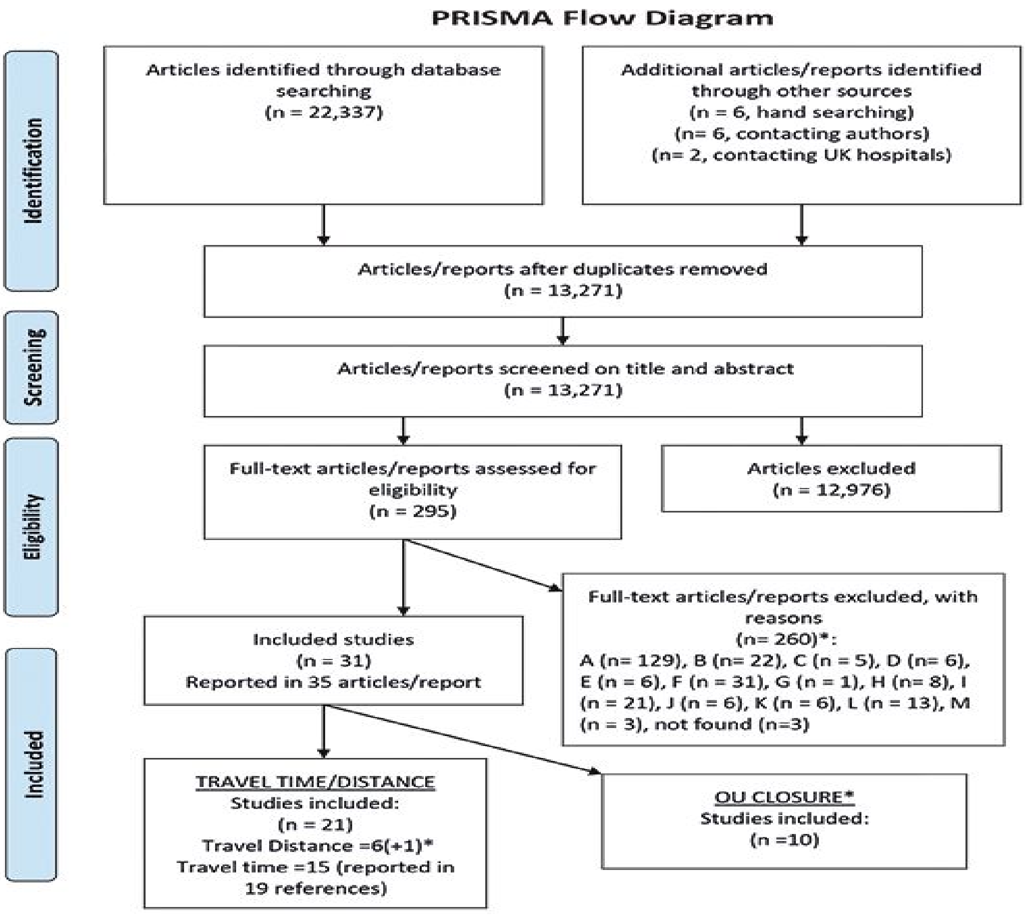

*One study, Blondel 2011 contribute data to both travel distance and closure review questions

From: Moher D, Liberati A, Tetzlaff J, Altman DG, The PRISMA Group (2009). *Preferred Reporting Items for Systematic Reviews and Meta-Analyses*: The PRISMA Statement. PLoS Med 6(7): e1000097. doi:10.1371/journal.pmed1000097

For more information, visit www.prisma-statement.org.

Reasons for inclusion:

A. Different study designs
B. Studies comparing different models of maternity care
C. Studies comparing different hospital volumes
D. Studies comparing different levels of care
E. Studies comparing different hospital types (private vs. public, local vs. central)
F. Transfer from primary to secondary birth centres
G. Cost of care
H. Neonatal transfer
I. Regionalisation
J. Not in high-income countries or in the USA
K. Distance or travel time proximity
L. No outcomes
M. Modelling studies
N. Not found

PRISMA Flow Diagram

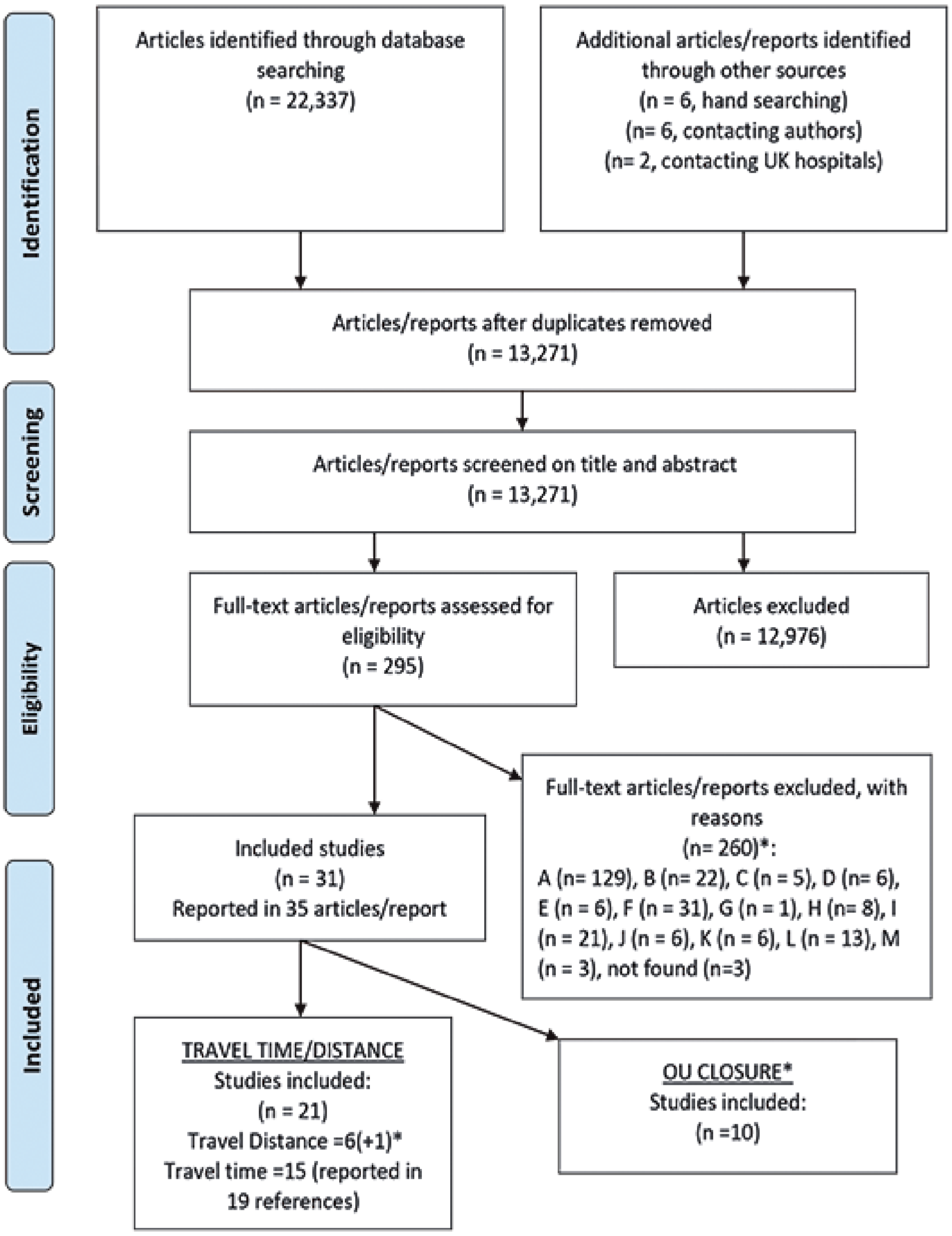

*One study, Blondel 2011 contribute data to both travel distance and closure review questions

From: Moher D, Liberati A, Tetzlaff J, Altman DG, The PRISMA Group (2009). *P*referred *R*eporting *I*tems for *S*ystematic Reviews and *M*eta-*A*nalyses: The PRISMA Statement. PLoS Med 6(7): e1000097. doi:10.1371/journal.pmed1000097

For more information, visit www.prisma-statement.org.

Reasons for inclusion:

A. Different study designs
B. Studies comparing different models of maternity care
C. Studies comparing different hospital volumes
D. Studies comparing different levels of care
E. Studies comparing different hospital types (private vs. public, local vs. central)
F. Transfer from primary to secondary birth centres
G. Cost of care
H. Neonatal transfer
I. Regionalisation
J. Not in high-income countries or in the USA
K. Distance or travel time proximity
L. No outcomes
M. Modelling studies
N. Not found

Appendix 1: Current high-income countries (OECD) members

- Australia
- Austria
- Belgium
- Canada
- Chile
- Czech Republic
- Denmark
- Estonia
- Finland
- France
- Germany
- Greece
- Hungary
- Iceland
- Ireland
- Israël
- Italy
- Japan
- Korea
- Latvia
- Luxembourg
- Mexico
- Netherlands
- New Zealand
- Norway
- Poland
- Portugal
- Slovak Republic
- Slovenia
- Spain

For peer review only

- Sweden
- Switzerland
- Turkey
- United Kingdom
- United States

http://www.oecd.org/about/membersandpartners/

For peer review only

Appendix 2: MEDLINE search results in October 2017

1	Maternal Health Services/	12707
2	Delivery Rooms/	1417
3	Intensive Care Units, Neonatal/ and Intensive Care, Neonatal/	860
4	exp Hospitals/ and (exp Pregnancy/ or exp Delivery, Obstetric/ or Prenatal Care/ or Perinatal Care/ or Postnatal Care/)	10061
5	exp Hospitals/ and (foetal or fetal or foetus or fetus or newborn* or neonat* or infant* or baby or babies or maternal or maternity or pregnant or pregnancy or pregnancies or prenatal or pre-natal or antenatal or ante-natal or perinatal or peri-natal or postnatal or post-natal).ti,ab.	13308
6	(Community Health Services/ or Rural health Services/) and (exp Pregnancy/ or exp Delivery, Obstetric/ or Prenatal Care/ or Perinatal Care/ or Postnatal Care/)	2130
7	(Community Health Services/ or Rural health Services/) and (foetal or fetal or foetus or fetus or newborn* or neonat* or infant* or baby or babies or maternal or maternity or pregnant or pregnancy or pregnancies or prenatal or pre-natal or antenatal or ante-natal or perinatal or peri-natal or postnatal or post-natal).ti,ab.	2436
8	((maternity or obstetric?) adj3 (service? or hospital? or ward? or unit? or department? or dept? or centre? or center?)).ti,ab.	20646
9	((maternity or maternal or maternal health or obstetric) adj care).ti,ab.	8066
10	((delivery or birth*) adj (service? or hospital? or ward? or unit? or department? or dept? or centre? or center?)).ti,ab.	2990
11	((delivery or birth) adj care).ti,ab.	797
12	((foetal or fetal or foetus or fetus or newborn* or neonat* or infant* or baby or babies or maternal or maternity or pregnant or pregnancy or pregnancies or prenatal or pre-natal or antenatal or ante-natal or perinatal or peri-natal or postnatal or post-natal) adj3 (service? or hospital? or ward? or unit? or department? or dept? or centre? or center?)).ti,ab.	49515
13	((foetal or fetal or foetus or fetus or newborn* or neonat* or infant* or baby or babies or maternal or maternity or pregnant or pregnancy or pregnancies or prenatal or pre-natal or antenatal or ante-natal or perinatal or peri-natal or postnatal or post-natal) adj care).ti,ab.	29272
14	(((communit* or rural or district? or local*) adj3 (service? or hospital? or ward? or unit? or department? or dept? or centre? or center?)) and (foetal or fetal or foetus or fetus or newborn* or neonat* or infant* or baby or babies or maternal or maternity or pregnant or pregnancy or pregnancies or prenatal or pre-natal or antenatal or ante-natal or perinatal or peri-natal or postnatal or post-natal)).ti,ab.	7602
15	(((communit* or rural or district? or local*) adj care) and (foetal or fetal or foetus or fetus or newborn* or neonat* or infant* or baby or babies or maternal or maternity or pregnant or pregnancy or pregnancies or prenatal or pre-natal or antenatal or ante-natal or perinatal or peri-natal or postnatal or post-natal)).ti,ab.	141
16	(((level 1 or level i or level one or level 2 or level ii or level two or level 3 or level iii or level three) adj5 (service? or hospital? or ward? or unit? or department? or dept? or centre? or center? or care)) and (foetal or fetal or foetus or fetus or newborn* or neonat* or infant* or maternal or maternity or pregnant or pregnancy or prenatal or pre-natal or antenatal or ante-natal or perinatal or peri-natal or postnatal or post-natal)).ti,ab.	1408
17	1 or 2 or 4 or 5 or 6 or 7 or 8 or 9 or 10 or 11 or 12 or 13 or 14 or 15 or 16	108968
18	regional health planning/ or "catchment area (health)"/	12545

For peer review only

19	"Transportation of Patients"/	8903
20	Transportation/	8928
21	Health Services Accessibility/	65383
22	Travel/	23261
23	(travel* adj5 (distance or time)).ti,ab.	7255
24	("distance to" adj5 (hospital? or service? or unit? or ward? or centre? or center?)).ti,ab.	5849
25	("time to" adj5 (hospital? or service? or unit? or ward? or centre? or center?)).ti,ab.	37417
26	(("travel to" or "travel?ing to") adj5 (hospital? or service? or unit? or ward? or centre? or center?)).ti,ab.	1394
27	(("transfer to" or "transferred to") adj5 (hospital? or service? or unit? or ward? or centre? or center?)).ti,ab.	13476
28	(("transport to" or "transported to" or "transportation to") adj5 (hospital? or service? or unit? or ward? or centre? or center?)).ti,ab.	6259
29	(("access to" or accessibility) adj5 (hospital? or service? or unit? or ward? or centre? or center?)).ti,ab.	22957
30	(access* or travel* or distance* or transport* or transfer*).ti.	360710
31	((regional* or central or decentral*) adj5 (hospital? or service? or unit? or ward? or centre? or center?)).ti,ab.	36894
32	(regionali?e* or regionali?ation or centrali?e* or centrali?ation or decentrali?e or decentrali?ation).ti,ab.	18456
33	(geographic adj5 (hospital? or service? or unit? or ward? or centre? or center?)).ti,ab.	1951
34	((geographic* or district? or residence) adj5 area?).ti,ab.	24860
35	catchment*.ti,ab.	10031
36	((small or medium or large) adj2 (hospital? or unit?)).ti,ab.	14557
37	(small volume or medium volume or high volume).ti,ab.	18240
38	Health Facility Closure/	2317
39	(close or closed or closing or closure? or discontinu*).ti,ab.	609722
40	(preclos* or pre-clos* or postclos* or post-clos*).ti,ab.	406
41	downsiz*.ti,ab.	1966
42	(midwifery led adj2 (service? or unit? or ward? or centre? or center?)).ti,ab.	46
43	(midwifery adj (service? or unit? or ward? or centre? or center?)).ti,ab.	352
44	((consultant led or obstetrician led) adj2 (service? or unit? or ward? or centre? or center?)).ti,ab.	132
45	((consultant or obstetrician) adj (service? or unit? or ward? or centre? or center?)).ti,ab.	159
46	(referral* adj5 (service? or unit? or ward? or centre? or center?)).ti,ab.	28869
47	("model of care" or care model* or "model of delivery" or delivery model* or "model of service" or service model*).ti,ab.	12723
48	18 or 19 or 20 or 21 or 22 or 23 or 24 or 25 or 26 or 27 or 28 or 30 or 31 or 32 or 33 or 34 or 35 or 36 or 37 or 38 or 39 or 40 or 41 or 42 or 43 or 44 or 45 or 46 or 47	1251197
49	17 and 48	15581

For peer review only

Meta-analysis of Observational Studies in Epidemiology (MOOSE) Checklist

The impact of obstetric unit closures, travel time and distance from women's home to obstetric services on maternal and neonatal outcomes: a systematic review

Criteria		Brief description of how the criteria were handled in the meta-analysis
**Reporting of background should include**		
√	Problem definition	Closure of small obstetric units (OUs) and centralisation of obstetric services in larger units has been proposed to increase levels of consultant obstetrician cover to improve safety and limit costs. However, closure of OUs or conversion of OUs to midwifery-led units/community-based services potentially leads to an increase in travel distance or time for women in labour from their home to the nearest obstetric unit (Page 1).
√	Hypothesis statement	Increases in travel time/distance could potentially increase the risk of adverse birth outcomes (Page 1).
√	Description of study outcomes	Maternal and neonatal outcomes.
√	Type of exposure or intervention used	The closure of obstetric units was compared to no closure of obstetric units in the same population.<br>A shorter travel distance or time to OUs was compared with a longer travel distance or time (Page 7&8).
√	Type of study designs used	We included any quantitative study design with a comparison group (Page 6).
√	Study population	We included studies of women giving birth in high-income (OECD) (Page 7).
**Reporting of search strategy should include**		
√	Qualifications of searchers	All authors contributed to developing the search strategy. The information specialist NR has been acknowledged (Page 9).
√	Search strategy, including time period included in the synthesis and keywords	See Medline search strategy in Appendix 2.<br>Search time: 1990 to 2019 (Page 9).
√	Databases and registries searched	We searched: Embase, Medline, PsycINFO, Applied Social Science Index and Abstracts (ASSIA) and Cumulative Index to Nursing and Allied Health (CINAHL) databases. We also searched British Library EThOS, Open Grey and ProQuest Dissertations & Theses Global (Page 9).
√	Search software used, name and version, including special features	OvidSP, We detailed the MeSH/ Emtree Headings and text words in Appendix 2 (Page 9).
√	Use of hand searching	Bibliographies of the included papers were hand searched for additional references (Page 9).
√	List of citations located and those excluded, including justifications	Details of the literature search process are outlined in the PRISMA flow chart (Figure 1). The citation list of excluded articles is available from authors upon request (Page 11).

For peer review only - http://bmjopen.bmj.com/site/about/guidelines.xhtml

√	Method of addressing articles published in languages other than English	We placed no restrictions on language. Studies published in languages other than English were translated into English and included in the review if they fulfilled the review inclusion criteria (Page 9).
√	Method of handling abstracts and unpublished studies	We contacted a number of authors for full report of relevant unpublished studies (Page 10).
√	Description of any contact with authors	We contacted authors of relevant articles for full report of their unpublished studies. NHS Trusts and Health Boards in the UK were also contacted where we had been able to identify an OU closure. For details of the authors who provided extra unpublished information, see the acknowledgments section (Page 29).
**Reporting of methods should include**		
√	Description of relevance or appropriateness of studies assembled for assessing the hypothesis to be tested	Detailed inclusion and exclusion criteria are described in tables 1A (Page 31 – 36), 2A (Page 44 – 51) and 3A (Page 62 – 69).
√	Rationale for the selection and coding of data	A data extraction form was developed (available on request) to extract data related to characteristics of the study participants, time and distance measured, outcomes reported and quality assessment (Page 9&10).
√	Assessment of confounding	We have highlighted where crude (unadjusted) odds ratios (cOR) and adjusted odds ratios (adj OR) have been reported (Page 10). However, data were adjusted for different confounding factors across the included studies.
√	Assessment of study quality, including blinding of quality assessors; stratification or regression on possible predictors of study results	We used a modified version of the Effective Practice and Organisation of Care (EPOC) and the risk of bias in non-randomised studies of interventions Newcastle-Ottawa scale (NOS) (for case-control studies). No meta-analysis was feasible therefore; we were unable to conduct any sensitivity analysis to compare results from only low risk of bias studies (Page 10).
√	Assessment of heterogeneity	NA
√	Description of statistical methods in sufficient detail to be replicated	We found the included studies varied in their study design, categories of exposure, outcomes reported, whether adjusted or unadjusted results were presented and factors adjusted for. Due to this clinical and methodological variability quantitative pooling data was not feasible (Page 10).
√	Provision of appropriate tables and graphics	We included PRISMA flow chart to show the method of studies identification.<br>Table 1A: Description of included studies - Obstetric unit closures (Page 31 – 36).<br>Table 1B: Risk of bias - Obstetric unit closure studies (Page 37 – 39).<br>Table 1C: Outcomes - Obstetric unit closure (Page 40 – 47).<br><br>Table 2A: Description of included studies - Travel distance (Page 47 – 51).

For peer review only - http://bmjopen.bmj.com/site/about/guidelines.xhtml

		Table 2B: Risk of bias - Travel distance (Page 51 – 55).<br>Table 2C: Outcomes - Travel distance (Page 56 – 61).<br><br>Table 3A: Description of included studies - Travel time (Page 62 – 69).<br>Table 3B: Risk of bias - Travel time (Page 70 – 74).<br>Table 3C: Outcomes - Travel time (Page 75 – 90).
**Reporting of results should include**		
√	Graph summarizing individual study estimates and overall estimate	NA
√	Table giving descriptive information for each study included	Table 1A (Page 31 – 36), 2A (Page 47 – 51), 3A (Page 62 – 69).
√	Results of sensitivity testing	NA
√	Indication of statistical uncertainty of findings	The 95% confidence interval (CI) is reported to estimate the precision of the ORs and RRs in table 1C (Page 40 -47), 2C (Page 56 – 61) and table 3C (Page 75 – 90).
**Reporting of discussion should include**		
√	Quantitative assessment of bias	
√	Justification for exclusion	Reasons for exclusion were reported in PRISMA flow chart.
√	Assessment of quality of included studies	All presented in details in table 1B (Page 37 – 39), 2B (Page 51 – 55) and 3B (Page 70 – 74).
**Reporting of conclusions should include**		
√	Consideration of alternative explanations for observed results	Results were inconsistent across studies and no meta-analysis was feasible (Page 26).
√	Generalization of the conclusions	We reported the fact that almost all of the included studies were from Europe. (Page 26).
√	Guidelines for future research	We highlighted the need to use routinely collected data. In addition to the need of applying, a standardized way of measuring travel time and distance from woman's place of residence to an OU. In addition to applying a statistical method which considers time-varying relationships and the outcomes (Page 28).
√	Disclosure of funding source	This research is funded by the National Institute for Health Research (NIHR) Policy Research Programme, conducted through the Policy Research Unit in Maternal Health and Care, 108/0001 (Page 29).

For peer review only - http://bmjopen.bmj.com/site/about/guidelines.xhtml

BMJ Open

Title: The impact of obstetric unit closures, travel time and distance to obstetric services on maternal and neonatal outcomes in high-income countries: a systematic review

Journal:	*BMJ Open*
Manuscript ID	bmjopen-2020-036852.R2
Article Type:	Original research
Date Submitted by the Author:	07-Sep-2020
Complete List of Authors:	Malouf, Reem; University of Oxford, Policy Research Unit in Maternal Health and Care, National Perinatal Epidemiology Unit, Nuffield Department of Population Health<br>Tomlinson, Claire; University of Birmingham, Institute of Applied Health Research, College of Medical and Dental Sciences<br>Henderson, Jane; University of Oxford, Policy Research Unit in Maternal Health and Care, National Perinatal Epidemiology Unit, Nuffield Department of Population Health<br>Opondo, Charles; University of Oxford, Policy Research Unit in Maternal Health and Care, National Perinatal Epidemiology Unit, Nuffield Department of Population Health;<br>Brocklehurst, Peter; University of Birmingham, Institute of Applied Health Research, College of Medical and Dental Sciences<br>Alderdice, Fiona; University of Oxford, Policy Research Unit in Maternal Health and Care, National Perinatal Epidemiology Unit, Nuffield Department of Population Health<br>Phalguni, Angaja; University of Birmingham, Institute of Applied Health Research, College of Medical and Dental Sciences<br>Dretzke, Janine; University of Birmingham, Institute of Applied Health Research, College of Medical and Dental Sciences
**Primary Subject Heading**:	Health services research
Secondary Subject Heading:	Obstetrics and gynaecology, Health services research, Evidence based practice
Keywords:	EPIDEMIOLOGY, OBSTETRICS, PUBLIC HEALTH

SCHOLARONE™
Manuscripts

I, the Submitting Author has the right to grant and does grant on behalf of all authors of the Work (as defined in the below author licence), an exclusive licence and/or a non-exclusive licence for contributions from authors who are: i) UK Crown employees; ii) where BMJ has agreed a CC-BY licence shall apply, and/or iii) in accordance with the terms applicable for US Federal Government officers or employees acting as part of their official duties; on a worldwide, perpetual, irrevocable, royalty-free basis to BMJ Publishing Group Ltd ("BMJ") its licensees and where the relevant Journal is co-owned by BMJ to the co-owners of the Journal, to publish the Work in this journal and any other BMJ products and to exploit all rights, as set out in our licence.

The Submitting Author accepts and understands that any supply made under these terms is made by BMJ to the Submitting Author unless you are acting as an employee on behalf of your employer or a postgraduate student of an affiliated institution which is paying any applicable article publishing charge ("APC") for Open Access articles. Where the Submitting Author wishes to make the Work available on an Open Access basis (and intends to pay the relevant APC), the terms of reuse of such Open Access shall be governed by a Creative Commons licence – details of these licences and which Creative Commons licence will apply to this Work are set out in our licence referred to above.

Other than as permitted in any relevant BMJ Author's Self Archiving Policies, I confirm this Work has not been accepted for publication elsewhere, is not being considered for publication elsewhere and does not duplicate material already published. I confirm all authors consent to publication of this Work and authorise the granting of this licence.

For peer review only

Title: The impact of obstetric unit closures, travel time and distance to obstetric services on maternal and neonatal outcomes in high-income countries: a systematic review

Reem Malouf[1*], Claire Tomlinson[2], Jane Henderson[1], Charles Opondo[1], Peter Brocklehurst[1], Fiona Alderdice[1], Angaja Phalguni[2], Janine Dretzke[2]

[1]Policy Research Unit in Maternal Health and Care, National Perinatal Epidemiology Unit, Nuffield Department of Population Health, University of Oxford, Old Road Campus, Old Road, Oxford, UK

[2]Institute of Applied Health Research, College of Medical and Dental Sciences, University of Birmingham

*** Corresponding author**

reem.malouf@npeu.ox.ac.uk

For peer review only

Abstract

Objectives: To systematically review a) The effect of obstetric unit (OU) closures on maternal and neonatal outcomes; b) The association between travel distance/time to an OU and maternal and neonatal outcomes.

Design: Systematic review of any quantitative studies with a comparison group.

Data sources: Embase, MEDLINE, PsycINFO, Applied Social Science Index and Abstracts, CINAHL and grey literature were searched.

Methods: Eligible studies explored the impact of closure of an OU or the effect of travel distance/time on pre-specified maternal or neonatal outcomes. Only studies of women giving birth in high-income countries with universal health coverage of maternity services comparable to the UK were included. Identification of studies, extraction of data and risk of bias assessment were undertaken by at least two reviewers independently. The risk of bias checklist was based on the Cochrane Effective Practice and Organisation of Care (EPOC) criteria and the Newcastle-Ottawa scale (NOS). Heterogeneity across studies precluded meta-analysis and synthesis was narrative, with key findings tabulated.

Results: 31 studies met the inclusion criteria. There was some evidence to suggest an increase in babies born before arrival (BBA) following OU closures and/or associated with longer travel distances or time. This may be associated with an increased risk of perinatal or neonatal mortality, but this finding was not consistent across studies. Evidence on other maternal and neonatal outcomes was limited but did not suggest worse outcomes after closures or with longer travel times/distances. Interpretation of findings for some studies was hampered by concerns around how accurately exposures were measured, and/or a lack of adjustment for confounders or temporal changes.

For peer review only - http://bmjopen.bmj.com/site/about/guidelines.xhtml

For peer review only

Conclusion: It is not possible to conclude from this review whether OU closure, increased travel distances or times are associated with worse outcomes for the mother or the baby.

Prospero registration number: CRD42017078503.

Strengths and Limitations

- This review is the first to synthesise systematically the current evidence on the impact of closure of obstetric units and of travel distance and travel time to obstetric units on neonatal and maternal outcomes.
- Rigorous systematic review methodology was applied including a sensitive search strategy to ensure all relevant evidence was identified.
- Heterogeneity across included studies precluded any form of meta-analysis.
- A paucity of evidence on a number of outcomes, and methodological concerns for some studies limited conclusions that could be drawn.

For peer review only

For peer review only - http://bmjopen.bmj.com/site/about/guidelines.xhtml

For peer review only

For peer review only - http://bmjopen.bmj.com/site/about/guidelines.xhtml

Background

Closure of small obstetric units (OUs) and centralisation of obstetric services in larger units has been proposed to increase levels of consultant obstetrician cover to improve safety and limit costs. However, closure of OUs or conversion of OUs to midwifery-led units/community-based services potentially leads to an increase in travel distance or time for women in labour from their home to the nearest obstetric unit. Increases in travel time could potentially increase the risk of adverse birth outcomes.

Travel time and distance are widely used as measures to explore the geographical accessibility of health services (1). In a systematic review (2) the association between travelling further to healthcare facilities and having worse health outcomes was established, but the review did not include studies of maternity care. The impact of OU closure and increase in travel time/distance to the OU on perinatal and maternal outcomes have not been systematically assessed. One review (3) evaluating the effects of regionalisation of perinatal services has been published. This concluded that regionalisation programmes appeared to be correlated with improvements in perinatal outcomes but that the evidence was weak. A narrative review (4) included 10 studies that explored travel time and distance to and between maternity services and adverse birth outcomes to inform the consultation on maternity services in Wales. The review was limited to studies reported in English and there was no clear association between travel distance or time and adverse birth outcomes

Therefore, uncertainty remains about the association between OU closure, prolonged time or distance to OUs and adverse perinatal outcomes. Specifically, there is a rise in the risk of babies born before arrival (BBA, also referred to as unplanned out of hospital births). Being born before arrival is more common before term and has been reported to be associated with higher perinatal mortality (5). Conversely, Lasswellet et al. (6), found neonatal mortality was reduced when services were configured to ensure very preterm infants are born in a large maternity hospital with neonatal intensive care unit (Level III). In addition to mortality, Apgar scores (a standardised measure of the

For peer review only - http://bmjopen.bmj.com/site/about/guidelines.xhtml

physical condition of a newborn infant) and neonatal admission to intensive care provide an indication of perinatal infant health.

The impact on maternal outcomes is also unclear. There are concerns that low risk women who give birth in larger hospitals may experience more interventions, for example, increased frequency of caesarean section (CS) (7). Along with CS, evidence on maternal mortality and maternal birth complications such as post-partum haemorrhage (PPH) and maternal blood transfusion, was also sought in this review to identify the potential impact of OU closure on maternal outcomes.

In this review we aimed to systematically identify, critically appraise and synthesise the evidence relating to:

a) The effect of OU closures on maternal and neonatal outcomes (compared with the surrounding area or a comparable population).

b) The association between travel distance or time to an OU and maternal and neonatal outcomes.

Review method

The protocol was registered with Prospero (registration number CRD42017078503) and the Meta-Analyses and Systematic Reviews of Observational Studies MOOSE reporting guideline was followed (8).

Criteria for considering studies for this review

Types of studies

Any quantitative study design with a comparison group was eligible for inclusion. Studies were included from 1990 onwards. The year 1990 was chosen as a cut-off date because significant advances were made in neonatal care in the early 1990s, such as surfactant therapy, assisted ventilation, prophylactic infection control and antenatal steroid therapy, which impacted on the

For peer review only - http://bmjopen.bmj.com/site/about/guidelines.xhtml

delivery of maternity services (9). The quantitative components of mixed methods studies were also eligible. Studies were included if they:

- Explored the impact of closure of an OU on maternal or neonatal outcomes either in a before-and-after comparison (same population catchment area), or a geographical comparison of different areas (comparable populations)

And/or

- Compared maternal and neonatal outcomes after an OU closure and retention or creation of midwifery led units to replace the OU
- Explored the effect of travel time and/or distance on maternal and neonatal outcomes providing at least two travel times and/or distances from women's homes to the nearest OU
- Explored maternal and neonatal outcomes following maternal transfer from planned or unplanned home birth to the nearest maternity centre.

We included studies of women giving birth in high-income (OECD) countries with universal health coverage (UHC) of maternity services comparable to the UK. The list of OECD countries is shown in appendix 1. UHC is defined as health care that meets everyone's right to access high quality essential health services where and when they need them without financial difficulty (10).

Types of exposures

OU closure: the closure of an OU was compared to no closure of an OU for the same or comparable geographical catchment areas prior to the closure. For a study comparing different geographical areas affected by the closure of an obstetric unit, the least affected area was used as a control group. For the purpose of this review we used the definition of an OU used in the Birthplace Research programme in England (11), which defined an OU as "*a clinical location in which care is provided by a team, with obstetricians taking primary professional responsibility for women at high*

For peer review only - http://bmjopen.bmj.com/site/about/guidelines.xhtml

For peer review only

risk of complications during labour and birth. Midwives offer care to all women in an OU, whether or not they are considered at high or low risk, and take primary responsibility for women with straightforward pregnancies during labour and birth. Diagnostic and treatment medical services including obstetric, neonatal and anaesthetic care are available on site, 24 hours a day"(11)(P12).

Travel distance or time to the nearest OU: a shorter travel distance or time was compared with a longer travel distance or time. We used the definition of a shorter or a longer time or distance as defined by the included studies. When a study compared several different travel times or distances to the nearest OU, those with the shortest travel distance or time were used as the control group.

The following types of studies were excluded:

- Studies comparing maternal and or neonatal outcomes based on hospital size, level of neonatal intensive care, type of hospital or model of care (e.g. caseload midwifery care versus consultant care)
- Studies on regionalisation of neonatal care (number of centres with neonatal intensive care (NICUs))
- Studies where a proximity rather than the actual travel time or travel distance was given (e.g. rural vs urban, remote vs very remote areas)
- Studies which did not report at least one of the outcomes.

Review outcomes

The following outcomes were pre-defined in the study protocol:

Maternal outcomes

For peer review only - http://bmjopen.bmj.com/site/about/guidelines.xhtml

Maternal mortality (MM), caesarean section (CS) (overall, emergency or intrapartum), severe perineal trauma (including 3rd and 4th degree tears), postpartum haemorrhage (PPH), maternal admission to intensive care unit (ICU) and maternal blood transfusion.

Neonatal outcomes

Stillbirth (SB) (overall or intrapartum), neonatal mortality (NM), perinatal mortality (PM), infant mortality (IM), babies born before arrival (BBA), neonatal unit admission (NNU), Apgar score and hypoxic-ischemic encephalopathy (HIE).

Review methods

A comprehensive search strategy was developed in collaboration with an information specialist (NR). We searched Embase, Medline, PsycINFO, Applied Social Science Index and Abstracts (ASSIA) and Cumulative Index to Nursing and Allied Health (CINAHL) databases (from 1990 to February 2019). We also searched the Grey literature in the databanks of British Library EThOS, Open Grey and ProQuest Dissertations & Theses Global. NHS Trusts and Health Boards in the UK were also contacted where we had been able to identify an OU closure to request information about any evaluations that were conducted. The references of eligible studies and relevant reviews were checked to identify additional studies not retrieved by the search. Searches were based on index terms and text words relating to the population/setting (e.g. maternity service, pregnancy, neonatal) and exposures (e.g. travel/distance or closure/regionalisation). Due to the variable nature of terms and indexing used, the strategy was kept broad by using a range of alternate terms and not limiting by outcome. No language restriction was applied. A sample search strategy for MEDLINE is shown in appendix 2.

At least two reviewers (RM, CT, AP, FA, JH) independently screened the references for relevance against the review eligibility criteria using Eppi-reviewer software (version 4)(12). Full-text study screening was also performed by at least two reviewers (RM, CT, CO, JH, FA). Disagreements

For peer review only - http://bmjopen.bmj.com/site/about/guidelines.xhtml

regarding study eligibility were resolved through discussion and consensus within the review team. We contacted authors of relevant studies published as abstracts for further information. Data extraction and risk of bias assessment were undertaken by at least two reviewers (RM, CT, CO, JH, FA, JD). The risk of bias checklist was adapted from the Effective Practice and Organisation of Care (EPOC) (13) and the risk of bias in non-randomised studies of interventions Newcastle-Ottawa scale (NOS) (for case-control studies) (14). Risk of bias assessment included selection of study groups, measurement of exposure and outcomes, missing data and appropriateness of analysis (e.g. logistic regression analysis). For case-control studies, selection and comparability of cases and controls were also considered. The review team rated the quality of evidence for each domain in the tool as low, high or unclear risk of bias, or yes, no and unclear in meeting quality criteria.

Results were synthesised narratively and the key findings tabulated. The included studies varied in their study design, categories of exposure, outcomes reported, whether adjusted or unadjusted results were presented and factors adjusted for. This clinical and methodological heterogeneity across the included studies precluded any form of meta-analysis. Pre-specified sub-groups were risk-status of woman (low versus high), parity, gestational age, UK studies compared with non-UK studies, and planned vs unplanned CS; formal sub-group analyses were however not possible . Evidence regarding OU closure, travel distance and travel time is reported separately, and by outcome. We have highlighted where crude (unadjusted) odds ratios (cOR) and adjusted odds ratios (adj OR) have been reported.

Patient and Public Involvement

We involved our Parent, Patient, and Public Involvement (PPPI) Stakeholders Network, to explore which outcomes were important from a maternal perspective. The dissemination of findings to stakeholders will be through plain language summaries developed with members of our PPPI stakeholder network.

For peer review only - http://bmjopen.bmj.com/site/about/guidelines.xhtml

Search results

Searches of bibliographic databases and other sources from 1990 to February 2019 yielded 13,271 unique references and the steps of study selection are presented in PRISMA flow chart (Figure 1). The eligibility of 295 full-text articles were assessed independently. 260 articles were excluded for various reasons, including: studies conducted in low/middle income countries, comparing different models or levels of maternity care, assessing women's transfer from primary to secondary maternity centres, or not providing quantifiable measures of travel time/distance (full list available from authors). Thirty-one studies, reported in 35 articles, met the review eligibility criteria (Figure 1). One study (5), included information on both OU closure and travel distance. Ten studies provided information on OU closures, seven studies compared different travel distances from women's homes to the nearest OU; and 15 studies compared different travel times from women's homes to the nearest OU.

Review results

Evidence from obstetric unit (OU) closures

A detailed description of the ten included OU closure studies is presented in Table 1A. Three studies were from the UK, with two reported as abstracts only (15, 16) and one an unpublished data series from East Lancashire Hospitals National Health Services (NHS) Trust, UK (East Lancashire Hospitals NHS Trust, unpublished data 2017). There appeared to be overlap between populations reported in two studies (East Lancashire Hospitals NHS Trust, unpublished data 2017) and (15). Three studies were from Scandinavia (7, 17, 18), three from Canada (19-21) and one from France (5).

Seven studies compared adverse birth outcomes before and after centralisation of services, which included closure of varying numbers of OUs. All three studies from the UK (15, 16) and (East Lancashire Hospitals NHS Trust, unpublished data 2017), examined the impact of the amalgamation

For peer review only - http://bmjopen.bmj.com/site/about/guidelines.xhtml

of two OUs. Four studies were published after 2014 (15-17, 20); the earliest was from 1990 (21). Three studies included all births (17, 18, 21); the other studies varied in their eligibility criteria, e.g. restricting the analysis to singletons, live births, various gestational ages and birthweight, hospital births or location. Reporting of eligibility criteria and participant characteristics across studies was inconsistent (Table 1A).

Risk of bias assessment

Risks of bias related to a lack of reporting of whether changes over time (other than closure/reconfiguration) could have influenced the findings, with only two (17, 20) of ten studies reporting that temporal variation was adjusted for in the analysis (Table 1B). Further, five out of ten studies either did not adjust results for potential confounding factors or provided insufficient information to know whether this was undertaken. Five out of ten studies did not provide sufficient information to gauge the completeness of data. Half of the studies reported and used appropriate data analysis methods. Other potential sources of bias (e.g. relating to selection, exposure and outcome) were less of a concern due to the use of routinely collected registry data before and after the closure and the objective nature of most outcomes.

For peer review only

Findings

A summary of maternal and neonatal outcomes is presented in Table 1C.

Maternal outcomes

Maternal mortality (MM)

In the two studies that reported maternal mortality (16, 20), the number of deaths (<5) was too low to allow comparisons between the pre- and post-closure groups .

For peer review only - http://bmjopen.bmj.com/site/about/guidelines.xhtml

Caesarean section (CS) (overall or intrapartum)

Total CS rates were reported in four studies (7, 15, 20, 21). One UK study (15) reported a decline in CS rates following the amalgamation of two units from 26.1% to 21.5%.

A Norwegian study (7), reported an increase in CS rates from 13.1% to 16.4% following OU closure, (cOR=1.31, 95%: 1.27, 1.35) as did two Canadian studies (21)cOR= 1.13, 95%CI: 1.09, 1.18) and (cOR= 1.10, 95%CI: 1.01, 1.19)(20). No adjusted results were reported.

Emergency caesarean section (CS)

Emergency CS rates were reported in one UK study (16), which found no difference before/after the amalgamation of two OUs (cOR=0.95, 95%CI: 0.86, 1.05).

Severe perineal trauma (3rd or 4th degree tear).

Two studies (16, 20) reported this outcome and found no statistically significant difference between the before/after closure groups. The incidence of the outcome in both studies was low (<3%).

Postpartum haemorrhage – No studies reported this outcome.

Maternal admission to intensive care unit (ICU)

Two studies (16, 20) found no significant difference before/after the amalgamation of two OUs on the number of women requiring admission to ICU (cOR= 0.80, 95%CI: 0.44, 1.46) (16). The numbers in one study (20) were too small (<5) to allow a comparison.

Maternal blood transfusion

One study (20) found no significant differences before/after OU closure (cOR=0.82, 95%CI: 0.55, 1.21). The incidence of the outcome was low (<1% of women).

Neonatal outcomes

Stillbirth (SB) (overall or intrapartum)

For peer review only - http://bmjopen.bmj.com/site/about/guidelines.xhtml

Three studies examined the impact of OU closure on stillbirth. One unpublished UK study (East Lancashire Hospitals NHS Trust, unpublished data 2017) showed a statistically significant reduction in stillbirths over the period after the amalgamation of two units (cOR=0.61, 95%CI: 0.47, 0.78). Similar findings were seen in one study from Canada (22) during post closure years (cOR=0.80, 95%CI: 0.67, 0.96). A third study from the UK (16) found no difference in stillbirth rates after OU closure.

Neonatal mortality (NM)

Three studies reported this outcome. Two studies from the UK (16) and (East Lancashire Hospitals NHS Trust, unpublished data 2017), showed no statistically significant difference in the rate of NM in the years after OU closure (cOR= 1.33, 95%CI: 0.81, 2.17; cOR= 0.80, 95%CI: 0.29, 2.26). A study from Norway (17) also reported no difference (no OR presented).

Perinatal mortality (PM)

Two studies reported this outcome. In a study from Norway (7), PM was significantly lower following OU closure (cOR= 0.76, 95%CI: 0.58, 0.98). A Canadian study (22) also reported a significant reduction in PM after OU closure (cOR= 0.75, 95%CI: 0.64, 0.87).

Infant mortality (IM)

One study (17) reported this outcome, IM rates were “not statistically elevated” after the closure of thirteen hospitals in Norway.

Born before arrival (BBA)

Six studies reported this outcome, with four suggesting a statistically significant increase in BBA following OU closure. Data from East Lancashire Hospitals NHS Trust (East Lancashire Hospitals NHS Trust, unpublished data 2017), showed the BBA rate almost doubled over the 10-year period (cOR= 1.88, 95%CI: 1.25, 2.82). Studies from Norway (7) and Finland (18) also found that the BBA rate increased over a similar period (cOR=1.8, 95%CI: 1.6, 1.9 and cOR=4.14, 95%CI: 3.16, 5.41

For peer review only - http://bmjopen.bmj.com/site/about/guidelines.xhtml

For peer review only

respectively). A Canadian study (20) found that the BBA rate trebled over a 16 year period (cOR= 3.47, 95%CI: 2.31, 5.20). One UK study (16) found no statistically significant change (cOR= 1.28, 95%CI: 0.63, 2.60) and in one French study (5), there was weak evidence of a small reduction in the adjusted risk of BBA in communities affected by OU closure (adjOR = 0.91, 95%CI: 0.84, 1.00).

Neonatal Unit admission (NNU)

One Canadian study (20) suggested a significant reduction in NNU admission following OU closure (cOR= 0.39, 95%CI: 0.25, 0.60).

Apgar score

One Canadian study (20) found no statistically difference in 5 minute Apgar score of less than 7 before and after OU closure (cOR= 1.13, 95%CI: 0.82, 1.55)

Hypoxic-Ischemic Encephalopathy (HIE) – No studies reported this outcome.

Evidence from travel distance studies

Description of included studies

Seven studies described the effect of travel distance to the nearest OU on maternal and neonatal outcomes (Table 2A). All were published in full between 1991 and 2015. The earliest study (23) was conducted in the UK, three more recent studies were conducted in France (5, 24, 25), and one each from Norway (26), Finland (27), and Canada (28). Four were retrospective population-based cohort studies, and three were case-control studies. The eligibility criteria varied across studies. Pasquier et al. (24), included a group with special needs in the form of babies with congenital malformations. Only singleton live births were included in two studies (5, 28).

Travel distance was estimated using geographical mapping software in all studies. However, only three studies (5, 24, 27) measured the actual distance from women's homes to the nearest OU. In two studies (25, 28) a central geographical point for the postcode or municipality was used to

For peer review only - http://bmjopen.bmj.com/site/about/guidelines.xhtml

For peer review only

estimate distances and in one study the distance was self-reported (26). Additionally, the studies differed regarding their distance categories, which ranged from 2km to 150km (Table 2A).

Risk of bias assessment

The main risk of bias concerns related to the measurement of exposure, as three studies (25, 26, 28) did not calculate the distance from the woman's home but used a central point instead or self-reported distance (Table 2B). Another study (23), measured distance between women's homes and hospital using a straight line. Further risk of bias related to a lack of comparability between study groups in the three case-control studies (23, 26, 27) , a lack of adjustment for confounders in two studies and missing data in two studies (26, 27). There were no risk of bias concerns relating to sample selection in the cohort studies or outcome measurement.

Findings

Maternal outcomes

Maternal mortality (MM)

In one case-control study from Finland (27), no maternal deaths were reported in either group (Table 2C).

Caesarean section (CS) (overall, or intrapartum)

One study from Canada (28) found no statistically significant differences in CS rates with increasing distance (<50k, 50-150k, >150k) based on both crude and adjusted results.

Emergency Caesarean section (CS)

The same study from Canada (28) reported no significant difference in emergency CS rates between women living at different distances from an OU based on crude ORs.

For peer review only - http://bmjopen.bmj.com/site/about/guidelines.xhtml

No studies reported on *severe perineal trauma, postpartum haemorrhage, maternal admission to intensive care unit (ICU) or maternal blood transfusion.*

Neonatal outcomes

Stillbirth (SB) (overall or intrapartum)

Two cohort studies (25, 28) reported this outcome. A Canadian study (28) included births to women aged over 35 years who lived <50km, 50-150km and >150 km from the OU. Stillbirth rates did not change by distance category in the adjusted analysis. A French study (25) reported stillbirth rates at different distances (<5 km, 5-15, 15-30, 30-44, 45+ km) from mother's municipality of residence to the closest OU. The crude odd ratios showed women living at 30-44, 15-29 and 5-14km from an OU had a statistically significantly lower rate of stillbirth compared to women living <5 km from an OU (5-14 km vs <5 km, cOR = 0.87, 95%CI: 0.85, 0.90; 15-29 km vs <5 km; cOR = 0.85, 95%CI: 0.82, 0.88; 30-44 km vs <5 km, cOR= 0.85, 95%CI: 0.81, 0.90). The findings still hold for the adjusted analysis (limited data reported). However, neither the crude nor the adjusted analysis showed a significant difference in risk of stillbirth for individuals resident 45+ km from an OU compared with <5km.

Neonatal mortality (NM)

Two French cohort studies (24, 25) reported this outcome. One study (24) examined the distance from women's homes to the nearest OU with neonatal surgical facilities for 706 foetuses with severe malformations. Analyses adjusted for malformation type, number of malformations, amniotic fluid anomaly, previous anomaly in the family and parity showed no association between NM and distance (<11 km vs 11-50 km, adj OR = 0.89, 95%CI: 0.34, 2.88; <11 km vs > 50 km, adj OR= 1.37, 95%CI: 0.49, 3.86). The other study (25) included all births and found that NM rates were significantly higher for women living <5 km compared to 5 -44 km away from an OU (25) (5-14 km vs < 5 km, cOR= 0.78, 95%CI: 0.75, 0.81; 15-29 km vs < 5 km cOR = 0.81, 95%CI: 0.78, 0.85; 30-44 km

For peer review only - http://bmjopen.bmj.com/site/about/guidelines.xhtml

vs < 5 km, cOR= 0.78, 95%CI: 0.72, 0.84; ≥ 45 km vs < 5 km, cOR= 0.82, 95%CI: 0.70, 0.96). In this latter study, the NM of babies born before arrival was also explored. For the BBA group, there was a statistically significant increase in the risk of NM when women had to travel 45km or more to an OU in comparison to <5 km (≥ 45 km vs < 5 km, cOR= 2.45, 95%CI: 1.14, 5.25).

Perinatal mortality (PM)

A study from Canada (28) reported that PM risk increased with travel distance in an adjusted model (<50 km v 50-150 km adjOR=1.53, 95%CI: 1.1-2.12; <50 km >150km adjOR=3.06, 95%CI: 2.20,4.24).

Infant mortality (IM) - No studies reported this outcome.

Born before arrival (BBA)

Three case control studies (23, 26, 27), and one cohort study (5) reported this outcome. All four studies reported a significant increase in BBA rate with longer travel distance, although only two reported adjusted analyses (5, 27). In the UK study (23), the risk of BBA increased 12 fold for women living >7 km from the OU compared to women living <2 km away (cOR = 12.5, 95%CI: 4.02, 36.01). The risk of BBA increased significantly for women living >13 km from an OU in a Norwegian study (26) (cOR= 1.81, 95%CI: 1.03, 3.18). The Finnish study (27) reported a fivefold increased risk of BBA for women living >35 km from the OU compared to <35 km (adj OR 5.02, 95%CI: 1.80, 14.04).

In France (5), the rate of BBA significantly increased with longer distances and it tripled for all women living 45+ km from the OU compared to women living <5km away (cOR: 3.77, 95%CI: 3.23, 4.39). The association persisted in an adjusted analysis which included women of parity three or higher and living 45+ km from the OU, who had a six fold increased risk of BBA compared to women living <5 km away and of parity one or two (adj OR= 6.49, 95%CI: 4.92, 8.48).

Neonatal unit admission (NNU)

A study from Canada (28) reported an increase in NNU admission for births to women living >150 km from an OU compared to those living <50 km away (6.8% vs 4.8%).

For peer review only - http://bmjopen.bmj.com/site/about/guidelines.xhtml

For peer review only

Apgar score – No studies reported this outcome.

Hypoxic-Ischemic Encephalopathy (HIE) – No studies reported this outcome.

Evidence from travel time studies

Description of included studies

Fifteen studies explored the impact of travel time from a woman's home to an OU (see Table 3A). Two studies (one reported as an abstract only) were conducted in the UK (29-31), three studies in France (32-34), three studies (reported in five articles) in the Netherlands (35-39), one study reported in two articles from Norway (40, 41), five studies in Canada (42-46) and one study in Japan (47).

Eleven studies were of a retrospective cohort design, one was a prospective cohort study (39), one was a before-and-after design (47) and two were case-control studies (33, 34). All the studies clearly stated the eligibility criteria. Only singleton births were included in five studies (30-32, 35-38, 42). One study (39) specifically enrolled women with postnatal haemorrhage after home birth, and one study (42) focussed on planned home birth regardless of the actual place of birth.

The studies were heterogeneous in their travel time intervals. With the exception of one study in Canada (42), longer time cut-off points were examined in studies from Norway, Japan and Canada compared to studies in other countries (all European). Travel duration was estimated using geographical mapping software in all studies. However, most studies estimated travel duration to and from central points within areas rather than actual addresses.

Risk of bias assessment

Quality assessment and supported explanations for each of the risk of bias domains are presented in Table 3B. With the exception of Stolp et al. (39), sample selection and measurement of outcomes

For peer review only - http://bmjopen.bmj.com/site/about/guidelines.xhtml

were considered to be at low risk of bias across all studies as such data were obtained from national databases and birth registries. The two case- control studies were appropriately selected and defined, however the case and control groups were not comparable in both studies (e.g. difference in antenatal care attendance and socio-demographics). Eight studies (29, 34-42), were considered at low risk of exposure measurement bias, as the women's actual place of residence was used to estimate travel time to nearest OU. The risk of attrition bias was low for the majority of the included studies. Similarly, analyses and adjustment for potential confounders were found to be appropriate in the majority of studies.

Findings

Maternal outcomes

Maternal mortality - no studies reported this outcome

Caesarean section (overall, or intrapartum)

Five Canadian studies (42-46) reported CS rates (Table 3C). Across three studies (43-45), crude ORs for CS rates were higher among women who lived closer to OUs with CS rates highest for women living less than one hour away compared with other categories (1-2 hours, 2-4 hours and > 4 hours). One study (46) included women who had a midwife involved in their care, and found no significant differences in CS rates for women living 1-2 hours and more than 2 hours away compared to within one hour of an OU (1-2 vs < 1 hr, cOR= 1.23, 95%CI: 0.80, 1.91 and>2 hrs vs < 1 hr, cOR=1.11, 95%CI: 0.71, 1.72). A further study (42) showed also a higher CS rate among women who planned a home birth and lived less than half an hour away from OU services (>30 mins vs ≤ 30 mins,(cOR=0.74, 95%CI: 0.59, 0.92).

Emergency Caesarean section

Shorter travel time to an OU was associated with a statistically significant higher emergency CS rate in one Canadian study (45) (> 1hr vs < 1 hr, cOR= 0.80, 95%CI: 0.75, 0.86).

For peer review only - http://bmjopen.bmj.com/site/about/guidelines.xhtml

For peer review only

Severe perineal trauma – No studies reported this outcome.

Postpartum haemorrhage (PPH)

One Canadian study found the risk of PPH was significantly higher for women who lived more than one hour away from obstetric services compared to women who lived less than one hour away (44) (> 1hr vs < 1 hr, cOR=1.27, 95%CI: 1.13, 1.43).

Maternal admission to intensive care unit

One study from the Netherlands (39) involved women who had a PPH after midwifery-supervised home births and examined adverse maternal outcomes associated with travel time longer than 45 minutes to hospital. No difference was found in the number of women admitted to ICU who travelled more than 45 minutes compared with < 45 travel time to hospital, but the numbers of events were low.

Maternal blood transfusion

One study from the Netherlands (39) found no significant difference in the median number of units of blood transfused to women who travelled more than 45 minutes to an OU compared to <45 minutes travel time.

Neonatal outcomes:

Stillbirth SB (overall or intrapartum)

Three studies examined the association between increasing travel time and SB, one study each from the UK (30, 31), France (32) and Canada (44).

In the UK study (30, 31), there was no association between travel time and SB when analysing all women (adjOR = 1.13, 95%CI: 0.98, 1.30). However subgroup analyses showed a significant increase in the risk of SB with every 15 minute increase in travel time to the OU for term pregnancies

For peer review only - http://bmjopen.bmj.com/site/about/guidelines.xhtml

(adjOR=1.36, 95%CI: 1.17, 1.59) and for nulliparous women (adjOR=1.21, 95%CI: 1.02,1.44). The other two studies (32, 44) found no significant increase in the incidence of SB with increasing travel time .

Neonatal mortality (NM)

Five studies examined the association between travel time and NM, two from the UK (29-31), one from the Netherland (35-37), one from Canada (44) and one from Japan (47).

The adjusted analysis in one UK study(29) showed no statistically significant association between NM and travel time. The adjusted analyses in the other UK study (31) showed a significant increase in early and late NM, with every 15 minutes increase in travel time (adjOR= 1.13, 95%CI: 1.07, 1.20) and (adjOR= 1.15, 95%CI: 1.05, 1.26) respectively. Subgroup analysis for nulliparous women showed a statistically significant increased risk of early NM associated with every 15 min increase in travel time from home to the OU (adjOR= 1.15, 95%CI: 1.06, 1.25). For term births, late (but not early) NM increased significantly with every 15 mins increase travel time from home to the OU (adjOR= 1.34, 95%CI: 1.13, 1.59).

In one study from the Netherlands (35), a travel time of 20 minutes or more was associated with a significant increase in the combined intrapartum, early and late NM (35-37) (≥20 mins vs <20 mins, adjOR= 1.23, 95%CI: 1.07, 1.41). No neonatal mortality events were reported in the study from Canada (44). The study from Japan (47) reported that following a median reduction in travel time from 67 minutes in 2002 to 39 minutes in 2006 that there was a decrease in NM rate from 1.67 to 1.28, however no further analyses were presented.

Perinatal mortality (PM)

For peer review only - http://bmjopen.bmj.com/site/about/guidelines.xhtml

Seven studies examined PM, one from France (32), one from the Netherlands (38) and five from Canada (42-46). The French study (32) found no significant association between increasing travel time to the nearest OU and PM based on unadjusted data. However, in the Dutch study a longer travel time (20 mins or more) was significantly associated with higher PM (38) (>=20 mins vs < 20 mins, adjOR= 1.66, 95%CI: 1.59, 1.74).

The Canadian studies also reported longer travel times to OUs being associated with an elevated risk of PM. A significant increase in PM was reported in women living more than four hours away from OUs compared to women living less than one hour (> 4hrs vs < 1 hr adjOR= 3.17, 95%CI: 1.45, 6.95) (43). However, findings from the same study suggested no significant increase for women living 1-2 hours and 2 to 4 hours from an OU compared to those living less than one hour from services. Similarly, the PM risk significantly increased in women who lived > 1 hour from OUs in a further Canadian study (44), (cOR=1.54, 95%CI: 1.09, 2.17). When this was divided into different Canadian provinces (45), the rates of PM were highest in communities living more than 4 hrs from an OU in comparison to less than one hour in British Colombia only (adjOR=2.84, 95%CI: 2.84,5.10). Stoll et al. (46), found that in women who received midwifery care only, PM was not statistically significantly different for women living more than two hours away from an OU compared to women living less than one hour from an OU based on an unadjusted analysis (cOR= 3.57, 95%CI: 0.81,15.76). In Darling et al. (42), the PM rates were not statistically significantly different for women with a planned home birth and more than 30 minute drive from hospital (adjRR=2.2, 95%CI: 0.67,7.43).

Infant Mortality (IM)

One Canadian study (43) reported no significant difference in IM rates for women living less than one hour travel time to OU compared with more than one hour travel time to OU (cOR= 1.51, 95%CI: 0.86, 2.63).

For peer review only - http://bmjopen.bmj.com/site/about/guidelines.xhtml

Born before arrival (BBA)

Six studies reported this outcome, four cohort studies (32, 41, 43, 44), and two case-control studies (33, 34). Five of the six studies found some association between travel time and BBA, four based on adjusted analyses.

There were three studies conducted in France (32-34). Combier et al. (32), reported that a travel time greater than 15 minutes was significantly associated with an increased risk of BBA (16-30 mins vs < 16 mins, adjOR= 1.73, 95%CI: 1.23, 2.46); (31-45 mins vs < 16 mins, adjOR= 1.64, 95%CI: 1.06, 2.54) (32). In a case control study (34) the BBA rate increased six fold when the travel time increased to more than 45 minutes from home to the OU compared to women who travelled less than 15 minutes (> 45 mins vs > 15 mins, adjOR= 6.18 95%CI: 1.33, 28.65). However, in the other case control study the risk of BBA was not significantly increased in women who travelled for greater than 20 minutes (33).

In a study from Norway (40, 41) the risk of BBA increased significantly with longer travel time to the nearest OU from home. Women who travelled more than two hours had an eight fold increased risk of BBA compared to women who lived within one hour of the nearest OU (>2hrs vs <1 hr, cOR= 8.21, 95%CI: 7.19, 9.37) (41).

The studies from Canada (43, 44) found a significant increase in BBA in women living in communities greater than one hour travel time from an OU compared to those living less than one hour away. In Grzybowski et al. (43), women who lived one to two hours from an OU had the highest risk of BBA compared to less than one hour (adjOR= 6.41, 95%CI: 3.69, 11.28) and women who lived greater than four hours away also had increased risk compared to those living less than one hour away (adjOR: 3.63, 95%CI: 1.40,9.40); however there was no difference between those who lived 2-4 hours from an OU and those living less than one hour away (adjOR=0.92, 95%CI: 0.22, 3.88). Gryzbowski et al. (44), found a five-fold increase in BBA in women who lived more than an hour

For peer review only - http://bmjopen.bmj.com/site/about/guidelines.xhtml

away from an OU in comparison to women who lived less than an hour away (cOR=5.06, 95%CI: 3.82,6.70).

Neonatal unit admission (NNU)

Three studies from Canada reported on NNU admission (42-44). The two studies from British Columbia (43, 44) reported NNU depending on whether the admission was for Level 2 care (High Dependency) or Level 3 care (Intensive Care). Findings from one of these studies (43), showed NNU level 2 admission increased significantly in babies born to women living more than one hour away from an OU compared to less than one hour (adjOR = 2.20, 95%CI: 1.59, 3.05). For those living 2-4 hours away, Level 2 admissions were significantly lower compared with those living less than 1 hour away (adjOR=0.31, 95%CI: 0.14,0.65). For those living more than 4 hours away there appeared to be no increase in NNU level 2 admission. For level 3 NNU admission, a significantly increased risk was found for the 1-2 hour category (1-2 hrs vs < 1 hr, cOR= 2.72, 95%CI: 1.46, 5.09). For the other two categories, 2-4 and >4 hours, neither crude nor adjusted analyses showed any significant difference. The number of women in each group decreased with increasing time from an OU. In Grzybowski et al. (44), there was no increased risk of admission to NNU level 2 in babies born to women living more than one hour from an OU compared to less than one hour, however admission to NNU level 3 was significantly higher (cOR = 1.50, 95%CI: 1.01, 2.23). The third Canadian study from Ontario (42), showed a lower relative risk of NNU admission for planned home births with a travel time greater than 30 minutes when compared to less than 30 minutes (adjRR = 0.6, 95% CI: 0.44, 0.81).

Apgar score

Two studies reported on Apgar Score; one from Canada and one from the Netherlands (37, 42). In the Canadian study (42) no significant difference was found for Apgar score <7 at five minutes

For peer review only - http://bmjopen.bmj.com/site/about/guidelines.xhtml

between women who planned home birth and lived more or less than 30 minutes away from an OU, either for nulliparous or multiparous sub-groups (adjRR =1 .02, 95%CI: 0.95, 1.10).

The study from the Netherlands (37) used a composite outcome of mortality and/or Apgar < 4 at 5 minutes and/or transfer to NNU, and showed a small but significant increase in this outcome in women whose travel time to an OU exceeded 15 minutes (15-20 mins vs <15 mins adjOR=1.11 95%CI: 1.02, 1.21 and >=20 mins vs <15 mins, adjOR= 1.27, 95%CI: 1.17, 1.38).

Hypoxic-Ischemic Encephalopathy (HIE) – No studies reported this outcome.

Discussion

This review describes studies which have explored the associations between OU closure, distance or travel time to an OU, and maternal and neonatal outcomes. The included studies were conducted in the UK, France, the Netherlands, Norway, Canada and Japan. Many studies were from parts of the world where service configuration varied and the study populations were sometimes dispersed over a large geographical area. The included studies differed in their design, geographical boundaries, outcomes measures used, and included a wide range of travel time/ distance thresholds used. In addition, although many studies reported that potential confounders were adjusted for in their analyses, many of the outcomes of interest for this review were crude measures of effect without adjustment. Therefore, comparing these studies with each other was a challenge.

All of these studies were brought together to explore whether women who had to travel longer and further to their planned OU were at increased risk of adverse outcomes. There was one reasonably consistent finding which was that there appeared to be an increased risk of BBA the longer it took to reach the OU. This may have been associated with an increased risk for the baby with a suggestion of an increased risk of perinatal or neonatal mortality in some studies, however, this effect was not consistent across all the studies. There was also an increase in CS rates following closure of an OU

For peer review only - http://bmjopen.bmj.com/site/about/guidelines.xhtml

and with shorter travel distance and time, however it is unclear if the difference was related to the exposure or unmeasured differences in CS rates.

Strengths and limitations of the review

This work is the first to synthesise systematically the current evidence relevant to obstetric unit closure and the impact of travel time and travel distance on maternal and neonatal outcomes. Rigorous systematic review methodology was applied, including a sensitive search strategy to identify all the relevant literature, and thorough assessment of potential risks of bias. All screening, data extraction and risk of bias assessment were performed independently by at least two reviewers.

The process of selecting studies for inclusion was challenging due to a lack of reporting of some details, for example, it was not always clear which level of maternity services the study referred to, in others, findings related to the impact of travel time and distance were not always presented despite this being described as a study objective.

Interpretation of findings

It is difficult to conclude from this review whether reconfiguration of maternity services, with closure of OUs, resulting in increased travel distances and times for women is unequivocally associated with worse outcomes for the mother or the baby. Assessing the impact of OU closure and prolonged travel time and distance is not straightforward; to isolate the impact of the closure and travel time and distance on maternal and neonatal outcomes we need to fully understand the models of maternity care, transport services, landscape characteristics, women's satisfaction with care and places of birth available to women in that specific geographical area. Understanding how services are delivered to women is vital when assessing the impact of travel distance and time as services may be adapted to meet the challenges for women living in remote areas , for example by

For peer review only - http://bmjopen.bmj.com/site/about/guidelines.xhtml

transferring women antenatally a few weeks before birth. Some studies found an increase in CS rates with shorter distance/travel time. Attributing this solely to closure or reconfiguration of services is problematic as simple analytical comparisons of rates before and after changes do not account for underlying time trends. Future studies might want to consider an interrupted time series design as a more appropriate method.

There remains an urgent need to evaluate the impact of changing maternity service provision. The imperative to close and consolidate OUs into larger units is based on a belief that this will improve safety for both mother and baby. If increasing travel times and distances increases risks to mothers and babies, then the postulated benefits of larger OUs could be offset by the harms of the reconfiguration.

Waiting for closure of OUs to prospectively evaluate the impact on the surrounding maternity population will always be challenging. However, exploring the existing impact of distance and travel time from home to an OU may be a reasonable approach to explore what the impact of reconfiguration may be for a proportion of the women in the area served by the OU which would have these parameters increased by closure of one of more local OU(s). Such a study would need to be large to explore the impact of travel time and distance on substantive harms such as mortality for the baby, so will almost certainly need to use routinely collected data to obtain large numbers. Such studies will also need to include vigorous evaluation of confounders, such as maternal characteristics, socioeconomic status and maternal medical history, which are known to influence birth outcomes; controlling for these factors is vital to determine the OU closure impacts. These studies should also collect data at multiple time points after the closure and apply statistical analysis which considers time-varying relationships and the outcomes.

Measurement of travel time and distance from the woman's place of residence to an OU would also need more sophisticated approaches than previously used in many studies; for example the use of

For peer review only - http://bmjopen.bmj.com/site/about/guidelines.xhtml

web-based route planners and adjustment for travel conditions rather than using straight line distances or relying on self-reports.

Many study designs assume that travel time and distance have a constant effect on outcomes. If local OUs are far away, it is possible that women will modify their behaviour in relation to when they set off for their OU in labour, if they know they have an hour's journey compared with a 20 minute journey. The extent to which this will mitigate the effects of longer travel times would not be seen in a study looking at existing travel times and distances.

Conclusion

Given the substantial variation across studies we were unable to draw firm conclusions regarding the association between OU closure, travel distance or time to obstetric services and maternal and neonatal outcomes. There appears to be a consistent association with BBA with increasing distance and travel time to an OU and a suggestion of increasing risk to the baby. However, few studies have rigorously controlled for potential confounders.

Funding source: This research is funded by the National Institute for Health Research (NIHR) Policy Research Programme, conducted through the Policy Research Unit in Maternal Health and Care, 108/0001. The views expressed are those of the author(s) and not necessarily those of the NIHR or the Department of Health and Social Care.

Competing Interests: None of the authors has any competing interest.

Acknowledgment: Our thanks to Pamela White for contacting NHS Trusts and obtaining papers, Nia Roberts for development of the search strategy and Mark Willett and Fiona Mackie for providing unpublished data.

Authors' contributions

PB, FA, RM and JD conceived the research. All authors developed the protocol and RM developed the search strategy. RM, CT, AP, FA and JH screened the search results and full papers. RM, CT, JH,

For peer review only - http://bmjopen.bmj.com/site/about/guidelines.xhtml

FA and CO assessed the quality of included papers, extracted the data and synthesised the results.

RM and FA drafted the manuscript and all authors agreed the final manuscript.

Data sharing statement

All the data included in this systematic review are in the public domain.

For peer review only

Tables:

Table 1A: Description of included studies – Obstetric unit closures

Author, year, Country	Study design & setting	Study objectives	Study period	Eligibility criteria	Participant characteristics	Description of exposure (change over time)	Services context information	Review Outcomes	
								Perinatal	Maternal
**UK STUDIES**									
Fleming/East Lancashire study									
Fleming 2013 (15), UK (abstract)	Retrospective analysis pre- and post-service reconfiguration<br><br>East Lancashire maternity services	To examine the effect of major service reconfiguration on CS rates	Time of reconfiguration: Nov 2010<br>Time of analysis: Jan-Jun 2010 vs Jan-Jun 2012	NR	N: NR<br><br>Characteristics: NR	Service reconfiguration<br>Pre-change: 2 OUs<br>Post-change: 1 OU + 3 MW-led units	Universal state provision of maternity care. Approx 7000 births /yr at the new unit.	NR	CS
East Lancashire Hospitals NHS Trust 2017, UK (unpublished-data)	Retrospective population-based analysis of routinely collected data of service reconfiguration	To review outcomes after centralisation of services on the Burnley general hospital site	Time of reconfiguration: Nov 2010<br>Time of analysis: 2009-2017	NR	2009-2017<br>N=53,870 births (2010 data excluded)<br><br>Characteristics: NR	Amalgamation of 2 obstetric units<br>Pre-change: 2 OUs<br>Post-change:	Universal state provision of maternity care. Changes driven by pressure on	BBA; SB; NM	NR

For peer review only - http://bmjopen.bmj.com/site/about/guidelines.xhtml

	East Lancashire, Blackburn and Burnley					1 OU plus 2 birth centres	staff rotas, European Working Time Directive, and desire to maintain high quality service. 6000-7000 births/yr at new unit.		
Mackie 2014 (16), UK (abstract & (unpublished data)	Retrospective analysis pre- and post- service reconfiguration Pennine Acute Trust (PAT): Royal Oldham Hospital (ROH), North Manchester General Hospital (NMGH) and Fairfield General Hospital (FGH)	To assess the effect of the amalgamation of 2 OUs to form a "super-centre" with increased consultant labour ward cover	Time of OUs amalgamation 2011 Time of analysis: pre-changes 2010 - 2011 vs. post changes 2011 - 2013	NR	Pre-amalgamation N=5422 Post-amalgamation N=5046<br><br>Characteristics : NR	Service reconfiguration<br><br>Pre-change: 2 OUs Post-change: 1 OU	Universal state provision of maternity care. Approx 5000 births /yr at the new unit.	SB; NM; BBA	Maternal mortality; Emergency CS; 3rd& 4th degree perineal tea; Maternal transfer to ICU
**OTHER EUROPEAN STUDIES**									
Blondel* 2011 (5), France	Retrospective population-based analysis of routinely	To report on BBA incidence in relation to distance from OU and the closure	Time of OU closure: 2003 and 2006 Time of analysis:	*Included:* Singleton births *Excluded:*	N=1,349,751 births; OoH n=5740 *N Births 1,349, 751*	Closure of maternity unit Pre-change: Number of OUs NR	Centralisation of births in larger units due to safety concerns,	BBA	NR

32

For peer review only

	collected data, OU closure<br><br>Across France	impact on different socio-demographic groups	2005-2006	Municipalities if >8% missing data, or high OoH rates. Departments excl. if >20% births already excl.	Age (yrs) (n):<br><20 - 26,152<br>20-24 - 188,350<br>25-29 - 427,462<br>30-34 - 442,089<br>35-39 - 213,534<br>40+ - 52,164<br><br>Nullip (n) 774,460<br>SES: occupation professional (n) 217,045<br>intellectual 325,746<br>admin 266,000<br>retail 122,727<br>skilled 49,201<br>unskilled 84,664<br>none 184,368<br>Ethnicity, education: NR	Post-change: Closure of units within 15km radius of home, number of units closed NR	financial pressure, efficiency savings, and staff shortage		
Hemminki 2011 (18), Finland	Retrospective population-based analysis of routinely collected data, OU closure	To describe centralisation trend, unplanned out of hospital births, perinatal mortality, health and birth	Time of OU closure: 1991-2008<br>Time of data analysis: Finland 1991-2008;	*Inclusion*: All births<br><br>*Exclusion*: NR	1991-2008 N=474,419<br><br>Characteristics: NR	Centralisation of births, maternity units number declined	Universal access to maternity care, minimal private care. Pre- and postnatal care	BBA	NR

For peer review only - http://bmjopen.bmj.com/site/about/guidelines.xhtml

	Across Finland and a specific district Uusimaa	outcomes in areas served by different levels hospitals	Uusimaa district 2004-2008			Pre-change: 49 OUs in 1991<br>Post-change: 34 OUs in 2008	decentralised, birth hospital-based service, care of high-risk pregnancies centralised.<br>Mean no. births/hospital increased from 1339-1733 over study period.		
Engjom 2014 (7), Norway	Retrospective population-based study, 3 cohort and 2 cross-sectional studies*, OU closure*<br><br>Across Norway	To assess the availability of OUs, the risk of unplanned delivery outside OU and maternal morbidity	Cohort: 1979-2009<br>Cross-sectional: 2000 and 2010<br>Time of OUs closure: 1979-2009<br><br>Time of analysis:<br>Cohort: 1979-2009<br>Cross-sectional: 2000 and 2010	*Included:* Age 15-49 yrs, known place of birth, GA ≥22 wks and/or bthwt ≥500g<br>*Excluded:* Missing maternal address, planned home birth	1979-83 n=252,621<br>2004-09 n=409,432<br><br>Characteristics : NR	Declined in number of OUs in Norway<br>Pre-change: 95 OUs in 1979<br>Post-change: 51 OUs in 2009	Universal access to maternity care; relatively dispersed population	Perinatal mortality (PM)	CS
Grytten 2014 (17), Norway	Retrospective population-based analysis of routinely collected data, OU closure	To study whether neonatal and infant mortality were independent of the type of hospital in which	Time of closures: between 1979 and 2005<br>Time period for analysis: 5 years pre-and post-	*Inclusion*: All births<br><br>*Exclusion*: NR	N=33,677<br><br>Characteristics : NR	Centralisation /OU closures<br>Number of local hospitals fell from 43 to 26 between 1979 and 2005. 17 maternity wards	Universal free access to maternity care. Low-risk births in local hospitals, high risk in central/ regional	NM; Infant mortality (IM)	NR

For peer review only

For peer review only - http://bmjopen.bmj.com/site/about/guidelines.xhtml

	Across Norway	the delivery was carried out	closure for each hospital			in local hospitals closed<br>Pre-change:<br>22 hospitals with neonatal department; 43 local hospitals; 30 maternity clinics<br><br>Post-change:<br>22 hospitals with neonatal department; 26 local hospitals, 10 maternity clinics	hospitals. Births in Central/Regional hospitals increased from 65-81% over study period.		
**CANADIAN STUDIES**									
Le Coutour 1990 (21), Canada	Retrospective population-based analysis of routinely collected data, OU closure<br><br>Montreal metropolitan area	To describe the evolution of obstetric practice in Montreal metropolitan area before/ after closure of units	Time of closure: 1984-1985<br>Time of analysis: 1981-1985	*Inclusion*: All births<br><br>*Exclusion*: NR	1981-1984<br>N=128,688<br><br>Characteristics: NR	7/13 university hospitals, 5/13 specialist hospitals, 4/13 smaller units closed<br>Pre-change: 39 units in 1981<br>Post-change:16	State provision of maternity care. Closures due to budgetary restrictions.	NR	Overall CS
Allen 2004 (22), Canada	Retrospective population-based analysis of routinely collected data, OU closure	To evaluate the effect of hospital closures on critical obstetrical interventions and perinatal	Pre-closure: 1988-1993<br><br>Post-closure: 1996-2002	*Inclusion*: All births<br><br>*Exclusion*: Delivery <20 weeks;	1988-93 n=69,213<br>1996-2002 n=63,510<br>*Range %*	1988-1993 =27 hospitals<br>1996-2002 =19 hospitals	State provision of maternity care. Reduction in number of	SB; Fetal/ neonatal mortality (NM)	NR

	Eastern, Northern, Western, and Central in Nova Scotia	outcomes in rural communities	Time of analysis: 1988 – 1993 vs. 1996 - 2002	bthwt <500g; triplets+; major congenital anomaly	Age >34 yrs: 5.6-14.8; Nullip 39.4-46.8 Twins 1.0-1.3<br><br>Ethnicity, socioeconomic status, education: NR	Reduction in maternity units from 42 to 11 between 1970 and 2002 Pre-change: 42 units in 1970 Post-change: 11 units in 2002	units and physicians due to financial constraints and difficulty maintaining clinical competence and confidence.		
Hutcheon 2017 (20), Canada	Retrospective population based analysis of routinely collected data, OU closure<br><br>25 communities within British Columbia, Canada	To examine the effect of obstetric service closures on intrapartum outcomes	1998-2014 Time of closures: between 2000 and 2012<br><br>Time of analysis: 1998-2014	*Inclusion*: All births recorded in British Columbia Perinatal Data Registry (99% of deliveries) *Exclusion*: Communities close to larger metropolitan areas and or uncertainty about dates of service closures.	*Pre-closure* N=5796 Median maternal age 27 yrs (IQR 23-31); Nullip 39.3%<br><br>*Post-closure* N=6153 Median maternal age 28 yrs (IQR 24-32); Nullip 40.7% Ethnicity, Socioeconomic status, education: NR	Centralisation /OU closures Between 1998 and 2014 one-third of hospitals stopped providing maternity services Pre-change: 21 hospitals with obstetric services<br><br>Post-change: Obstetric services closed in same 21 hospitals	State provision of maternity care. Centralisation of obstetric services, majority of hospital closures in low-volume hospitals	BBA; Perinatal / Neonatal mortality ; NNU admission	Overall CS; Maternal mortality; 3rd/4th degree perineal tear, blood transfusion, maternal admission to ICU

Abbreviations: Approx: approximately; bthwt: Birth Wight; BBA: Born before arrival; CS: Caesarean Section; excl: Excluded; ITU: Intensive Care Unit; IQR: Interquartile Range; MW: Midwife; NHS: National Health Services; NNU: Neonatal Unit; NM: Neonatal Mortality, NR: Not Reported; Nullip: Nulliparous; OU: Obstetric unit; SB: Stillbirth; wo: Without; yr: Year. *Blondel 2011 is also included in travel distance.

For peer review only - http://bmjopen.bmj.com/site/about/guidelines.xhtml

Table 1B: Risk of bias - Obstetric unit closure studies

Author, year, country	Study sample selection bias	Bias in measurement of exposure	Bias in measurement of outcomes	Attrition bias	Analysis method reported and appropriate	Closure independent of other changes over time	Potential confounders adjusted for and listed
**UK STUDIES**							
Fleming/East Lancashire study							
Fleming 2013 (15), UK (abstract)	**LOW** All births in East Lancashire Maternity Services catchment	**LOW** All births in catchment area affected by the closure Number of OUs closed reported	**LOW** Objective outcome (CS)	**UNCLEAR** Not reported	**UNCLEAR** None reported	**UNCLEAR** None reported	**UNCLEAR** None reported
East Lancashire Hospitals NHS Trust 2017, UK (unpublished data)	**UNCLEAR** Unpublished data, no details	**UNCLEAR** Unpublished data, no details	**LOW** Objective outcomes (BBA, SB, NM)	**UNCLEAR** Unpublished data, no details	**UNCLEAR** Unpublished data, no details	**UNCLEAR** Unpublished data, no details	**UNCLEAR** Unpublished data, no details
Mackie 2014 (16), UK (abstract & unpublished)	**LOW** Data from Maternity Information System	**LOW** All births in catchment area affected by the closure. Number of OUs closed reported	**LOW** Objective outcomes (SB, NM, BBA, MM, ICU admission, perineal tears)	**UNCLEAR** Not reported	**UNCLEAR** None reported	**UNCLEAR** None reported	**UNCLEAR** None reported

For peer review only - http://bmjopen.bmj.com/site/about/guidelines.xhtml

**OTHER EUROPEAN STUDIES**								
Blondel 2011 (5), France*	**LOW** Data from birth certificates	**LOW** Number of OUs closed reported	**LOW** Objective outcome (BBA)	**LOW** 11% excluded due to missing data	**LOW** Analysis method was described and appropriate, a multi-level model analysis	**UNCLEAR** None reported	**LOW** Adjusted for maternal age, occupational category and rurality
Hemminki 2011(18), Finland	**LOW** Data from Finnish medical birth register	**LOW** All births in catchment area affected by the closure Number of OUs closed reported	**LOW** Objective outcome (BBA)	**LOW** Births with missing information excluded (<0.05%)	**LOW** Analysis method was described and appropriate, a regression model with adjusted analysis	**UNCLEAR** None reported	**LOW** Adjusted for Parity, plurality, age, socioeconomic status and smoking
Engjom 2014 (7), Norway	**LOW** Data from Medical Birth Registry of Norway	**LOW** All births in Norway affected by the closure Number of OUs closed reported	**LOW** Objective outcome (BBA)	**LOW** All units report to Medical Birth Registry	**LOW** Analysis method appropriate, a logistic regression model, crude and adjusted results given	**UNCLEAR** None reported	**LOW** Adjusted for maternal age, parity, education and partner status
Grytten 2014 (17), Norway	**LOW** Data from Medical Birth Registry of Norway	**LOW** All births in Norway Number of OUs closed reported	**LOW** Objective outcomes (NM, Infant mortality)	**LOW** All maternity units report to Medical Birth Registry	**Unclear** Difference-in-difference statistical method used, but reporting of findings were unclear	**LOW** Adjusted for trend in infant outcomes based on local hospitals that were not closed.	**LOW** Maternal age, immigrant status, level of education, marital status, predisposing medical factors and characteristics of the birth

For peer review only - http://bmjopen.bmj.com/site/about/guidelines.xhtml

CANADIAN STUDIES							
Le Coutour 1990 (21), Canada	**LOW** Data from MED-ECHO - regional data collection system	**LOW** All births in catchment area Number of OUs closed reported	**LOW** Objective outcome (CS)	**UNCLEAR** No information	**UNCLEAR** No details on data analysed method	**UNCLEAR** None reported	**HIGH** No adjustment
Allen 2004 (22), Canada	**LOW** Data from Nova Scotia Atlee Perinatal Database	**LOW** All births in catchment area affected by the closure Number of OUs closed reported	**LOW** Objective outcomes (SB, NM)	**UNCLEAR** Population based dataset but no information about missing data	**LOW** Analysis method appropriate and data from logistic regression models were reported	**UNCLEAR** None reported	**LOW** Maternal age, smoking and maternal diseases
Hutcheon 2017 (20), Canada	**LOW** Data from British Columbia Perinatal registry	**LOW** All births in catchment area affect by the closure Number of OUs closed reported	**LOW** Except for 3rd/4th degree tears. Objective outcomes (BBA, PM, NM, ICU admission, CS, MM, blood transfusion, Maternal admission to ICU)	**LOW** >99% complete	**LOW** Used a within-community fixed-effects design and Poisson regression	**LOW** Using difference in difference analysis which separates the effect of the closure from underlying time trends of reported outcomes	**HIGH** No adjustment

Abbreviations: BBA: Born Before Arrival; CS: Caesarean Section; ICU: Intensive Care Unit; MM: Maternal Mortality; NHS: National Health Services; NM: Neonatal Mortality; PM: Perinatal Mortality; SB: Stillbirth. *Blondel 2011 included in travel distance & OU closure.

For peer review only - http://bmjopen.bmj.com/site/about/guidelines.xhtml

Table 1C: Outcomes - Obstetric unit closure

Outcomes	Author, Year, Country	Exposure and comparator groups	Participants (N, n, %)			Findings		
**MATERNAL OUTCOMES**								
**Maternal mortality (MM)**	Mackie 2014 (16), UK (unpublished data)	Before and after amalgamation of 2 OUs	**Year**	**Deliveries (N= 15,349)**	**MM n (%)**	**Year**	**Crude OR (95%CI)**	**Adjusted OR (95%CI)**
			Pre 2010-2011	5354	1 (0.02)	Pre 2010-2011	1	NR
			Post 2011-2013	9995	1(0.01)	Post 2011-2013	*0.54 (0.03, 8.56)*	NR
	Hutcheon 2017 (20), British Columbia, Canada	Before and after OU closure (1998-2014)	**Closure status**	**Deliveries (N=11,949)**	**Maternal deaths n (%)**	**Closure status**	***Crude OR (95%CI)***	***Adjusted OR (95%CI)***
			Preclosure	5796	<5 (<0.09)	Preclosure	NR	NR
			Postclosure	6153	<5 (<0.08)	Postclosure	NR	NR
			No significant difference pre/post closure in adverse events during labour and delivery					
**Caesarean section (CS) (overall or intrapartum)**	Fleming 2013 (15), UK (abstract)	Before and after closure of OU in 2010	***Closure status***	***Deliveries (N=NR)***	***CS* n (%)**	**Closure status**	**Crude OR (95%CI)**	**Adjusted OR (95%CI)**
			Preclosure, early 2010	NR	(NR) 26.1	Preclosure, early 2010	NR	NR
			Postclosure, 2012	NR	(NR) 21.5	Postclosure, 2012	NR	NR

For peer review only - http://bmjopen.bmj.com/site/about/guidelines.xhtml

			Proportions of CS presented with no other data.					
	Engjom 2014 (7), Norway	2000 compared to 2009 during which time number of OUs declined from 47 to 41	**Year**	**Deliveries (N=2,177,934)**	**CS n (%)**	**Year**	**Crude OR (95% CI)**	**Adjusted OR (95%CI)**
			Pre 2000	58,632	7653 (13.10)	Pre 2000	1	NR
			Post 2009	61,895	10,154 (16.41)	Post 2009	*1.31 (1.27, 1.35)*	NR
	Le Coutour 1990 (21), Quebec, Canada	Before and after closure of OUs between 1982 and 1983	**Year**	**Deliveries (N= 64,274)**	**CS n (%)**	**Year**	**Crude OR (95% CI)**	**Adjusted OR (95%CI)**
			Pre 1981	32,807	5852 (17.84)	Pre 1981	1	NR
			Post 1984	31,467	6214 (19.7)	Post 1984	*1.13 (1.09, 1.18)*	NR
	Hutcheon 2017 (20), British Columbia, Canada	Before and after OUs, closure (1998-2014)	**Closure status**	**Deliveries (N= 11,949)**	**CS n (%)**	**Closure status**	**Crude OR (95%CI)**	**Adjusted OR (95%CI)**
			Preclosure	5796	1387 (23.93)	Preclosure	1	NR
			Postclosure	6153	1579 (25.70)	Postclosure	*1.10 (1.01, 1.19)*	NR
**Emergency caesarean section (CS)**	Mackie 2014 (16), UK (abstract only)	Before and after amalgamation of two OUs	**Year**	**Deliveries (N=15,349)**	**Emergency CS n(%)**	**Year**	**Crude OR (95%CI)**	**Adjusted OR (95%CI)**
			Pre 2010-2011	5354	739 (13.80)	Pre 2010-2011	1	NR
			Post 2011-2013	9995	1322 (13.23)	Post 2011-2013	*0.95 (0.86, 1.05)*	NR
**Severe perineal trauma (3rd or 4th**	Mackie 2014 (16), UK (unpublished data)	Before and after amalgamation of two OUs	**Year**	**Deliveries (N=15,349)**	**3rd & 4th n (%)**	**Year**	**Crude OR (95%CI)**	**Adjusted OR (95%CI)**
			Pre 2010-2011	5354	133 (2.48)	Pre 2010-2011	1	NR

For peer review only - http://bmjopen.bmj.com/site/about/guidelines.xhtml

**degree tear)**			Post 2011-2013	9995	276 (2.76)	Post 2011-2013	*1.11 (0.90, 1.37)*	NR
	Hutcheon 2017 (20), British Columbia, Canada	Before and after OU closure (1998-2014)	**Closure status**	**Deliveries (N=11,949)**	**3rd or 4th degree tear n (%)**	**Closure status**	**Crude OR (95%CI)**	**Adjusted OR (95%CI)**
			Preclosure	5796	136 (2.40)	Preclosure	1	NR
			Postclosure	6153	174 (2.82)	Postclosure	*1.21 (0.96, 1.52)*	NR
**Postpartum haemorrhage**	No studies							
**Maternal admission to ICU**	Mackie 2014 (16), UK (abstract only)	Before and after amalgamation of two OUs	**Year**	**Deliveries (N=15,349)**	**n (%)**	**Year**	**Crude OR (95%CI)**	**Adjusted OR (95%CI)**
			Pre 2010-2011	5354	18 (0.34)	Pre 2010-2011	1	NR
			Post 2011-2013	9995	27(0.27)	Post 2011-2013	*0.80 (0.44, 1.46)*	NR
	Hutcheon 2017 (20), British Columbia, Canada	Before and after OU closure (1998-2014)	**Closure status**	**Deliveries (N= 11,949)**	**ICU admission n**	**Closure status**	**Crude OR (95%CI)**	**Adjusted OR (95%CI)**
			Preclosure	5796	<5	Preclosure	NR	NR
			Postclosure	6153	<5	Postclosure	NR	NR
**Maternal Blood transfusion**	Hutcheon 2017 (20), British Columbia, Canada	Before and after OU closure (1998-2014)	**Closure status**	**Deliveries (N= 11,949)**	**Blood transfusion n (%)**	**Closure status**	**Crude OR (95%CI)**	**Adjusted OR (95%CI)**
			Preclosure	5796	53 (0.91)	Preclosure	1	NR
			Postclosure	6153	46 (0.75)	Postclosure	*0.82 (0.55, 1.21)*	NR

For peer review only - http://bmjopen.bmj.com/site/about/guidelines.xhtml

**NEONATAL OUTCOMES**								
**Stillbirth (SB)**	East Lancashire Hospitals NHS Trust, 2017, UK (unpublished data)	Before and after amalgamation of two obstetric units (OUs) in 2010	**Year**	**Deliveries (N= 53,870)**	**SB n (%)**	**Year**	**Crude OR (95%CI)**	**Adjusted OR (95%CI)**
			Pre 2009	6492	75 (1.16)	Pre 2009	1	NR
			Post 2011-2017	47,378	333 (*0.70*)	Post 2011-2017	*0.61 (0.47, 0.78)*	NR
			Stillbirth>24 weeks					
	Allen 2004 (22), Nova Scotia, Canada	Before and after closure of OUs	**Year**	**Deliveries (N=132,723)**	**SB n (%)**	**Year**	**Crude OR (95%CI)**	**Adjusted OR (95%CI)**
			Pre 1988-93	69,213	291 (*0.42*)	Pre 1988-1993	1	NR
			Post 1996-2002	63,510	214 *(0.34)*	Post 1996-2002	*0.80 (0.67, 0.96)*	NR
	Mackie 2014(16), UK (unpublished data)	Before and after amalgamation of 2 OUs	**Year**	**Deliveries (N=15,552)**	**SB n (%)**	**Year**	**Crude OR (95%CI)**	**Adjusted OR (95%CI)**
			Pre 2010-2011	5422	29 (*0.53*)	Pre 2010 -2011	1	NR
			Post 2011-2013	10,130	60 (*0.59*)	Post 2011-2013	*1.11 (0.71, 1.73)*	NR
**Neonatal mortality (NM)**	East Lancashire Hospitals NHS Trust, 2017, UK (unpublished data)	Before and after amalgamation of two obstetric units in 2010	**Year**	**Deliveries (N=53,870)**	**NM n (%)**	**Year**	**Crude OR (95%CI)**	**Adjusted OR (95%CI)**
			Pre 2009	6492	4 (*0.06*)	Pre 2009	1	NR
			Post 2011-2017	47,378	39 (*0.08*)	Post 2011-2017	*1.33 (0.81, 2.17)*	NR
			NM not defined					

For peer review only - http://bmjopen.bmj.com/site/about/guidelines.xhtml

	Mackie 2014(16), UK (unpublished data)	Before and after amalgamation of 2 OUs	**Year**	**Deliveries (N=15,552)**	**NM n (%)**	**Year**	**Crude OR (95%CI)**	**Adjusted OR (95%CI)**
			Pre 2010-2011	5422	6 (*0.11*)	Pre 2010-2011	1	NR
			Post 2011-2013	10,130	9 (*0.90*)	Post 2011-2013	*0.80 (0.29, 2.26)*	NR
	Grytten 2014 (17), Norway	Before and after 13 hospital closures	**Year**	**Deliveries (N=33,677)**	**NM n (%)**	**Year**	**Crude OR (95%CI)**	**Adjusted OR(95%CI)**
			5 yrs before	16,297	NR	5 yrs before	NR	NR
			5 yrs after	17,380	NR	5 yrs after	NR	NR
			No statistically significant difference					
**Perinatal mortality (PM)**	Engjom 2014 (7), Norway	2000 compared to 2009 during which time number of OUs declined from 47 to 41	**Year**	**Deliveries (N=2,177,934)**	**PM n (/1000 births)**	**Year**	**Crude OR (95%CI)**	**Adjusted OR(95%CI)**
			Pre 2000	58,632	124 (2.11)	Pre 2000	1	NR
			Post 2009	61,895	99 (1.60)	Post 2009	*0.76 (0.58, 0.98)*	NR
			PM (Intrapartum & neonatal death < 24 hrs, both live & still born)					
	Allen 2004 (22), Nova Scotia, Canada	Before and after closure of OUs	**Years**	**Deliveries (N=132,723)**	**PM n (%)**	**Years**	**Crude OR (95%CI)**	**Adjusted OR (95%CI)**
			Pre 1988-1993	69,213	422 (0.61)	Pre 1988-93	1	NR
			Post 1996-2002	63,510	278 (0.43)	Post 1966-2002	*0.75 (0.64, 0.87)*	*NR*
			Fetal/neonatal mortality not defined					
**Infant mortality**	Grytten 2014 (17), Norway	Before and after 13	**Year**	**Deliveries (N=33,677)**	**IM n (%)**	**Years**	**Crude OR (95%CI)**	**Adjusted OR (95%CI)**

For peer review only - http://bmjopen.bmj.com/site/about/guidelines.xhtml

		hospital closures	5 yrs before	16,297	NR	5 yrs before	NR	NR
			5 yrs after	17,380	NR	5 yrs after	NR	NR
			No significant difference in infant mortality					
**Born before arrival (BBA)**	East Lancashire Hospitals NHS Trust, 2017, UK (unpublished data)	Before and after amalgamation of two obstetric units	**Year**	**Deliveries (N= 53,870)**	**BBA n (%)**	**Year**	**Crude OR (95%CI)**	**Adjusted OR (95%CI)**
			Pre 2009	6492	25 (0.39)	Pre 2009	1	NR
			Post 2011-2017	47,378	341 (0.72)	Post 2011-2017	*1.88 (1.25, 2.82)*	NR
	Mackie 2014 (16), UK (unpublished data)	Before and after amalgamation of 2 OUs	**Year**	**Deliveries (N=15,349)**	**BBA n (%)**	**Year**	**Crude OR (95%CI)**	**Adjusted OR (95%CI)**
			Pre 2010-2011	5354	11 (0.21)	Pre 2010-2011	1	NR
			Post 2011-2013	9995	26 (0.26)	Post 2011-2013	*1.28 (0.63, 2.60)*	NR
	Blondel 2011 (5), France	OU closure within 15 km radius 2003-2006	**Yrs 2003-2006**	**Deliveries (N=1,349,751)**	**BBA n (/1000 births)**	**Yrs 2003-2009**	**Crude OR (95%CI)**	**Adjusted OR (95%CI)**
			No closure	1,001,858	4531 (4.52)	No closure	1	1
			Closure within 15km radius	347,893	1209 (3.47)	Closure within 15km radius	0.77 (0.72, 0.82)	0.91 (0.84, 1.00)
	Engjom 2014 (7), Norway	1979-83 compared to 2004-09, number of emergency OUs declined from 47 to 41	**Year**	**Deliveries (N=662,053)**	**BBA n (%)**	**Year**	**Crude OR (95%CI)**	**Adjusted OR (95%CI)**
			Pre 1979-83	252,621	984 (0.39)	Pre 1979-83	1	1
			Post 2004-09	409,432	2832 (0.69)	Post 2004-09	1.8 (1.6, 1.9)	2.0 (1.9, 2.2)

For peer review only - http://bmjopen.bmj.com/site/about/guidelines.xhtml

	Hemminki 2011(18), Finland	Centralisation of hospitals over years 1991-2008	**Year**	**Births (N)**	**Unplanned BBA n(/1000)**	**Planned or unplanned BBA n(/1000)**	**Year**	**Crude OR (95%CI)**	**Adjusted OR (95%CI)**
			Pre 1991	65,632	-	68 (1.0)	Pre 1991	1	NR
			Post 2004-2008	56,873	222 (3.76)	243 (4.1)	Post 2004-2008	*4.14 (3.16, 5.41)*	NR
			Total N (1991-2008)= 122,505						
	Hutcheon 2017(20), British Columbia, Canada	Before and after OU closure (1998-2014)	**Closure status**	**Deliveries (N=11,949)**	**Unplanned BBA n (%)**	**Closure status**	**Crude OR (95%CI)**	**Adjusted OR (95%CI)**	
			Preclosure	5796	30 (0.5)	Preclosure	1	NR	
			Postclosure	6153	109 (1.8)	Postclosure	*3.47 (2.31, 5.20)*	NR	
**Neonatal unit admission (NNU) >2 days or transfer within 24 hrs of birth to ICU facility for newborn >/=2500g**	Hutcheon 2017(20), British Columbia, Canada	Before and after OU closure (1998-2014)	**Closure status**	**Deliveries (N=11,949)**	**NNU admission n (%)**	**Closure status**	**Crude OR (95%CI)**	**Adjusted OR (95%CI)**	
			Preclosure	5796	68 (1.17)	Preclosure	1	NR	
			Postclosure	6153	28 (0.46)	Postclosure	*0.39 (0.25, 0.60)*	NR	
**Apgar score (5 minute Apgar score <7)**	Hutcheon 2017(20), British	Before and after OU closure (1998-	**Closure status**	**Deliveries (N=11,949)**	**5 min Apgar score <7 n (%)**	**Closure status**	**Crude OR (95%CI)**	**Adjusted OR (95%CI)**	
			Preclosure	5796	71 (1.22)	Preclosure	1	NR	

For peer review only

For peer review only - http://bmjopen.bmj.com/site/about/guidelines.xhtml

	Columbia, Canada	2014) compared to communities unaffected by closure	Postclosure	6153	85 (1.28)	Postclosure	*1.13 (0.82, 1.55)*	NR
**Hypoxic Ischaemic Encephalopathy (HIE)**	No studies							

Abbreviations: aOR: Adjusted Odds Ratio; BBA: Born Before Arrival; ICU: Intensive Care Unit; NHS: National Health Services; PM: Perinatal Mortality; OR: Odds Ratio; OU: Obstetric Unit.

For peer review on

Table 2A: Description of included studies – Travel distance

**Author, year, country**	**Study design & setting**	**Study objectives**	**Study period**	**Eligibility criteria**	**Participant characteristics**	**Description of exposures Travel Distance**	**Services context information**	**Review Outcomes**	
								**Perinatal**	**Maternal**
**UK STUDIES**									
Bhoopalam 1991 (23), UK	Case-control study, 2 OUs	To establish BBA prevalence and women at risk of BBA, and	1983 – 1987	*Included cases:* Women and their BBA babies	N(BBAs) =137, 1 twins<br>All N= 398<br>Age yrs (n):	Distance (km):<br><2<br>2-7	Universal state provision, 2 units 6 miles apart, serving	BBA	NR

For peer review only - http://bmjopen.bmj.com/site/about/guidelines.xhtml

		morbidity and mortality associated with BBA births		*Included controls:* Two controls for each BBA case, one random (next born in the same hospital), one matched (next born in same hospital matched by GA and BW) *Excluded:* BW < 500g	<21 (69) 21-35 (339) >35 (27) Nullips (107) Ethnicity (n): European(191) Asian (101) Other (16) SES & education: NR	>7	rural areas of Warwickshire		
**OTHER EUROPEAN STUDIES**									
Pasquier 2007(24), France	Retrospective, population-based cohort, 3 Level-III maternity wards with neonatal surgical centre, Rhône-Alpes Region	To examine maternal origin, distance to the nearest maternity ward with a neonatal surgical centre, on perinatal diagnosis, elective termination of pregnancy, delivery in an adequate place and neonatal mortality for pregnancies	1990-95 & 1996-2000 (2 periods separated due to changes in prenatal screening)	*Included:* Fetuses with omphalocele, gastroschisis, diaphragmatic hernia or spina bifida that required surgical repair *Excluded:* Chromosomal anomalies fetuses and babies without anomalies	N =706 infants N= 554 (analysed) Age: yrs (n, %) <21 (15, 2) 21-35 (550, 82) >35 (106, 16) Ethnicity: (n,%) Western European (393, 76) Non-Western European (124, 24) Parity, SES & education: NR	Distance (km): <11 11-50 >50	Distance to Level III maternity ward with NNU and a neonatal surgical centre, there were 3 in the Rhone-Alps Region	Neonatal Mortality (NM)	NR

For peer review only - http://bmjopen.bmj.com/site/about/guidelines.xhtml

		with severe malformations							
Blondel 2011 (5), France*	Retrospective cohort, population based study, metropolitan France	To calculate the incidence of BBA birth in relation to distance from maternity units and the impact of recent closure on different sociodemographic groups	2005-2006	*Included:* Singleton live births *Excluded:* Municipalities with >8% missing data, unrealistically high BBA births, Departments were excl. if >20% births already excl.	N=1,517,599 livebirths N=1,349,751(analysed) Age yrs - n <20 - 26,152 20-34 – 1,057,90135-39 - 213,534 40+ - 52,164 Parity: Nullip n - 774,460 SES n: Occupation professional-217,045 Intellectual-325,746 Admin- 266,000 Retail-122,727 Skilled- 149,201 Unskilled- 84,664 None- 84,368 Ethnicity & education: NR	Distance (km): <5 5-14 15-29 30-44 45+	Centralising births in larger units	BBA; BBA by parity	NR
Pilkington 2014 (25), France	Retrospective, population-based cohort study, French National Vital Statistics registry from	To investigate the impact of distance to closest maternity unit on perinatal outcomes	2001-2008, Stillbirth data 2002-2005	*Included:* All births *Excluded:* *NR*	N= 3,086,128 all births N=3,085,839(analysed) Age yrs (n) <25 - 494,689 25-34 – 2,008,320	Distance (km): <5 5-14 15-29 30-44 ≥45	1998 to 2003, 20% of maternity units closed Mean distance to	Stillbirths (SB); NM	NR

For peer review only - http://bmjopen.bmj.com/site/about/guidelines.xhtml

	mainland France				35-39 - 469,975<br>40+ - 113,144<br>Singleton pregnancy N= 2,988,169<br>Multiple pregnancy: N= 97,959<br>Parity, ethnicity and socioeconomic status & education: NR		nearest maternity unit increased (6.6–7.2 km)		
Ovaskainen 2015 (27), Finland	Case–control study, one centre, Tampere University Hospital	To establish if BBA births increased over time, to identify risk factors associated with BBAs, also if BBAs babies were more prone to neonatal morbidities compared to those delivered in hospital	1996 - 2011	*Included Cases:*<br>Planned and unplanned BBA<br>*Included Controls:*<br>2 controls for infant and mother for each BBA case<br>***Excluded:***<br>BBA with no information whether planned or unplanned	Cases:<br>BBAs (n = 67 births): Age yrs (mean, SD) (range)- 29.0, 5.9 (15-47)<br>Parity 1 (0-16)<br>Controls:<br>N= 134<br>Plurality, ethnicity, education & SES: NR.	Distance (km):<br><35<br>≥35	Tampere University Hospital is the catchment area for 23 municipalities, 521700 residents 5000 births/yr	BBA	NR
Fougner 2000 (26), Norway	Case-control study, 14 municipalities , Oppland County	To compare the experience and care of women who delivered during	1989 - 1997	*Included Cases:*<br>Women who delivered their babies before	N=202<br>Cases: N= 115 BBA women<br>Parity n (%)-<br>Nullips 15 (13%)	Distance (km):<br>< 12.88<br>≥12.88	Oppland county: 4 hospitals	BBA	NR

For peer review only - http://bmjopen.bmj.com/site/about/guidelines.xhtml

For peer review only

		transport to hospital and women who delivered an hour after arriving to hospital		arriving at hospital<br>*Included Controls:* Women who delivered their babies with one hour after arriving at hospital<br>*Excluded:* NR	Controls: N=87 women<br>Parity- Nullips 18 (20%)<br>Age, ethnicity, education & SES: NR				
**CANADIAN STUDIES**									
Lisonkova 2011 (28), Canada	Retrospective population-based cohort study, British Columbia	To examine the association between rural residence and birth outcomes in older mothers	1999 - 2003	*Included:* Singleton mothers aged 35+<br>*Excluded:* Women with missing postcodes, babies with congenital anomaly	N= 29,698 women<br>Age >35 yrs<br>Parity n (%):<br>Nullip 87,733 (0%)<br>Low SES (n, %)<br>(4385 14% 22.6 vs 3,615, 13.7)<br>Ethnicity n(%)<br>1st nation 826 (2,8%) Education: NR	Distance (km):<br><50<br>50-150<br>>150	17 small maternity units (250-2,500 births/yr) closed between 1999 and 2003	SB; Perinatal Mortality (PM); NNU admission ≥1day	All CS; Emergency CS

Abbreviations: BBA: Born Before Arrival; BW: Birth Weight; CS: Caesarean Section; GA: Gestational Age; NNU: Neonatal Unit; Nullip: Nulliparous; SES: Socio Economic Status. *Blondel 2011 is also included in the OU closure.

Table 2B: Risk of bias – Travel distance

**Author, year, country**	**Study sample selection bias** Additional criteria for case control studies	**Bias in measurement of exposure**	**Bias in measurement of outcomes**	**Attrition bias**	**Analysis method reported and appropriate**	**Potential confounders adjusted for and listed**

For peer review only - http://bmjopen.bmj.com/site/about/guidelines.xhtml

**UK STUDIES**								
Bhoopalam 1991 (23), UK	Case definition adequate	**YES** From hospital records	**HIGH** Distance measured in straight line from home address	**LOW** Objective outcome (BBA)	**LOW** 3/134 (2.2%) BBA cases & 10/274 (3.6%) excluded from distance analysis	**HIGH** Descriptive analysis only	**HIGH** No adjustments
	Representative-ness of cases	**YES** BBA cases from 2 hospitals over 5 yrs					
	Appropriate selection of controls	**YES** 2 controls from same hospital					
	Definition of control appropriate	**YES** The outcome (BBA) could not have occurred					
	Comparability of cases and controls	**NO** Significant differences in maternal age, parity, ethnicity & antenatal booking					
**OTHER EUROPEAN STUDIES**								

For peer review only - http://bmjopen.bmj.com/site/about/guidelines.xhtml

Pasquier 2007 (24), France	**LOW** Data from France Central-East malformation registry		**LOW** GIS software used to estimate distance between maternal residence and nearest maternity ward with neonatal surgical centre	**LOW** Objective outcomes (NM)	**LOW** 12 births (0.03%) missing survival data	**LOW** Method detailed and appropriate, univariate analysis & multiple logistic regression were reported	**LOW** Adjusted for parity, ethnicity and other characteristics
Blondel 2011(5), France*	**LOW** Birth certificates		**LOW** GIS software used to estimate distance to hospital from home	**LOW** Objective outcome (BBA)	**LOW** 11% of births excluded	**LOW** Method detailed and appropriate, multilevel model was reported	**LOW** Adjusted for maternal age, occupation, parity and other characteristics
Pilkington 2014 (25), France	**LOW** French National Vital Statistics Registry		**HIGH** Distance calculated from centre of municipality not home address, using road networks provided by the French National Geography Institute	**LOW** Objective outcomes (SB, NM)	**LOW** 10% missing for type of pregnancy and 17% for maternal age	**LOW** Method detailed and appropriate, logistic regression analysis was reported	**LOW** Adjusted for maternal age, plurality, unemployment rate, single parent households
Fougner 2000 (26), Finland	Case definition adequate	**NO** Data from a questionnaire	**HIGH** Data for distance was self-reported	**LOW** Objective outcome (BBA)	**HIGH** Data from women who responded to	**HIGH** Descriptive analysis only	**HIGH** No adjustment

For peer review only

For peer review only - http://bmjopen.bmj.com/site/about/guidelines.xhtml

	Representative-ness of cases	**YES** Cases from 3 hospitals in one county over 8 yrs	in questionnaire		questionnaire		
	Appropriate selection of controls	**Yes** Women who delivered their babies within 1 hour of arriving at hospital					
	Definition of control appropriate	**Yes** Women with no BBA					
	Comparability of cases and controls	**Unclear**					
Ovaskainen 2015 (27), Finland	Case definition adequate	**YES** Medical records	**LOW** Distance from women's home calculated using web-based route planner	**LOW** Objective outcome (BBA)	**High** 13 out-of-hospital deliveries (19%) excluded as could not ascertain whether planned or not planned	**LOW** Method detailed and appropriate, logistic regression data were given	**LOW** Adjusted for single-mother, parity, and other characteristics
	Representative-ness of cases	**YES** Cases from 1 centre, but over 15 yrs					
	Appropriate selection of controls	**YES** Births occurring immediately preceding and following case					

For peer review only - http://bmjopen.bmj.com/site/about/guidelines.xhtml

	Definition of control appropriate	**YES** The outcome (BBA) could not have occurred					
	Comparability of cases and controls	**No** Sig. differences for parity, partnership status, smoking, antenatal visits, labour duration &distance to delivery unit					
**CANADIAN STUDIES**							
Lisonkova 2011 (28), Canada	**LOW** Population based study		**HIGH** GIS used to calculate distance from postcode central point of residence to hospital; mostly using straight line distance	**LOW** Objective outcomes (SB; PM); NNU admission)	**LOW** 492 (1.7%) women excluded due to missing postcodes	**LOW** Method detailed and appropriate, multivariate regression analysis reported	**LOW** Adjusted for parity, single mother, low income, ethnicity and other characteristics

Abbreviations: BBA: Born Before Arrival; GIS Geographical Information System; NM: Neonatal Mortality; NNU: Neonatal Unit; PM: Perinatal Mortality; Sig: Significant; SB: Stillbirth. *Blondel 2011 included in travel distance & OU closure.

For peer review only

For peer review only - http://bmjopen.bmj.com/site/about/guidelines.xhtml

Table 2C: Outcomes – Travel distance

Outcomes	Author, year, country	Exposure groups	Participants (N, n, %)			Findings		
**MATERNAL OUTCOMES**								
**Maternal mortality (MM)**	Ovaskainen 2015 (27), Finland	**Travel distance (km):**<br><35<br>≥35	**Groups**	**N (201)**	**NM n (%)**	No events in either group		
			< 35	NR	0			
			≥35	NR	0			
**Caesarean section (CS) (overall or intrapartum)**	Lisonkova 2011 (28), Canada	**Travel distance (km):**<br><50<br>50-150<br>>150	**Groups**	**N (29,698)**	**CS n (%)**	**Groups**	**Crude OR (95%CI)**	**Adjusted OR (95%CI)**
			<50	27,836	9099 (32.70)	<50	1	NR
			50-150	1534	464 (30.25)	50-150	*0.89 (0.80, 1.00)*	NR
			>50	328	94 (28.70)	>50	*0.83 (0.65, 1.05)*	NR
**Emergency CS**	Lisonkova 2011 (28), Canada	**Travel distance (km):**<br><50<br>50-150<br>>150	**Groups**	**N (9,657)**	**Emergency CS n (%)**	**Groups**	**Crude OR (95%CI)**	**Adjusted OR (95%CI)**
			<50	9,099	5,378 (59.11)	<50	*1*	NR
			50-150	464	258 (55.60)	50-150	*0.87 (0.72, 1.05)*	NR
			>50	94	52 (55.32)	>50	*0.86 (0.57, 1.29)*	NR

**Severe perineal trauma**	No studies							
**Postpartum haemorrhage**	No studies							
**Admission to ICU**	No studies							
**Blood transfusion**	No studies							
**NEONATAL OUTCOMES**								
**Stillbirth (SB)**	Pilkington 2014 (25), France	**Travel distance (km):** <5 5-14 15-29 30-44 ≥45	**Groups**	**N (3,085,839) (2002-2005)**	**SB n (/per 1000)**	**Groups**	**Crude OR (95%CI)**	**Adjusted OR (95%CI)**
			<5	1,404,665	*13,204* (9.4)	<5	1	1
			5-14	81, 1775	*6,657* (8.2)	5-14	*0.87 (0.85, 0.90)*	Reported as RR 0.87 (NR)*
			15-29	648,495	*5,188* (8.0)	15-29	*0.85 (0.82, 0.88)*	Reported as RR 0.85 (NR)*
			30-44	186,537	*1492* (8.0)	30-44	*0.85 (0.81, 0.90)*	Reported as RR 0.85 (NR)*
			≥45	34,367	*306* (8.9)	≥45	*0.95 (0.84, 1.06)*	Reported as RR 0.95 (NR)(NS)
	Lisonkova 2011 (28), Canada	**Travel distance (km):** <50 50-150 >150	**Groups**	**N (29,698)**	**NM n (%)**	**Groups**	**Crude OR (95%CI)**	**Adjusted OR (95%CI)**
			<50	27,836	150	<50	NR	NR
			50-150	1534	NR	50-150	NR	NR

For peer review only - http://bmjopen.bmj.com/site/about/guidelines.xhtml

			>150	328	NR	>150	NR	NR
			OR not reported. Authors noted stillbirth rate was higher among women living 50- 150 km and >150 km versus <50 km, no significant difference found after adjusting for confounders.					
**Neonatal mortality (NM)**	Pasquier 2007(24), France	**Travel distance (km):**<br><11<br>11-50<br>>50	**Groups**	**N (554)**	**NM n (%)**	**Groups**	**Crude OR (95%CI)**	**Adjusted OR (95%CI)**
			<11	239	NR	<11	NR	1
			11-50	156	NR	11-50	NR	0.98 (0.34, 2.88)
			>50	159	NR	>50	NR	1.37 (0.49, 3.86)
	Pilkington 2014 (25), France	**Travel distance (km):**<br><5<br>5-14<br>15-29<br>30-44<br>≥45	**Groups**	**N (6,202,918) (2001-2008)**	**NM n(/per 1000)**	**Groups**	**Crude OR (95%CI)**	**Adjusted RR (95%CI)**
			<5	2,808,068	*7582* (2.7)	<5	1	1
			5-14	1,626,885	*3416* (2.1)	5-14	*0.78 (0.75, 0.81)*	Reported as RR 0.91 (NR) *
			15-29	1,316,329	*2896* (2.2)	15-29	*0.81 (0.78, 0.85)*	Reported as RR 0.94 (NR)(NS)
			30-44	381,288	*801* (2.1)	30-44	*0.78 (0.72, 0.84)*	Reported as RR 0.9 (NR)*
			≥45	69,787	*154* (2.2)	≥45	*0.82 (0.70, 0.96)*	Reported as RR 0.96 (NR)(NS)
			**NM After BBA**			**NM after BBA**		
			**Groups**	**N (6,202,918) (2001-2008)**	**NM n (/per 100,000)**	**Groups**	**Crude OR (95%CI)**	**Adjusted RR (95%CI)**
			<5	2,808,068	*115* (4.1)	<5	1	1

For peer review only - http://bmjopen.bmj.com/site/about/guidelines.xhtml

			5-14	1,626,885	*65* (4.0)	5-14	*0.98 (0.72, 1.32)*	Reported as RR 1.1 (NR)(NS)
			15-29	1,316,329	*72* (5.5)	15-29	*1.34 (0.99, 1.79)*	Reported as RR 1.58 (NR)*
			30-44	381,288	*23* (6.0)	30-44	*1.47 (0.94, 2.30)*	Reported as RR 1.51 (NR)(NS)
			≥45	69,787	*7* (10.0)	≥45	*2.45 (1.14, 5.25)*	Reported as RR 3.68 (NR)*
**Perinatal mortality (PM)**	Lisonkova 2011 (28), Canada	**Travel distance (km):** <50 50-150 >150	**Groups**	**N (29,698)**	**PM n (%)**	**Groups**	***Crude OR (95%CI)***	**Adjusted OR (95%CI)**
			<50	27,836	221 (0.80)	<50	*1*	1
			50-150	1534	19 (1.24)	50-150	*1.57 (0.98, 2.51)*	1.53 (1.10, 2.12)
			>150	328	8 (2.44)	>50	*3.12 (1.53, 6.38)*	3.06 (2.20, 4.24)
**Infant mortality (IM)**	No studies							
**Born before arrival (BBA)**	Bhoopalam 1991 (23), UK	**Travel distance (km):** <2 2-7 >7	**Groups**	**N (398) Cases & controls**	**BBA cases n (%)**	**Groups**	**Crude OR (95%CI)**	**Adjusted OR (95%CI)**
			<2	*59*	4 *(6.80)*	<2	*1*	NR
			2-7	*249*	88 *(35.34)*	2-7	*7.52 (2.64, 21.43)*	NR
			>7	*90*	42 *(46.70)*	>7	*12.03 (4.02, 36.01)*	NR
	Blondel 2011 (5), France	**Travel distance (km):**	**Groups**	**N (1,359,756)**	***BBA n* (Rate /1000 births)**	**Groups**	**Crude OR (95%CI)**	**Adjusted OR (95%CI)**

For peer review only

		<5<br>5-14<br>15-29<br>30-44<br>>45	<5	596,363	*1849* (3.1)	<5	*1*	NR
			5-14	352,279	*1395* (3.9)	5-14	*1.28 (1.19, 1.37)*	NR
			15-29	296,734	*1659* (5.6)	15-29	*1.81 (1.69, 1.93)*	NR
			30-44	88,670	*692* (7.8)	30-44	*2.53 (2.32, 2.76)*	NR
			>45	15,705	*182* (11.)	45+	*3.77 (3.23, 4.39)*	NR
							**Parity 1&2<br>N =152,426**	**Parity 3+<br>N = 197,325**
						**Groups**	**Adjusted OR (95%CI)**	**Adjusted OR (95%CI)**
						<5	1	1.73 (1.57, 1.90)[a]
						5-14	1.14 (1.03, 1.27)	2.32 (2.04, 2.63)
						15-29	1.39 (1.24, 1.57)	3.25 (2.84, 3.71)
						30-44	1.78 (1.55, 2.05)	3.71 (3.13, 4.41)
						>45	2.47 (2.02, 3.02)	6.46 (4.92, 8.48)
	Ovaskainen 2015 (27), Finland	**Travel distance (km):**<br>< 35<br>≥35	**Groups**	**N (201)**	**BBA n (%)**	**Groups**	**Crude OR (95%CI)**	**Adjusted OR (95%CI)**
			BBA cases	67	NR	<35	NR	1
			Controls	134	NR	≥35	NR	5.02 (1.80, 14.04)
			<35 km	NR	NR			

For peer review only - http://bmjopen.bmj.com/site/about/guidelines.xhtml

			≥35 km	NR	NR			
	Fougner 2000 (26), Norway	**Travel distance (km):** <12.88 ≥12.88	**Groups**	**N (202) Cases & controls**	**BBA n (%)**	**Groups**	**Crude OR (95%CI)**	**Adjusted OR (95%CI)**
			<12.88	*90*	*44 (48.90)*	<12.88	1	NR
			≥12.88	*112*	*71 (63.34)*	≥12.88	*1.81 (1.03, 3.18)*	NR
**Neonatal unit admission (NNU)**	Lisonkova 2011 (28), Canada	**Travel distance (km):** <50 50-150 >150	**Groups**	**N (15,325)**	**NNU n (%)**	**Groups**	**Crude OR (95%CI)**	**Adjusted OR (95%CI)**
			<50	14,333	648 (4.80)	<50	1	NR
			50-150	815	32 (3.92)	50-150	*0.86 (0.60, 1.24)*	NR
			>150	177	12 (6.80)	>150	*1.54 (0.85, 2.77)*	NR
**Apgar score**	No studies							
**HIE**	No studies							

Abbreviations: CS: caesarean section; GA: Gestational age; HIE: Hypoxic-ischemic encephalopathy; ICU: intensive care unit; NNU: neonatal unit; NS: not significant, NR: not reported; RR: relative risk, OR: Odd ratio.

*Significant difference

[a] Reference group: women with 1&2 parity and < 5 km

For peer review only - http://bmjopen.bmj.com/site/about/guidelines.xhtml

Table 3A: Description of included studies – Travel time

Author, year, country	Study design & setting	Study objectives	Study period	Eligibility criteria	Participant characteristics	Description of exposures Travel Distance	Services context information	Review Outcomes	
								Perinatal	Maternal
**UK STUDIES**									
Dummer & Parker 2004 (29), UK	Retrospective population-based cohort study, Cumbria	To investigate whether geographical accessibility to hospitals affected SB rates and infant mortality	1950-1993 grouped: 1950-59 1960-69 1970-79 1980-93	*Included:* All births *Excluded:* Women with missing postcodes	N=283,668 births<br><br>Other characteristics: NR	Travel time (mins): < 17 18-35 >35	Universal state provision of maternity care. 1950-93: 4 hospitals opened, 2 closed	Neonatal mortality (NM); Early NM; Post NM	NR
Paranjothy 2013, 2014 (30, 31), UK (abstract& full paper)	Retrospective cohort study, All Wales Perinatal Survey & National Community Child Health Database	To study the association between travel time from home to OU on intrapartum stillbirth (SB) & NM	1995-2009	*Included:* All registered birth >23 wks GA *Excluded:* Antepartum SB, lethal congenital anomalies, multiple pregnancies, invalid or missing GA, missing	N= 466,255 singleton births<br>*Maternal age yrs %*<br>*<20 9.7*<br>*20-34 76.5*<br>*34-44 13.8*<br>*45+ 0.1*<br>*Parity:*<br>*Nullips 44.9%*<br>*Social deprivation quintile %:*<br>*1 (least depr) 16.7*<br>*2-4 57.8*	Travel time (mins): <15 15-29 30-44 >45	Universal state provision of maternity care. 50 hospitals (16 outside Wales)	Intrapartum SB; Early NM; Late NM	NR

For peer review only - http://bmjopen.bmj.com/site/about/guidelines.xhtml

				maternal age/postcode/ hospital of birth or baby's gender	*5 (most depr) 25.6* Ethnicity, education: NR				
**OTHER EUROPEAN STUDIES**									
Combier 2013 (32), France	Retrospective cohort study, based on hospital discharge summaries, Burgundy	To analyse the effect of travel time to closest OU on pregnancy outcome & prenatal management in Burgundy	2002 - 2009	*Included:* Singleton births >21 wks GA *Excluded:* Medical ToP, multiple pregnancy, births outside Burgundy, births in 2002 & 2008 due to closure of 3 units	N=111,001 births<br><br>Other characteristics =NR	Travel time (mins):<br>≤15<br>16-30<br>31-45<br>≥46	2000-2001: 2 private maternity units closed 2002-2009: 3 public maternity units closed.<br><br>Units(n):<br>2000 (20)<br>2009 (15)	SB; Perinatal mortality (PM); BBA	NR
Renesme 2013 (34), France	Case-control, multicentre study, 8 units, Finistere District, Brittany	To evaluate the social-geographical factors associated with BBAs	2007 - 2009	*Included cases:* BBA of live birth *Included controls*: 2 controls for each case irrespective of delivery mode. *Excluded:* GA <22 weeks,	N=225<br>Cases vs controls<br>N= 76 vs 149<br>Age (median, range) yrs: 30(16–41) vs 30(16-41)<br>Parity (median, range): 2(1–6)<br>Maternal INSEE code n(%): 1, 2, 3 or 4 = 15 (23.8) vs	Travel time (mins):<br>15<br>15–29<br>30–44<br>>45	9700 births/year in Finistere In 2012 units with <300 births/yr were closed. Universal state provision of maternity care	BBA	NR

For peer review only - http://bmjopen.bmj.com/site/about/guidelines.xhtml

				BW <500g, planned home birth	56(43.4); 5 or 6: 20 (31.8) vs 55 (42,6); 8 = 28 (44.4%) vs 18(14)<br>Ethnicity, education*:* NR				
Nguyen 2016 (33), France	Case control study, university hospital in Caen	To estimate the incidence of BBA during the study period	2002-2009	*Included cases:* Unplanned BBA<br>*Included controls:* Next spontaneous birth in hospital<br>*Excluded*: NR	N=188<br>*Cases* n=94<br>Mean age: 28.9 yrs<br>Parity: 1.8<br>SES: 73.4% no profession/student<br>Control n=94<br>Mean age: 29.2 yrs<br>Parity: 0.9<br>SES: 47.9% no profession/student<br>Ethnicity, education: NR	Travel time (mins):<br>≤ 20 mins<br>>20 mins	University Hospital with neonatal care facilities. Universal state provision of maternity care	BBA	NR
RAVELLI STUDY									

For peer review only - http://bmjopen.bmj.com/site/about/guidelines.xhtml

Ravelli 2011 (35-37), Netherlands (full papers & abstract**)**	Retrospective population-based cohort study, rural and urban areas, 12 provinces	To study the effect of travel time from home to OU on mortality and other adverse outcomes in pregnant women at term in primary and secondary care	2000 – 2006	*Included:* Singleton term births *Excluded:* Antepartum deaths, congenital anomalies, invalid/missing postcodes or outpatient codes, or births from Wadden islands, home deliveries, hospitals participated for 1-2 yrs	N=751,926 singleton births Age yrs ,% <20, 2 20-34, 78.3 35-39, 17.2 ≥40, 2.4 Parity*:* Nullips: 49.9% Ethnicity*:* White 81.7% SES % *: high 25.2, medium 48.2* , low 26.7 Education: NR	Travel time (mins): < 20 >=20	Universal state provision of maternity care. 99 OUs including tertiary perinatal centres	NM (Combined intrapartum & early & late NM up to 28 days) NM (0-24 hrs) NM (0-27 days) NM(8-27 days); Combined (mortality and/or Apgar <4 at 5min, and/or NNU admission)	NR
Ravelli 2012 (38), Netherlands	Retrospective cohort study in 9 regions	To investigate provincial differences in perinatal mortality and to determine the influence of different risk factors, including travel time from home to	2000-2006	*Included:* Singleton births *Excluded:* Women with incorrect post codes	N=1,242,725 singletons Age yrs, % <20, 1.8 >35, 19.5 Parity, % Nullips, 46.3 Ethnicity*, %* Non-western 16.2 SES low (10th centile): 10% Education NR	Travel time(mins): <20 >=20	Universal state provision of maternity care	PM	NR

For peer review only - http://bmjopen.bmj.com/site/about/guidelines.xhtml

		the OU during labour							
Stolp 2015 (39), Netherlands	Prospective cohort study, rural & urban areas	To assess whether the limit of 45 mins is met for ambulance transfer of women with PPH after home birth	2008-2010	*Included:* Women with PPH after MW supervised home birth *Excluded:* Cases of PPH with missing data	N= 72 (54 analysed) Age median (range) yrs: 31 (23-41); Parity (n,%): Primip 27-50% Ethnicity, Education, SES: NR	Travel time (mins): <45 >45	Home birth for low risk women and hospital birth. Universal state provision of maternity care	NR	Maternal admission to intensive care; (ICU); Blood transfusion; Postpartum haemorrhage (PPH)
ENGJOM STUDY									
Engjom 2017 & 2015 (40, 41), Norway (full paper & abstract)	Retrospective population-based cohort study, Medical Birth Registry of Norway and Statistics Norway, 19 counties	To assess peripartum mortality associated with place of birth and availability of obstetric units.	1999-2009	*Included:* All births in Norway with GA >=22 wks or BW>=500g *Excluded:* Lack of address and municipality, antepartum stillbirths, planned home births	N= 646,898 96.4% singletons. Age yrs, % <20 2.4 20-35, 80.7 >35, 16.9%; Multips 58.7%; Education >11y 77.2 Ethnicity: Western 90.7%	Travel time (hrs): <1 1-2 >2	Basic obstetric care for normal delivery; Emergency obstetric care < 1500 births/yr. Universal state provision of maternity care	BBA	NR
**CANADIAN STUDIES**									
Grzybowski & Stroll Study									

For peer review only - http://bmjopen.bmj.com/site/about/guidelines.xhtml

Grzybowski 2011(43), Canada	Retrospective cohort study, rural areas of British Columbia	To document newborn and maternal outcomes in relation to travel time to the nearest OU with CS capability	2000-2004	*Included:* All deliveries >20 weeks' GA *Excluded:* Multiple birth, congenital anomalies or late ToP, core urban areas	*N=35,426 births* *Groups:* *< 1 hr, 1-2, 2-4, >4 hrs* Group N: 32,814, 1359, 747, 506 Mean maternal age yrs: 28.7,28.67, 27.25, 27.2 Parity % primips: 42.6, 38.6, 36.7, 36.8 SES[1]: 0.12, 0.10, 0.30, 0.33, 1st Nations % 0.05, 0.30, 0.23, 0.42 Education: NR	Travel time (hrs): <1 1-2 2-4 >4	Universal medical coverage for core health care, 13 NNUs, 42,000 births /year	Perinatal mortality (SB & early NND); BBA; NNU admission	CS
Grzybowski, 2013 (44), Canada	Retrospective cohort study, rural areas of British Columbia	To compare rural maternity care by level of services	2000 - 2007	*Included:* Singleton births *Excluded:* Women with residential postcode of large urban centres	N=4672 births; Mean age, yrs: 27.7 Parity: primips: 39.7 % SES[1]: 0.22%; Ethnicity: 1st Nations 0.3% Education: NR	Travel time (hrs): <1 >1	Universal medical coverage for core health care	SB; NND (late < 1 month); PM; IM; BBA; NNU admission	CS; Emergency CS; PPH
Grzybowski 2015 (45), Canada	Retrospective cohort study, British Columbia (BC), Alberta, Nova Scotia (NS)	To examine the safety of rural Canadian maternity services	2003-2008	*Included:* Singleton deliveries *Excluded:* Multiple births, infants born with congenital anomalies, planned	*Alberta, BC, NS* Age yrs (n%) <18: 1618 (2.3), 1256 (2.0), 413 (2.2) >35 yrs: 5127 (7.3), 8866 (14.3), 2387 (12.7) Multips n(%) 41730 (59.6), 35089 (56.6), 10656 (56.8)	Travel time (hrs): <1 1-2 2-4 >4	Universal medical coverage for core health care , 20 small maternity closures since 2000	Perinatal mortality (SB & NND up to 7 days)	CS

For peer review only

For peer review only - http://bmjopen.bmj.com/site/about/guidelines.xhtml

				home births, accidental BBA	Ethnicity, SES, education: NR				
Stoll 2014 (46), Canada	Retrospective cohort study, rural British Columbia	To report on characteristics and perinatal outcomes of rural women with only MW involved in care	2003-2008	*Included:* Women residing outside core urban areas, singletons >20wk GA & care by a MW *Excluded:* Late ToP, congenital anomalies	*< 1 hr, 1-2 hrs, > 2hrs:* N= 3438, 124, 130 Mean age yrs: 29.78, 31.4, 30.5 Primips n(%) 1574 (45), 63 (50.8), 63 (48.5) Ethnicity, SES & Education*:* NR	Travel time (hrs): <1 1-2 >2	Universal medical coverage for core health care, closure of 22 rural maternity services	Perinatal mortality (SB & NND up to 7 days)	CS
Darling 2019 (42), Canada	Retrospective population-based cohort study, Ontario	Whether greater diving distances to OU associated with a higher risk of adverse neonatal outcomes	2012-2015	*Included:* Women who planned home births regardless of actual place of births *Excluded:* Multiple births, Preterm < 37 wks	*N= 11,869* *Age yrs,%:* <25, 9.5 25-39,87.6 ≥40, 2.9 Primps n (%) 4208 (35.5) SES low, n(%) 2465(20.8) Ethnicity & education: NR	Travel time (mins): ≤30 >30	Universal medical coverage for core health care	Perinatal mortality (PM); NNU admission; 5-mins Apgar <7	CS
**OTHER COUNTRIES**									

For peer review only - http://bmjopen.bmj.com/site/about/guidelines.xhtml

Aoshima 2011 (47), Japan	Before and after study design, data from perinatal care centres	Whether reducing travel time influences the neonatal mortality rate (NM)	2002-2006	*Included:* All births *Excluded:* Municipalities consisting of isolated islands	Number of births: 2002= 347,284 2006 = 322,514 Other characteristics: NR	Travel time (hrs): <= 1 >1	Universal healthcare insurance system, 346 perinatal care centres	NM	NR

Abbreviations: BBA: Born Before Arrival; Bthwt: Birth Weight; depr: deprive; GA: Gestational Age; IM: Infant Mortality; LBW: low birth weight; MW: midwife; NNU: Neonatal Unit; NM: Neonatal Mortality;NND: neonatal death; OBs: Obstetrician; PM: Perinatal mortality; PPH: postpartum haemorrhage; SB: stillbirth; SES: Socioeconomic status; ToP: Termination of pregnancy .

INSEE: Institute National de la Statistique et des Etudes Economiques; INSEE codes: 1: farmer; 2: craftsperson, merchant or entrepreneur; 3: business executive, intellectual occupation; 4: other professionals; 5: employee; 6: worker; 8: no occupation;

[1]SES: Catchment level Social vulnerability -1 to +1

[2]GPESS = general practitioner with enhanced surgical skills.

For peer review only - http://bmjopen.bmj.com/site/about/guidelines.xhtml

Table 3B: Risk of bias – Travel time

Author, year, country	Study sample selection bias<br>Additional criteria for case control studies	Bias in measurement of exposure	Bias in measurement of outcomes	Attrition bias	Analysis method reported and appropriate	Potential confounders adjusted for and listed
**UK STUDIES**						
Dummer & Parker 2004 (29), UK	**LOW**<br>Cumbrian Births Database	**LOW**<br>Modelled using GIS	**LOW**<br>Objective outcome (NM)	**LOW**<br>Of 3,352 live births, 42 stillbirths excluded as the outcome NM	**LOW**<br>Method detailed, results of LR were reported	**LOW**<br>Adjusted for year of birth, social class, birth order, multiple births
Paranjothy 2013 & 2014 (30, 31), UK	**LOW**<br>National Community Child Health Database & All Wales Perinatal Survey (AWPS)	**HIGH**<br>Women's address replaced by population-weighted centroid, travel time calculated using Google Maps API (v3)	**LOW**<br>Objective outcomes (SB, NM)	**LOW**<br>11% excluded where information on parity was missing	**LOW**<br>Analysis method described and multilevel LR data were reported	**LOW**<br>Adjusted for maternal age, parity, urban/rural location, SES, and other characteristics
**EUROPEAN STUDIES**						
Combier 2013 (32), France	**LOW**<br>Burgundy perinatal network database	**HIGH**<br>Municipality town hall not woman's home address	**LOW**<br>Objective outcomes (SB, PM, BBA)	**LOW**<br>All births identified included in the analysis	**LOW**<br>Method described; hierarchical LR and multilevel LR reported	**LOW**<br>Adjusted for maternal age, urbanization level and other characteristics

For peer review only - http://bmjopen.bmj.com/site/about/guidelines.xhtml

Renesme 2013 (34), France	Case definition	**YES** linked to perinatal network database	**LOW** Distance & travel time estimated using GIS	**LOW** Objective outcomes retrieved from regional and hospital databases	**LOW** 5/81 (6%) BBAs missing, 3/162 (2%) controls missing	**LOW** Method described and univariate and appropriate; multivariate reported	**LOW** Adjusted for age, family status, INSEE maternal occupation, parity, and other characteristics
	Representativeness of cases	**YES** All cases in defined period					
	Appropriate selection of controls	**YES** Controls chosen randomly from same databases and from births occurring at the nearest delivery date and hour to cases					
	Definition of control appropriate	**YES** Outcome could not have occurred					
	Comparability of cases and controls	**NO** Difference in antenatal care attendance					
Nguyen 2016 (33), France	Case definition	**YES** Using medical records	**UNCLEAR** No information	**LOW** Objective outcome (BBA)	**UNCLEAR** No information	**HIGH** No details of the analysis method and analysis was only descriptive	**HIGH** No adjustment for any potential confounders
	Representativeness of cases	**YES** All cases in defined period					

For peer review only

For peer review only - http://bmjopen.bmj.com/site/about/guidelines.xhtml

	Appropriate selection of controls	**YES** Next birth, of equivalent GA					
	Definition of control appropriate	**YES** Outcome could not have occurred					
	Comparability of cases and controls	**NO** Significant differences in parity, smoking, pregnancy monitoring, profession					
Ravelli 2011 (35-37), Netherlands (abstract & full papers)	**LOW** Population based study using the Netherlands Perinatal Registry		**LOW** GIS software used to measure travel time from women's postcodes	**LOW** Objective outcomes from perinatal registry	**LOW** Small proportion (0.3%) of women excluded due to incorrect zip code	**LOW** Method reported; descriptive analysis & LR results given	**LOW** Analysis adjusted for age, parity, ethnicity, SES
Ravelli 2012 (38), Netherlands	**LOW** Population based study using the Netherlands Perinatal Registry		**LOW** GIS software used to measure travel time from women's postcodes	**LOW** Objective outcomes from perinatal registry	**LOW** Small proportion 4% of women excluded	**UNCLEAR** No information	**LOW** Adjusted for age, parity, very urban /very rural, SES
Stolp 2015 (39), Netherlands	**HIGH** Study participants were selected by midwives		**LOW** Ambulance interval includes total time from dispatch call to arrival at hospital	**UNCLEAR** Method of measuring blood loss not reported	**HIGH** Missing data 18/72 (25%) due to incomplete documentation	**HIGH** Data only analysed descriptively	**HIGH** No adjusted analysis

For peer review only - http://bmjopen.bmj.com/site/about/guidelines.xhtml

Engjom 2017 & Engjom 2015 (40, 41), Norway (abstract & full paper)	**LOW** Medical Birth Registry of Norway	**LOW** Travel time polygon from home address using GIS	**LOW** Objective outcomes from birth registry	**UNCLEAR** No information	**LOW** Analysis appropriate, details of LR, multilevel modelling were reported	**LOW** Adjusted for maternal age, parity, education, ethnicity
Grzybowski 2011 (43), Canada	**LOW** Population based study using British Columbia Perinatal Health Program	**HIGH** GIS used to create one hour travel zone for each maternity service, but central postal code to the nearest maternity care used	**LOW** Objective outcomes from Perinatal Health Program	**LOW** 0.3% excluded due to incorrect zip code	**LOW** Analysis appropriate, descriptive analysis & hierarchical LR reported	**LOW** Adjusted for maternal age, parity, SES, ethnicity
Grzybowski 2013 (44), Canada	**LOW** Data from Perinatal Data Registry	**HIGH** Community central postal code used not women's home address	**LOW** Objective outcomes from Perinatal Data Registry	**HIGH** Number of women excluded due to incorrect postal address not reported	**LOW** Analysis appropriate descriptive analysis & LR	**LOW** Adjusted for maternal age, parity, lone parent status, ethnicity, SES
Grzybowski 2015 (45), Canada	**LOW** Provincial perinatal registries	**HIGH** Community central point postal code used not women's home address	**LOW** Objective outcomes from Perinatal Data Registries	**UNCLEAR** No information on missing data	**LOW** Analysis appropriate, descriptive analysis & LR reported	**LOW** Adjusted for maternal age, parity

For peer review only - http://bmjopen.bmj.com/site/about/guidelines.xhtml

Stoll 2014 (46), Canada	**LOW**<br>Based on British Columbia Perinatal Database Registry	**LOW**<br>Used GIS and Google maps; travel times were adjusted for travel conditions	**LOW**<br>Objective outcomes (CS)	**LOW**<br>No missing data	**HIGH**<br>Data were only analysed descriptively	**HIGH**<br>No adjusted analysis
Darling 2019 (42), Canada	**LOW**<br>Data from Perinatal Registries	**LOW**<br>Driving time from women's residence using online mapping tool ArcGIS	**LOW**<br>Objective outcomes from Perinatal Data Registries	**LOW**<br>3.7% excluded not being able to calculate distance to nearest hospital	**LOW**<br>Method reported, results of descriptive analysis & LR reported	**LOW**<br>Adjusted for maternal age, parity, gestational age, season, SES
Aoshima 2011 (47), Japan	**LOW**<br>All Japan except for isolated islands outside road network (96.6% of all Medical Service Areas)	**HIGH**<br>Used central point of municipality not home address but analysis based on (larger) Medical Service Areas.	**LOW**<br>Objective outcomes from Medical Service Area databases	**UNCLEAR**<br>No information on missing data	**LOW**<br>Method appropriate, unpaired t-test, difference-in-difference analysis	**HIGH**<br>No adjusted analysis

Abbreviations: BBA: Born Before Arrival; CS: caesarean section; GA gestational age; GIS: Geographical Information System; INSEE: Institute National de la Statistique et des Etudes Economiques; LR: Logistic regression; NM: Neonatal Mortality; NNU: Neonatal Unit; PM: Perinatal Mortality; SES: socioeconomic status; SB: Still Birth.

For peer review only - http://bmjopen.bmj.com/site/about/guidelines.xhtml

Table C3: Outcomes – Travel time

Outcomes	Study, Year, Country	Exposure groups	Participants (N, n, %)			Findings		
**MATERNAL OUTCOMES**								
**Maternal mortality (MM)**	No studies							
**Caesarean section (CS) (overall or intrapartum)**	Grzybowski 2011 (43), Canada	**Travel time (hrs):** **<1** 1-2 2-4 >4				**All CS**		
			**Groups**	**N (35,429)**	**All CS n (%)**	**Groups**	**Crude OR (95%CI)**	**Adjusted OR (95%CI)**
			<1	32814	*8597* (26.2)	<1	1	NR
			1-2	1359	*313* (23)	1-2	*0.84 (0.74, 0.96)*	NR
			2-4	747	*156* (20.9)	2-4	*0.74 (0.62, 0.89)*	NR
			>4	509	*97* (19.06)	>4	*0.66 (0.53, 0.83)*	NR
	Grzybowski 2013 (44), Canada	**Travel time (hrs):** <1 >1				**All CS**		
			**Groups**	**N (59,386)**	***n* (%)**	**Groups**	**Crude OR (95%CI)**	**Adjusted OR (95%CI)**
			<1	54 714	*14882* (27.20)	<1	1	NR
			>1	4672	*1075* (23.01)	>1	*0.80 (0.75, 0.86)*	NR

For peer review only - http://bmjopen.bmj.com/site/about/guidelines.xhtml

	Grzybowski 2015 (45), Canada	**Travel time (hrs):**<br><1<br>1-2<br>2-4<br>>4				**Alberta CS**		
			**Groups**	**Alberta N (34,453)**	**n%**	**Groups**	**Crude OR (95%CI)**	**Adjusted OR (95%CI)**
			<1 hr	29906	NR	<1	NR	1
			1-2	2940	NR	1-2	NR	0.86 (0.78, 0.94)
			2-4	1297	NR	2-4	NR	0.67 (0.58, 0.77)
			>4	310	NR	>4	NR	0.64 (0.48, 0.87)
						**British Columbia CS**		
			**Groups**	**British Columbia N (42,217)**	**n%**	**Groups**	**Crude OR (95%CI)**	**Adjusted OR (95%CI)**
			<1 hr	39,101	NR	<1	NR	1
			1-2	1892	NR	1-2	NR	0.92 (0.83, 1.03)
			2-4	623	NR	2-4	NR	0.74 (0.61, 0.90)
			>4	601	NR	>4	NR	0.70 (0.57, 0.85)
						**Nova Scotia CS**		
			**Groups**	**Nova Scotia N (17,336)**	**n%**	**Groups**	**Crude OR (95%CI)**	**Adjusted OR (95%CI)**
			<1 hr	15465	NR	<1	NR	1
			1-2	1772	NR	1-2	NR	0.87 (0.77, 0.98)

For peer review only - http://bmjopen.bmj.com/site/about/guidelines.xhtml

			2-4	99	NR	2-4	NR	0.67 (0.40, 1.10)
						>4	NR	-
	Stoll 2014 (46), Canada	**Travel time (hrs):**<br><1<br>1-2<br>>2	**Groups**	**N (3,692)**	**n (%)**	**Groups**	**Crude OR (95%CI)**	**Adjusted OR (95%CI)**
			<1	3438	633 (18.41)	<1	1	NR
			1-2	124	27 (21.80)	1-2	*1.23 (0.80, 1.91)*	NR
			>2	130	26 (20.0)	>2	*1.11 (0.71, 1.72)*	NR
	Darling 2019 (42), Canada	**Travel time (mins):**<br>≤ 30<br>>30	**Groups**	**N**	**n (%)**	**Groups**	***Crude OR (95%CI)***	**Adjusted OR (95%CI)**
			≤ 30	9189	536 (5.83)	≤ 30	*1*	NR
			>30	2236	98 (4.44)	>30	*0.74 (0.59, 0.92)*	NR
**Emergency CS**	Grzybowski 2013 (44), Canada	**Travel time (hrs):**<br><1<br>>1	**Groups**	**N (59,386)**	**n (%)**	**Groups**	***Crude OR (95%CI)***	**Adjusted OR (95%CI)**
			<1	54,714	9247 (16.99)	<1	*1*	NR
			>1	4672	701 (15.00)	>1	*0.80 (0.75, 0.86)*	NR
**Severe perineal trauma (3rd or 4th degree tear)**	No studies							
**Postpartum haemorrhage**	Stolp 2015 (39),	**Travel time (mins):**	**Groups**	**N (54)**	**n (%)**	**Groups**	**Median (range) ml**	

For peer review only - http://bmjopen.bmj.com/site/about/guidelines.xhtml

	Netherlands	<45<br>>45	<45	34	NR	<45	2,000 (1,100–7,000)	
			>45	20	NR	>45	2,050 (1,000–6,000) (P=0.9)	
	Grzybowski 2013 (44), Canada	**Travel time (hrs):**<br><1<br>>1	**Groups**	**N (59,386)**	**n (%)**	**Groups**	**Crude OR (95%CI)**	
			<1	54 714	*3064* (5.6)	<1	1	
			>1	4672	*327* (7.0)	>1	*1.27 (1.13, 1.43)*	
**Maternal admission to intensive care unit**	Stolp 2015 (39), Netherlands	**Travel time (mins):**<br><45<br>>45	**Groups**	**N (54)**	**n (%)**	**Groups**	**Crude OR (95%CI)**	**Adjusted OR (95%CI)**
			≤45	34	1 *(2.94)*	≤45	1	NR
			>45	20	1 *(5.0)*	>45	*1.74 (0.10, 29.39)*	NR
**Maternal blood transfusion**	Stolp 2015 (39), Netherlands	**Travel time (mins):**<br><45<br>>45	**Groups**	**N (54)**	**n (%)**	**Groups**	**Median (range) L**	
			≤45	34	≤45	≤45	0 (0-8)	
			>45	20	>45	>45	2 (0-8)	
**NEONATAL OUTCOMES**								
**Stillbirth (SB) (overall or intrapartum)**	Paranjothy 2014 (31), UK	**Every 15 min increase in travel time** (continuous variable)	**Groups**	**N (412,827)**	**SB n (%)**	**Groups**	**Crude OR (95%CI)**	**Adjusted OR (95%CI)**
			All women	412,827	135 (0.03)	All women	1.29 (1.14, 1.47)	1.13 (0.98, 1.30)

For peer review only - http://bmjopen.bmj.com/site/about/guidelines.xhtml

			Term births in hospital	387,429	85 (*0.02*)	Term births only	1.35 (1.16, 1.57)	1.36 (1.17, 1.59)
			Nullips births in hospital	185,419	69 (*0.04*)	Nullips only	1.33 (1.13, 1.57)	1.21 (1.02, 1.44)
	Combier 2013 (32), France	**Travel time (mins):**<br>≤ 15<br>16-30<br>31-45<br>≥46	**Groups**	**N (111,001)**	**SB n (%)**	**Groups**	**Crude OR (95%CI)**	**Adjusted OR (95%CI)**
			≤ 15	70,427	333 (0.47)	≤ 15	1	1
			16-30	31,792	148 (0.47)	16-30	0.98 (0.81, 1.20)	1.16 (0.96, 1.40)
			31-45	8445	50 (0.59)	31-45	1.25 (0.93, 1.69)	1.31 (0.89, 1.93)
			≥46	337	3 (0.89)	≥46	1.89 (0.60, 5.92)	1.90 (0.70, 5.15)
	Grzybowski 2013 (44), Canada	**Travel time (hrs):**<br><1<br>>1	**Groups**	**N (59,386)**	**SB n (Rate/1000)**	**Groups**	**Crude OR (95%CI)**	**Adjusted OR (95%%CI)**
			<1	54,714	*274* (5.0)	<1	1	1
			>1	4672	*28* (6.0)	>1	*1.20 (0.81, 1.77)*	NR
**Neonatal mortality (NM)**	Dummer 2004 (29), UK	**Travel time (mins):**<br>≤ 17<br>17-35<br>>35	**Groups**	**N (28,7993)**	**Early NM (0-6 days) n (%)**	**Early NM (0-6 days) Groups**	**Crude OR (95%CI)**	**Adjusted OR (95%CI)**
			≤ 17	NR	1850 (NR)	≤ 17	NR	1
			17-35	NR	789 (NR)	17-35	NR	0.97 (0.89, 1.06)
			>35	NR	196 (NR)	>35	NR	0.95 (0.81,1.1)

For peer review only - http://bmjopen.bmj.com/site/about/guidelines.xhtml

			**Groups**	**N (28,7993)**	**NM (0-27days) n (%)**	**NM (0-27 days) Groups**	**Crude OR (95%CI)**	**Adjusted OR (95%CI)**
			≤ 17	NR	1854 (NR)	≤ 17	NR	1
			17-35	NR	946 (NR)	17-35	NR	0.96 (0.89, 10.4)
			>35	NR	239 (NR)	>35	NR	0.95 (0.83, 1.09)
			Groups	**N (28,7993)**	**Post NM (28-1yr) n (%)**	**Post NM (28 days – 1yr) Groups**	**Crude OR (95%CI)**	**Adjusted OR (95%CI)**
			≤ 17	NR	961 (NR)	≤ 17	NR	1
			17-35	NR	400 (NR)	17-35	NR	0.97 (0.86,10.9)
			>35	NR	98 (NR)	>35	NR	0.95 (0.77, 1.17)
	Paranjothy 2014 (31), UK	**Every 15 min increase in travel time** (continuous variable)	**Groups**	**N**	**Early NM n (%)**	**Early NM (0-6 days) Groups**	**Crude OR (95%CI)**	**Adjusted OR (95%CI)**
			All women	412,827	609 *(0.15)*	All women	1.37 (1.31, 1.45)	1.13 (1.07, 1.20)
			Term births only	387,429	177 *(0.05)*	Term births only	1.02 (0.86, 1.21)	0.97 (0.80, 1.17)
			Nullips only	185,419	303 *(0.16)*	Nullips only	1.42 (1.33, 1.51)	1.15 (1.06, 1.25)
			**Groups**	**N**	**Late NM n (%)**	**Late NM (7-27 days) Groups**	**Crude OR (95%CI)**	**Adjusted OR (95%CI)**
			All women	412,827	251 *(0.06)*	All women	1.33 (1.23, 1.44)	1.15 (1.05, 1.26)

For peer review only - http://bmjopen.bmj.com/site/about/guidelines.xhtml

For peer review only

			Term births only	387,429	77 *(0.02)*	Term births only	1.24 (1.03, 1.50)	1.34 (1.13, 1.59)
			Nullips only	185,419	116 *(0.06)*	Nullips only	1.31 (1.15, 1.49)	1.11 (0.97, 1.28)
	Ravelli 2011 (35-37), Netherlands	**Travel time (mins):** < 20 mins ≥20 mins	**NM (Combined intrapartum & early neonatal mortality)**					
			**Groups**	**N (1,054,342)**	**NM n (%)**	**Groups**	**Crude OR (95%CI)**	**Adjusted OR (95%CI)**
			< 20	558,181	789 *(0.14)*	< 20 mins	1	1
			≥20	193,745	336 *(0.17)*	≥20 mins	*1.23 (1.08, 1.39)*	1.23 (1.07, 1.41)
			**Low risk women**	**N (120,896)**	**NM n(/1000) 63 (0.05/1000)**	**Low risk women**	**Crude OR (95%CI)**	**Adjusted OR (95%CI)**
			< 20	**NR**	**NR**	< 20	NR	1
			≥20	**NR**	**NR**	≥20	NR	0.8 (0.4, 1.7)
			**Low risk women became high risk during labour**	**N (142,824)**	**NM n(/1000) 1.9/1000**	**Low risk women became high risk during labour**	**Crude OR (95%CI)**	**Adjusted OR (95%CI)**
			<20	NR	NR	<20	NR	1
			≥20	NR	NR	≥20	NR	1.23 (1.04, 1.47)
		**Travel time (mins):** <15 15-19				**NM (Combined intrapartum & early & late NM up to 28 days)**		
			**Groups**	**N (751,926)**	**NM n (1125) (%)**	**Groups**	**Crude OR (95%CI)**	**Adjusted OR (95%CI)**

For peer review only - http://bmjopen.bmj.com/site/about/guidelines.xhtml

		≥20	<15	425,952	NR	<15	1	1
			15-19	132,229	NR	15-19	0.97 (0.82, 1.15)	0.94 (0.79, 1.12)
			≥20	193,745	336	≥ 20	1.22 (1.07, 1.39)	1.17 (1.02, 1.36)
						**Neonatal mortality within 24 hrs**		
			**Groups**	**N (751,926)**	**NM within 24 hrs n (255) (%)**	**Groups**	**Crude OR (95%CI)**	**Adjusted OR (95%CI)**
			<20	558,181	NR	<20	1	1
			≥20	193,745	NR	≥ 20	1.52 (1.17, 1.97)	1.51 (1.13, 2.02)
						**Neonatal mortality 0-7 days**		
			**Groups**	**N (751,926)**	**NM 0-7 dys (523) (%)**	**Groups**	**Crude OR (95%CI)**	**Adjusted OR (95%CI)**
			<20	558,181	NR	<20	1	1
			≥20	193,745	NR	≥ 20	1.44 (1.20, 1.72)	1.37 (1.12, 1.67)
						**Neonatal mortality 8-27 days**		
			**Groups**	**N (751,926)**	**NM 8-27 dys (58) (%)**	**Groups**	**Crude OR (95%CI)**	**Adjusted OR(95%CI)**
			<20	558,181	NR	<20	1	1
			≥20	193,745	NR	≥ 20	1.30 (0.74, 2.26)	1.24 (0.67, 2.27)
	Grzybowski 2013 (44),	**Travel time**	**Groups**	**N (59,386)**	**NM n (%)**	Late neonatal mortality age <1 month, no events		

For peer review only - http://bmjopen.bmj.com/site/about/guidelines.xhtml

	Canada	**(hrs):**<br><1<br>>1	<1	54,714	0			
			>1	4672	0			
	Aoshima 2011 (47), Japan	**Travel time (mins):**<br>Median 39.09 (2006)<br>Median 66.99 (2002)	**Groups**	**N**	**NM n (Rate/ 1000)**	**Groups**	**Crude OR (95%CI)**	**Adjusted OR (95%CI)**
			2006	NR	NR (1.28)	39.09	NR	NR
			2002	NR	NR (1.67)	66.99	NR	NR
**Perinatal mortality (PM)**	Combier 2013 (32), France	**Travel time (mins):**<br>≤15<br>16 -30<br>31-45<br>≥46	**Groups**	**N (110,664)**	**PM n (%)**	**Groups**	Crude OR (95%CI)	**Adjusted OR (95%CI)**
			≤15	70,427	452 (0.64)	≤15	1	1
			16-30	31,792	195 (0.61)	16-30	0.96 (0.81, 1.13)	1.08 (0.90, 1.29)
			31-45	8445	59 (0.7.0)	31-45	1.09 (0.83, 1.43)	1.18 (0.86, 1.62)
			≥46	337	4 (1.19)	≥46	1.86 (0.69, 5.01)	1.85 (0.66, 5.19)
	Ravelli 2012 (38), Netherlands	**Travel time (mins):**<br><20<br>≥20	**Groups**	**N (1,242,725)**	**PM n (Rate/1000)**	**Groups**	**Crude OR (95%CI)**	**Adjusted OR (95%CI)**
			<20	*1,006,607*	81 (0.08)	<20	1	1
			≥20	*236,118*	19 (0.08)	≥ 20	1.53 (1.47,1.50)	1.66 (1.59,1.74)
	Grzybowski 2011(43), Canada	**Travel time (hrs):**<br><1<br>1-2<br>2-4<br>>4	**Groups**	**N (35,429)**	**PM n (Rate/1000)**	**Groups**	**Crude OR (95%CI)**	**Adjusted OR (95%CI)**
			<1	32,814	*197* (6.0)	<1	1	1
			1-2	1359	*8* (6.0)	1-2	0.98 (0.48, 1.99)	1.04 (0.48, 2.22)

For peer review only - http://bmjopen.bmj.com/site/about/guidelines.xhtml

			2-4	747	*4* (5.0)	2-4	0.89 (0.33, 2.40)	0.92 (0.33, 2.53)
			>4	509	*9* (18.0)	>4	2.98 (1.52, 5.85)	3.17 (1.45, 6.95)
			PM (stillbirths & early neonatal mortality)					
	Grzybowski 2013 (44), Canada	**Travel time (hrs):**<br><1<br>>1	**Groups**	**N (59,386)**	**PM n (Rate/1000)**	**Groups**	**Crude OR (95%CI)**	**Adjusted (OR 95%CI)**
			< 1	54 714	*383* (7.0)	<1	1	NR
			>1	4672	*37* (8.0)	>1	*1.54 (1.09, 2.17)*	NR
	Grzybowski 2015 (45), Canada	**Travel time (hrs):**<br><1<br>1-2<br>2-4<br>>4				**PM (stillbirths & early neonatal mortality) Alberta**		
			**Groups**	**Alberta (N=34,453)**	**PM n (%)**	**Groups**	**Crude OR (95%CI)**	**Adjusted OR (95%CI)**
			<1 hr	29,906	NR	<1	1	1
			1-2	2940	NR	1-2	NR	1.50 (1.03, 2.18)
			2-4	1297	NR	2-4	NR	1.35 (0.77, 2.38)
			>4	310	NR	>4	NR	1.40 (0.44, 4.39)
						**PM (stillbirths & early neonatal mortality) BC**		
			**Groups**	**British Columbia (N=42,317)**	**PM n (%)**	**Groups**	**Crude OR (95%CI)**	**Adjusted OR (95%CI)**
			<1 hr	39,101	NR	<1	1	1

For peer review only - http://bmjopen.bmj.com/site/about/guidelines.xhtml

			1-2	1892	NR	1-2	NR	0.79 (0.43, 1.45)
			2-4	623	NR	2-4	NR	1.33 (0.59, 3.01)
			>4	601	NR	>4	NR	2.84 (1.58, 5.10)
						**PM (stillbirths & early neonatal mortality) Nova Scotia**		
			**Groups**	**Nova Scotia (N= 17,336)**	**PM n (%)**	**Groups**	**Crude OR (95%CI)**	**Adjusted OR (95%CI)**
			<1 hr	15,465	NR	<1	1	1
			1-2	1772	NR	1-2	NR	0.66 (0.38, 1.14)
			2-4	99	NR	2-4	NR	NR
			>4	0	NR	>4	NR	NR
	Stoll 2014 (46), Canada	**Travel time (hrs):**<br><1<br>1-2<br>>2	**Groups**	**N (3,692)**	**PM n (%)**	**Groups**	**Crude OR (95%CI)**	**Adjusted OR (95%CI)**
			<1	3438	15 (0.4)	<1	1	NR
			1-2	124	0	1-2	*0.89 (0.05, 14.91)*	NR
			>2	130	2 (1.5)	>2	*3.57 (0.81, 15.76)*	NR
			PM (Stillbirth and early neonatal death up to 7 days)					
	Darling 2019 (42), Canada	**Travel time (mins):**<br>≤ 30<br>>30	**Groups**	**N (10,681)**	**PM n (%)**	**Groups**	***Crude OR (95%CI)***	**Adjusted OR (95%CI)**
			≤ 30	NR	NR	≤ 30	NR	NR

For peer review only

			>30	NR	NR	>30	NR	NR<br>Reported as RR<br>2.2 (0.67, 7.43)
**Infant mortality (IM)**	Grzybowski 2013 (44), Canada	*Travel time (hrs):*<br><1<br>>1				**IM (age 1-12 month)**		
			**Groups**	**N (59,386)**	**IM n(Rate/1000)**	**Groups**	**Crude OR (95%CI)**	**Adjusted OR (95%CI)**
			<1	54,714	*109* (2.0)	<1	1	NR
			>1	4672	*14* (3.0)	>1	*1.51 (0.86, 2.63)*	NR
**Born before arrival (BBA)**	Combier 2013 (32), France	Travel time (mins):<br><15<br>15-29<br>30-44<br>>45	**Groups**	**N (111,001)**	**BBA n (%)**	**Groups**	**Crude OR (95%CI)**	**Adjusted OR (95%CI)**
			<16	70,427	132 (0.19)	<16	1	1
			16-30	31,792	93 (0.29)	16-30	1.56 (1.20, 2.04)	1.73 (1.23, 2.46)
			31-45	8445	29 (0.34)	31-45	1.84 (1.23, 2.75)	1.64 (1.06, 2.54)
			>45	337	0	>45	-	-
	Renesme 2013 (34), France	**Travel time (mins):**<br><15<br>15-29<br>30-44<br>>45	**Groups**	**Cases N (73) (%)**	**Control N(148) (%)**	**Groups**	**Crude OR (95%CI)**	**Adjusted OR(95%CI)**
			<15	22 (30.2)	59 (39.9)	<15	1	1
			15-29	33 (45.2)	64 (43.2)	15-29	*1.79 (0.87, 3.68)*	1.92 (0.86, 4.96)
			30-44	9 (12.3)	18 (12.2)	30-44	*1.68 (0.58, 4.87)*	1.10 (0.35, 3.48)
			>45	9 (12.3)	7 (4.7)	>45	*5.89 (1.12, 30.89)*	6.18 (1.33, 8.65)

For peer review only - http://bmjopen.bmj.com/site/about/guidelines.xhtml

	Nguyen 2016 (33), France	**Travel time (mins):**<br>≤ 20<br>> 20	**Groups**	**N (188)**	**BBA n (%)**	**Groups**	**Crude OR (95%CI)**	**Adjusted OR(95%CI)**
			>20	94 controls	22 (23.4)	>20 controls	1	NR
			>20	94 cases	27 (28.7)	>20 cases	*1.3 (0.7, 2.6)*	NR
	Engjom 2017 (41), Norway	**Travel time (hrs):**<br><1<br>1-2<br>>2	**Groups**	**N (646,898)**	**BBA n (%)**	**Groups**	**Crude OR (95%CI)**	**Adjusted OR (95%CI)**
			1	615,896	3488 (0.60)	1	1	1
			1-2	25,494	844 (3.31)	1-2	*6.01 (5.57, 6.49)*	NR reported as RR* 5.3 (5.0,5.8)
			>2	5508	246 (4.50)	>2	*8.21 (7.19, 9.37)*	NR reported as RR* 7.2 (6.3,8.2)
	Grzybowski 2011 (43), Canada	**Travel time (hrs):**<br><1<br>1-2<br>2-4<br>>4	**Groups**	**N (35,429)**	**BBA n (%)**	**Groups**	**Crude OR (95%CI)**	**Adjusted OR (95%CI)**
			<1	32,814	*66* (0.20)	<1	1	1
			1-2	1359	*31* (2.30)	1-2	*11.58 (7.53, 17.81)*	6.41(3.69,11.28)
			2-4	747	*3* (0.3)	2-4	*2.00 (0.63, 6.38)*	0.92 (0.22, 3.88)
			>4	506	*7* (1.4)	>4	*6.96 (3.18, 15.25)*	3.63 (1.40, 9.40)
	Grzybowski 2013 (44), Canada	**Travel time (hrs):**<br><1<br>>1	**Groups**	**N (59,386)**	**BBA n (%)**	**Groups**	**Crude OR (95%CI)**	**Adjusted OR (95%CI)**
			<1	54,714	*164* (0.3)	<1	1	NR
			>1	4672	*70* (1.5)	>1	*5.06 (3.82, 6.70)*	NR
**Combined mortality &**	Ravelli 2011 (35-37),	**Travel time (mins):**	**Groups**	**N(751,926)**	**Event n (4,543) (%)**	**Groups**	**Crude OR (95%CI)**	**Adjusted OR (95%CI)**

For peer review only - http://bmjopen.bmj.com/site/about/guidelines.xhtml

**or Apgar < 4 at 5 mins & or transfer to NICU**	Netherlands	<15<br>15-19<br>>=20	<15	425,952	NR	<15	1	1
			15-20	132,229	NR	15-20	0.99 (091, 1.07)	1.11 (1.02, 1.21)
			>=20	193,745	NR	>=20	1.11 (1.04, 1.19)	1.27 (1.17, 1.38)
**Neonatal Unit admission (NNU)**	Grzybowski 2011 (43), Canada	Travel time (hrs):<br><1<br>1-2<br>2-4<br>>4	**NICU level 2 admissions per 1000 births (2001-2004)**			**NICU 2**		
			**Groups**	**N (35,429)**	**NICU2 n (Rate/1000)**	**Groups**	**Crude OR (95%CI)**	**Adjusted OR (95%CI)**
			<1	32,814	*1082* (33.0)	<1	1	1
			1-2	1359	*69* (51.0)	1-2	*1.57 (1.22, 2.01)*	2.20 (1.59, 3.05)
			2-4	747	*8* (11.0)	2-4	*0.32 (0.16, 0.64)*	0.31 (0.14, 0.65)
			>4	506	*14* (27.0)	>4	*0.83 (0.49, 1.42)*	1.07 (0.54, 2.12)
			**NICU level 3 per 1000 births (2001-2004)**			**NICU 3**		
			**Groups**	**N (34,920)**	**NICU3 n (Rate/1000)**	**Groups**	**Crude OR (95%CI)**	**Adjusted OR (95%CI)**
			<1	32,814	*98* (3.0)	<1	1	NR
			1-2	1359	*11* (8.0)	1-2	*2.72 (1.46, 5.09)*	NR
			2-4	747	*4* (5.0)	2-4	*1.80 (0.66, 4.90)*	NR
			>4	509	*2* (4.0)	>4	*1.32 (0.32, 5.35)*	NR
	Grzybowski 2013 (44),	**Travel time (hrs):**	**NICU level 2 (2001-2 & 2006-7) n=74697**			**NICU 2**		

For peer review only

	Canada	<1<br>>1	**Groups**	**N**	**NICU2 n (Rate/1000)**	**Groups**	**Crude OR (95%CI)**	**Adjusted OR (95%CI)**
			<1	54,714	*1751* (32.0)	<1	1	NR
			>1	4672	*154* (33.0)	>1	*1.03 (0.87, 1.22)*	NR
			**NICU level 3 (2001-2 & 2006-7) n=74697**			**NICU 3**		
			**Groups**	**N**	**NICU3 n(Rate/1000)**	**Groups**	**Crude OR (95%CI)**	**Adjusted OR (95%CI)**
			<1	54,714	*219* (4.0)	<1	1	NR
			>1	4672	*28* (6.0)	>1	*1.50 (1.01, 2.23)*	NR
			**NICU (2&3)**			**NICU admission**		
			**Groups**	**N (59,386)**	**NICU n (Rate/1000)**	**Groups**	**Crude OR (95%CI)**	**Adjusted OR (95%CI)**
			<1	54,714	*1970 (36.0)*	<1	1	NR
			>1	4672	*182 (39.0)*	>1	*1.09 (0.93, 1.27)*	NR
	Darling 2019 (42), Canada	**Travel time (mins):**<br>≤ 30<br>>30	**Groups**	**N (10,687)**	**NICU n (%)**	**Groups**	**Crude OR (95%CI)**	**Adjusted OR (95%CI)**
			≤ 30	NR	NR	≤ 30	NR	1
			>30	NR	NR	>30	NR	NR<br>Reported as RR*<br>0.6 (0.44, 0.81)
**Apgar <7 at 5 mins**	Darling 2019 (42), Canada	**Travel time (mins):**	**Groups**	**N (10,578)**	**Apgar <7 n (%)**	**Groups**	**Crude RR (95%CI)**	**Adjusted OR (95%CI)**

For peer review only - http://bmjopen.bmj.com/site/about/guidelines.xhtml

		≤ 30<br>>30	≤ 30	NR	NR	≤ 30	NR	1
			>30	NR	NR	>30	NR	NR<br>Reported as RR*<br>1.02 (0.95, 1.10)
			**Nullips**	**N (4208)**	**Apgar <7 n (%)**	**Groups**	**Crude OR (95%CI)**	**Adjusted OR (95%CI)**
			≤ 30	3425	51 (1.5)	≤ 30	1	NR
			>30	621	14 (2.3)	>30	*1.53 (0.84, 2.77)*	NR
			**Mullips**	**N (7661)**	**Apgar <7 n (%)**	**Groups**	**Crude OR (95%CI)**	**Adjusted OR (95%CI)**
			≤ 30	5764	30 (0.5)	≤ 30	*1*	NR
			>30	1615	11 (0.7)	>30	*1.31 (0.66, 2.62)*	NR
**HIE**	No studies reported							

Abbreviations: BW: Birth Weight; NR: not reported; Nullips: Nulliparous; NICU: Neonatal Intensive Care Unit; * RR: relative risk.

For peer review only - http://bmjopen.bmj.com/site/about/guidelines.xhtml

References:

1. Jordan H, Roderick P, Martin D, Barnett S. Distance, rurality and the need for care: access to health services in South West England. International Journal of Health Geographics. 2004;3(1):21.
2. Kelly C, Hulme C, Farragher T, Clarke G. Are differences in travel time or distance to healthcare for adults in global north countries associated with an impact on health outcomes? A systematic review. BMJ open. 2016;6(11):e013059-e.
3. Rashidian A, Omidvari AH, Vali Y, Mortaz S, Yousefi-Nooraie R, Jafari M, et al. The effectiveness of regionalization of perinatal care services - a systematic review. Public Health. 2014;128(10):872-85.
4. Price Sian. Research evidence review -impact of distance/travel time to maternity services on birth outcomes. Public Health Wales NHS Trust 2015.
5. Blondel BD, Nicolas; Pilkington, Hugo; Zeitlin, Jennifer. Out-of-hospital births and the supply of maternity units in France. Health & Place. 2011;17(5):1170-3.
6. Lasswell SM, Barfield WD, Rochat RW, Blackmon L. Perinatal regionalization for very low-birth-weight and very preterm infants: a meta-analysis. Jama. 2010;304(9):992-1000.
7. Engjom HMM, N. H.; Norheim, O. F.; Klungsoyr, K. Availability and access in modern obstetric care: a retrospective population-based study. BJOG: An International Journal of Obstetrics & Gynaecology. 2014;121(3):290-9.
8. Stroup DF, Berlin JA, Morton SC, Olkin I, Williamson GD, Rennie D, et al. Meta-analysis of Observational Studies in EpidemiologyA Proposal for Reporting. JAMA. 2000;283(15):2008-12.
9. Merenstein G, Glicken A. Best Evidence-Based Practices: A Historic Perspective. Neonatal Network. 2002(5):31-5.
10. World Health Organization. Universal health coverage (UHC). https://wwwwhoint/news-room/fact-sheets/detail/universal-health-coverage-(uhc). 2019.
11. Rowe R, The Birthplace in England Collaborative Group, ., McCourt, C. and MacFarlane, A, . Birthplace terms and definitions: consensus process Birthplace in England research programme. (Final Report 2. 08/1604/140). Southampton: HMSO. http://openaccesscityacuk/3651/1/Birthplace%20definitions%20rpt%20SDO_FR2_08-1604-140_V02pdf. 2011.
12. Thomas J, Brunton J, S. G. EPPI-Reviewer 40: software for research synthesis EPPI-Centre Software London: Social Science Research Unit, Institute of Education, University of London. 2010.
13. Cochrane Effective Practice and Organisation of Care (EPOC). Suggested risk of bias cri teria for EPOC reviews. EPOC Resources fo r review authors. Available at: http ://ep occochraneorg/res ources /epoc-res ources-revi ew-authors. 2017.
14. Stang A. Critical evaluation of the Newcastle-Ottawa scale for the assessment of the quality of nonrandomized studies in meta-analyses. Eur J Epidemiol. 2010;25(9):603-5.
15. Fleming AM, E. A.; Schram, C. M. H. Reducing caesarean section rates through choice and collaboration. Archives of Disease in Childhood: Fetal and Neonatal Edition Conference: 16th Annual Conference of the British Maternal and Fetal Medicine Society Dublin Ireland Conference Publication:. 2013;98(no pagination).
16. Mackie FLA, F.; Moise, J.; Amu, O. Maternal and neonatal outcomes after the amalgamation of two maternity units and consequent increased consultant labour ward presence: A retrospective population-based study. Archives of Disease in Childhood: Fetal and Neonatal Edition. 2014;99:A21-A2.
17. Grytten JM, Lars; Skau, Irene; Sorensen, Rune. Regionalization and local hospital closure in Norwegian maternity care--the effect on neonatal and infant mortality. Health Services Research. 2014;49(4):1184-204.
18. Hemminki EH, A.; Gissler, M. Should births be centralised in higher level hospitals? Experiences from regionalised health care in Finland. BJOG: An International Journal of Obstetrics & Gynaecology. 2011;118(10):1186-95.

For peer review only - http://bmjopen.bmj.com/site/about/guidelines.xhtml

19. Allen VM, Jilwah N, Joseph KS, Dodds L, O'Connell CM, Luther ER, et al. The Influence of Hospital Closures in Nova Scotia on Perinatal Outcomes. Journal of Obstetrics and Gynaecology Canada. 2004;26(12):1077-85.
20. Hutcheon J, A.; Riddell Corinne, A.; Strumpf Erin, C.; Lee, Lily; Harper, Sam. Safety of labour and delivery following closures of obstetric services in small community hospitals. CMAJ Canadian Medical Association Journal. 2017;189(11):E431-E6.
21. Le Coutour I-R, C.; Danzon, A. [Regionalization of health care and obstetric practice]. Revue d Epidemiologie et de Sante Publique. 1990;38(3):211-20.
22. Allen V, M.; Jilwah, Nadia; Joseph, K. S.; Dodds, Linda; O'Connell Colleen, M.; Luther Edwin, R.; Fahey, T. John; Attenborough, Rebecca; Allen Alexander, C. The influence of hospital closures in Nova Scotia on perinatal outcomes. Journal of Obstetrics & Gynaecology Canada: JOGC. 2004;26(12):1077-85.
23. Bhoopalam PS, Watkinson M. Babies born before arrival at hospital. BJOG: An International Journal of Obstetrics & Gynaecology. 1991;98(1):57-64.
24. Pasquier JCM, M.; Bagouet, S.; Moret, S.; Luo, Z. C.; Rabilloud, M.; Gaucherand, P.; Robert-Gnansia, E. Effects of residential distance to hospitals with neonatal surgery care on prenatal management and outcome of pregnancies with severe fetal malformations. Ultrasound in Obstetrics & Gynecology. 2007;29(3):271-5.
25. Pilkington HB, Beatrice; Drewniak, Nicolas; Zeitlin, Jennifer. Where does distance matter? Distance to the closest maternity unit and risk of foetal and neonatal mortality in France. European Journal of Public Health. 2014;24(6):905-10.
26. Fougner BN, J. [Deliveries during transportation and shortly after admission to hospital]. Tidsskrift for Den Norske Laegeforening. 2000;120(11):1288-90.
27. Ovaskainen KO, Riitta; Gissler, Mika; Luukkaala, Tiina; Tammela, Outi. Out-of-hospital deliveries have risen involving greater neonatal morbidity: Risk factors in out-of-hospital deliveries in one University Hospital region in Finland. Acta Paediatrica. 2015;104(12):1248-52.
28. Lisonkova S, Sheps SB, Janssen PA, Lee SK, Dahlgren L, MacNab YC. Birth Outcomes Among Older Mothers in Rural Versus Urban Areas: A Residence-Based Approach. The Journal of Rural Health. 2011;27(2):211-9.
29. Dummer TJBP, L. Hospital accessibility and infant death risk. Archives of Disease in Childhood. 2004;89(3):232-4.
30. Paranjothy SW, W. J.; Rolfe, K.; Gong, Y.; Adappa, R.; Dunstan, F.; Kotecha, S. Perinatal outcomes and travel time to maternity services: Analysis of birth outcome data in Wales from 1995 to 2009. Archives of Disease in Childhood: Fetal and Neonatal Edition Conference: 16th Annual Conference of the British Maternal and Fetal Medicine Society Dublin Ireland Conference Publication:. 2013;98(no pagination).
31. Paranjothy SW, W. John; Rolfe, Kim; Adappa, Roshan; Gong, Yi; Dunstan, Frank; Kotecha, Sailesh. Perinatal outcomes and travel time from home to hospital: Welsh data from 1995 to 2009. Acta Paediatrica. 2014;103(12):e522-7.
32. Combier EC, Helene; Le, Vaillant; Marc,; Michaut, Francis; Ferdynus, Cyril; Amat-Roze, Jeanne-Marie; Gouyon, Jean-Bernard; Quantin, Catherine; Zeitlin, Jennifer. Perinatal health inequalities and accessibility of maternity services in a rural French region: closing maternity units in Burgundy. Health & Place. 2013;24:225-33.
33. Nguyen MLL, P.; Dreyfus, M. [Maternal and neonatal outcomes of unplanned deliveries]. Journal de Gynecologie, Obstetrique et Biologie de la Reproduction. 2016;45(1):86-91.
34. Renesme L, Garlantézec R, Anouilh F, Bertschy F, Carpentier M, Sizun J. Accidental out-of-hospital deliveries: a case–control study. Acta Paediatrica. 2013;102(4):e174-e7.
35. Ravelli ACJJ, K. J.; De, Groot; M, H.; Rijninks-Van, Driel; G, C.; Tromp, M.; Eskes, M.; Willem, B.; Mol, J.; Abu-Hanna, A.; Jaap, J.; Erwich, H. M. Travel time from home to hospital and intrapartum and neonatal mortality in term pregnant women. Journal of Maternal-Fetal and Neonatal Medicine. 2010;23:234.

For peer review only - http://bmjopen.bmj.com/site/about/guidelines.xhtml

36. Ravelli ACJJ, K. J.; De, Groot; M, H.; Erwich, J. J. H. M.; Rijninks-Van, Driel; G, C.; Tromp, M.; Eskes, M.; Abu-Hanna, A.; Mol, B. W. J. Travel time from home to hospital and adverse perinatal outcomes in women at term in the Netherlands. Obstetrical and Gynecological Survey. 2011;66(7):396-8.
37. Ravelli ACJJ, K. J.; de, Groot; M, H.; Erwich, J. J. H. M.; Rijninks-van, Driel; G, C.; Tromp, M.; Eskes, M.; Abu-Hanna, A.; Mol, B. W. J. Travel time from home to hospital and adverse perinatal outcomes in women at term in the Netherlands. BJOG: An International Journal of Obstetrics & Gynaecology. 2011;118(4):457-65.
38. Ravelli AEM, Erwich JJHM, Brouwers HAA, Abu-Hanna A, Kerkhof FGM, van der Post JAM, H.O.PC., Neoned* & B.W.J. Mol. Perinatale sterfteverschillen in de negen perinatale zorgregio's Nederlands tijdschrift voor Obstetrie & Gynaecologie 2012 125, (augustus 2012):270-7.
39. Stolp IS, Marrit; Luxemburg, Sanne; Akker, Thomas; Waard, Jan; Roosmalen, Jos; Vos, Rien. Ambulance Transfer in Case of Postpartum Hemorrhage after Birth in Primary Midwifery Care in The Netherlands: A Prospective Cohort Study. Birth: Issues in Perinatal Care. 2015;42(3):227-34.
40. Engjom HM, N. H.; Hoydal, E.; Norheim, O. F.; Klungsoyr, K. Obstetric health system structure and perinatal outcomes in Norway. International Journal of Gynecology and Obstetrics. 2015;131:E487-E8.
41. Engjom HM, Morken N-H, Høydahl E, Norheim OF, Klungsøyr K. Increased risk of peripartum perinatal mortality in unplanned births outside an institution: a retrospective population-based study. American Journal of Obstetrics and Gynecology. 2017;217(2):210.e1-.e12.
42. Darling EK, Lawford KMO, Wilson K, Kryzanauskas M, Bourgeault IL. Distance from Home Birth to Emergency Obstetric Services and Neonatal Outcomes: A Cohort Study. Journal of Midwifery & Women's Health. 2019;64(2):170-8.
43. Grzybowski SS, Kathrin; Kornelsen, Jude. Distance matters: a population based study examining access to maternity services for rural women. BMC Health Services Research. 2011;11:147.
44. Grzybowski SS, Kathrin; Kornelsen, Jude. The outcomes of perinatal surgical services in rural British Columbia: a population-based study. Canadian Journal of Rural Medicine. 2013;18(4):123-9.
45. Grzybowski SF, John; Lai, Barbara; Zhang, Sharon; Aelicks, Nancy; Leung Brenda, M.; Stoll, Kathrin; Attenborough, Rebecca. The safety of Canadian rural maternity services: a multi-jurisdictional cohort analysis. BMC Health Services Research. 2015;15:410.
46. Stoll KK, Jude. Midwifery Care in Rural and Remote British Columbia: A Retrospective Cohort Study of Perinatal Outcomes of Rural Parturient Women With a Midwife Involved in Their Care, 2003 to 2008. Journal of Midwifery & Women's Health. 2014;59(1):60-6.
47. Aoshima KK, H.; Kawahara, K. Neonatal mortality rate reduction by improving geographic accessibility to perinatal care centers in Japan. Journal of Medical and Dental Sciences. 2011;58(2):29-40.

Figure 1. PRISMA Flow Diagram

For peer review only - http://bmjopen.bmj.com/site/about/guidelines.xhtml

PRISMA Flow Diagram

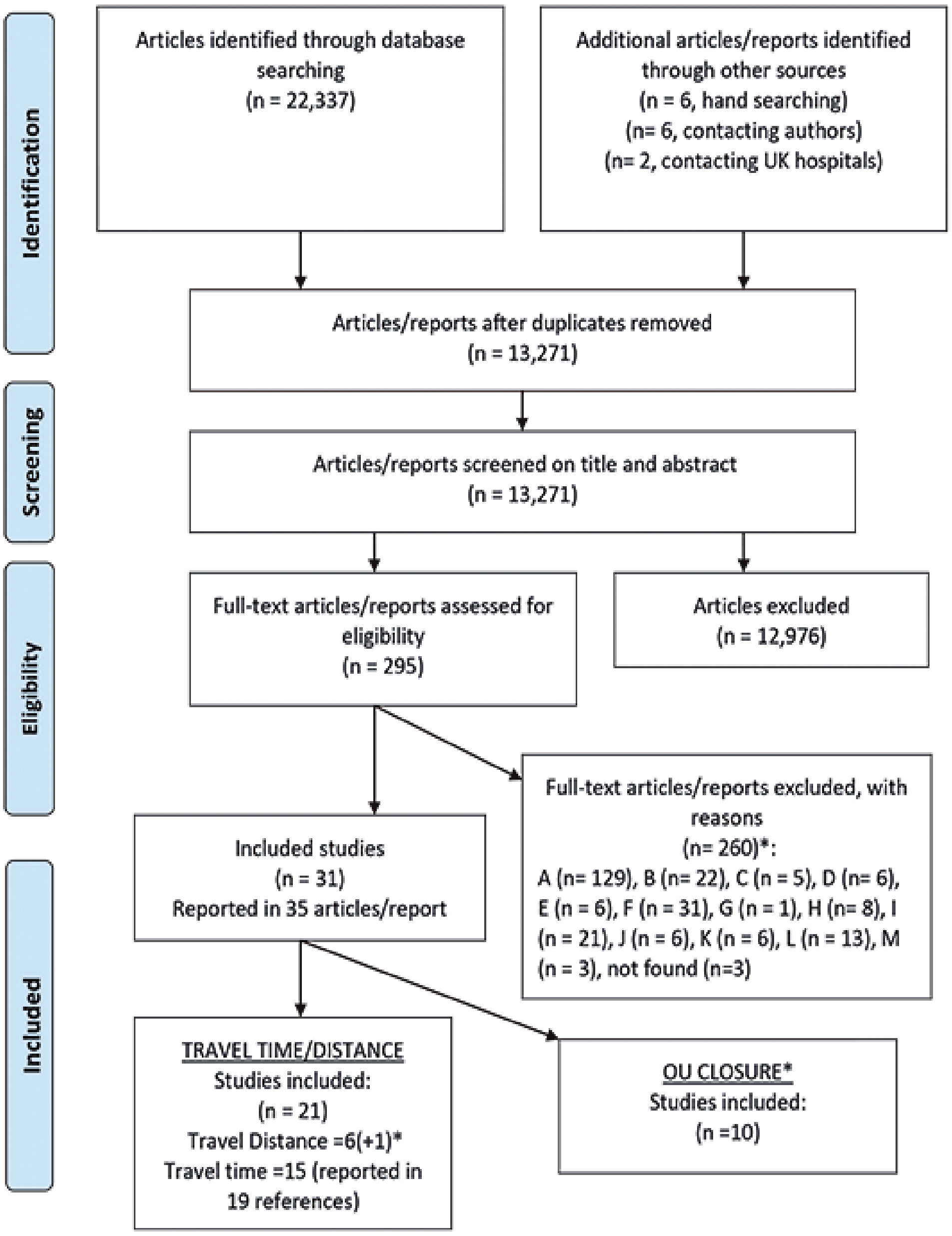

*One study, Blondel 2011 contribute data to both travel distance and closure review questions

From: Moher D, Liberati A, Tetzlaff J, Altman DG, The PRISMA Group (2009). *Preferred Reporting Items for Systematic Reviews and Meta-Analyses*: The PRISMA Statement. PLoS Med 6(7): e1000097. doi:10.1371/journal.pmed1000097

For more information, visit www.prisma-statement.org.

Reasons for inclusion:

A. Different study designs
B. Studies comparing different models of maternity care
C. Studies comparing different hospital volumes
D. Studies comparing different levels of care
E. Studies comparing different hospital types (private vs. public, local vs. central)
F. Transfer from primary to secondary birth centres
G. Cost of care
H. Neonatal transfer
I. Regionalisation
J. Not in high-income countries or in the USA
K. Distance or travel time proximity
L. No outcomes
M. Modelling studies
N. Not found

Appendix 1: Current high-income countries (OECD) members

- Australia
- Austria
- Belgium
- Canada
- Chile
- Czech Republic
- Denmark
- Estonia
- Finland
- France
- Germany
- Greece
- Hungary
- Iceland
- Ireland
- Israël
- Italy
- Japan
- Korea
- Latvia
- Luxembourg
- Mexico
- Netherlands
- New Zealand
- Norway
- Poland
- Portugal
- Slovak Republic
- Slovenia
- Spain

For peer review only

- Sweden
- Switzerland
- Turkey
- United Kingdom
- United States

http://www.oecd.org/about/membersandpartners/

For peer review only

Appendix 2: MEDLINE search results in October 2017

1	Maternal Health Services/	12707
2	Delivery Rooms/	1417
3	Intensive Care Units, Neonatal/ and Intensive Care, Neonatal/	860
4	exp Hospitals/ and (exp Pregnancy/ or exp Delivery, Obstetric/ or Prenatal Care/ or Perinatal Care/ or Postnatal Care/)	10061
5	exp Hospitals/ and (foetal or fetal or foetus or fetus or newborn* or neonat* or infant* or baby or babies or maternal or maternity or pregnant or pregnancy or pregnancies or prenatal or pre-natal or antenatal or ante-natal or perinatal or peri-natal or postnatal or post-natal).ti,ab.	13308
6	(Community Health Services/ or Rural health Services/) and (exp Pregnancy/ or exp Delivery, Obstetric/ or Prenatal Care/ or Perinatal Care/ or Postnatal Care/)	2130
7	(Community Health Services/ or Rural health Services/) and (foetal or fetal or foetus or fetus or newborn* or neonat* or infant* or baby or babies or maternal or maternity or pregnant or pregnancy or pregnancies or prenatal or pre-natal or antenatal or ante-natal or perinatal or peri-natal or postnatal or post-natal).ti,ab.	2436
8	((maternity or obstetric?) adj3 (service? or hospital? or ward? or unit? or department? or dept? or centre? or center?)).ti,ab.	20646
9	((maternity or maternal or maternal health or obstetric) adj care).ti,ab.	8066
10	((delivery or birth*) adj (service? or hospital? or ward? or unit? or department? or dept? or centre? or center?)).ti,ab.	2990
11	((delivery or birth) adj care).ti,ab.	797
12	((foetal or fetal or foetus or fetus or newborn* or neonat* or infant* or baby or babies or maternal or maternity or pregnant or pregnancy or pregnancies or prenatal or pre-natal or antenatal or ante-natal or perinatal or peri-natal or postnatal or post-natal) adj3 (service? or hospital? or ward? or unit? or department? or dept? or centre? or center?)).ti,ab.	49515
13	((foetal or fetal or foetus or fetus or newborn* or neonat* or infant* or baby or babies or maternal or maternity or pregnant or pregnancy or pregnancies or prenatal or pre-natal or antenatal or ante-natal or perinatal or peri-natal or postnatal or post-natal) adj care).ti,ab.	29272
14	(((communit* or rural or district? or local*) adj3 (service? or hospital? or ward? or unit? or department? or dept? or centre? or center?)) and (foetal or fetal or foetus or fetus or newborn* or neonat* or infant* or baby or babies or maternal or maternity or pregnant or pregnancy or pregnancies or prenatal or pre-natal or antenatal or ante-natal or perinatal or peri-natal or postnatal or post-natal)).ti,ab.	7602
15	(((communit* or rural or district? or local*) adj care) and (foetal or fetal or foetus or fetus or newborn* or neonat* or infant* or baby or babies or maternal or maternity or pregnant or pregnancy or pregnancies or prenatal or pre-natal or antenatal or ante-natal or perinatal or peri-natal or postnatal or post-natal)).ti,ab.	141
16	(((level 1 or level i or level one or level 2 or level ii or level two or level 3 or level iii or level three) adj5 (service? or hospital? or ward? or unit? or department? or dept? or centre? or center? or care)) and (foetal or fetal or foetus or fetus or newborn* or neonat* or infant* or maternal or maternity or pregnant or pregnancy or prenatal or pre-natal or antenatal or ante-natal or perinatal or peri-natal or postnatal or post-natal)).ti,ab.	1408
17	1 or 2 or 4 or 5 or 6 or 7 or 8 or 9 or 10 or 11 or 12 or 13 or 14 or 15 or 16	108968
18	regional health planning/ or "catchment area (health)"/	12545

For peer review only

19	"Transportation of Patients"/	8903
20	Transportation/	8928
21	Health Services Accessibility/	65383
22	Travel/	23261
23	(travel* adj5 (distance or time)).ti,ab.	7255
24	("distance to" adj5 (hospital? or service? or unit? or ward? or centre? or center?)).ti,ab.	5849
25	("time to" adj5 (hospital? or service? or unit? or ward? or centre? or center?)).ti,ab.	37417
26	(("travel to" or "travel?ing to") adj5 (hospital? or service? or unit? or ward? or centre? or center?)).ti,ab.	1394
27	(("transfer to" or "transferred to") adj5 (hospital? or service? or unit? or ward? or centre? or center?)).ti,ab.	13476
28	(("transport to" or "transported to" or "transportation to") adj5 (hospital? or service? or unit? or ward? or centre? or center?)).ti,ab.	6259
29	(("access to" or accessibility) adj5 (hospital? or service? or unit? or ward? or centre? or center?)).ti,ab.	22957
30	(access* or travel* or distance* or transport* or transfer*).ti.	360710
31	((regional* or central or decentral*) adj5 (hospital? or service? or unit? or ward? or centre? or center?)).ti,ab.	36894
32	(regionali?e* or regionali?ation or centrali?e* or centrali?ation or decentrali?e or decentrali?ation).ti,ab.	18456
33	(geographic adj5 (hospital? or service? or unit? or ward? or centre? or center?)).ti,ab.	1951
34	((geographic* or district? or residence) adj5 area?).ti,ab.	24860
35	catchment*.ti,ab.	10031
36	((small or medium or large) adj2 (hospital? or unit?)).ti,ab.	14557
37	(small volume or medium volume or high volume).ti,ab.	18240
38	Health Facility Closure/	2317
39	(close or closed or closing or closure? or discontinu*).ti,ab.	609722
40	(preclos* or pre-clos* or postclos* or post-clos*).ti,ab.	406
41	downsiz*.ti,ab.	1966
42	(midwifery led adj2 (service? or unit? or ward? or centre? or center?)).ti,ab.	46
43	(midwifery adj (service? or unit? or ward? or centre? or center?)).ti,ab.	352
44	((consultant led or obstetrician led) adj2 (service? or unit? or ward? or centre? or center?)).ti,ab.	132
45	((consultant or obstetrician) adj (service? or unit? or ward? or centre? or center?)).ti,ab.	159
46	(referral* adj5 (service? or unit? or ward? or centre? or center?)).ti,ab.	28869
47	("model of care" or care model* or "model of delivery" or delivery model* or "model of service" or service model*).ti,ab.	12723
48	18 or 19 or 20 or 21 or 22 or 23 or 24 or 25 or 26 or 27 or 28 or 30 or 31 or 32 or 33 or 34 or 35 or 36 or 37 or 38 or 39 or 40 or 41 or 42 or 43 or 44 or 45 or 46 or 47	1251197
49	17 and 48	15581

For peer review only

Meta-analysis of Observational Studies in Epidemiology (MOOSE) Checklist

The impact of obstetric unit closures, travel time and distance from women's home to obstetric services on maternal and neonatal outcomes: a systematic review

Criteria		Brief description of how the criteria were handled in the meta-analysis
**Reporting of background should include**		
√	Problem definition	Closure of small obstetric units (OUs) and centralisation of obstetric services in larger units has been proposed to increase levels of consultant obstetrician cover to improve safety and limit costs. However, closure of OUs or conversion of OUs to midwifery-led units/community-based services potentially leads to an increase in travel distance or time for women in labour from their home to the nearest obstetric unit (Page 1).
√	Hypothesis statement	Increases in travel time/distance could potentially increase the risk of adverse birth outcomes (Page 1).
√	Description of study outcomes	Maternal and neonatal outcomes.
√	Type of exposure or intervention used	The closure of obstetric units was compared to no closure of obstetric units in the same population.<br>A shorter travel distance or time to OUs was compared with a longer travel distance or time (Page 7&8).
√	Type of study designs used	We included any quantitative study design with a comparison group (Page 6).
√	Study population	We included studies of women giving birth in high-income (OECD) (Page 7).
**Reporting of search strategy should include**		
√	Qualifications of searchers	All authors contributed to developing the search strategy. The information specialist NR has been acknowledged (Page 9).
√	Search strategy, including time period included in the synthesis and keywords	See Medline search strategy in Appendix 2.<br>Search time: 1990 to 2019 (Page 9).
√	Databases and registries searched	We searched: Embase, Medline, PsycINFO, Applied Social Science Index and Abstracts (ASSIA) and Cumulative Index to Nursing and Allied Health (CINAHL) databases. We also searched British Library EThOS, Open Grey and ProQuest Dissertations & Theses Global (Page 9).
√	Search software used, name and version, including special features	OvidSP, We detailed the MeSH/ Emtree Headings and text words in Appendix 2 (Page 9).
√	Use of hand searching	Bibliographies of the included papers were hand searched for additional references (Page 9).
√	List of citations located and those excluded, including justifications	Details of the literature search process are outlined in the PRISMA flow chart (Figure 1). The citation list of excluded articles is available from authors upon request (Page 11).

For peer review only - http://bmjopen.bmj.com/site/about/guidelines.xhtml

√	Method of addressing articles published in languages other than English	We placed no restrictions on language. Studies published in languages other than English were translated into English and included in the review if they fulfilled the review inclusion criteria (Page 9).
√	Method of handling abstracts and unpublished studies	We contacted a number of authors for full report of relevant unpublished studies (Page 10).
√	Description of any contact with authors	We contacted authors of relevant articles for full report of their unpublished studies. NHS Trusts and Health Boards in the UK were also contacted where we had been able to identify an OU closure. For details of the authors who provided extra unpublished information, see the acknowledgments section (Page 29).
**Reporting of methods should include**		
√	Description of relevance or appropriateness of studies assembled for assessing the hypothesis to be tested	Detailed inclusion and exclusion criteria are described in tables 1A (Page 31 – 36), 2A (Page 44 – 51) and 3A (Page 62 – 69).
√	Rationale for the selection and coding of data	A data extraction form was developed (available on request) to extract data related to characteristics of the study participants, time and distance measured, outcomes reported and quality assessment (Page 9&10).
√	Assessment of confounding	We have highlighted where crude (unadjusted) odds ratios (cOR) and adjusted odds ratios (adj OR) have been reported (Page 10). However, data were adjusted for different confounding factors across the included studies.
√	Assessment of study quality, including blinding of quality assessors; stratification or regression on possible predictors of study results	We used a modified version of the Effective Practice and Organisation of Care (EPOC) and the risk of bias in non-randomised studies of interventions Newcastle-Ottawa scale (NOS) (for case-control studies). No meta-analysis was feasible therefore; we were unable to conduct any sensitivity analysis to compare results from only low risk of bias studies (Page 10).
√	Assessment of heterogeneity	NA
√	Description of statistical methods in sufficient detail to be replicated	We found the included studies varied in their study design, categories of exposure, outcomes reported, whether adjusted or unadjusted results were presented and factors adjusted for. Due to this clinical and methodological variability quantitative pooling data was not feasible (Page 10).
√	Provision of appropriate tables and graphics	We included PRISMA flow chart to show the method of studies identification.<br>Table 1A: Description of included studies - Obstetric unit closures (Page 31 – 36).<br>Table 1B: Risk of bias - Obstetric unit closure studies (Page 37 – 39).<br>Table 1C: Outcomes - Obstetric unit closure (Page 40 – 47).<br><br>Table 2A: Description of included studies - Travel distance (Page 47 – 51).

For peer review only - http://bmjopen.bmj.com/site/about/guidelines.xhtml

		Table 2B: Risk of bias - Travel distance (Page 51 – 55).<br>Table 2C: Outcomes - Travel distance (Page 56 – 61).<br><br>Table 3A: Description of included studies - Travel time (Page 62 – 69).<br>Table 3B: Risk of bias - Travel time (Page 70 – 74).<br>Table 3C: Outcomes - Travel time (Page 75 – 90).
**Reporting of results should include**		
√	Graph summarizing individual study estimates and overall estimate	NA
√	Table giving descriptive information for each study included	Table 1A (Page 31 – 36), 2A (Page 47 – 51), 3A (Page 62 – 69).
√	Results of sensitivity testing	NA
√	Indication of statistical uncertainty of findings	The 95% confidence interval (CI) is reported to estimate the precision of the ORs and RRs in table 1C (Page 40 -47), 2C (Page 56 – 61) and table 3C (Page 75 – 90).
**Reporting of discussion should include**		
√	Quantitative assessment of bias	
√	Justification for exclusion	Reasons for exclusion were reported in PRISMA flow chart.
√	Assessment of quality of included studies	All presented in details in table 1B (Page 37 – 39), 2B (Page 51 – 55) and 3B (Page 70 – 74).
**Reporting of conclusions should include**		
√	Consideration of alternative explanations for observed results	Results were inconsistent across studies and no meta-analysis was feasible (Page 26).
√	Generalization of the conclusions	We reported the fact that almost all of the included studies were from Europe. (Page 26).
√	Guidelines for future research	We highlighted the need to use routinely collected data. In addition to the need of applying, a standardized way of measuring travel time and distance from woman's place of residence to an OU. In addition to applying a statistical method which considers time-varying relationships and the outcomes (Page 28).
√	Disclosure of funding source	This research is funded by the National Institute for Health Research (NIHR) Policy Research Programme, conducted through the Policy Research Unit in Maternal Health and Care, 108/0001 (Page 29).

For peer review only - http://bmjopen.bmj.com/site/about/guidelines.xhtml
